# STD NMR Epitope Perturbation by Mutation Unveils the Mechanism of YM155 as an Arginine-Glycosyltransferases Inhibitor Effective in Treating Enteropathogenic Diseases

**DOI:** 10.1021/jacsau.4c01140

**Published:** 2025-03-05

**Authors:** Jonathan Ramírez-Cárdenas, Víctor Taleb, Valeria Calvaresi, Weston B. Struwe, Samir El Qaidi, Congrui Zhu, Kamrul Hasan, Yingxin Zhang, Philip R. Hardwidge, Billy Veloz, Juan C. Muñoz-García, Ramón Hurtado-Guerrero, Jesús Angulo

**Affiliations:** † Instituto de Investigaciones Químicas (CSIC—Universidad de Sevilla), 49 Américo Vespucio Street, Sevilla 41092, Spain; ‡ Institute of Biocomputation and Physics of Complex Systems, University of Zaragoza, Mariano Esquillor s/n, Campus Rio Ebro, Edificio I+D, Zaragoza 50018, Spain; § Department of Biochemistry, University of Oxford, Oxford OX1 3QU, U.K.; ∥ The Kavli Institute for Nanoscience Discovery, University of Oxford, Dorothy Crowfoot Hodgkin Building, South Parks Road, Oxford OX1 3QU, U.K.; ⊥ College of Veterinary Medicine, Kansas State University, Manhattan, Kansas 66506, United States; # Copenhagen Center for Glycomics, Department of Cellular and Molecular Medicine, University of Copenhagen, Blegdamsvej 3B, Copenhagen 2200, Denmark; ¶ Fundación ARAID, Zaragoza 50018, Spain

**Keywords:** enteropathogenic arginine-glycosyltransferases, arginine *N*-glycosyltransferase inhibition, STD NMR epitope perturbation by mutation, enzyme inhibitors

## Abstract

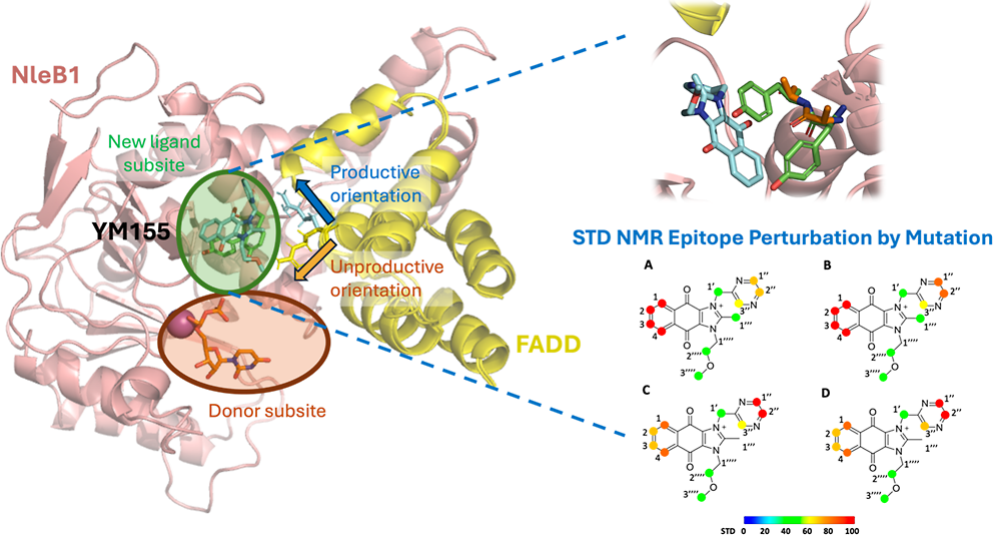

Enteropathogenic arginine-glycosyltransferases (Arg-GTs) alter higher eukaryotic proteins by attaching a GlcNAc residue to arginine acceptor sites, disrupting essential pathways such as NF-κB signaling, which promotes bacterial survival. These enzymes are potential drug targets for treating related diseases. In this study, we present a novel STD NMR Epitope Perturbation by Mutation spectroscopic approach that, in combination with hydrogen–deuterium exchange mass spectrometry (HDX-MS), and molecular dynamics simulations, shows that the highly potent broad-spectrum anticancer drug YM155 serves as a potential noncompetitive inhibitor of these enzymes. It induces a conformation of the arginine acceptor site unfavorable for GlcNAc transfer, which underlies the molecular mechanism by which this compound exerts its inhibitory function. Finally, we also demonstrate that YM155 effectively treats enteropathogenic diseases in a mouse model, highlighting its therapeutic potential. Overall, our data suggest that this compound can be repurposed to not only treat cancer but also infectious diseases.

## Introduction

Glycosyltransferases (GTs) are enzymes responsible for the transfer of sugar moieties to a larger myriad of potential acceptor substrates and serve as crucial determinants of biological functions.^1,2^ Among these, arginine-GTs (Arg-GTs) from enteropathogens stand out for their role in bacterial virulence, and represent a novel class of enzymes that subvert host immune responses by modifying key regulatory proteins.^2,3^ Arg-GTs such as NleB1, found in both enterohemorrhagic and enteropathogenic *Escherichia coli* (EHEC and EPEC), along with NleB from *Citrobacter rodentium* and their *Salmonella enterica* orthologs SseK1, SseK2, and SseK3, are distinctive for their ability to attach an *N*-acetylglucosamine (GlcNAc) moiety to the arginine residues of host proteins.^2,4,5^ This posttranslational modification does not naturally occur within mammalian systems, indicating a sophisticated bacterial strategy to disrupt host cellular processes.^2,4,5^ Additionally, both EHEC and EPEC strains produce another NleB1 paralogue, NleB2, which adds a glucose moiety instead of a GlcNAc.^6^ It has been discovered that these Arg-GTs can also glycosylate proteins from the original bacteria that produce them.^7,8^

At the structural level, these enzymes show a high degree of similarity and are built by two conserved major domains and a C-terminal lid, which is also required for the catalytic activity of the enzyme. The GT-A fold-adopting catalytic domain is the largest domain and includes the essential DxD and HEN (His–Glu–Asn) motifs. The helix–loop–helix (HLH) domain comprises two helices connected by a loop.^9^

At the kinetic level, Arg-GTs have been characterized to follow an ordered kinetic mechanism in which UDP-GlcNAc induces a conformational change that closes the C-terminal lid, thereby forming the enzyme’s active state.^3^ Studies utilizing the death domains of receptor-interacting serine/threonine-protein kinase 1 (RIPK1), receptor-associated death domain protein (TRADD), and Fas-associated death domain protein (FADD) as acceptor substrates have revealed that these enzymes function as inverting glycosyltransferases. A conserved Glu residue is likely to serve as the catalytic base, facilitating the deprotonation of the acceptor Arg.^3,10^ Intriguingly, when suboptimal peptide substrates derived from these death domains are employed, the enzymes can alternatively follow a retaining mechanism.^9^

The glycosylation of Arg residues by these Arg-GTs originated from a sophisticated evolutionary adaptation aimed at interfering with the host cell physiological processes, and is pivotal in the context of infections, underscoring the complex interplay between pathogenic bacteria and their human hosts. Bacterial pathogens leverage such enzymes to modify key signaling proteins, such as receptor-interacting serine/threonine-protein kinase 1 (RIPK1), tumor necrosis factor receptor-associated death domain protein (TRADD), glyceraldehyde 3-phosphate dehydrogenase (GAPDH) and Fas-associated death domain protein (FADD).^4,5^ The alterations in these proteins function disrupt critical pathways like NF-κB signaling, crucial for the initiation and regulation of the immune response.^4,5,11^

Hence, the identification of inhibitors targeting Arg-GTs could be a game-changer in treating diseases caused by enteropathogens. Early research yielded promising candidates like compounds 100066N and 102644N,^12^ which inhibited the activity of these enzymes. Yet, the limited availability and solubility issues of these compounds prompted us to discover sepantronium bromide (YM155), a more soluble compound that also inhibited the function of these Arg-GTs.^12^ However, the exact mechanism by which YM155 inhibits these enzymes, and its viability as a treatment for such diseases, remain to be clarified. This compound is primarily recognized for its role in cancer therapy by inhibiting survivin expression and has also been shown to interact with receptor-interacting protein kinase 2.^13^ Herein, we have combined multiple techniques such as STD NMR spectroscopy, hydrogen–deuterium exchange mass spectrometry (HDX-MS), and molecular dynamics simulations to demonstrate that YM155 serves as a potential noncompetitive inhibitor by conformationally rearranging the acceptor Arg residue in the quaternary NleB1/UDP/YM155/FADD complex, thereby inhibiting the enzyme. Furthermore, we demonstrate that YM155 significantly reduces *C. rodentium* infection in a mouse model of disease, thereby supporting its potential application in the treatment of enteropathogenic diseases.

## Results and Discussion

### Structural Information on the Interaction of the Ligand YM155 with NleB1^WT^ from STD NMR: Binding Epitope Mapping

**Figure 1:**
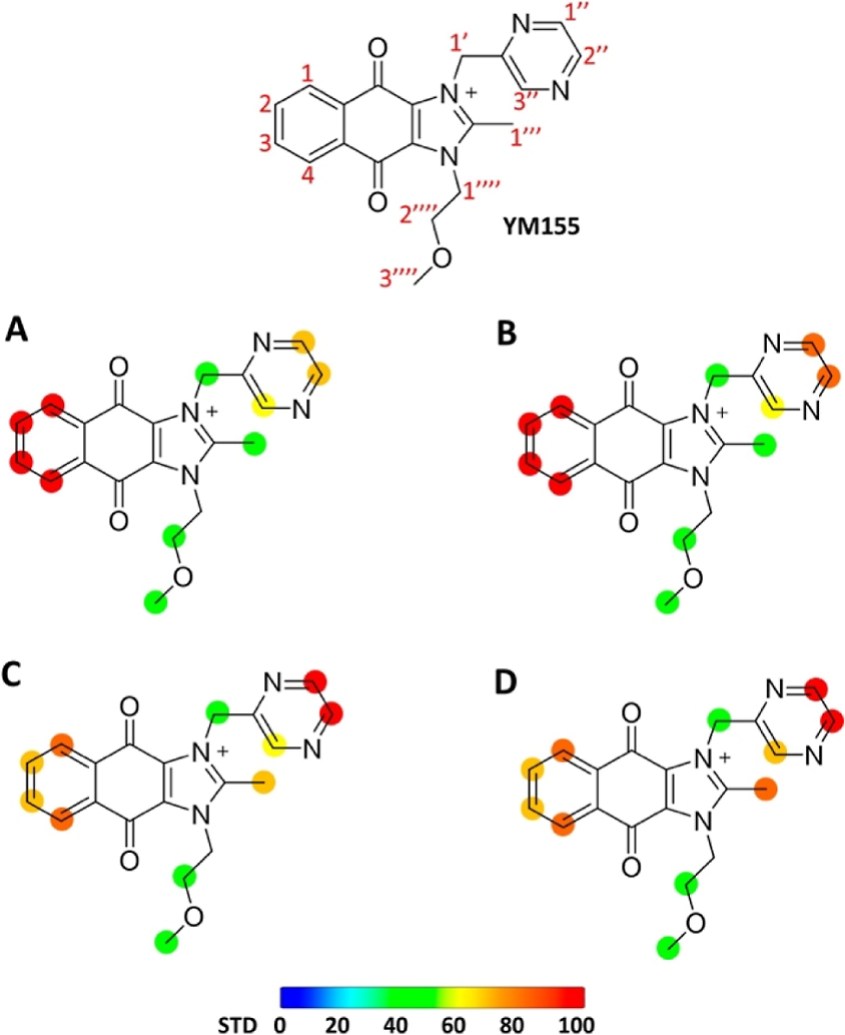
Binding epitope mappings of YM155 interacting with NleB1, NleB1 single mutants, and SseK2. STD initial slopes from 1D ^1^H STD NMR were used to study the binding epitope map of YM155 upon interaction with (A) NleB1^WT^, (B) NleB1^Y283A^, (C) NleB1^Y284A^ and (D) SseK2^WT^. Protein saturation was achieved by irradiation at 0.5 ppm. The colored spheres represent normalized STD NMR intensities. The largest STD initial slope among ligand protons was assigned 100% and the epitope was determined by normalizing the rest of values against that one in a percentage scale (see Table S1). For simplicity, the colored spheres are placed on the carbon atoms.

1D ^1^H STD NMR spectroscopy is a very powerful technique to gain structural information on weak/medium affinity protein–ligand interactions, reporting on the spatial contacts of a ligand in the binding site of the receptor. The resulting analysis produces the so-called binding epitope maps that provides information on the binding mode of the ligand. The technique works generally well for micro-to millimolar *K*_D_ interactions. We first used STD NMR experiments to analyze the interaction of YM155 with NleB1^WT^ in solution.^14,15^ The strong STD NMR signals observed indicated that the binding of YM155 to NleB1^WT^ takes place with a kinetics appropriate for a sensitive analysis by the STD NMR technique. We then analyzed the full build–up curves from 1D ^1^H STD NMR experiments (Figure S1), obtaining the corresponding STD initial slopes (STD_0_) for each measurable ligand proton, which allowed us to obtain the experimental binding epitope mapping depicting the main spatial contacts of the ligand YM155 with NleB1^WT^ in the bound state (Figure 1A).

The binding epitope map of YM155 for its interaction with NleB1^WT^ (Figure 1A) shows that both aromatic ends of the YM155 ligand are establishing the closest contacts with the protein in the bound state. This supports a binding mode of YM155 where both aromatic rings are buried in the enzyme binding pocket. The strongest relative STD values are observed for the phenyl ring, indicating that this residue makes closest contacts with the protein in comparison with the pyrazine ring at the other end. The central region of YM155 as well as the ether side chain show residual contacts, indicating that this area is farther from the surface of the protein, and most likely more solvent exposed.

### STD NMR Epitope Perturbation by Mutation for Localization of the Ligand Binding Site on NleB1: Y283 and Y284 are Key Side Chains for the YM155-NleB1^WT^ Interaction

1D ^1^H STD NMR spectroscopy cannot provide direct information on the localization of the binding pocket in the protein surface where the interaction with the ligand takes place, although methods have been developed to gain information on the nature of the amino acid side chains contacting the ligand.^16^ Here, we decided to carry out a novel approach to test whether binding of YM155 takes place in the NleB1 active site: the analysis of STD NMR experiments carried out on single mutants of key amino acids in the NleB1 enzyme active site. In a previous study, we had demonstrated that NleB substrate selectivity is strongly determined by a second shell residue, Y284, contiguous to the catalytic machinery.^3^ The residue Y284 is key to couple protein substrate binding to catalysis, where CH-π interactions between the side chains of Y284 and Y283 assist proper accommodation of the side chain of the acceptor residue R117^FADD^ in the active site with a suitable orientation toward the catalytic base E253. Due to the hydrophobic character of those two residues, we hypothesized that if the binding of the inhibitor YM155 takes place in the NleB1 active site, it should involve contacts with one or both tyrosine side chains. We then assessed this by producing two single NleB1 mutants (Y283A and Y284A) for which binding to YM155 was investigated again by STD NMR experiments.

As anticipated, given the strategic location of the two Tyr residues at the enzyme-acceptor substrate interface, mutating these residues to Ala led to a moderate reduction in activity under saturated substrate conditions, retaining approximately 40% of the wild-type enzyme activity (Figure S2). Binding of YM155 to both Y283A and Y284A NleB1 mutants was detectable by using STD NMR, indicating that the interaction is not completely abolished by any of the single mutations. This is a key result for our investigations, as it indicates that even if affinity is affected by a mutation (most likely being reduced), as long as the interaction still takes place, STD NMR spectroscopy is able to detect it due to its high sensitivity for weak/medium affinity interactions. It is in these cases where the analysis of the impact of the different mutations on the intensities of the ligand STD NMR signals can be used to confirm binding in the proximity of the mutated residues.

**Figure 2:**
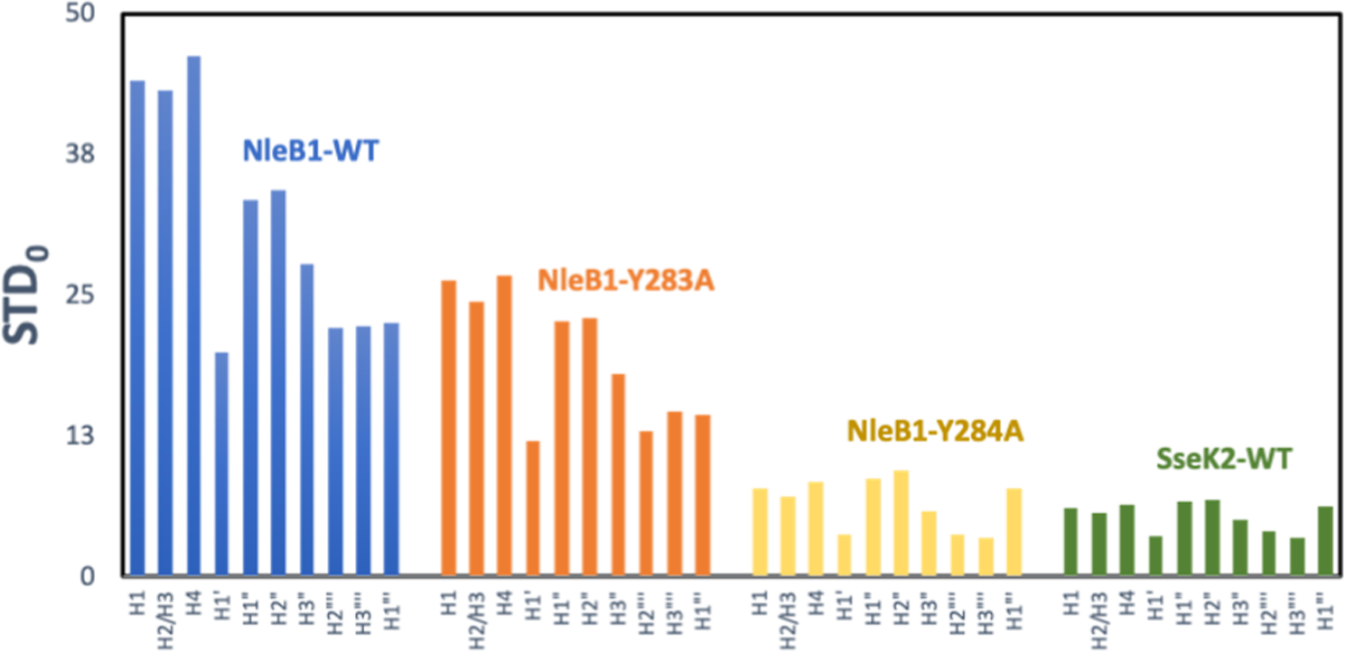
STD NMR Initial slope values (STD_0_) of the binding of YM155 with NleB1^WT^, NleB1^Y283A^, NleB1^Y284A^ and SseK2^WT^. Initial slopes were obtained from STD NMR build-up curves for each proton of YM155 (Figure S1). Note that, to probe differences in STD NMR responses of the different mutants, STD_0_ values are not normalized as in Figure 1. In blue: STD_0_ values for the binding of NleB1^WT^ with YM155. In orange: STD_0_ values for the binding of NleB1^Y283A^ with YM155. In gray: STD_0_ values for the binding of NleB1^Y284A^ with YM155. In yellow: STD_0_ values for the binding of SseK2^WT^ with YM155. STD_0_ has units of s^–1^.

In fact, the STD NMR experiments with both mutants clearly showed significant impacts on the STD NMR signals. First, in comparison to the experiments with NleB1^WT^, the interaction with the mutant NleB1^Y283A^, under the same experimental conditions, led to a significant reduction in the intensities of the STD NMR signals of YM155 (Figure 2). However, the binding epitope map was similar to that observed for the interaction with the wild-type enzyme (Figure 1B). This result strongly supports that the Y283A mutation impacts mostly the affinity of the interaction but does not significantly affect the ligand binding mode.

On the other hand, a much stronger intensity reduction of the STD NMR signals was observed for the interaction with the NleB1^Y284A^ mutant (Figure 2), in comparison to NleB1^WT^. Additionally, this intensity reduction was also accompanied by a significant impact on some parts of the ligand binding epitope map (Figure 1C). This result supports that the Y284A mutation affects both the binding affinity for YM155 and the contacts of the protein with the ligand in the bound state, as a result of the removal of the bulky aromatic side chain of Y284. The STD NMR Epitope Perturbation by Mutation experiments indeed provided structural hints on the orientation of the YM155 ligand in the binding pocket, as the Y284A mutation affects more significantly the protons of the phenyl ring of YM155. This result supports that this residue is most likely close to the tyrosine aromatic side chain in the bound state for the wild type enzyme. Nevertheless, the internal dynamics of both side chains (residues 283 and 284) and the ligand in the bound state precludes an accurate disentangling of the specific contributions of the protons of each aromatic side chain to the observed perturbation. Taken together, the STD NMR Epitope Perturbation by Mutation analysis unambiguously shows that the side chains of Y283 and Y284 are key players for the interaction of NleB1 with the ligand YM155.

### Competition and STD NMR Epitope Perturbation by Mutation Experiments Show That YM155 Binds to a Novel Subsite Adjacent to the Acceptor Site

The previous STD NMR Epitope Perturbation by Mutation analysis prompted us to investigate further where the YM155 interaction takes place within the NleB1 enzyme active site. To that aim, we first carried out competition studies by STD NMR experiments between YM155 and different nucleotides: UDP-GlcNAc (donor), UDP (product of the reaction), or UDP-GalNAc (epimer of the donor) and MgCl_2_ with NleB1^WT^.^17^ Note that both MgCl_2_ and MnCl_2_ have been utilized with these enzymes; MnCl_2_ was found to be slightly more effective in binding to UDP-GlcNAc than MgCl_2_, demonstrating that these metals play similar roles in stabilizing the donor substrate,^18^ but we avoided the use of the Mn^2+^ ion in the STD NMR spectra to prevent the large paramagnetically induced broadening of the NMR signals. All the tested nucleotides bind NleB1 as they produced clear STD NMR signals in the presence of the enzyme. On the other hand, the study confirmed that there is no competition between YM155 and any of the nucleotides (Tables S2–S4). Additionally, competition studies between YM155 and FADD (natural protein ligand acceptor of NleB1) for their binding to NleB1^WT^ also indicated that there is no competition between YM155 and FADD for binding to NleB1^WT^. FADD needs UDP to bind to the enzyme,^3^ so the experiment with FADD was performed in the presence of an excess of UDP and MgCl_2._

The absence of donor- or acceptor-site competition pointed toward YM155 binding to a novel subsite, which, nonetheless, should be adjacent to the acceptor site, as Y283 and Y284 were shown to be important for the interaction of NleB1 with YM155. From this result, we next moved forward by exploring potential binding sites adjacent to the acceptor site using Schrödinger^19^ (see Materials and Methods). After conducting a comprehensive analysis of possible binding sites at NleB1^WT^, we identified a novel potential site (*Site-2*, Figure S3) adjacent to the acceptor site, which involves the side chains of Y283 and Y284.

Docking calculations were then performed at *Site-2* to generate energetically favorable 3D molecular models of the complex between NleB1^WT^ and YM155. The best 3D model of the complex was selected based on its quantitative validation against the experimental STD NMR data by using RedMat, a new software capable of predicting theoretical STD NMR binding epitope maps from both static (docking simulations) and dynamic (MD simulations) 3D models of protein–ligand complexes.^20^ RedMat analysis revealed that the agreement with STD NMR experimental data was excellent for two models (11 and 12, Table S5), which showed NOE *R*-factors below 0.3. Model 12 was chosen as it was the energetically most favorable docking solution.

**Figure 3:**
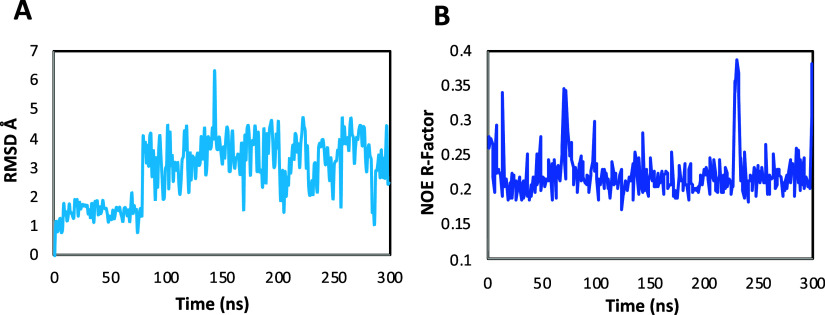
MD simulation and NMR validation of the complex of YM155 with NleB1. (A) Evolution of the root mean squared deviation (RMSD) of the YM155 ligand (all atoms except protons) with respect to the protein binding site (residues within 5 Å from the ligand). (B) Evolution of the NOE *R*-factor of YM155 ligand over the 300 ns MD simulation.

We next studied the stability and dynamics of the 3D molecular model of YM155-NleB1 complex, through a 300 ns molecular dynamics (MD) simulation. A trajectory analysis based on the root mean squared deviation (RMSD) of the YM155 ligand heavy atoms with respect to the protein binding site (residues within 5 Å from the ligand) showed that the 3D model of the complex is stable, with a modest amount of mobility observed at the binding site (Figure 3A). Additionally, by using RedMat, we analyzed the agreement between the ensemble of 3D models of the complex generated by the MD simulation and the experimental STD NMR based binding epitope mapping. This was done by monitoring the evolution of the NOE *R*-factor throughout the entire MD trajectory (Figure 3B).

**Figure 4:**
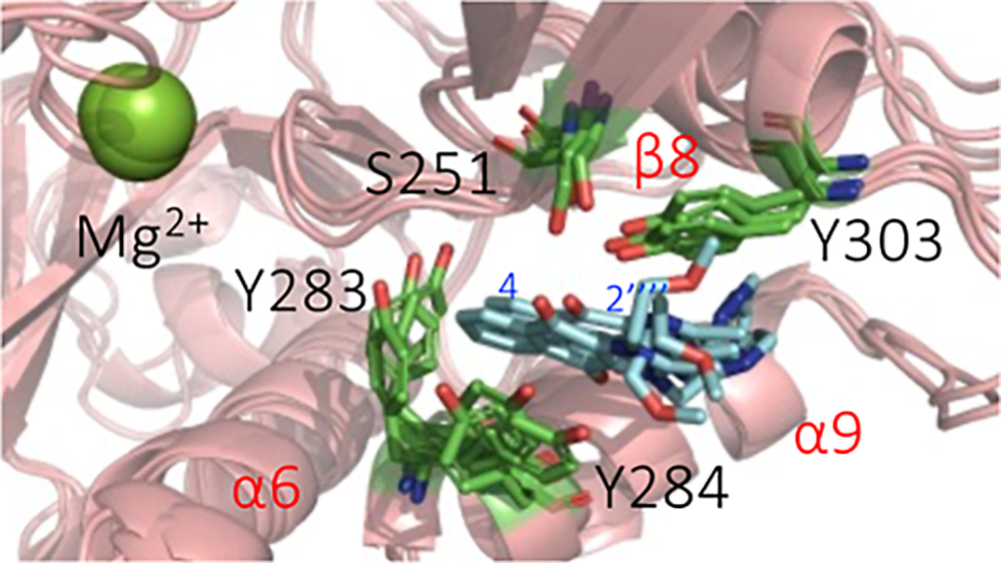
NMR and HDX-MS validated structures of the complex of YM155 with NleB1 from MD simulations. Superposition of 3 frames from the MD simulation of the binding of NleB1^WT^ with YM155. NleB1^WT^ is shown in salmon colored cartoon. YM155 is shown in cyan sticks. Y283 and Y284 are shown in green sticks.

Most of the resulting MD frames exhibited NOE *R*-factor values less than 0.3, indicating good agreement between the 3D molecular model of the complex and the experimental STD NMR binding epitope data. To visualize the structural features of the YM155-NleB1 complex, several frames of the MD trajectory were extracted. In these structures, stabilizing π–π stacking and CH–π interactions between YM155 and the three tyrosines (Y283, Y284 and Y303) were observed. Stabilizing hydrogen bonds between YM155 and residues Y303 and S251 were also identified (Figure 4).

### HDX-MS Unveils the Conformational Impact of YM155 Binding to NleB1 and SseK2

We employed HDX-MS experiments to shed light on the mechanism of inhibition of YM155 at the protein conformational level. HDX-MS provides peptide-level structural dynamics information based on the exchange rate of backbone amide hydrogens with deuterium atoms in solution.^21^ Here, HDX-MS experiments were carried out by comparing NleB1 and SseK2 proteins under apo and YM155-bound states to pinpoint conformational differences stemming from direct binding and binding-induced allosteric effects.

**Figure 5:**
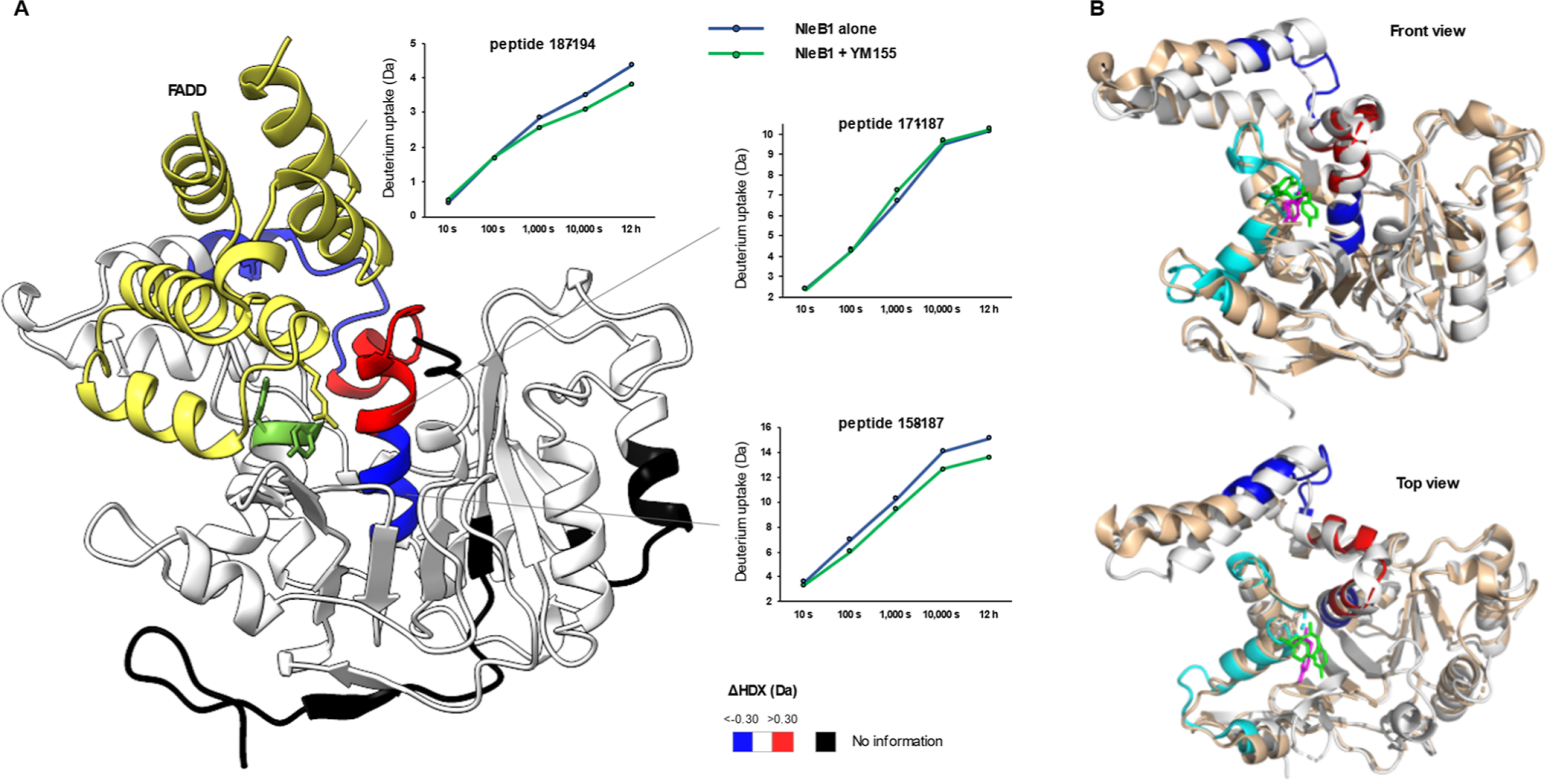
HDX binding fingerprints of YM1555 to NleB1 and SseK2. (A) Differences in HDX between apo- and YM155-bound NleB1 are superimposed into the structure of NleB1 in complex with FADD death domain (PDB: 6ACI). NleB1 residues Y283 and Y284 are colored in green; FADD is colored in yellow. HDX effects spanning region 159–187 are colored in blue when the HDX was observed to decrease and in red when the HDX was observed to increase. Deuterium uptake plot of representative peptides spanning this region are shown. (B). HDX effects generated upon binding of YM155 to NleB1 (blue and red colors) and SseK2 (cyan color) are superimposed on the aligned structures of NleB1 (PDB 6E66, orange color) and SseK2 (PDB 5H62, white color). NleB1 residues Y283 and Y284 are colored in green, SseK2 residue Y301 is colored in magenta.

To note, protein HDX monitored by bottom-up MS (the workflow utilized here) is able to highlight changes affecting the protein backbone amides, but cannot detect changes of the amino acid side-chains because of their inevitable loss of deuterium labels.^21^ Compared to apo NleB1, NleB1 bound to YM155 showed a strong decrease in HDX in the region spanning residues 159–171 (−20%) and 188–194 (−19%), and a minor increase in HDX (+5%) in the region spanning residues 172–187. No HDX effect was detected at Y283 and Y284, suggesting their association to YM155 could occur through side chains (Figures 5A and S4, S5, and S11). Strikingly, the 181–194 segment comprises a helix that is located in front of both Y283–Y284 of NleB1 and the acceptor R117 of FADD, indicating that significant conformational changes observed in this helix are likely contributing to the reorientation of the acceptor side chain R117^FADD^ upon YM155 binding. Furthermore, we performed a comparative HDX-MS experiment between apo and YM155-bound SseK2 (Figures S6–S8 and S12) and observed a decrease in HDX in the region spanning residues 292–321 (+33%). This region comprises Y301, which aligns to Y283 and Y284 in NleB1. We did not observe significant conformational changes in the aforementioned helix yet detected an increased HDX (+20%) in the 77–83 segment. This could indicate a different binding mode of YM155 to SseK2 in respect to NleB1, but this requires further validation. Although the HDX binding fingerprints of YM155 to NleB1 and SseK2 are different (likely due to a different in conformational dynamics between the two proteins and/or a different ligand binding pose), superimposing the HDX effects on the aligned structures of NleB1 and SseK2 (Figure 5B) shows an overall conformational landscape where Y283–Y284 (NleB1) and Y301 (SseK2) can be the pivotal residues for the interaction to YM155 and that as well explains how conformational effects could be transmitted from these residues to R177^FADD^.

### STD NMR Study of the Interaction of YM155 with SseK2^WT^ Confirms the Validity of the STD NMR Epitope Perturbation by Mutation Approach

We previously demonstrated that enzyme effectors NleB1^WT^, NleB, SseK1, and SseK2 are all inhibited by YM155.^12^ Interestingly, in comparison to NleB1^WT^, SseK2^WT^ retains the Tyr residue at position Y283 found in but features an Asn residue at position Y284 instead,^3^ constituting a natural mutant at position 284 related to that position in NleB1^WT^. STD NMR experiments were then conducted to investigate the binding of YM155 to SseK2^WT^. A 1D ^1^H STD NMR experiment was performed whereby, in comparison to the binding study with NleB1^WT^, perturbations of STD NMR intensities of YM155 were indeed observed. The perturbations manifested as a notable impact on the epitope mapping (Figure 1D) and a significant decrease in STD intensities (Figure 2), similar to the observed impact in the single mutant NleB1^Y284A^. The reproducibility of the impact on the binding epitope mapping of the mutation (either artificially introduced or naturally occurring), supports the validity of the STD Epitope Perturbation by Mutation NMR approach to provide information on the location of the ligand along the protein 3D structure.

### Molecular Dynamics Suggests that YM155 Binding Induces a Reorientation of the Acceptor Side Chain R117F^ADD^, Potentially Leading to Noncompetitive Inhibition

The STD NMR competition experiments showed that YM155 is able to form a quaternary complex in solution with NleB1^WT^, FADD, UDP, and YM155, raising the question on what inhibitory mechanism YM155 follows. We first decided to assess the dynamics stability of the quaternary complex. To that aim, a 300 ns MD simulation was performed, revealing that the quaternary complex remained stable throughout the entire MD trajectory.

**Figure 6:**
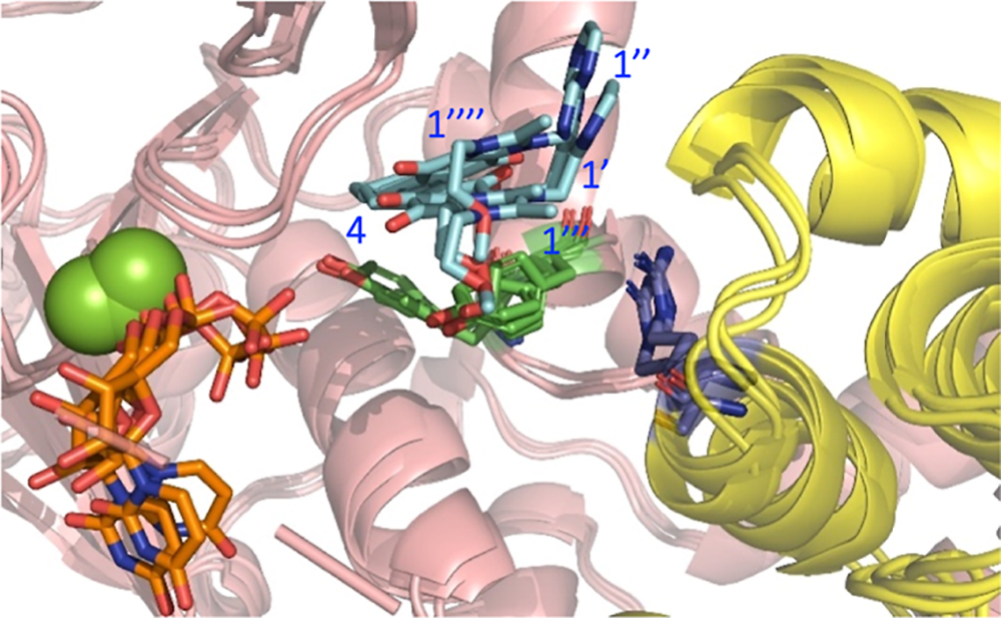
Molecular dynamics structures of the quaternary complex of NleB1/UDP/YM155/FADD. Superposition of 3 frames of the MD simulation of the quaternary complex. NleB1^WT^ and FADD are shown in salmon and yellow colored cartoon, respectively. YM155 is shown in cyan sticks. UDP is shown in orange sticks. The acceptor R117 is shown un purple sticks.

As previously reported,^3^ Y284 is a second-shell residue with respect to the catalytic machinery that controls the orientation of the side chain of R117^FADD^ for its proper interaction with the catalytic base E253. A comprehensive analysis of these two residues along the MD trajectory of the quaternary complex revealed a reorientation of Y284 due to the presence of YM155 in *Site-2*, which simultaneously caused a reorientation of the side chain of the key acceptor residue R117^FADD^ (Figure 6).

The orientation of R117^FADD^ relative to the HEN motif is crucial for the catalytic process to occur.^9^ The observed reorientation of this residue in the presence of YM155 suggests a disruption of productive binding, potentially leading to noncompetitive inhibition of the glycosylation process. This may explain YM155’s inhibitory effect on these enzymes (see the comparison of the dynamics of the R117^FADD^ side chain with and without YM155 in the molecular dynamics movie in the Supporting Information). Note that we previously demonstrated that Y284 is optimal for binding and catalysis. However, orthologs such as SseK1, SseK2, and SseK3 feature alternative residues—Ser, Asn, and Ile—instead of Y284, which are less effective for both binding and catalysis.^3^ Based on our findings with SseK2, we hypothesize that SseK1 and SseK3 may also bind poorly to YM155.

### YM155 Effectively Treats Enteropathogenic Diseases in a Mouse Model

We tested the efficacy of YM155 in treating or preventing the infection of mice by *C. rodentium*, a natural pathogen of mice that expresses NleB and is often used as an animal model of EPEC infections. We infected five-week-old C57BL/6 mice with 1.0 × 10^9^ CFU of *C. rodentium* and treated the mice with YM155 (0.5 mg/kg) either 10 min before infection (preinfection) or 24 h postinfection.

**Figure 7:**
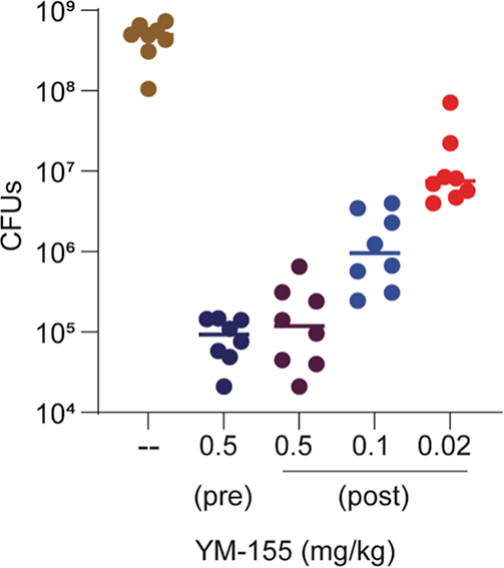
Citrobacter infections. Mice were infected with 1.0 × 10^9^ CFU of *C. rodentium* and treated with YM155 (0.5 mg/kg) either 10 min before infection (preinfection) or 24 h postinfection. Dose de-escalation studies were also performed using YM155 at 0.1 mg/kg and 0.02 mg/kg. Mice were euthanized 7 d after infection and *C. rodentium* CFUs in the intestine were enumerated. Asterisks indicate significantly different CFUs as compared to untreated mice, *p* < 0.05, Dunn’s.

We observed that both the pre- and postinfection treatment conditions significantly reduced the burden of *C. rodentium* in the colon 7 days postinfection (Figure 7), suggesting that YM155 inhibition of NleB in vivo was effective in reducing *C. rodentium* virulence. We also performed dose de-escalation studies in mice treated with reduced concentrations of YM155 24 h postinfection and observed that 0.1 mg/kg YM155, but not 0.02 mg/kg YM155 significantly reduced pathogen burdens 7 days postinfection.

## Conclusions

In this study, we present a novel STD NMR Epitope Perturbation by Mutation method which, in the context of a multidisciplinary approach combining binding epitope maps from STD NMR, hydrogen–deuterium exchange mass spectrometry (HDX-MS), and molecular dynamics (MD) simulations, provides compelling evidence that the anticancer drug YM155 inhibits the arginine-glycosyltransferase activity of *E. coli* NleB1 and its *S. enterica* orthologues in a potentially noncompetitive manner by targeting a previously unknown subsite adjacent to the acceptor substrate site. The interaction of YM155 with NleB1 in the novel subsite induces an unproductive conformation of the side chain of the FADD acceptor Arg that ultimately leads to enzyme inhibition. Significantly, our results demonstrate the efficacy of YM155 in treating enteropathogenic diseases, as shown by its capability to inhibit *Citrobacter* infection in mice in a dose-dependent manner, opening promising avenues for the treatment of these infections in humans.

This novel binding site, and its structural characterization, illustrate how bacterial glycosyltransferases, which are key players in disrupting host immune pathways, can be effectively targeted by small molecule inhibitors such as YM155. Furthermore, our findings on SseK2 suggest that the inhibition mechanism of YM155 may extend to multiple members of the Arg-GT family, broadening its potential application. Finally, the novel STD NMR Epitope Perturbation by Mutation approach, validated by experiments on both NleB1 and SseK2, has been shown to be an effective method for localizing ligand binding sites and mapping binding epitopes. The successful application of this novel NMR approach opens new possibilities for the study of ligand–protein interactions, particularly in enzymes where traditional X-ray crystallography or other methods can be challenging.

The ability of YM155 to inhibit bacterial virulence in a mouse model paves the way for promising new treatments for enteropathogenic infections. Further studies must explore the broader applicability of YM155 to other glycosyltransferases and related bacterial pathogens, potentially opening new therapeutic avenues for combating infectious diseases.

## Material and Methods

### Site-Directed Mutagenesis

The mutants (Y283 and Y284) were made by GenScript using the pMALC2x-12Hist-TEV-NleB1^EHEC^ as the template.^3^

### Protein Expression and Purification

NleB1^WT^, the two mutants Y283 and Y284 and SseK2 were expressed and purified as described before.^3^

### Kinetic Analysis

Enzyme kinetics for the NleB1^WT^, and the mutants Y283A and Y284A were determined using the UDP-Glo luminescence assays (Promega). Reactions contained 10 nM of the enzymes in 25 mM Tris pH 7.5, 150 mM NaCl, 50 μM MnCl_2_ and saturating concentrations of UDP-GlcNAc (800 μM) and FADD^DD^ (800 μM). Reactions were incubated 30 min at 37 °C and stopped using 5 μL of UDP-detection reagent at a 1:1 ratio in a White and opaque 384-well plate. Then, the plates were incubated in the dark for 1 h at room temperature. Subsequently, the values were obtained by using a CLARIOstar (BMG LABTECH). To estimate the amount of UDP produced in the glycosyltransferase reaction, we created a UDP standard curve. GraphPad Prism 6 software was used to represent the percentage values of the activity. All experiments were performed in triplicate.

### STD NMR Experiments

1D ^1^H STD NMR experiments were performed on a 600 MHz on a Bruker Avance III spectrometer equipped with a cryoprobe QCI Cryo 5 mm (1H/19F 15N/13C) for 1H, 15N, 13C, and 19F with 2H decoupling. NMR sample was prepared in 500 μL in buffer D2O (150 mM NaCl, 10 mM MgCl_2_, 25 mM d-Tris, pD 7,5), and with 23 μM of the protein for the studies with NleB1^Y283A^ and NleB1^Y284A^, but 50 μM for the study with NleB1^WT^ and SseK2^WT^. The concentration of the ligand was 1 mM for the studies with NleB1^Y283A^ and NleB1^Y284A^, but 2 mM for the study with NleB1^WT^ and SseK2^WT^. All experiments were carried out at 5 °C. The on- and off-resonance spectra were acquired using a train of 50 ms Gaussian selective saturation pulses using a variable saturation time from 0.25 to 5 s, and a relaxation delay (D1) of 5 s. The residual protein resonances were filtered using a T1ρ-filter of 25 ms. All spectra were acquired with a spectral width of 9 kHz and 24K data points using 32 scans in saturation times of 0.25, 0.5, 0.75, and 16 scans in 1, 1.25, 1.75, 2, 2.5, 3, 4, 5 s for the study with NleB1^WT^ and NleB1^Y283A^. 64 scans in saturation times of 0.25, 0.5 s, 32 scans in 0.75, 1, 1.25, and 16 scans in 1.75, 2, 2.5, 3, 4, 5 s for the study with NleB1^Y284A^. 128 scans in saturation times of 0.5, 0.75, 1 s, 64 scans in 1.25, 1.75, and 32 scans in 2, 2.5, 3, 4, 5 s for the study with SseK2^WT^. The on-resonance spectra were acquired by saturating aliphatic hydrogens, specifically at 0.5 ppm for all the experiments, whereas the off-resonance spectra were in all cases acquired by saturating at 40 ppm. To obtain accurate structural information from the STD NMR data and to minimize any T_1_ relaxation bias, the equation STD(*t*_sat_) = STD_max_·(1 – exp(−*k*_sat_·*t*_sat_)) was fitted to the experimental STD build–up curves, calculating the initial growth rate STD_0_ factor as the product of the two resulting fitting parameters, ST*D*_max_·*k*_sat_, and then normalizing all of them to the highest value.^22^ As a slope of the curve of STD vs saturation time, the units of STD_0_ are s^–1^, as any STD factor is a uniless ratio between intensities.

For STD NMR competition experiments between YM155 and UDP with NleB1^WT^ the first NMR sample was in 500 μL in buffer D2O (150 mM NaCl, 10 mM MgCl_2_, 25 mM d-Tris, pD 7,5) with 50 μM of the protein and 2 mM of YM155. Then, an equimolar concentration (2 mM) of UDP was added. For STD NMR competition experiments between YM155 with UDP-GlcNAc and YM155 with UDP-GalNAc using NleB1^WT^ the first NMR sample was in 500 μL in buffer D2O (150 mM NaCl, 10 mM MgCl_2_, 25 mM d-Tris, pD 7,5) with 20 μM of the protein and 1 mM of YM155. Then, an equimolar concentration (1 mM) of UDP-GlcNAc and UDP-GalNAc was added in each case. For STD NMR competition experiments between YM155 and FADD with NleB1^WT^ the first NMR sample was in 500 μL in buffer D2O (150 mM NaCl, 10 mM MgCl_2_, 25 mM d-Tris, pD 7,5) with 50 μM of the protein and the concentration of the YM155 and UDP were 2 mM. Then, an excess of FADD over the enzyme (68 μM) was added.

### Molecular Docking Calculations

Crystal structure of NleB1^WT^ (PDB: 6ACI) was imported into Schrödinger Maestro^19^ and prepared with the Protein Preparation Wizard.^23^ All buffer atoms, nonbridging waters, chain H and UDP were removed. Protons were then added to the model, using PROPKA to predict the protonation state of polar side chains at pH 7.^24^ The hydrogen-bonding network was automatically optimized by sampling asparagine, glutamine, and histidine rotamers. The model was then minimized using OPLS3^25^ force field and a heavy atom convergence threshold of 0.3 Å. Different binding sites were obtained with SiteMap,^26^ using a more restrictive definition of hydrophobicity and standard grid. Conformers of YM155 were generated in MacroModel^27^ using the MC/SD tool and 100 different conformers were obtained. Clustering of the conformers was carried by heavy atom RMSD to eliminate redundant poses, and 10 clusters were generated. From each cluster, the lowest energy conformer was chosen considering the potential energy-OPLS3e term. Docking of the different conformers of YM155 to NleB1^WT^ was then performed using Glide.^28^ A cubic grid was generated centered on the Site-2, with an outer box length of 20 Å and an inner box length of 10 Å. All ligand conformers were subjected to rigid docking (i.e., protein residues are kept fixed in the initial conformation) using the SP algorithm, obtaining 100 different poses for each conformer. Docking poses were then clustered by heavy atom RMSD, and the pose closer to the centroid of each cluster was selected. Finally, selected poses ware assessed against experimental STD NMR data using RedMat and the models with the lowest *R*-NOE factors were chosen.

### Molecular Dynamics (MD) Simulations

#### Input Preparation and Equilibration

The initial coordinates of the NleB1^WT^–YM155 complex were built from the coordinates of the model with the lowest *R*-NOE factor obtained from docking simulations. The initial coordinates of the NleB1^WT^–YM155 of the quaternary complex NleB1^WT^–FADD-UDP-YM155 were built from the coordinates of the model with the lowest *R*-NOE factor obtained from docking simulations, and the initial coordinates of FADD and UDP of the quaternary complex were constructed from the X-ray structure (PDB code: 6ACI) after an alignment with the model mentioned above. The MD simulation setup and equilibration were performed with the BioExcel Building Blocks (BioBB) library.^29^ The ligands were parametrized and minimized using the acpype and babel modules, respectively, of BioBB (biobb_chemistry.acpype and biobb_chemistry.babel). The minimization of the ligands was performed with the steepest descent method and the GAFF force field. The topologies of the complexes were generated with the biobb_amber.leap module, and ff14SB^30^ and GAFF^31^ force fields were used to parametrize protein^30^ and ligand,^31^ respectively. Subsequently, the biobb_amber.sander module was employed to minimize, first, the protein protons using positional restraints of 50 kcal/mol·Å2 on the protein heavy atoms and, second, the whole protein structure using positional restraints of 500 kcal/mol·Å2 on the ligand to avoid potential changes in ligand orientation due to protein repulsion. Then, each protein–ligand complex was immersed in a TIP3P^32^ truncated octahedron water box with a distance from the protein to the box edge of 9.0 Å and periodic boundary conditions, followed by the addition of a 150 mM concentration of NaCl. Each solvated system was minimized using the steepest descent protocol and applying positional restraints of 15 kcal/mol·Å2 to the ligand, followed by heating up to 300 K over 2500 steps applying the Langevin thermostat^33^ with a collision frequency of 1 ps–1 and positional restraints on the ligand of 10 kcal/mol·Å2 (for this, the biobb_amber.sander module was used). Next, each system was subjected to *NVT* followed by *NPT* equilibration of 100 ps each. A nonbonded interactions cutoff of 10.0 Å, the SHAKE algorithm for constraining the length of bonds involving hydrogen atoms, the Langevin thermostat with a collision frequency of 5 ps–1, and smooth positional restraints on the ligand (5 and 2.5 kcal/mol·Å2 for *NVT* and *NPT*, respectively) were employed. During the *NPT* equilibration, a pressure of 1 bar was kept constant using isotropic position scaling with a pressure relaxation time of 2 ps.

#### Molecular Dynamics

A 300 ns of MD production run was carried out for each complex on a AMD-Ryzen 4xGPU 3070 Computing Cluster using the pmemd.cuda module of AMBER 20.^34^ The production dynamics was performed at a constant temperature of 300 K, by applying the Langevin thermostat^33^ with a collision frequency of 1 ps–1, and a constant pressure of 1 bar (using isotropic position scaling with a pressure relaxation time of 1 ps). A nonbonded interactions cutoff of 9.0 Å, periodic boundary conditions (PBC),^35^ and the Particle Mesh Ewald method^36^ (PME) to account for the long-range electrostatic effect were employed. The SHAKE algorithm^37,38^ was also employed, thus allowing 2 fs between time steps. Trajectory coordinates were saved every nanosecond. The analysis of the MD trajectories was performed using the CPPTRAJ module (version 4.25.6) of AMBER 20.^34^ The evolution of protein and ligand RMSD over the simulation time was calculated against the first frame of the trajectory. To monitor ligand orientation and dynamics within the protein binding site, MD trajectories were aligned based on the protein backbone atoms within 5 Å of the ligand (in the first frame) and, subsequently, the ligand backbone RMSD was calculated in-place (no superposition).

### Reduced Matrix (RedMat) STD NMR Binding Epitope Calculations

For the RedMat calculation, we selected irradiated atoms in methyl protons, we used a dissociation constant of 500 μM and a cutoff distance of 18 Å. The ligand and protein concentrations were 2 mM and 50 μM, respectively, according to the experimental conditions.

### Hydrogen–Deuterium Exchange (HDX) Mass Spectrometry

NleB1 and SseK2 were incubated at a concentration of 20 μM with YM155 at 4.8 mM (protein: ligand ratio 1:240) or with an equivalent volume of protein buffer (25 mM Tris, 100 mM NaCl for NleB1 and 25 mM Tris, 300 mM NaCl for SseK2). The HDX reaction was initiated by 8-fold dilution in deuterated buffers, which had the same composition as the protein buffers but were in 100% D_2_O (pH_read_ 7.25). The reaction was carried out at 23 °C for 10 s, 100 s, 1000 s and 10,000 s; and at 28 °C for 12 h. After these selected time intervals, an 8 μL-aliquot was withdrawn from the labeling mixture and diluted with 52 μL of an in ice-cold quenching solution containing 23 μL of deuterated buffer and 29 μL of 4 M Urea in acidic phosphate buffer, which reduced the pH/D_read_ of the sample to 2.3 and the D_2_O content to 50%. The quenched samples where immediately snap-frozen in liquid nitrogen and kept frozen at −80 °C for 2–4 days before LC–MS analysis. Triplicates were performed at every time point, except for 12 h at 28 °C, which was performed in duplicates (Table S6). Frozen protein samples were quickly thawed and injected into an Acquity UPLC M-Class System with HDX Technology (Waters). Proteins were online digested at 20 °C into a homemade Pepsin column and trapped/desalted with solvent A (0.23% formic acid in water, pH 2.5) for 3 min at 200 μL/min and at 0 °C through an Acquity BEH C18 VanGuard precolumn (1.7 μm, 2.1 mm × 5 mm, Waters). Peptides were eluted into an Acquity UPLC BEH C18 analytical column (1.7 μm, 2.1 mm × 100 mm, Waters) with a 7 min-linear gradient raising from 8 to 35% of solvent B (0.23% formic acid in acetonitrile) at a flow rate of 40 μL/min and at 0 °C. Then, peptides went through electrospray ionization in positive mode and underwent MS analysis with ion mobility separation with a Synapt G2-Si mass spectrometer (Waters). Peptides were identified by digesting the nondeuterated NleB1 and SseK2 using the same protocol and identical LC gradient as detailed above and performing MS^E^ analysis with collision energy ramping from 20 to 30 k . Leucine enkephalin was applied for mass accuracy correction. MS^E^ runs were analyzed with ProteinLynx Global Server (PLGS) 3.0 (Waters) and peptides identified in 3 out of 4 runs, with at least 0.2 fragments per amino acid and 2 fragments in total, with minimum intensity 1481, minimum PLGS score 6.62 and mass error below 7.5 ppm were selected in DynamX 3.0 (Waters) to be successively searched as deuterated peptides in the HDX-MS runs. Peptide-level deuterium uptake was calculated with DynamX 3.0 and data visually inspected and curated (Tables S7 and S8). The threshold for the statistically significant difference in HDX (ΔHDX) was established at the significance level of 98%, based on an approach described earlier (Table S6).^39^ Peptides showing a significant difference in HDX in at least one time point were considered positive hits, and the magnitude of their HDX effect was calculated as sum of the ΔHDX of every time point, normalized by the number of exchangeable amides at 0.875 deuterium fraction (no back-exchange correction was applied).

### Mouse Studies

Mouse experiments were performed according to Institutional Animal Care and Use guidelines (Animal Welfare Assurance #4543) and under Institutional Biosafety Committee-approved protocols (approval #1540). Five-week-old C57BL/6 mice (Jackson Laboratory) were housed at Kansas State University. *C. rodentium* DBS100 was cultivated in LB broth with shaking at 200 rpm at 37 °C overnight. Mice were infected via oral gavage with 10^9^ CFUs of *C. rodentium* in 100 μL PBS. YM155 was administered to one group of mice via intraperitoneal (IP) injection immediately before oral gavage of *C. rodentium*. YM155 was provided to another group of mice via IP injection at 24 h after oral gavage of *C. rodentium*. Mice were euthanized 7 days after infection, colons were homogenized, serially diluted, and plated on MacConkey agar, with enumeration of viable bacterial counts taking place the following day.

## Supporting Information Available

In the Supporting Information, we provide STD build–up curves (Figure S1), activity of mutants Y283A and Y284A (Figure S2), STD NMR competition experiment (Tables S2–S4), binding sites obtained from Maestro Schrödinger (Figure S3), NOE *R*-factors calculated for the 3D models obtained from docking simulations (Table S5), HDX sequence coverages (Figures S4 and S6), HDX differential plots (Figures S5 and S7), HDX effects superimposed to SseK2 structure (Figure S8), HDX summary table (Table S6), deuterium uptake value tables (Tables S7 and S8), deuterium, uptake plots (Figures S11 and S12) (PDF)
Movie MD ternary complexes with & without YM155 (MP4)
