# Supplementary material for: STD NMR Epitope Perturbation by Mutation Unveils the Mechanism of YM155 as an Arginine-Glycosyltransferases Inhibitor Effective in Treating Enteropathogenic Diseases
Source: JACS Au. 2025 Mar 5;5(3):1279–88. doi: 10.1021/jacsau.4c01140 (PMC11937963; doi:10.1021/jacsau.4c01140)
Supplement: Supplementary file 1 — au4c01140_si_001.pdf [file au4c01140_si_001.pdf]

SUPPORTING INFORMATION

STD NMR Epitope Perturbation by Mutation Unveils the Mechanism of YM155 as an Arginine-Glycosyltransferases Inhibitor Effective in Treating Enteropathogenic Diseases

Jonathan Ramírez-Cárdenas[a], Víctor Taleb[b], Valeria Calvaresi[c,d], Weston B. Struwe[c,d], Samir El Qaidi[e], Congrui Zhu[e], Kamrul Hasan[e], Yinxin Zhang[e], Philip R. Hardwidge[e], Billy Veloz [b], Juan Carlos Muñoz-García[a], Ramón Hurtado-Guerrero*[b,f,g], Jesús Angulo*[a]

[[a] Instituto de Investigaciones Químicas (CSIC – Universidad de Sevilla)
49 Américo Vespucio St., Sevilla, 41092, Spain
E-mail: j.angulo@iiq.csic.es

[b] Institute of Biocomputation and Physics of Complex Systems, University of Zaragoza
Mariano Esquillor s/n, Campus Rio Ebro, Edificio I+D, Zaragoza, 50018, Spain
E-mail: rhurtado@bifi.es

[c] Department of Biochemistry, University of Oxford,
Oxford, OX1 3QU, United Kingdom

[d] The Kavli Institute for Nanoscience Discovery, University of Oxford, Dorothy Crowfoot Hodgkin Building, South Parks Road, Oxford OX1 3QU, United Kingdom

[e] College of Veterinary Medicine, Kansas State University, Manhattan, KS, 66506, USA.

[f] Copenhagen Center for Glycomics, Department of Cellular and Molecular Medicine University of Copenhagen, Copenhagen, Blegdamsvej 3B, 2200, Denmark

[g] Fundación ARAID, Zaragoza, 50018, Spain

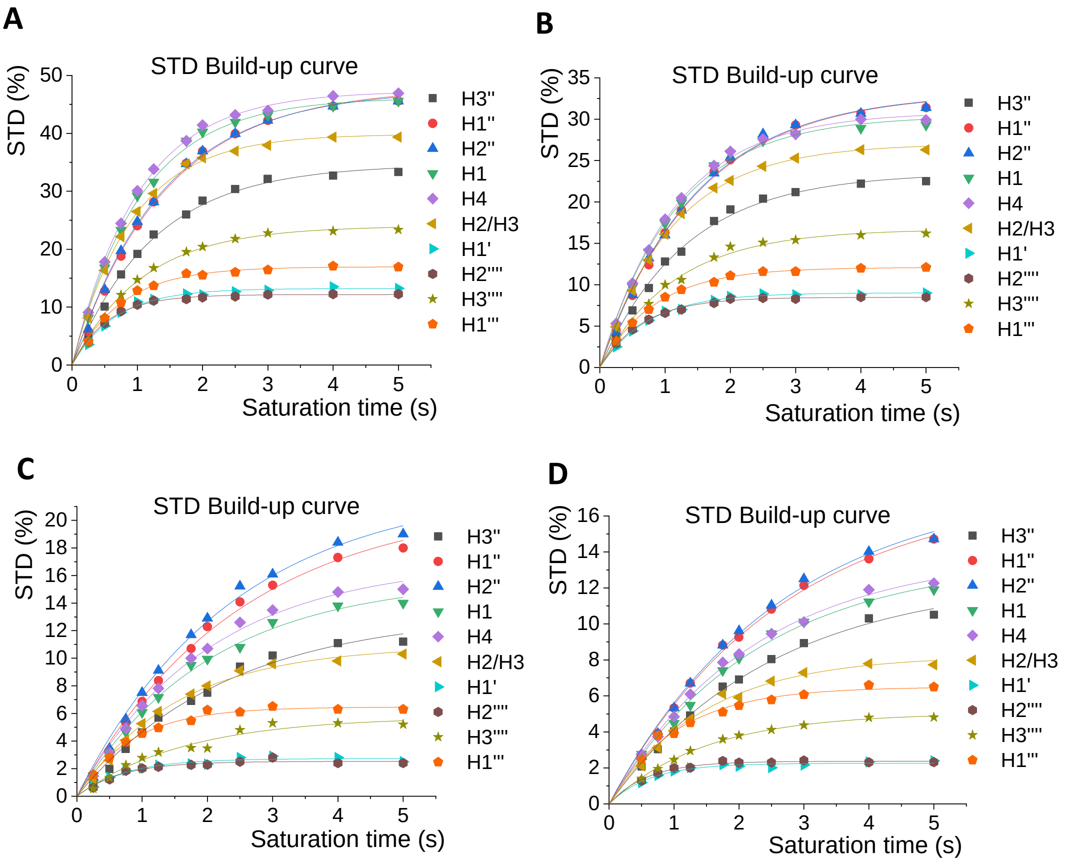

Figure S1. STD NMR study of the binding of NleB1^{WT}, NleB1^{Y283A} and NleB1^{Y284A} and SseK2^{WT} with YM155 with protein irradiation in the aliphatic spectral region. STD NMR build-up curves for NleB1^{WT} (**A**), NleB1^{Y283A} (**B**), NleB1^{Y284A} (**C**) and SseK2^{WT} (**D**) in complex with YM155. Temperature 5°C. Saturation frequency set at 0.5 ppm.

Table S1. STD intensities obtained by normalising of the STD_0 values from build-up curves of YM155 interacting with $NleB1^{WT}$, $NleB1^{Y283A}$ and $NleB1^{Y284A}$ and $SseK2^{WT}$. STD-YM155-$NleB1^{WT}$ represents the values of STD intensities obtained with $NleB1^{WT}$ and YM155 in the sample. STD-YM155-$NleB1^{Y283A}$ represents the values of STD intensities obtained with $NleB1^{Y283A}$ and YM155 in the sample. STD-YM155-$NleB1^{Y284A}$ represents the values of STD intensities obtained with $NleB1^{Y284A}$ and YM155 in the sample. STD-YM155-$SseK2^{WT}$ represents the values of STD intensities obtained with $SseK2^{WT}$ and YM155 in the sample.

Protons	STD-YM155-$NleB1^{WT}$	STD-YM155-$NleB1^{Y283A}$	STD-YM155-$NleB1^{Y284A}$	STD-YM155-$SseK2^{WT}$
**H1**	95	98	83	89
**H2-H3**	93	91	76	83
**H4**	100	100	90	93
**H1'**	43	45	40	53
**H1''**	72	85	92	98
**H2''**	74	86	100	100
**H3''**	60	67	62	75
**H2''''**	48	48	40	59
**H3''''**	48	54	37	51
**H1'''**	48	53	83	93

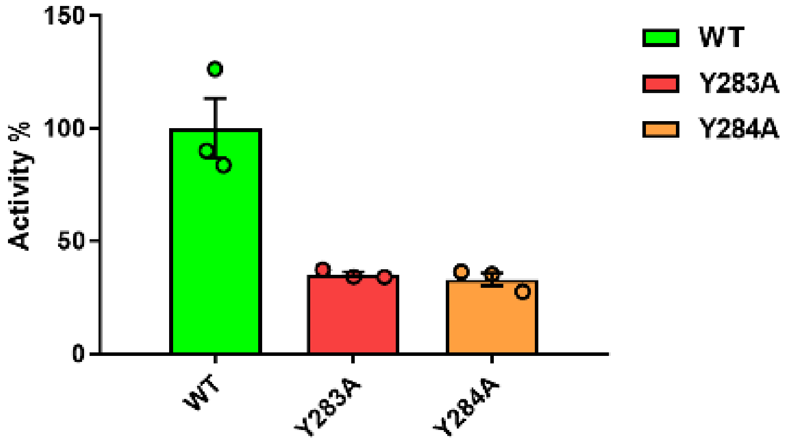

Figure S2. Activity of mutants Y283A and Y284A. Histogram showing the relative activities of the mutants compared to the wild-type (WT) protein. All experiments were done in triplicate (n = 3 independent experiments). Error bars represent the standard deviation calculated by the GraphPad Prism fit of the data sets.

Table S2. STD NMR competition experiment between YM155 and UDP-GlcNAc with NleB1^{WT}. The experiments were carried out at a single saturation time (2 s). Temperature 5 °C. Protein concentration was 20 μM and ligand concentrations were 1 mM. Saturation frequency set at 0.5 ppm.

Proton	STD (%): YM155	STD (%): YM155 + UDP-GlcNAc
H3''	13	10
H1''	20	12
H2''	20	13
H1	18	14
H4	18	14
H2-H3	15	12
H1'	5	4
H3''''	8	7
H1'''	8	6

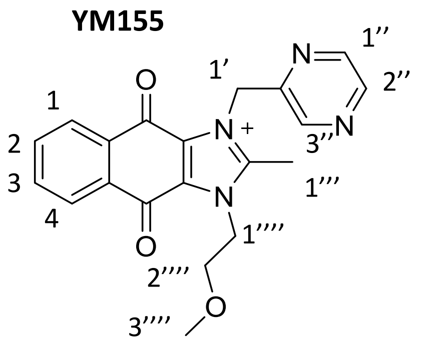

Proton	STD (%): UDP-GlcNAc	STD (%): UDP-GlcNAc + YM155
H1	6	6
H11	4	4
H12	3	3
H13	4	5

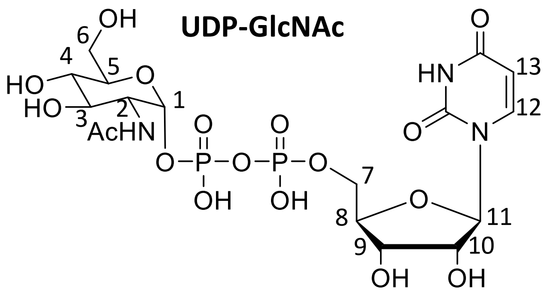

Table S3. STD NMR competition experiment between YM155 and UDP with NleB1^{WT}. The experiments were carried out at a single saturation time (2 s). Temperature 5 °C. Protein concentration was 50 μM and ligand concentrations were 2 mM. Saturation frequency set at 0.5 ppm.

Proton	STD (%): YM155	STD (%): YM155 + UDP
**H3''**	48	46
**H1''**	59	57
**H2''**	59	57
**H1**	63	62
**H4**	63	62
**H2-H3**	59	56
**H1'**	27	26
**H2''''**	26	23
**H3''''**	41,	38
**H1'''**	31	28

YM155

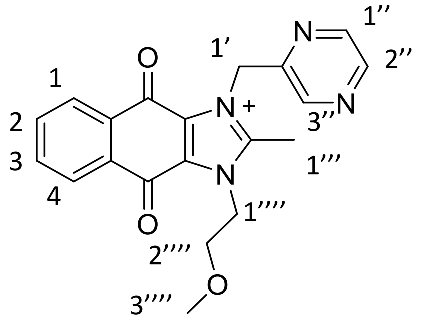

Proton	STD (%): UDP	STD (%): UDP + YM155
**H1**	1	1
**H2**	2	2
**H3**	1	1
**H4**	1	1
**H5a-H5b**	1	1

UDP

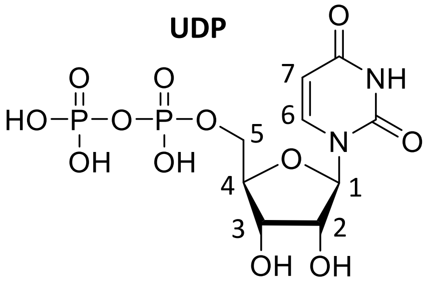

Table S4. STD NMR competition experiment between YM155 and UDP-GalNAc with NleB1^{WT}. The experiments were carried out at a single saturation time (2 s). Temperature 5 °C. Protein concentration was 20 μM and ligand concentrations were 1 mM. Saturation frequency set at 0.5 ppm.

Proton	STD (%): YM155	STD (%): YM155 + UDP-GalNAc
**H3''**	11	9
**H1''**	14	11
**H2''**	16	12
**H1**	15	13
**H4**	16	13
**H2-H3**	12	11
**H1'**	4	4
**H3''''**	6	6
**H1'''**	7	6

YM155

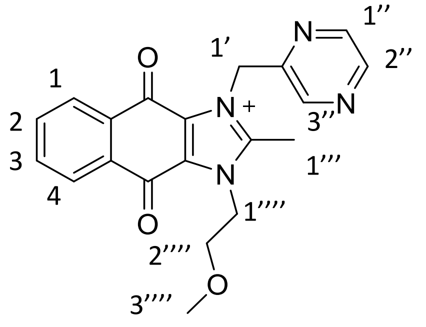

Protons	STD (%): UDP-GalNAc	STD (%): UDP-GalNAc + YM155
**H1**	5	6
**H11**	3	3
**H12**	4	4
**H13**	4	5

UDP-GalNAc

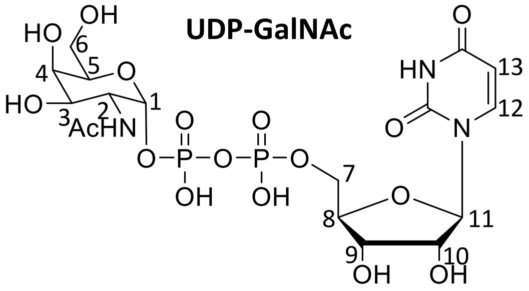

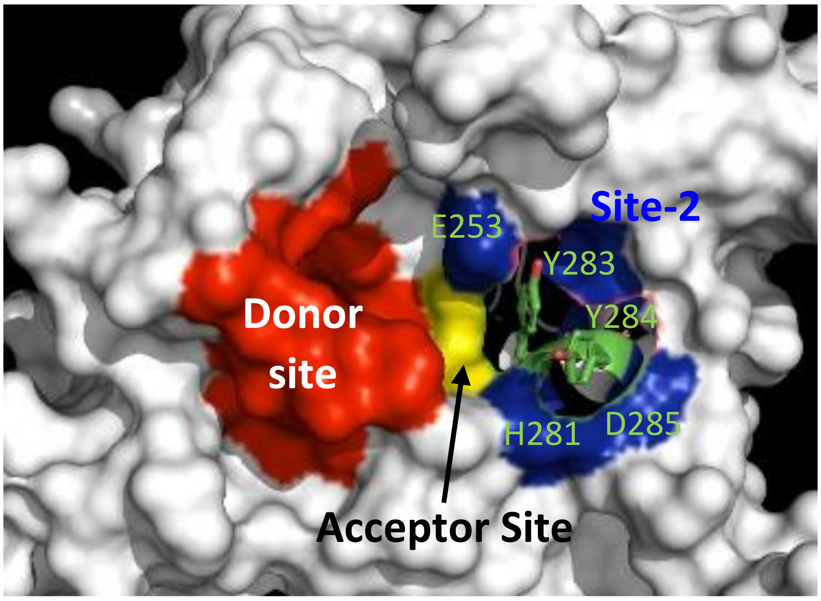

Figure S3. Binding sites obtained through *sitemap* tool from Maestro by Schrodinger. In yellow: acceptor site. In red: donor site. In blue: *Site-2* which is the new binding site consisting of residues D186, L252, E253, N254, H281, P282, Y283, Y284, D285, L287 and G288, some of them are shown in the picture for easy identification. In green: key tyrosine residues Y283 and Y284.

Table S5. NOE R-factors calculated for the 3D models obtained from docking simulations. Dockings simulations were carried out on *Site-2.* Dissociation constant set at 500 μM. Correlation time set at 50 ns. Cutoff distance set at 18 Å.

	**R-NOE**
**Model 1**	0,48
**Model 2**	0,44
**Model 3**	0,46
**Model 4**	0,58
**Model 5**	0,60
**Model 6**	0,48
**Model 7**	0,55
**Model 8**	0,51
**Model 9**	0,45
**Model 10**	0,30
**Model 11**	0,29
**Model 12**	0,29
**Model 13**	0,49
**Model 14**	0,50
**Model 15**	0,33
**Model 16**	0,32
**Model 17**	0,46
**Model 18**	0,51
**Model 19**	0,38
**Model 20**	0,35

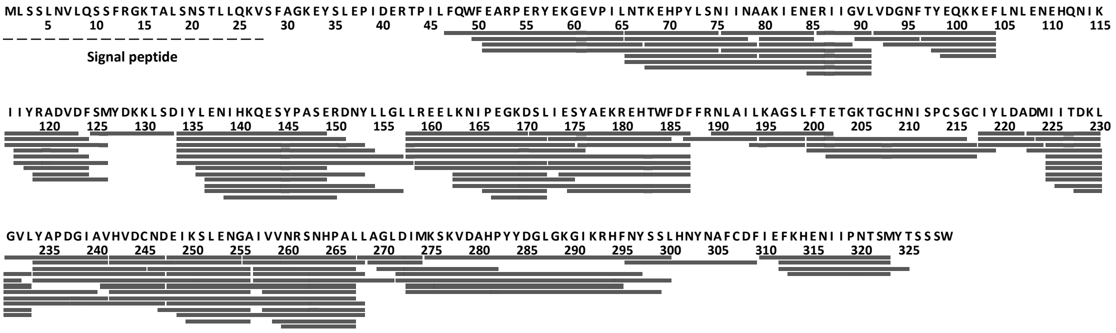

125 Peptides, 89.1% Coverage, 6.29 Redundancy

Figure S4. Effective HDX sequence coverage of NleB1. Peptides whose HDX was followed are depicted along the protein sequence of NleB1.

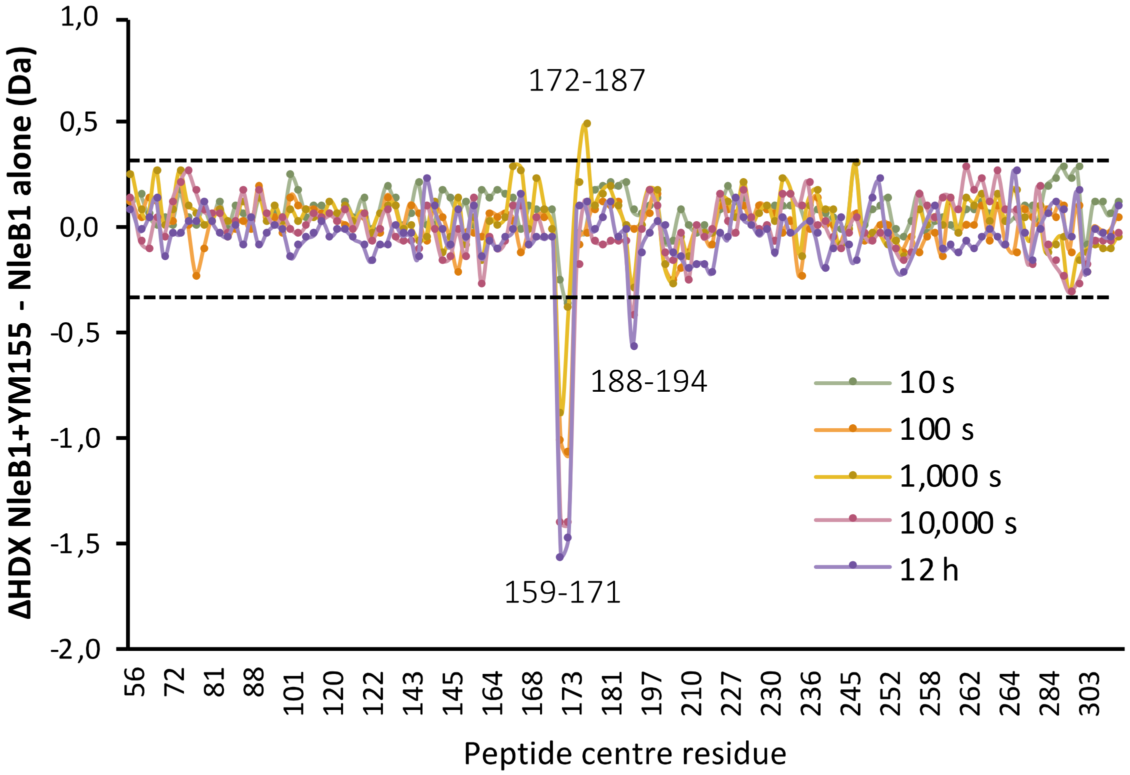

Figure S5. Difference plot illustrating peptide-level differences in HDX between apo- and YM155-bound NleB1 over the different time points studied. The 125 peptides followed are arranged on the x-axis based on their central residue according to their position from the N- to the C-terminus. On the y-axis, their ΔHDX (bound – apo) in expressed in Da. The dotted black lines represent the threshold of significance calculated at ΔHDX>0.30 Da.

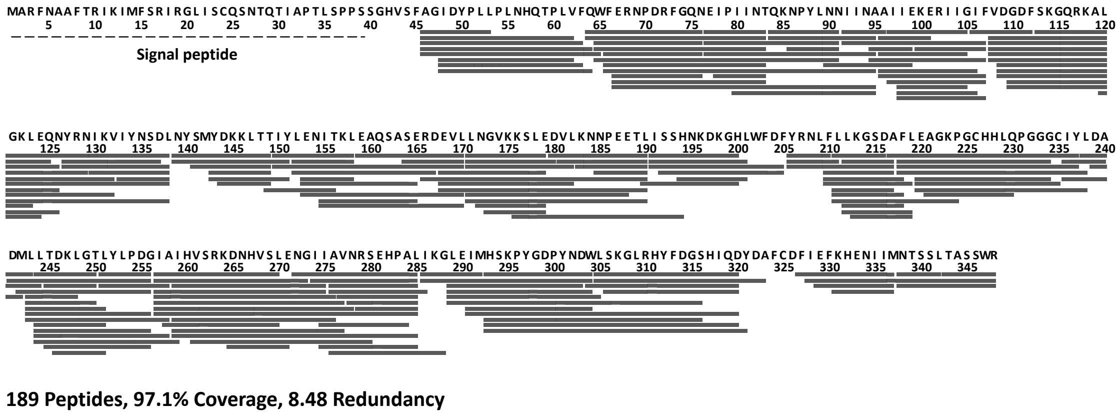

Figure S6. Effective HDX sequence coverage of SseK2. Peptides whose HDX was followed are depicted along the protein sequence of SseK2.

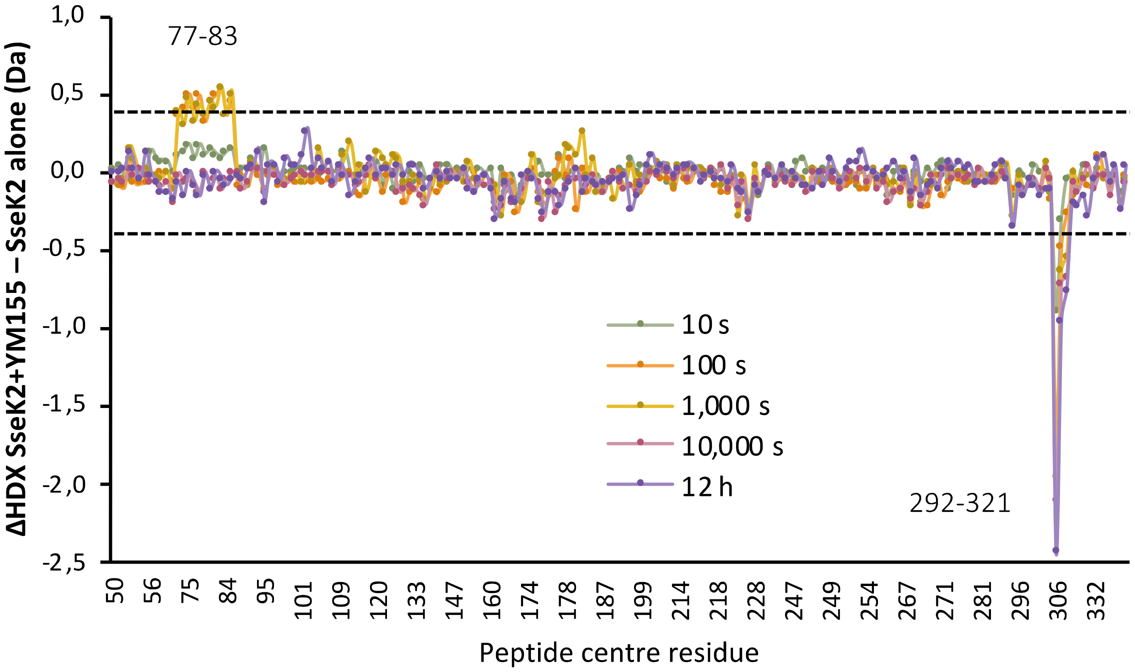

Figure S7. Difference plot illustrating peptide-level differences in HDX between apo- and YM155-bound SseK2 over the different time points studied. The 189 peptides followed are arranged on the x-axis based on their central residue according to their position from the N- to the C-terminus. On the y-axis, their ΔHDX (bound – apo) in expressed in Da. The dotted black lines represent the threshold of significance calculated at ΔHDX>0.38 Da.

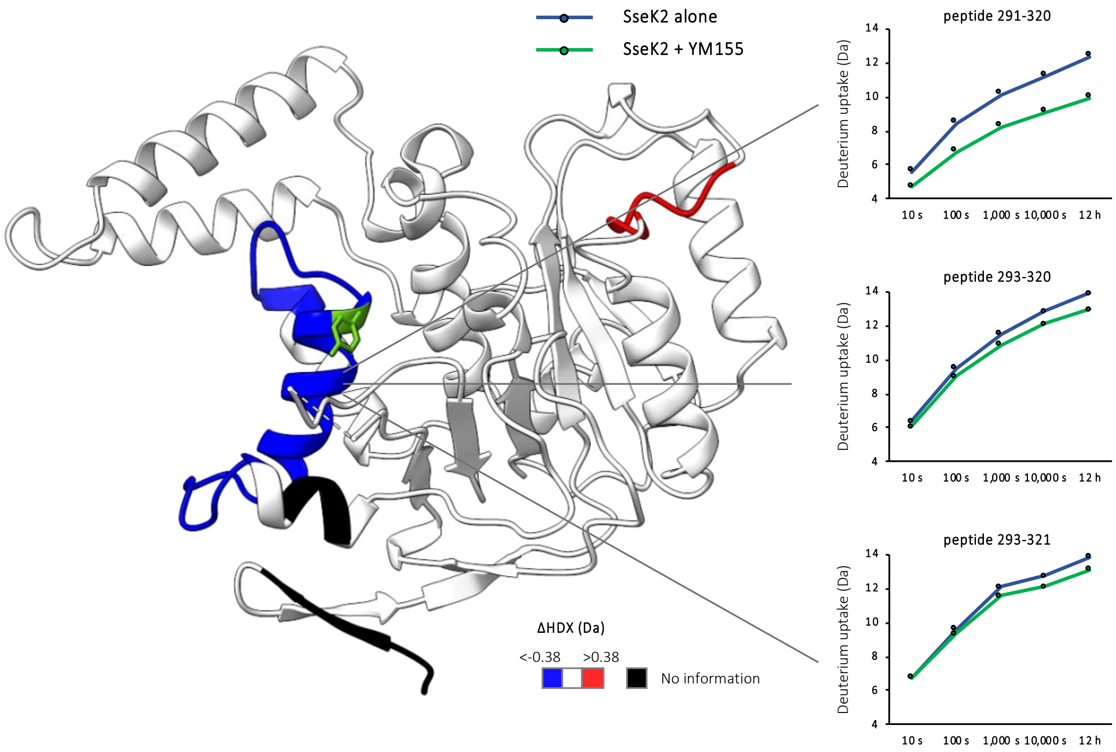

Figure S8. Differences in HDX between apo- and YM155-bound SseK2 are superimposed into the structure PDB: 5H62. On the left, region 292-321 is coloured in blue as showing significant decrease in HDX in the bound state, whereas the loop 77-83 is coloured in red as it manifests a significant increase in HDX in the bound state. Y301 is coloured in green. On the right, the deuterium uptake plot of peptides spanning region 292-321 showing significant differences in HDX are shown.

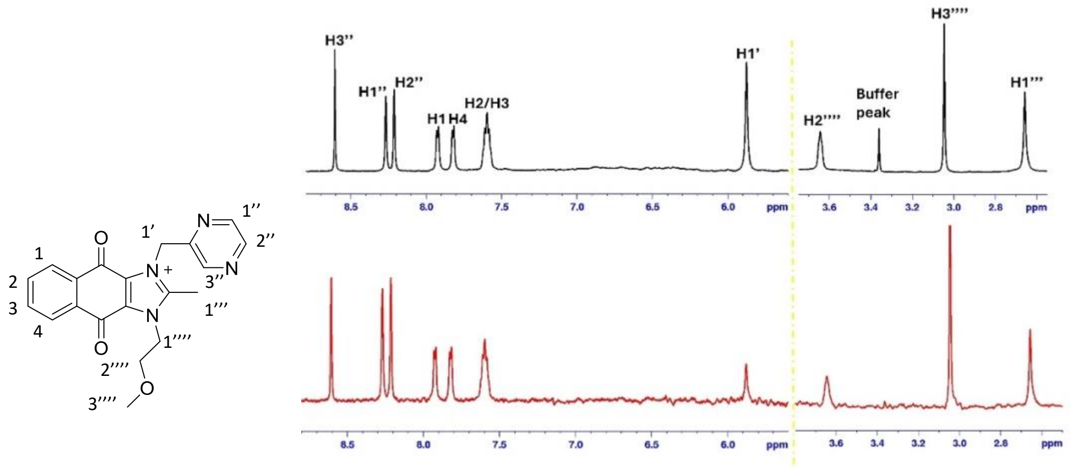

Figure S9. ^{1}H-NMR and STD spectra of YM155 in the presence of NleB1^{WT}. The black spectrum represents the ^{1}H-NMR spectrum of YM155 in the presence of NleB1^{WT}, with the corresponding assignment of the different ligand protons. The red spectrum corresponds to the STD spectrum of YM155 in the presence of NleB1^{WT}, with proton assignments omitted for clarity. The saturation time for the STD NMR experiment was 2s. The protein and ligand concentrations were 50 and 2000 µM, respectively.

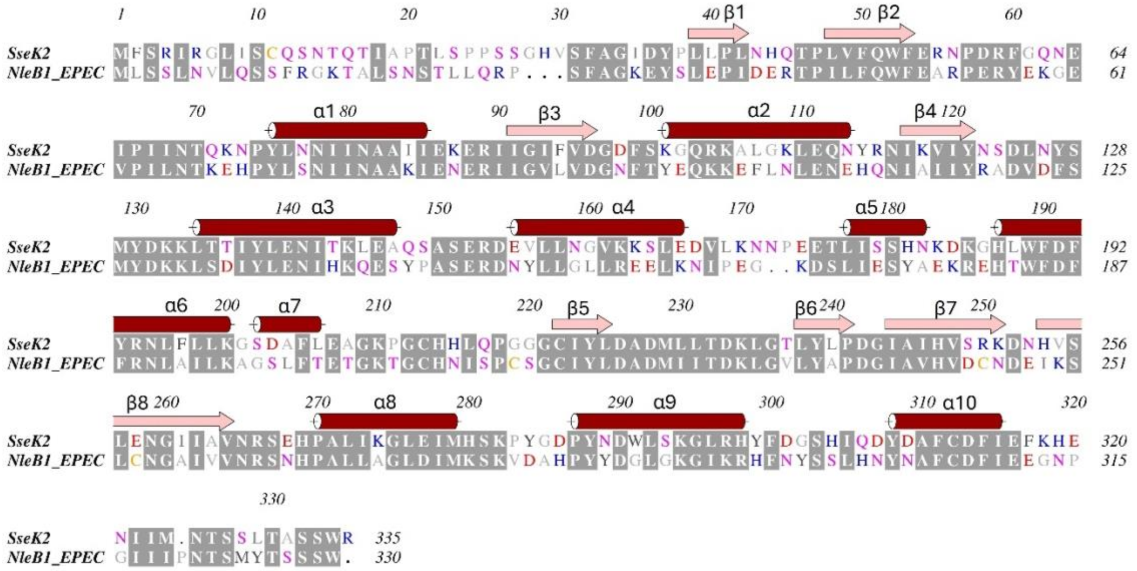

Figure S10. Sequence alignment of NleB1^{WT} and SseK2^{WT}. All residues highlighted with a gray background are either perfectly conserved or highly similar based on their physicochemical properties. Residues with a white background exhibit low similarity. Different colors indicate groups of residues with similar physicochemical characteristics.

Dataset	NleB1 alone	NleB1 + YM155
HDX reaction details	25 mM Tris, 100 mM NaCl in D_2O, pHread 7.25, %D_2O 87.5%	
HDX time courses	10 s, 100 s, 1,000 s and 10,000 s at 23 °C; 12 h at 28 °C	
HDX control sample	no MaxD control	
Back-exchange (Mean/IQR)	ND	
# peptides/ average peptide length	125 / 13.50	
Sequence coverage	89.1%	
Replicates (technical)	duplicate for 12 h; triplicates for all other time points	
Repeatability (average STD)	0.03487426	0.047353416
Significant difference in HDX	ΔHDX> 0.30 (98% CI)	

Dataset	SseK2 alone	SseK2 + YM155
HDX reaction details	25 mM Tris, 300 mM NaCl in D_2O, pHread 7.25, %D_2O 87.5%	
HDX time courses	10 s, 100 s, 1,000 s and 10,000 s at 23 °C; 12 h at 28 °C	
HDX control sample	no MaxD control	
Back-exchange (Mean/IQR)	ND	
# peptides/ average peptide length	189 / 13.46	
Sequence coverage	97.1%	
Replicates (technical)	duplicate for 12 h; triplicates for all other time points	
Repeatability (average STD)	0.059614468	0.043960507
Significant difference in HDX	ΔHDX> 0.38 (98% CI)	

Table S6. Summary of the method of HDX-MS experiments.

Protein	Start	End	Sequence	State	Exposure	Uptake (Da)	Uptake SD	RT (min)
NleB1	47	65	FQWFEARPERYEKGEVPIL	NleB1 +YM155	10 s (23 C)	3.40	0.059473	7.43
NleB1	47	65	FQWFEARPERYEKGEVPIL	NleB1 +YM155	100 s (23 C)	6.13	0.100287	7.40
NleB1	47	65	FQWFEARPERYEKGEVPIL	NleB1 +YM155	1,000 s (23 C)	8.24	0.07149	7.41
NleB1	47	65	FQWFEARPERYEKGEVPIL	NleB1 +YM155	10,000 s (23 C)	9.43	0.069225	7.41
NleB1	47	65	FQWFEARPERYEKGEVPIL	NleB1 +YM155	12 h (28 C)	10.15	0.095498	7.41
NleB1	47	65	FQWFEARPERYEKGEVPIL	NleB1 alone	10 s (23 C)	3.28	0.03154	7.44
NleB1	47	65	FQWFEARPERYEKGEVPIL	NleB1 alone	100 s (23 C)	6.01	0.070163	7.41
NleB1	47	65	FQWFEARPERYEKGEVPIL	NleB1 alone	1,000 s (23 C)	7.98	0.011984	7.42
NleB1	47	65	FQWFEARPERYEKGEVPIL	NleB1 alone	10,000 s (23 C)	9.28	0.028187	7.42
NleB1	47	65	FQWFEARPERYEKGEVPIL	NleB1 alone	12 h (28 C)	10.06	0.125082	7.40
NleB1	50	65	FEARPERYEKGEVPIL	NleB1 +YM155	10 s (23 C)	3.47	0.086051	5.80
NleB1	50	65	FEARPERYEKGEVPIL	NleB1 +YM155	100 s (23 C)	5.80	0.027313	5.75
NleB1	50	65	FEARPERYEKGEVPIL	NleB1 +YM155	1,000 s (23 C)	7.18	0.053374	5.77
NleB1	50	65	FEARPERYEKGEVPIL	NleB1 +YM155	10,000 s (23 C)	7.92	0.060386	5.77
NleB1	50	65	FEARPERYEKGEVPIL	NleB1 +YM155	12 h (28 C)	8.14	0.008044	5.76
NleB1	50	65	FEARPERYEKGEVPIL	NleB1 alone	10 s (23 C)	3.30	0.051388	5.84
NleB1	50	65	FEARPERYEKGEVPIL	NleB1 alone	100 s (23 C)	5.76	0.056934	5.81
NleB1	50	65	FEARPERYEKGEVPIL	NleB1 alone	1,000 s (23 C)	7.09	0.049664	5.82
NleB1	50	65	FEARPERYEKGEVPIL	NleB1 alone	10,000 s (23 C)	7.98	0.042884	5.82
NleB1	50	65	FEARPERYEKGEVPIL	NleB1 alone	12 h (28 C)	8.15	0.081355	5.80
NleB1	51	67	EARPERYEKGEVPILNT	NleB1 +YM155	10 s (23 C)	2.77	0.090078	5.21
NleB1	51	67	EARPERYEKGEVPILNT	NleB1 +YM155	100 s (23 C)	5.73	0.056324	5.17
NleB1	51	67	EARPERYEKGEVPILNT	NleB1 +YM155	1,000 s (23 C)	7.87	0.026718	5.18
NleB1	51	67	EARPERYEKGEVPILNT	NleB1 +YM155	10,000 s (23 C)	8.61	0.056421	5.18
NleB1	51	67	EARPERYEKGEVPILNT	NleB1 +YM155	12 h (28 C)	8.89	0.005042	5.17
NleB1	51	67	EARPERYEKGEVPILNT	NleB1 alone	10 s (23 C)	2.70	0.029235	5.24
NleB1	51	67	EARPERYEKGEVPILNT	NleB1 alone	100 s (23 C)	5.59	0.018174	5.21

NleB1	51	67	EARPERYEKGEVPILNT	NleB1 alone	1,000 s (23 C)	7.82	0.054178	5.21
NleB1	51	67	EARPERYEKGEVPILNT	NleB1 alone	10,000 s (23 C)	8.70	0.027443	5.21
NleB1	51	67	EARPERYEKGEVPILNT	NleB1 alone	12 h (28 C)	8.84	0.05001	5.19
NleB1	51	75	EARPERYEKGEVPILNTKEHPYLSN	NleB1 +YM155	10 s (23 C)	3.90	0.109657	4.99
NleB1	51	75	EARPERYEKGEVPILNTKEHPYLSN	NleB1 +YM155	100 s (23 C)	7.08	0.087413	4.96
NleB1	51	75	EARPERYEKGEVPILNTKEHPYLSN	NleB1 +YM155	1,000 s (23 C)	9.87	0.037664	4.97
NleB1	51	75	EARPERYEKGEVPILNTKEHPYLSN	NleB1 +YM155	10,000 s (23 C)	12.26	0.14945	4.97
NleB1	51	75	EARPERYEKGEVPILNTKEHPYLSN	NleB1 +YM155	12 h (28 C)	13.25	0.033371	4.96
NleB1	51	75	EARPERYEKGEVPILNTKEHPYLSN	NleB1 alone	10 s (23 C)	3.87	0.037	5.00
NleB1	51	75	EARPERYEKGEVPILNTKEHPYLSN	NleB1 alone	100 s (23 C)	6.95	0.050711	4.98
NleB1	51	75	EARPERYEKGEVPILNTKEHPYLSN	NleB1 alone	1,000 s (23 C)	9.59	0.085584	4.99
NleB1	51	75	EARPERYEKGEVPILNTKEHPYLSN	NleB1 alone	10,000 s (23 C)	12.21	0.026954	4.99
NleB1	51	75	EARPERYEKGEVPILNTKEHPYLSN	NleB1 alone	12 h (28 C)	13.11	0.194449	4.97
NleB1	66	75	NTKEHPYLSN	NleB1 +YM155	10 s (23 C)	0.86	0.01065	3.68
NleB1	66	75	NTKEHPYLSN	NleB1 +YM155	100 s (23 C)	0.94	0.044732	3.66
NleB1	66	75	NTKEHPYLSN	NleB1 +YM155	1,000 s (23 C)	1.33	0.033013	3.67
NleB1	66	75	NTKEHPYLSN	NleB1 +YM155	10,000 s (23 C)	2.20	0.05404	3.66
NleB1	66	75	NTKEHPYLSN	NleB1 +YM155	12 h (28 C)	2.58	0.046154	3.65
NleB1	66	75	NTKEHPYLSN	NleB1 alone	10 s (23 C)	0.80	0.067289	3.69
NleB1	66	75	NTKEHPYLSN	NleB1 alone	100 s (23 C)	0.92	0.047635	3.66
NleB1	66	75	NTKEHPYLSN	NleB1 alone	1,000 s (23 C)	1.32	0.02664	3.64
NleB1	66	75	NTKEHPYLSN	NleB1 alone	10,000 s (23 C)	2.23	0.030507	3.67
NleB1	66	75	NTKEHPYLSN	NleB1 alone	12 h (28 C)	2.71	0.014446	3.62
NleB1	66	78	NTKEHPYLSNIIN	NleB1 +YM155	10 s (23 C)	1.23	0.033762	5.76
NleB1	66	78	NTKEHPYLSNIIN	NleB1 +YM155	100 s (23 C)	1.44	0.039634	5.73
NleB1	66	78	NTKEHPYLSNIIN	NleB1 +YM155	1,000 s (23 C)	2.01	0.091901	5.73
NleB1	66	78	NTKEHPYLSNIIN	NleB1 +YM155	10,000 s (23 C)	3.58	0.066261	5.73
NleB1	66	78	NTKEHPYLSNIIN	NleB1 +YM155	12 h (28 C)	4.86	0.015765	5.72

NleB1	66	78	NTKEHPYLSNIIN	NleB1 alone	10 s (23 C)	1.21	0.022704	5.81
NleB1	66	78	NTKEHPYLSNIIN	NleB1 alone	100 s (23 C)	1.41	0.05017	5.78
NleB1	66	78	NTKEHPYLSNIIN	NleB1 alone	1,000 s (23 C)	1.93	0.025702	5.79
NleB1	66	78	NTKEHPYLSNIIN	NleB1 alone	10,000 s (23 C)	3.46	0.019184	5.79
NleB1	66	78	NTKEHPYLSNIIN	NleB1 alone	12 h (28 C)	4.88	0.060803	5.77
NleB1	66	79	NTKEHPYLSNIINA	NleB1 +YM155	10 s (23 C)	1.04	0.037481	6.08
NleB1	66	79	NTKEHPYLSNIINA	NleB1 +YM155	100 s (23 C)	1.18	0.031885	6.04
NleB1	66	79	NTKEHPYLSNIINA	NleB1 +YM155	1,000 s (23 C)	1.62	0.032396	6.05
NleB1	66	79	NTKEHPYLSNIINA	NleB1 +YM155	10,000 s (23 C)	2.82	0.077643	6.05
NleB1	66	79	NTKEHPYLSNIINA	NleB1 +YM155	12 h (28 C)	3.84	0.043867	6.05
NleB1	66	79	NTKEHPYLSNIINA	NleB1 alone	10 s (23 C)	0.87	0.002844	6.12
NleB1	66	79	NTKEHPYLSNIINA	NleB1 alone	100 s (23 C)	0.97	0.05337	6.10
NleB1	66	79	NTKEHPYLSNIINA	NleB1 alone	1,000 s (23 C)	1.35	0.014142	6.10
NleB1	66	79	NTKEHPYLSNIINA	NleB1 alone	10,000 s (23 C)	2.60	0.015847	6.11
NleB1	66	79	NTKEHPYLSNIINA	NleB1 alone	12 h (28 C)	3.86	0.039846	6.08
NleB1	66	91	NTKEHPYLSNIINAAKIENERIIGVL	NleB1 +YM155	10 s (23 C)	1.58	0.056316	7.86
NleB1	66	91	NTKEHPYLSNIINAAKIENERIIGVL	NleB1 +YM155	100 s (23 C)	1.93	0.015072	7.83
NleB1	66	91	NTKEHPYLSNIINAAKIENERIIGVL	NleB1 +YM155	1,000 s (23 C)	3.00	0.018567	7.83
NleB1	66	91	NTKEHPYLSNIINAAKIENERIIGVL	NleB1 +YM155	10,000 s (23 C)	4.72	0.025096	7.84
NleB1	66	91	NTKEHPYLSNIINAAKIENERIIGVL	NleB1 +YM155	12 h (28 C)	6.32	0.189173	7.82
NleB1	66	91	NTKEHPYLSNIINAAKIENERIIGVL	NleB1 alone	10 s (23 C)	1.56	0.075291	7.87
NleB1	66	91	NTKEHPYLSNIINAAKIENERIIGVL	NleB1 alone	100 s (23 C)	2.16	0.064056	7.83
NleB1	66	91	NTKEHPYLSNIINAAKIENERIIGVL	NleB1 alone	1,000 s (23 C)	2.92	0.057694	7.83
NleB1	66	91	NTKEHPYLSNIINAAKIENERIIGVL	NleB1 alone	10,000 s (23 C)	4.55	0.052854	7.84
NleB1	66	91	NTKEHPYLSNIINAAKIENERIIGVL	NleB1 alone	12 h (28 C)	6.28	0.104543	7.82
NleB1	68	79	KEHPYLSNIINA	NleB1 +YM155	10 s (23 C)	0.15	0.019032	6.06
NleB1	68	79	KEHPYLSNIINA	NleB1 +YM155	100 s (23 C)	0.20	0.006497	6.02
NleB1	68	79	KEHPYLSNIINA	NleB1 +YM155	1,000 s (23 C)	0.69	0.006135	6.03

NleB1	68	79	KEHPYLSNIINA	NleB1 +YM155	10,000 s (23 C)	2.33	0.055236	6.02
NleB1	68	79	KEHPYLSNIINA	NleB1 +YM155	12 h (28 C)	3.73	0.029662	6.02
NleB1	68	79	KEHPYLSNIINA	NleB1 alone	10 s (23 C)	0.10	0.015324	6.11
NleB1	68	79	KEHPYLSNIINA	NleB1 alone	100 s (23 C)	0.19	0.02259	6.09
NleB1	68	79	KEHPYLSNIINA	NleB1 alone	1,000 s (23 C)	0.58	0.011618	6.09
NleB1	68	79	KEHPYLSNIINA	NleB1 alone	10,000 s (23 C)	2.06	0.030112	6.09
NleB1	68	79	KEHPYLSNIINA	NleB1 alone	12 h (28 C)	3.70	0.030499	6.06
NleB1	68	91	KEHPYLSNIINAAKIENERIIGVL	NleB1 +YM155	10 s (23 C)	0.81	0.029114	7.87
NleB1	68	91	KEHPYLSNIINAAKIENERIIGVL	NleB1 +YM155	100 s (23 C)	1.05	0.035424	7.83
NleB1	68	91	KEHPYLSNIINAAKIENERIIGVL	NleB1 +YM155	1,000 s (23 C)	2.02	0.056686	7.83
NleB1	68	91	KEHPYLSNIINAAKIENERIIGVL	NleB1 +YM155	10,000 s (23 C)	3.55	0.076509	7.84
NleB1	68	91	KEHPYLSNIINAAKIENERIIGVL	NleB1 +YM155	12 h (28 C)	5.08	0.013501	7.83
NleB1	68	91	KEHPYLSNIINAAKIENERIIGVL	NleB1 alone	10 s (23 C)	0.72	0.046389	7.88
NleB1	68	91	KEHPYLSNIINAAKIENERIIGVL	NleB1 alone	100 s (23 C)	1.16	0.014276	7.83
NleB1	68	91	KEHPYLSNIINAAKIENERIIGVL	NleB1 alone	1,000 s (23 C)	2.00	0.019872	7.84
NleB1	68	91	KEHPYLSNIINAAKIENERIIGVL	NleB1 alone	10,000 s (23 C)	3.46	0.01701	7.85
NleB1	68	91	KEHPYLSNIINAAKIENERIIGVL	NleB1 alone	12 h (28 C)	4.95	0.003162	7.82
NleB1	76	85	IINAAKIENE	NleB1 +YM155	10 s (23 C)	0.64	0.015901	4.71
NleB1	76	85	IINAAKIENE	NleB1 +YM155	100 s (23 C)	1.08	0.010328	4.67
NleB1	76	85	IINAAKIENE	NleB1 +YM155	1,000 s (23 C)	1.41	0.017989	4.68
NleB1	76	85	IINAAKIENE	NleB1 +YM155	10,000 s (23 C)	1.46	0.012774	4.68
NleB1	76	85	IINAAKIENE	NleB1 +YM155	12 h (28 C)	3.04	0.083425	4.67
NleB1	76	85	IINAAKIENE	NleB1 alone	10 s (23 C)	0.61	0.026778	4.72
NleB1	76	85	IINAAKIENE	NleB1 alone	100 s (23 C)	1.04	0.030525	4.69
NleB1	76	85	IINAAKIENE	NleB1 alone	1,000 s (23 C)	1.38	0.007457	4.70
NleB1	76	85	IINAAKIENE	NleB1 alone	10,000 s (23 C)	1.38	0.018982	4.70
NleB1	76	85	IINAAKIENE	NleB1 alone	12 h (28 C)	3.00	0.060834	4.68
NleB1	76	91	IINAAKIENERIIGVL	NleB1 +YM155	10 s (23 C)	0.66	0.005512	6.87

NleB1	76	91	IINAAKIENERIIGVL	NleB1 +YM155	100 s (23 C)	1.04	0.050377	6.84
NleB1	76	91	IINAAKIENERIIGVL	NleB1 +YM155	1,000 s (23 C)	1.48	0.023606	6.86
NleB1	76	91	IINAAKIENERIIGVL	NleB1 +YM155	10,000 s (23 C)	1.59	0.053836	6.86
NleB1	76	91	IINAAKIENERIIGVL	NleB1 +YM155	12 h (28 C)	2.27	0.00887	6.86
NleB1	76	91	IINAAKIENERIIGVL	NleB1 alone	10 s (23 C)	0.66	0.039315	6.91
NleB1	76	91	IINAAKIENERIIGVL	NleB1 alone	100 s (23 C)	1.08	0.02561	6.88
NleB1	76	91	IINAAKIENERIIGVL	NleB1 alone	1,000 s (23 C)	1.45	0.01519	6.89
NleB1	76	91	IINAAKIENERIIGVL	NleB1 alone	10,000 s (23 C)	1.60	0.014608	6.89
NleB1	76	91	IINAAKIENERIIGVL	NleB1 alone	12 h (28 C)	2.31	0.052969	6.88
NleB1	80	85	AKIENE	NleB1 +YM155	10 s (23 C)	0.75	0.015593	2.50
NleB1	80	85	AKIENE	NleB1 +YM155	100 s (23 C)	1.12	0.022205	2.47
NleB1	80	85	AKIENE	NleB1 +YM155	1,000 s (23 C)	1.45	0.011845	2.48
NleB1	80	85	AKIENE	NleB1 +YM155	10,000 s (23 C)	1.47	0.02506	2.49
NleB1	80	85	AKIENE	NleB1 +YM155	12 h (28 C)	2.00	0.013417	2.47
NleB1	80	85	AKIENE	NleB1 alone	10 s (23 C)	0.62	0.008683	2.50
NleB1	80	85	AKIENE	NleB1 alone	100 s (23 C)	1.03	0.019919	2.47
NleB1	80	85	AKIENE	NleB1 alone	1,000 s (23 C)	1.37	0.011163	2.48
NleB1	80	85	AKIENE	NleB1 alone	10,000 s (23 C)	1.40	0.014231	2.49
NleB1	80	85	AKIENE	NleB1 alone	12 h (28 C)	2.02	0.002361	2.47
NleB1	80	89	AKIENERIIG	NleB1 +YM155	10 s (23 C)	0.75	0.023046	4.58
NleB1	80	89	AKIENERIIG	NleB1 +YM155	100 s (23 C)	0.99	0.016989	4.54
NleB1	80	89	AKIENERIIG	NleB1 +YM155	1,000 s (23 C)	1.32	0.031601	4.56
NleB1	80	89	AKIENERIIG	NleB1 +YM155	10,000 s (23 C)	1.44	0.010437	4.56
NleB1	80	89	AKIENERIIG	NleB1 +YM155	12 h (28 C)	3.20	0.098585	4.55
NleB1	80	89	AKIENERIIG	NleB1 alone	10 s (23 C)	0.64	0.013127	4.59
NleB1	80	89	AKIENERIIG	NleB1 alone	100 s (23 C)	0.99	0.046268	4.56
NleB1	80	89	AKIENERIIG	NleB1 alone	1,000 s (23 C)	1.29	0.00683	4.57
NleB1	80	89	AKIENERIIG	NleB1 alone	10,000 s (23 C)	1.40	0.002834	4.57

NleB1	80	89	AKIENERIIG	NleB1 alone	12 h (28 C)	3.18	0.023284	4.55
NleB1	80	91	AKIENERIIGVL	NleB1 +YM155	10 s (23 C)	0.42	0.033139	6.32
NleB1	80	91	AKIENERIIGVL	NleB1 +YM155	100 s (23 C)	0.59	0.020766	6.29
NleB1	80	91	AKIENERIIGVL	NleB1 +YM155	1,000 s (23 C)	0.80	0.040185	6.29
NleB1	80	91	AKIENERIIGVL	NleB1 +YM155	10,000 s (23 C)	0.97	0.047408	6.30
NleB1	80	91	AKIENERIIGVL	NleB1 +YM155	12 h (28 C)	2.10	0.049884	6.29
NleB1	80	91	AKIENERIIGVL	NleB1 alone	10 s (23 C)	0.35	0.005837	6.38
NleB1	80	91	AKIENERIIGVL	NleB1 alone	100 s (23 C)	0.55	0.046323	6.36
NleB1	80	91	AKIENERIIGVL	NleB1 alone	1,000 s (23 C)	0.67	0.02639	6.37
NleB1	80	91	AKIENERIIGVL	NleB1 alone	10,000 s (23 C)	0.79	0.015173	6.37
NleB1	80	91	AKIENERIIGVL	NleB1 alone	12 h (28 C)	2.18	0.0317	6.35
NleB1	85	91	ERIIGVL	NleB1 +YM155	10 s (23 C)	0.10	0.012577	6.52
NleB1	85	91	ERIIGVL	NleB1 +YM155	100 s (23 C)	0.09	0.026108	6.49
NleB1	85	91	ERIIGVL	NleB1 +YM155	1,000 s (23 C)	0.13	0.016485	6.48
NleB1	85	91	ERIIGVL	NleB1 +YM155	10,000 s (23 C)	0.14	0.031129	6.48
NleB1	85	91	ERIIGVL	NleB1 +YM155	12 h (28 C)	1.17	0.016167	6.48
NleB1	85	91	ERIIGVL	NleB1 alone	10 s (23 C)	0.09	0.01099	6.63
NleB1	85	91	ERIIGVL	NleB1 alone	100 s (23 C)	0.10	0.026145	6.60
NleB1	85	91	ERIIGVL	NleB1 alone	1,000 s (23 C)	0.09	0.019724	6.62
NleB1	85	91	ERIIGVL	NleB1 alone	10,000 s (23 C)	0.11	0.008755	6.62
NleB1	85	91	ERIIGVL	NleB1 alone	12 h (28 C)	1.12	0.039464	6.60
NleB1	86	91	RIIGVL	NleB1 +YM155	10 s (23 C)	0.30	0.036615	6.29
NleB1	86	91	RIIGVL	NleB1 +YM155	100 s (23 C)	0.42	0.025503	6.27
NleB1	86	91	RIIGVL	NleB1 +YM155	1,000 s (23 C)	0.34	0.010589	6.26
NleB1	86	91	RIIGVL	NleB1 +YM155	10,000 s (23 C)	0.43	0.038071	6.26
NleB1	86	91	RIIGVL	NleB1 +YM155	12 h (28 C)	1.18	0.059846	6.26
NleB1	86	91	RIIGVL	NleB1 alone	10 s (23 C)	0.16	0.011673	6.39
NleB1	86	91	RIIGVL	NleB1 alone	100 s (23 C)	0.22	0.039382	6.36

NleB1	86	91	RIIGVL	NleB1 alone	1,000 s (23 C)	0.19	0.012955	6.37
NleB1	86	91	RIIGVL	NleB1 alone	10,000 s (23 C)	0.24	0.02836	6.38
NleB1	86	91	RIIGVL	NleB1 alone	12 h (28 C)	1.26	0.046744	6.36
NleB1	90	96	VLVDGNF	NleB1 +YM155	10 s (23 C)	0.95	0.024267	6.52
NleB1	90	96	VLVDGNF	NleB1 +YM155	100 s (23 C)	1.72	0.024982	6.49
NleB1	90	96	VLVDGNF	NleB1 +YM155	1,000 s (23 C)	2.08	0.018339	6.49
NleB1	90	96	VLVDGNF	NleB1 +YM155	10,000 s (23 C)	2.18	0.00822	6.49
NleB1	90	96	VLVDGNF	NleB1 +YM155	12 h (28 C)	2.73	0.025838	6.49
NleB1	90	96	VLVDGNF	NleB1 alone	10 s (23 C)	0.88	0.016848	6.60
NleB1	90	96	VLVDGNF	NleB1 alone	100 s (23 C)	1.67	0.025168	6.57
NleB1	90	96	VLVDGNF	NleB1 alone	1,000 s (23 C)	2.05	0.022428	6.58
NleB1	90	96	VLVDGNF	NleB1 alone	10,000 s (23 C)	2.11	0.026402	6.59
NleB1	90	96	VLVDGNF	NleB1 alone	12 h (28 C)	2.75	0.0132	6.57
NleB1	92	104	VDGNFTYEQKKEF	NleB1 +YM155	10 s (23 C)	2.25	0.048387	5.32
NleB1	92	104	VDGNFTYEQKKEF	NleB1 +YM155	100 s (23 C)	3.19	0.015114	5.28
NleB1	92	104	VDGNFTYEQKKEF	NleB1 +YM155	1,000 s (23 C)	4.88	0.039774	5.29
NleB1	92	104	VDGNFTYEQKKEF	NleB1 +YM155	10,000 s (23 C)	5.98	0.084871	5.29
NleB1	92	104	VDGNFTYEQKKEF	NleB1 +YM155	12 h (28 C)	6.89	0.037672	5.28
NleB1	92	104	VDGNFTYEQKKEF	NleB1 alone	10 s (23 C)	2.17	0.021789	5.35
NleB1	92	104	VDGNFTYEQKKEF	NleB1 alone	100 s (23 C)	3.13	0.063604	5.32
NleB1	92	104	VDGNFTYEQKKEF	NleB1 alone	1,000 s (23 C)	4.78	0.031984	5.33
NleB1	92	104	VDGNFTYEQKKEF	NleB1 alone	10,000 s (23 C)	5.97	0.06302	5.34
NleB1	92	104	VDGNFTYEQKKEF	NleB1 alone	12 h (28 C)	6.87	0.106549	5.32
NleB1	93	104	DGNFTYEQKKEF	NleB1 +YM155	10 s (23 C)	2.11	0.073632	5.32
NleB1	93	104	DGNFTYEQKKEF	NleB1 +YM155	100 s (23 C)	2.84	0.044678	5.28
NleB1	93	104	DGNFTYEQKKEF	NleB1 +YM155	1,000 s (23 C)	4.61	0.057103	5.29
NleB1	93	104	DGNFTYEQKKEF	NleB1 +YM155	10,000 s (23 C)	5.57	ND	5.30
NleB1	93	104	DGNFTYEQKKEF	NleB1 +YM155	12 h (28 C)	6.58	0.01797	5.28

NleB1	93	104	DGNFTYEQKKEF	NleB1 alone	10 s (23 C)	2.06	0.03321	5.35
NleB1	93	104	DGNFTYEQKKEF	NleB1 alone	100 s (23 C)	2.86	0.009592	5.33
NleB1	93	104	DGNFTYEQKKEF	NleB1 alone	1,000 s (23 C)	4.58	0.04206	5.34
NleB1	93	104	DGNFTYEQKKEF	NleB1 alone	10,000 s (23 C)	5.51	0.010681	5.35
NleB1	93	104	DGNFTYEQKKEF	NleB1 alone	12 h (28 C)	6.59	0.002662	5.32
NleB1	97	104	TYEQKKEF	NleB1 +YM155	10 s (23 C)	0.85	0.063467	3.98
NleB1	97	104	TYEQKKEF	NleB1 +YM155	100 s (23 C)	0.98	0.057132	3.94
NleB1	97	104	TYEQKKEF	NleB1 +YM155	1,000 s (23 C)	1.64	0.005465	3.95
NleB1	97	104	TYEQKKEF	NleB1 +YM155	10,000 s (23 C)	2.41	0.039914	3.96
NleB1	97	104	TYEQKKEF	NleB1 +YM155	12 h (28 C)	2.90	0.031466	3.94
NleB1	97	104	TYEQKKEF	NleB1 alone	10 s (23 C)	0.60	0.01271	3.98
NleB1	97	104	TYEQKKEF	NleB1 alone	100 s (23 C)	0.83	0.066881	3.95
NleB1	97	104	TYEQKKEF	NleB1 alone	1,000 s (23 C)	1.55	0.017227	3.95
NleB1	97	104	TYEQKKEF	NleB1 alone	10,000 s (23 C)	2.41	0.039153	3.97
NleB1	97	104	TYEQKKEF	NleB1 alone	12 h (28 C)	3.04	0.051724	3.93
NleB1	98	104	YEQKKEF	NleB1 +YM155	10 s (23 C)	0.76	0.043365	3.98
NleB1	98	104	YEQKKEF	NleB1 +YM155	100 s (23 C)	0.90	0.040423	3.95
NleB1	98	104	YEQKKEF	NleB1 +YM155	1,000 s (23 C)	1.62	0.018905	3.96
NleB1	98	104	YEQKKEF	NleB1 +YM155	10,000 s (23 C)	2.41	0.024019	3.96
NleB1	98	104	YEQKKEF	NleB1 +YM155	12 h (28 C)	3.03	0.019317	3.94
NleB1	98	104	YEQKKEF	NleB1 alone	10 s (23 C)	0.58	0.008813	3.98
NleB1	98	104	YEQKKEF	NleB1 alone	100 s (23 C)	0.80	0.011853	3.95
NleB1	98	104	YEQKKEF	NleB1 alone	1,000 s (23 C)	1.59	0.017396	3.95
NleB1	98	104	YEQKKEF	NleB1 alone	10,000 s (23 C)	2.44	0.020692	3.97
NleB1	98	104	YEQKKEF	NleB1 alone	12 h (28 C)	3.12	0.020131	3.93
NleB1	99	104	EQKKEF	NleB1 +YM155	10 s (23 C)	0.65	0.033203	3.98
NleB1	99	104	EQKKEF	NleB1 +YM155	100 s (23 C)	0.89	0.03426	3.95
NleB1	99	104	EQKKEF	NleB1 +YM155	1,000 s (23 C)	1.65	0.010215	3.96

NleB1	99	104	EQKKEF	NleB1 +YM155	10,000 s (23 C)	2.38	0.021849	3.96
NleB1	99	104	EQKKEF	NleB1 +YM155	12 h (28 C)	2.96	0.048439	3.94
NleB1	99	104	EQKKEF	NleB1 alone	10 s (23 C)	0.60	0.018955	3.99
NleB1	99	104	EQKKEF	NleB1 alone	100 s (23 C)	0.80	0.046756	3.96
NleB1	99	104	EQKKEF	NleB1 alone	1,000 s (23 C)	1.56	0.014676	3.95
NleB1	99	104	EQKKEF	NleB1 alone	10,000 s (23 C)	2.36	0.015093	3.97
NleB1	99	104	EQKKEF	NleB1 alone	12 h (28 C)	3.00	0.032709	3.93
NleB1	116	123	IIYRADVD	NleB1 +YM155	10 s (23 C)	0.82	0.020745	4.66
NleB1	116	123	IIYRADVD	NleB1 +YM155	100 s (23 C)	1.59	0.030663	4.62
NleB1	116	123	IIYRADVD	NleB1 +YM155	1,000 s (23 C)	1.95	0.005011	4.64
NleB1	116	123	IIYRADVD	NleB1 +YM155	10,000 s (23 C)	2.46	0.062562	4.64
NleB1	116	123	IIYRADVD	NleB1 +YM155	12 h (28 C)	3.12	0.008781	4.62
NleB1	116	123	IIYRADVD	NleB1 alone	10 s (23 C)	0.71	0.038058	4.67
NleB1	116	123	IIYRADVD	NleB1 alone	100 s (23 C)	1.50	0.018655	4.64
NleB1	116	123	IIYRADVD	NleB1 alone	1,000 s (23 C)	1.89	0.013747	4.64
NleB1	116	123	IIYRADVD	NleB1 alone	10,000 s (23 C)	2.39	0.007768	4.65
NleB1	116	123	IIYRADVD	NleB1 alone	12 h (28 C)	3.15	0.01541	4.62
NleB1	116	124	IIYRADVDF	NleB1 +YM155	10 s (23 C)	1.52	0.0232	6.56
NleB1	116	124	IIYRADVDF	NleB1 +YM155	100 s (23 C)	2.36	0.019812	6.53
NleB1	116	124	IIYRADVDF	NleB1 +YM155	1,000 s (23 C)	2.77	0.020672	6.52
NleB1	116	124	IIYRADVDF	NleB1 +YM155	10,000 s (23 C)	3.37	0.029084	6.52
NleB1	116	124	IIYRADVDF	NleB1 +YM155	12 h (28 C)	4.23	0.020022	6.51
NleB1	116	124	IIYRADVDF	NleB1 alone	10 s (23 C)	1.41	0.029763	6.64
NleB1	116	124	IIYRADVDF	NleB1 alone	100 s (23 C)	2.29	0.016917	6.61
NleB1	116	124	IIYRADVDF	NleB1 alone	1,000 s (23 C)	2.72	0.011628	6.62
NleB1	116	124	IIYRADVDF	NleB1 alone	10,000 s (23 C)	3.32	0.018556	6.63
NleB1	116	124	IIYRADVDF	NleB1 alone	12 h (28 C)	4.20	0.024187	6.61
NleB1	116	126	IIYRADVDFSM	NleB1 +YM155	10 s (23 C)	2.31	0.054132	7.06

NleB1	116	126	IIYRADVDFSM	NleB1 +YM155	100 s (23 C)	3.55	0.047366	7.03
NleB1	116	126	IIYRADVDFSM	NleB1 +YM155	1,000 s (23 C)	4.13	0.03217	7.05
NleB1	116	126	IIYRADVDFSM	NleB1 +YM155	10,000 s (23 C)	4.79	0.02932	7.05
NleB1	116	126	IIYRADVDFSM	NleB1 +YM155	12 h (28 C)	5.62	0.017061	7.04
NleB1	116	126	IIYRADVDFSM	NleB1 alone	10 s (23 C)	2.19	0.006241	7.07
NleB1	116	126	IIYRADVDFSM	NleB1 alone	100 s (23 C)	3.54	0.044563	7.05
NleB1	116	126	IIYRADVDFSM	NleB1 alone	1,000 s (23 C)	4.02	0.018756	7.06
NleB1	116	126	IIYRADVDFSM	NleB1 alone	10,000 s (23 C)	4.70	0.010016	7.06
NleB1	116	126	IIYRADVDFSM	NleB1 alone	12 h (28 C)	5.63	0.003537	7.04
NleB1	117	123	IYRADVD	NleB1 +YM155	10 s (23 C)	0.73	0.031522	3.96
NleB1	117	123	IYRADVD	NleB1 +YM155	100 s (23 C)	1.43	0.028993	3.92
NleB1	117	123	IYRADVD	NleB1 +YM155	1,000 s (23 C)	1.49	0.01925	3.93
NleB1	117	123	IYRADVD	NleB1 +YM155	10,000 s (23 C)	1.66	0.030087	3.94
NleB1	117	123	IYRADVD	NleB1 +YM155	12 h (28 C)	2.37	0.057379	3.92
NleB1	117	123	IYRADVD	NleB1 alone	10 s (23 C)	0.65	0.01706	3.96
NleB1	117	123	IYRADVD	NleB1 alone	100 s (23 C)	1.36	0.006326	3.93
NleB1	117	123	IYRADVD	NleB1 alone	1,000 s (23 C)	1.37	0.026751	3.93
NleB1	117	123	IYRADVD	NleB1 alone	10,000 s (23 C)	1.60	0.010443	3.94
NleB1	117	123	IYRADVD	NleB1 alone	12 h (28 C)	2.41	0.005601	3.91
NleB1	117	124	IYRADVDF	NleB1 +YM155	10 s (23 C)	1.41	0.055412	6.21
NleB1	117	124	IYRADVDF	NleB1 +YM155	100 s (23 C)	2.18	0.003956	6.17
NleB1	117	124	IYRADVDF	NleB1 +YM155	1,000 s (23 C)	2.20	0.03172	6.18
NleB1	117	124	IYRADVDF	NleB1 +YM155	10,000 s (23 C)	2.41	0.044813	6.18
NleB1	117	124	IYRADVDF	NleB1 +YM155	12 h (28 C)	3.20	0.007848	6.18
NleB1	117	124	IYRADVDF	NleB1 alone	10 s (23 C)	1.34	0.0156	6.27
NleB1	117	124	IYRADVDF	NleB1 alone	100 s (23 C)	2.15	0.004813	6.25
NleB1	117	124	IYRADVDF	NleB1 alone	1,000 s (23 C)	2.13	0.010714	6.26
NleB1	117	124	IYRADVDF	NleB1 alone	10,000 s (23 C)	2.36	0.018829	6.27

NleB1	117	124	IYRADVDF	NleB1 alone	12 h (28 C)	3.21	0.000412	6.24
NleB1	117	126	IYRADVDFSM	NleB1 +YM155	10 s (23 C)	2.20	0.04911	6.73
NleB1	117	126	IYRADVDFSM	NleB1 +YM155	100 s (23 C)	3.39	0.055783	6.70
NleB1	117	126	IYRADVDFSM	NleB1 +YM155	1,000 s (23 C)	3.53	0.038148	6.72
NleB1	117	126	IYRADVDFSM	NleB1 +YM155	10,000 s (23 C)	3.77	0.021673	6.71
NleB1	117	126	IYRADVDFSM	NleB1 +YM155	12 h (28 C)	4.55	0.016389	6.71
NleB1	117	126	IYRADVDFSM	NleB1 alone	10 s (23 C)	2.06	0.026644	6.81
NleB1	117	126	IYRADVDFSM	NleB1 alone	100 s (23 C)	3.34	0.037527	6.78
NleB1	117	126	IYRADVDFSM	NleB1 alone	1,000 s (23 C)	3.45	0.013541	6.79
NleB1	117	126	IYRADVDFSM	NleB1 alone	10,000 s (23 C)	3.70	0.022853	6.80
NleB1	117	126	IYRADVDFSM	NleB1 alone	12 h (28 C)	4.63	0.009302	6.78
NleB1	118	124	YRADVDF	NleB1 +YM155	10 s (23 C)	1.20	0.033218	5.96
NleB1	118	124	YRADVDF	NleB1 +YM155	100 s (23 C)	1.85	0.010411	5.91
NleB1	118	124	YRADVDF	NleB1 +YM155	1,000 s (23 C)	1.89	0.011411	5.93
NleB1	118	124	YRADVDF	NleB1 +YM155	10,000 s (23 C)	2.07	0.021463	5.93
NleB1	118	124	YRADVDF	NleB1 +YM155	12 h (28 C)	2.54	0.014246	5.92
NleB1	118	124	YRADVDF	NleB1 alone	10 s (23 C)	1.14	0.024469	6.02
NleB1	118	124	YRADVDF	NleB1 alone	100 s (23 C)	1.85	0.022254	5.99
NleB1	118	124	YRADVDF	NleB1 alone	1,000 s (23 C)	1.85	0.014558	6.00
NleB1	118	124	YRADVDF	NleB1 alone	10,000 s (23 C)	2.07	0.004437	6.01
NleB1	118	124	YRADVDF	NleB1 alone	12 h (28 C)	2.59	0.02468	5.98
NleB1	119	124	RADVDF	NleB1 +YM155	10 s (23 C)	0.99	0.031337	5.42
NleB1	119	124	RADVDF	NleB1 +YM155	100 s (23 C)	1.25	0.00919	5.37
NleB1	119	124	RADVDF	NleB1 +YM155	1,000 s (23 C)	1.29	0.018415	5.39
NleB1	119	124	RADVDF	NleB1 +YM155	10,000 s (23 C)	1.40	0.024883	5.39
NleB1	119	124	RADVDF	NleB1 +YM155	12 h (28 C)	1.74	0.020239	5.38
NleB1	119	124	RADVDF	NleB1 alone	10 s (23 C)	0.99	0.01306	5.43
NleB1	119	124	RADVDF	NleB1 alone	100 s (23 C)	1.29	0.017136	5.40

NleB1	119	124	RADVDF	NleB1 alone	1,000 s (23 C)	1.30	0.010037	5.41
NleB1	119	124	RADVDF	NleB1 alone	10,000 s (23 C)	1.46	0.002476	5.42
NleB1	119	124	RADVDF	NleB1 alone	12 h (28 C)	1.90	0.012202	5.39
NleB1	119	126	RADVDFSM	NleB1 +YM155	10 s (23 C)	2.01	0.059237	6.19
NleB1	119	126	RADVDFSM	NleB1 +YM155	100 s (23 C)	2.74	0.021972	6.15
NleB1	119	126	RADVDFSM	NleB1 +YM155	1,000 s (23 C)	2.90	0.020622	6.16
NleB1	119	126	RADVDFSM	NleB1 +YM155	10,000 s (23 C)	3.05	0.035975	6.17
NleB1	119	126	RADVDFSM	NleB1 +YM155	12 h (28 C)	3.39	0.016356	6.16
NleB1	119	126	RADVDFSM	NleB1 alone	10 s (23 C)	1.94	0.016745	6.24
NleB1	119	126	RADVDFSM	NleB1 alone	100 s (23 C)	2.77	0.022505	6.21
NleB1	119	126	RADVDFSM	NleB1 alone	1,000 s (23 C)	2.84	0.019512	6.22
NleB1	119	126	RADVDFSM	NleB1 alone	10,000 s (23 C)	3.05	0.017587	6.23
NleB1	119	126	RADVDFSM	NleB1 alone	12 h (28 C)	3.47	0.045231	6.20
NleB1	125	133	SMYDKKLSD	NleB1 +YM155	10 s (23 C)	0.58	0.044994	4.07
NleB1	125	133	SMYDKKLSD	NleB1 +YM155	100 s (23 C)	0.94	0.035316	4.03
NleB1	125	133	SMYDKKLSD	NleB1 +YM155	1,000 s (23 C)	1.46	0.025082	4.04
NleB1	125	133	SMYDKKLSD	NleB1 +YM155	10,000 s (23 C)	2.50	0.047288	4.04
NleB1	125	133	SMYDKKLSD	NleB1 +YM155	12 h (28 C)	3.02	0.018476	4.02
NleB1	125	133	SMYDKKLSD	NleB1 alone	10 s (23 C)	0.37	0.041155	4.07
NleB1	125	133	SMYDKKLSD	NleB1 alone	100 s (23 C)	0.80	0.017614	4.04
NleB1	125	133	SMYDKKLSD	NleB1 alone	1,000 s (23 C)	1.36	0.071714	4.04
NleB1	125	133	SMYDKKLSD	NleB1 alone	10,000 s (23 C)	2.42	0.058487	4.06
NleB1	125	133	SMYDKKLSD	NleB1 alone	12 h (28 C)	3.10	0.014711	4.02
NleB1	134	149	IYLENIHKQESYPASE	NleB1 +YM155	10 s (23 C)	3.02	0.019711	5.07
NleB1	134	149	IYLENIHKQESYPASE	NleB1 +YM155	100 s (23 C)	4.84	0.021717	5.03
NleB1	134	149	IYLENIHKQESYPASE	NleB1 +YM155	1,000 s (23 C)	6.22	0.067077	5.05
NleB1	134	149	IYLENIHKQESYPASE	NleB1 +YM155	10,000 s (23 C)	7.46	0.073092	5.05
NleB1	134	149	IYLENIHKQESYPASE	NleB1 +YM155	12 h (28 C)	8.16	0.061981	5.04

NleB1	134	149	IYLENIHKQESYPASE	NleB1 alone	10 s (23 C)	2.89	0.006647	5.10
NleB1	134	149	IYLENIHKQESYPASE	NleB1 alone	100 s (23 C)	4.74	0.037736	5.07
NleB1	134	149	IYLENIHKQESYPASE	NleB1 alone	1,000 s (23 C)	6.10	0.02996	5.07
NleB1	134	149	IYLENIHKQESYPASE	NleB1 alone	10,000 s (23 C)	7.50	0.016103	5.08
NleB1	134	149	IYLENIHKQESYPASE	NleB1 alone	12 h (28 C)	8.14	0.155805	5.06
NleB1	134	151	IYLENIHKQESYPASERD	NleB1 +YM155	10 s (23 C)	4.23	0.088391	4.73
NleB1	134	151	IYLENIHKQESYPASERD	NleB1 +YM155	100 s (23 C)	6.25	0.036932	4.70
NleB1	134	151	IYLENIHKQESYPASERD	NleB1 +YM155	1,000 s (23 C)	7.49	0.063003	4.70
NleB1	134	151	IYLENIHKQESYPASERD	NleB1 +YM155	10,000 s (23 C)	8.77	0.102484	4.70
NleB1	134	151	IYLENIHKQESYPASERD	NleB1 +YM155	12 h (28 C)	9.50	0.004858	4.70
NleB1	134	151	IYLENIHKQESYPASERD	NleB1 alone	10 s (23 C)	4.22	0.059422	4.75
NleB1	134	151	IYLENIHKQESYPASERD	NleB1 alone	100 s (23 C)	6.26	0.050997	4.72
NleB1	134	151	IYLENIHKQESYPASERD	NleB1 alone	1,000 s (23 C)	7.49	0.047074	4.73
NleB1	134	151	IYLENIHKQESYPASERD	NleB1 alone	10,000 s (23 C)	8.83	0.048346	4.73
NleB1	134	151	IYLENIHKQESYPASERD	NleB1 alone	12 h (28 C)	9.53	0.151977	4.71
NleB1	134	153	IYLENIHKQESYPASERDNY	NleB1 +YM155	10 s (23 C)	4.59	0.062629	5.39
NleB1	134	153	IYLENIHKQESYPASERDNY	NleB1 +YM155	100 s (23 C)	6.57	0.032692	5.36
NleB1	134	153	IYLENIHKQESYPASERDNY	NleB1 +YM155	1,000 s (23 C)	7.82	0.042353	5.37
NleB1	134	153	IYLENIHKQESYPASERDNY	NleB1 +YM155	10,000 s (23 C)	8.69	0.095021	5.36
NleB1	134	153	IYLENIHKQESYPASERDNY	NleB1 +YM155	12 h (28 C)	9.01	0.08533	5.36
NleB1	134	153	IYLENIHKQESYPASERDNY	NleB1 alone	10 s (23 C)	4.57	0.079382	5.43
NleB1	134	153	IYLENIHKQESYPASERDNY	NleB1 alone	100 s (23 C)	6.63	0.030883	5.40
NleB1	134	153	IYLENIHKQESYPASERDNY	NleB1 alone	1,000 s (23 C)	7.87	0.055067	5.41
NleB1	134	153	IYLENIHKQESYPASERDNY	NleB1 alone	10,000 s (23 C)	8.58	0.204182	5.41
NleB1	134	153	IYLENIHKQESYPASERDNY	NleB1 alone	12 h (28 C)	8.77	0.085788	5.40
NleB1	134	154	IYLENIHKQESYPASERDNYL	NleB1 +YM155	10 s (23 C)	5.48	0.110002	5.78
NleB1	134	154	IYLENIHKQESYPASERDNYL	NleB1 +YM155	100 s (23 C)	7.91	0.04038	5.75
NleB1	134	154	IYLENIHKQESYPASERDNYL	NleB1 +YM155	1,000 s (23 C)	9.42	0.080131	5.75

NleB1	134	154	IYLENIHKQESYPASERDNYL	NleB1 +YM155	10,000 s (23 C)	10.64	0.105698	5.76
NleB1	134	154	IYLENIHKQESYPASERDNYL	NleB1 +YM155	12 h (28 C)	11.49	0.018796	5.75
NleB1	134	154	IYLENIHKQESYPASERDNYL	NleB1 alone	10 s (23 C)	5.45	0.022824	5.82
NleB1	134	154	IYLENIHKQESYPASERDNYL	NleB1 alone	100 s (23 C)	7.82	0.036144	5.79
NleB1	134	154	IYLENIHKQESYPASERDNYL	NleB1 alone	1,000 s (23 C)	9.30	0.033282	5.80
NleB1	134	154	IYLENIHKQESYPASERDNYL	NleB1 alone	10,000 s (23 C)	10.64	0.023448	5.80
NleB1	134	154	IYLENIHKQESYPASERDNYL	NleB1 alone	12 h (28 C)	11.39	0.168134	5.78
NleB1	134	157	IYLENIHKQESYPASERDNYLLGL	NleB1 +YM155	10 s (23 C)	4.54	0.029713	7.07
NleB1	134	157	IYLENIHKQESYPASERDNYLLGL	NleB1 +YM155	100 s (23 C)	6.97	0.055809	7.04
NleB1	134	157	IYLENIHKQESYPASERDNYLLGL	NleB1 +YM155	1,000 s (23 C)	9.54	0.074058	7.06
NleB1	134	157	IYLENIHKQESYPASERDNYLLGL	NleB1 +YM155	10,000 s (23 C)	10.87	0.083978	7.06
NleB1	134	157	IYLENIHKQESYPASERDNYLLGL	NleB1 +YM155	12 h (28 C)	11.91	0.03735	7.06
NleB1	134	157	IYLENIHKQESYPASERDNYLLGL	NleB1 alone	10 s (23 C)	4.46	0.069325	7.08
NleB1	134	157	IYLENIHKQESYPASERDNYLLGL	NleB1 alone	100 s (23 C)	7.18	0.031555	7.06
NleB1	134	157	IYLENIHKQESYPASERDNYLLGL	NleB1 alone	1,000 s (23 C)	9.39	0.027532	7.07
NleB1	134	157	IYLENIHKQESYPASERDNYLLGL	NleB1 alone	10,000 s (23 C)	10.88	0.111254	7.07
NleB1	134	157	IYLENIHKQESYPASERDNYLLGL	NleB1 alone	12 h (28 C)	11.82	0.254932	7.05
NleB1	134	158	IYLENIHKQESYPASERDNYLLGLL	NleB1 +YM155	10 s (23 C)	4.67	0.089197	7.85
NleB1	134	158	IYLENIHKQESYPASERDNYLLGLL	NleB1 +YM155	100 s (23 C)	8.01	0.053668	7.82
NleB1	134	158	IYLENIHKQESYPASERDNYLLGLL	NleB1 +YM155	1,000 s (23 C)	10.43	0.030286	7.83
NleB1	134	158	IYLENIHKQESYPASERDNYLLGLL	NleB1 +YM155	10,000 s (23 C)	12.13	0.067798	7.83
NleB1	134	158	IYLENIHKQESYPASERDNYLLGLL	NleB1 +YM155	12 h (28 C)	13.01	0.03615	7.82
NleB1	134	158	IYLENIHKQESYPASERDNYLLGLL	NleB1 alone	10 s (23 C)	4.66	0.021391	7.85
NleB1	134	158	IYLENIHKQESYPASERDNYLLGLL	NleB1 alone	100 s (23 C)	8.04	0.099252	7.83
NleB1	134	158	IYLENIHKQESYPASERDNYLLGLL	NleB1 alone	1,000 s (23 C)	10.37	0.033968	7.83
NleB1	134	158	IYLENIHKQESYPASERDNYLLGLL	NleB1 alone	10,000 s (23 C)	12.00	0.078768	7.84
NleB1	134	158	IYLENIHKQESYPASERDNYLLGLL	NleB1 alone	12 h (28 C)	12.90	0.203011	7.82
NleB1	136	149	LENIHKQESYPASE	NleB1 +YM155	10 s (23 C)	2.69	0.054437	5.07

NleB1	136	149	LENIHKQESYPASE	NleB1 +YM155	100 s (23 C)	4.40	0.052233	5.04
NleB1	136	149	LENIHKQESYPASE	NleB1 +YM155	1,000 s (23 C)	5.69	0.114811	5.05
NleB1	136	149	LENIHKQESYPASE	NleB1 +YM155	10,000 s (23 C)	6.85	0.025973	5.05
NleB1	136	149	LENIHKQESYPASE	NleB1 +YM155	12 h (28 C)	7.37	0.041281	5.04
NleB1	136	149	LENIHKQESYPASE	NleB1 alone	10 s (23 C)	2.61	0.019065	5.09
NleB1	136	149	LENIHKQESYPASE	NleB1 alone	100 s (23 C)	4.29	0.039536	5.07
NleB1	136	149	LENIHKQESYPASE	NleB1 alone	1,000 s (23 C)	5.68	0.029533	5.07
NleB1	136	149	LENIHKQESYPASE	NleB1 alone	10,000 s (23 C)	6.90	0.060458	5.08
NleB1	136	149	LENIHKQESYPASE	NleB1 alone	12 h (28 C)	7.40	0.05997	5.06
NleB1	136	153	LENIHKQESYPASERDNY	NleB1 +YM155	10 s (23 C)	5.60	0.069646	4.14
NleB1	136	153	LENIHKQESYPASERDNY	NleB1 +YM155	100 s (23 C)	7.46	0.097563	4.11
NleB1	136	153	LENIHKQESYPASERDNY	NleB1 +YM155	1,000 s (23 C)	8.38	0.022802	4.12
NleB1	136	153	LENIHKQESYPASERDNY	NleB1 +YM155	10,000 s (23 C)	8.88	0.050411	4.12
NleB1	136	153	LENIHKQESYPASERDNY	NleB1 +YM155	12 h (28 C)	9.33	0.028698	4.11
NleB1	136	153	LENIHKQESYPASERDNY	NleB1 alone	10 s (23 C)	5.42	0.021196	4.15
NleB1	136	153	LENIHKQESYPASERDNY	NleB1 alone	100 s (23 C)	7.40	0.043306	4.12
NleB1	136	153	LENIHKQESYPASERDNY	NleB1 alone	1,000 s (23 C)	8.50	0.007995	4.12
NleB1	136	153	LENIHKQESYPASERDNY	NleB1 alone	10,000 s (23 C)	9.03	0.076673	4.14
NleB1	136	153	LENIHKQESYPASERDNY	NleB1 alone	12 h (28 C)	9.33	0.12273	4.10
NleB1	137	149	ENIHKQESYPASE	NleB1 +YM155	10 s (23 C)	3.04	0.036232	2.98
NleB1	137	149	ENIHKQESYPASE	NleB1 +YM155	100 s (23 C)	4.70	0.01988	3.31
NleB1	137	149	ENIHKQESYPASE	NleB1 +YM155	1,000 s (23 C)	5.62	0.013427	3.31
NleB1	137	149	ENIHKQESYPASE	NleB1 +YM155	10,000 s (23 C)	5.92	0.045094	3.30
NleB1	137	149	ENIHKQESYPASE	NleB1 +YM155	12 h (28 C)	6.15	0.05874	3.28
NleB1	137	149	ENIHKQESYPASE	NleB1 alone	10 s (23 C)	2.81	0.023338	3.34
NleB1	137	149	ENIHKQESYPASE	NleB1 alone	100 s (23 C)	4.64	0.009009	3.29
NleB1	137	149	ENIHKQESYPASE	NleB1 alone	1,000 s (23 C)	5.67	0.095786	2.86
NleB1	137	149	ENIHKQESYPASE	NleB1 alone	10,000 s (23 C)	6.02	0.011146	3.31

NleB1	137	149	ENIHKQESYPASE	NleB1 alone	12 h (28 C)	6.29	0.043933	2.69
NleB1	137	154	ENIHKQESYPASERDNYL	NleB1 +YM155	10 s (23 C)	5.55	0.09392	4.94
NleB1	137	154	ENIHKQESYPASERDNYL	NleB1 +YM155	100 s (23 C)	7.63	0.024555	4.90
NleB1	137	154	ENIHKQESYPASERDNYL	NleB1 +YM155	1,000 s (23 C)	8.69	0.071667	4.91
NleB1	137	154	ENIHKQESYPASERDNYL	NleB1 +YM155	10,000 s (23 C)	8.98	0.086585	4.91
NleB1	137	154	ENIHKQESYPASERDNYL	NleB1 +YM155	12 h (28 C)	9.35	0.05719	4.91
NleB1	137	154	ENIHKQESYPASERDNYL	NleB1 alone	10 s (23 C)	5.42	0.024715	4.97
NleB1	137	154	ENIHKQESYPASERDNYL	NleB1 alone	100 s (23 C)	7.65	0.023966	4.93
NleB1	137	154	ENIHKQESYPASERDNYL	NleB1 alone	1,000 s (23 C)	8.77	0.030148	4.94
NleB1	137	154	ENIHKQESYPASERDNYL	NleB1 alone	10,000 s (23 C)	9.11	0.02987	4.95
NleB1	137	154	ENIHKQESYPASERDNYL	NleB1 alone	12 h (28 C)	9.40	0.139521	4.93
NleB1	137	157	ENIHKQESYPASERDNYLLGL	NleB1 +YM155	10 s (23 C)	4.96	0.125634	6.67
NleB1	137	157	ENIHKQESYPASERDNYLLGL	NleB1 +YM155	100 s (23 C)	7.61	0.122053	6.64
NleB1	137	157	ENIHKQESYPASERDNYLLGL	NleB1 +YM155	1,000 s (23 C)	9.79	0.135491	6.66
NleB1	137	157	ENIHKQESYPASERDNYLLGL	NleB1 +YM155	10,000 s (23 C)	10.35	0.088136	6.65
NleB1	137	157	ENIHKQESYPASERDNYLLGL	NleB1 +YM155	12 h (28 C)	10.90	0.003142	6.65
NleB1	137	157	ENIHKQESYPASERDNYLLGL	NleB1 alone	10 s (23 C)	4.78	0.10563	6.75
NleB1	137	157	ENIHKQESYPASERDNYLLGL	NleB1 alone	100 s (23 C)	7.66	0.059346	6.73
NleB1	137	157	ENIHKQESYPASERDNYLLGL	NleB1 alone	1,000 s (23 C)	9.95	0.049247	6.74
NleB1	137	157	ENIHKQESYPASERDNYLLGL	NleB1 alone	10,000 s (23 C)	10.62	0.038627	6.74
NleB1	137	157	ENIHKQESYPASERDNYLLGL	NleB1 alone	12 h (28 C)	11.04	0.125749	6.73
NleB1	139	150	IHKQESYPASER	NleB1 +YM155	10 s (23 C)	1.86	0.057242	5.47
NleB1	139	150	IHKQESYPASER	NleB1 +YM155	100 s (23 C)	3.24	0.052226	5.43
NleB1	139	150	IHKQESYPASER	NleB1 +YM155	1,000 s (23 C)	4.15	0.011531	5.44
NleB1	139	150	IHKQESYPASER	NleB1 +YM155	10,000 s (23 C)	4.55	0.005567	5.44
NleB1	139	150	IHKQESYPASER	NleB1 +YM155	12 h (28 C)	4.57	0.022129	5.44
NleB1	139	150	IHKQESYPASER	NleB1 alone	10 s (23 C)	1.71	0.005447	5.50
NleB1	139	150	IHKQESYPASER	NleB1 alone	100 s (23 C)	3.30	0.025127	5.48

NleB1	139	150	IHKQESYPASER	NleB1 alone	1,000 s (23 C)	4.20	0.066939	5.48
NleB1	139	150	IHKQESYPASER	NleB1 alone	10,000 s (23 C)	4.69	0.061626	5.49
NleB1	139	150	IHKQESYPASER	NleB1 alone	12 h (28 C)	4.66	0.035364	5.47
NleB1	158	170	LREELKNIPEGKD	NleB1 +YM155	10 s (23 C)	2.69	0.015361	4.14
NleB1	158	170	LREELKNIPEGKD	NleB1 +YM155	100 s (23 C)	4.08	0.037454	4.10
NleB1	158	170	LREELKNIPEGKD	NleB1 +YM155	1,000 s (23 C)	5.01	0.025369	4.11
NleB1	158	170	LREELKNIPEGKD	NleB1 +YM155	10,000 s (23 C)	5.77	0.051007	4.12
NleB1	158	170	LREELKNIPEGKD	NleB1 +YM155	12 h (28 C)	5.81	0.026213	4.11
NleB1	158	170	LREELKNIPEGKD	NleB1 alone	10 s (23 C)	2.54	0.051082	4.15
NleB1	158	170	LREELKNIPEGKD	NleB1 alone	100 s (23 C)	4.01	0.028973	4.11
NleB1	158	170	LREELKNIPEGKD	NleB1 alone	1,000 s (23 C)	4.97	0.010214	4.12
NleB1	158	170	LREELKNIPEGKD	NleB1 alone	10,000 s (23 C)	5.81	0.02482	4.13
NleB1	158	170	LREELKNIPEGKD	NleB1 alone	12 h (28 C)	5.87	0.011921	4.10
NleB1	158	172	LREELKNIPEGKDSL	NleB1 +YM155	10 s (23 C)	2.44	0.023376	4.94
NleB1	158	172	LREELKNIPEGKDSL	NleB1 +YM155	100 s (23 C)	3.88	0.04771	4.90
NleB1	158	172	LREELKNIPEGKDSL	NleB1 +YM155	1,000 s (23 C)	5.10	0.006615	4.91
NleB1	158	172	LREELKNIPEGKDSL	NleB1 +YM155	10,000 s (23 C)	6.22	0.049742	4.91
NleB1	158	172	LREELKNIPEGKDSL	NleB1 +YM155	12 h (28 C)	6.42	0.003328	4.91
NleB1	158	172	LREELKNIPEGKDSL	NleB1 alone	10 s (23 C)	2.25	0.028923	4.96
NleB1	158	172	LREELKNIPEGKDSL	NleB1 alone	100 s (23 C)	3.83	0.036457	4.93
NleB1	158	172	LREELKNIPEGKDSL	NleB1 alone	1,000 s (23 C)	5.09	0.0815	4.94
NleB1	158	172	LREELKNIPEGKDSL	NleB1 alone	10,000 s (23 C)	6.33	0.065191	4.94
NleB1	158	172	LREELKNIPEGKDSL	NleB1 alone	12 h (28 C)	6.51	0.01492	4.92
NleB1	158	175	LREELKNIPEGKDSLIES	NleB1 +YM155	10 s (23 C)	2.62	0.056229	5.19
NleB1	158	175	LREELKNIPEGKDSLIES	NleB1 +YM155	100 s (23 C)	4.48	0.058151	5.16
NleB1	158	175	LREELKNIPEGKDSLIES	NleB1 +YM155	1,000 s (23 C)	6.55	0.008327	5.17
NleB1	158	175	LREELKNIPEGKDSLIES	NleB1 +YM155	10,000 s (23 C)	8.82	0.004397	5.17
NleB1	158	175	LREELKNIPEGKDSLIES	NleB1 +YM155	12 h (28 C)	9.33	0.020019	5.16

NleB1	158	175	LREELKNIPEGKDSLIES	NleB1 alone	10 s (23 C)	2.48	0.025991	5.21
NleB1	158	175	LREELKNIPEGKDSLIES	NleB1 alone	100 s (23 C)	4.44	0.059491	5.18
NleB1	158	175	LREELKNIPEGKDSLIES	NleB1 alone	1,000 s (23 C)	6.26	0.014473	5.19
NleB1	158	175	LREELKNIPEGKDSLIES	NleB1 alone	10,000 s (23 C)	8.71	0.086869	5.20
NleB1	158	175	LREELKNIPEGKDSLIES	NleB1 alone	12 h (28 C)	9.34	0.057007	5.17
NleB1	158	176	LREELKNIPEGKDSLIESY	NleB1 +YM155	10 s (23 C)	2.40	0.026728	5.75
NleB1	158	176	LREELKNIPEGKDSLIESY	NleB1 +YM155	100 s (23 C)	4.17	0.01668	5.73
NleB1	158	176	LREELKNIPEGKDSLIESY	NleB1 +YM155	1,000 s (23 C)	6.46	0.131018	5.72
NleB1	158	176	LREELKNIPEGKDSLIESY	NleB1 +YM155	10,000 s (23 C)	8.95	0.05294	5.72
NleB1	158	176	LREELKNIPEGKDSLIESY	NleB1 +YM155	12 h (28 C)	9.79	0.028086	5.71
NleB1	158	176	LREELKNIPEGKDSLIESY	NleB1 alone	10 s (23 C)	2.41	0.023474	5.78
NleB1	158	176	LREELKNIPEGKDSLIESY	NleB1 alone	100 s (23 C)	4.29	0.09689	5.75
NleB1	158	176	LREELKNIPEGKDSLIESY	NleB1 alone	1,000 s (23 C)	6.18	0.076686	5.75
NleB1	158	176	LREELKNIPEGKDSLIESY	NleB1 alone	10,000 s (23 C)	8.85	0.126373	5.76
NleB1	158	176	LREELKNIPEGKDSLIESY	NleB1 alone	12 h (28 C)	9.63	0.249066	5.74
NleB1	158	187	LREELKNIPEGKDSLIESYAEKREHTWFDF	NleB1 +YM155	10 s (23 C)	3.34	0.029654	7.07
NleB1	158	187	LREELKNIPEGKDSLIESYAEKREHTWFDF	NleB1 +YM155	100 s (23 C)	6.01	0.112853	7.04
NleB1	158	187	LREELKNIPEGKDSLIESYAEKREHTWFDF	NleB1 +YM155	1,000 s (23 C)	9.42	0.049409	7.06
NleB1	158	187	LREELKNIPEGKDSLIESYAEKREHTWFDF	NleB1 +YM155	10,000 s (23 C)	12.70	0.077803	7.06
NleB1	158	187	LREELKNIPEGKDSLIESYAEKREHTWFDF	NleB1 +YM155	12 h (28 C)	13.63	0.155165	7.04
NleB1	158	187	LREELKNIPEGKDSLIESYAEKREHTWFDF	NleB1 alone	10 s (23 C)	3.60	0.130805	7.08
NleB1	158	187	LREELKNIPEGKDSLIESYAEKREHTWFDF	NleB1 alone	100 s (23 C)	7.02	0.051812	7.06
NleB1	158	187	LREELKNIPEGKDSLIESYAEKREHTWFDF	NleB1 alone	1,000 s (23 C)	10.31	0.031525	7.06
NleB1	158	187	LREELKNIPEGKDSLIESYAEKREHTWFDF	NleB1 alone	10,000 s (23 C)	14.10	0.141401	7.07
NleB1	158	187	LREELKNIPEGKDSLIESYAEKREHTWFDF	NleB1 alone	12 h (28 C)	15.19	0.213757	7.04
NleB1	159	172	REELKNIPEGKDSL	NleB1 +YM155	10 s (23 C)	2.50	0.04469	4.63
NleB1	159	172	REELKNIPEGKDSL	NleB1 +YM155	100 s (23 C)	3.97	0.013574	4.60
NleB1	159	172	REELKNIPEGKDSL	NleB1 +YM155	1,000 s (23 C)	5.01	0.028212	4.61

NleB1	159	172	REELKNIPEGKDSL	NleB1 +YM155	10,000 s (23 C)	5.92	0.043494	4.61
NleB1	159	172	REELKNIPEGKDSL	NleB1 +YM155	12 h (28 C)	6.01	0.003672	4.60
NleB1	159	172	REELKNIPEGKDSL	NleB1 alone	10 s (23 C)	2.33	0.042154	4.65
NleB1	159	172	REELKNIPEGKDSL	NleB1 alone	100 s (23 C)	3.90	0.021446	4.62
NleB1	159	172	REELKNIPEGKDSL	NleB1 alone	1,000 s (23 C)	4.96	0.059218	4.62
NleB1	159	172	REELKNIPEGKDSL	NleB1 alone	10,000 s (23 C)	5.98	0.023586	4.63
NleB1	159	172	REELKNIPEGKDSL	NleB1 alone	12 h (28 C)	6.05	0.048512	4.61
NleB1	159	187	REELKNIPEGKDSLIESYAEKREHTWFDF	NleB1 +YM155	10 s (23 C)	3.32	0.083976	7.05
NleB1	159	187	REELKNIPEGKDSLIESYAEKREHTWFDF	NleB1 +YM155	100 s (23 C)	5.89	0.135589	7.01
NleB1	159	187	REELKNIPEGKDSLIESYAEKREHTWFDF	NleB1 +YM155	1,000 s (23 C)	9.44	0.085895	7.02
NleB1	159	187	REELKNIPEGKDSLIESYAEKREHTWFDF	NleB1 +YM155	10,000 s (23 C)	12.21	0.233096	7.03
NleB1	159	187	REELKNIPEGKDSLIESYAEKREHTWFDF	NleB1 +YM155	12 h (28 C)	12.98	0.340066	7.01
NleB1	159	187	REELKNIPEGKDSLIESYAEKREHTWFDF	NleB1 alone	10 s (23 C)	3.67	0.19724	7.06
NleB1	159	187	REELKNIPEGKDSLIESYAEKREHTWFDF	NleB1 alone	100 s (23 C)	6.96	0.012323	7.03
NleB1	159	187	REELKNIPEGKDSLIESYAEKREHTWFDF	NleB1 alone	1,000 s (23 C)	9.81	0.051258	7.04
NleB1	159	187	REELKNIPEGKDSLIESYAEKREHTWFDF	NleB1 alone	10,000 s (23 C)	13.61	0.14222	7.04
NleB1	159	187	REELKNIPEGKDSLIESYAEKREHTWFDF	NleB1 alone	12 h (28 C)	14.45	0.316714	7.01
NleB1	163	172	KNIPEGKDSL	NleB1 +YM155	10 s (23 C)	2.35	0.03885	3.92
NleB1	163	172	KNIPEGKDSL	NleB1 +YM155	100 s (23 C)	3.25	0.025486	3.89
NleB1	163	172	KNIPEGKDSL	NleB1 +YM155	1,000 s (23 C)	3.80	0.01039	3.90
NleB1	163	172	KNIPEGKDSL	NleB1 +YM155	10,000 s (23 C)	4.25	0.028049	3.90
NleB1	163	172	KNIPEGKDSL	NleB1 +YM155	12 h (28 C)	4.31	0.013624	3.89
NleB1	163	172	KNIPEGKDSL	NleB1 alone	10 s (23 C)	2.25	0.008472	3.93
NleB1	163	172	KNIPEGKDSL	NleB1 alone	100 s (23 C)	3.26	0.030077	3.90
NleB1	163	172	KNIPEGKDSL	NleB1 alone	1,000 s (23 C)	3.81	0.014392	3.90
NleB1	163	172	KNIPEGKDSL	NleB1 alone	10,000 s (23 C)	4.33	0.019646	3.91
NleB1	163	172	KNIPEGKDSL	NleB1 alone	12 h (28 C)	4.38	0.035085	3.88
NleB1	163	175	KNIPEGKDSLIES	NleB1 +YM155	10 s (23 C)	2.42	0.063446	4.51

NleB1	163	175	KNIPEGKDSLIES	NleB1 +YM155	100 s (23 C)	3.56	0.053513	4.47
NleB1	163	175	KNIPEGKDSLIES	NleB1 +YM155	1,000 s (23 C)	4.65	0.034496	4.48
NleB1	163	175	KNIPEGKDSLIES	NleB1 +YM155	10,000 s (23 C)	6.22	0.018164	4.48
NleB1	163	175	KNIPEGKDSLIES	NleB1 +YM155	12 h (28 C)	6.63	0.083682	4.47
NleB1	163	175	KNIPEGKDSLIES	NleB1 alone	10 s (23 C)	2.32	0.006914	4.51
NleB1	163	175	KNIPEGKDSLIES	NleB1 alone	100 s (23 C)	3.51	0.069379	4.48
NleB1	163	175	KNIPEGKDSLIES	NleB1 alone	1,000 s (23 C)	4.41	0.032747	4.49
NleB1	163	175	KNIPEGKDSLIES	NleB1 alone	10,000 s (23 C)	6.16	0.011369	4.49
NleB1	163	175	KNIPEGKDSLIES	NleB1 alone	12 h (28 C)	6.67	0.102629	4.47
NleB1	163	187	KNIPEGKDSLIESYAEKREHTWFDF	NleB1 +YM155	10 s (23 C)	3.30	0.051971	7.20
NleB1	163	187	KNIPEGKDSLIESYAEKREHTWFDF	NleB1 +YM155	100 s (23 C)	6.05	0.12263	7.18
NleB1	163	187	KNIPEGKDSLIESYAEKREHTWFDF	NleB1 +YM155	1,000 s (23 C)	8.54	0.032398	7.19
NleB1	163	187	KNIPEGKDSLIESYAEKREHTWFDF	NleB1 +YM155	10,000 s (23 C)	11.02	0.144459	7.18
NleB1	163	187	KNIPEGKDSLIESYAEKREHTWFDF	NleB1 +YM155	12 h (28 C)	12.23	0.052835	7.17
NleB1	163	187	KNIPEGKDSLIESYAEKREHTWFDF	NleB1 alone	10 s (23 C)	3.33	0.052553	7.19
NleB1	163	187	KNIPEGKDSLIESYAEKREHTWFDF	NleB1 alone	100 s (23 C)	6.12	0.023027	7.17
NleB1	163	187	KNIPEGKDSLIESYAEKREHTWFDF	NleB1 alone	1,000 s (23 C)	8.32	0.077939	7.18
NleB1	163	187	KNIPEGKDSLIESYAEKREHTWFDF	NleB1 alone	10,000 s (23 C)	11.19	0.116712	7.19
NleB1	163	187	KNIPEGKDSLIESYAEKREHTWFDF	NleB1 alone	12 h (28 C)	12.12	0.045632	7.16
NleB1	166	172	PEGKDSL	NleB1 +YM155	10 s (23 C)	1.32	0.057622	4.63
NleB1	166	172	PEGKDSL	NleB1 +YM155	100 s (23 C)	2.19	0.03671	4.60
NleB1	166	172	PEGKDSL	NleB1 +YM155	1,000 s (23 C)	2.70	0.028695	4.61
NleB1	166	172	PEGKDSL	NleB1 +YM155	10,000 s (23 C)	3.04	0.03645	4.61
NleB1	166	172	PEGKDSL	NleB1 +YM155	12 h (28 C)	3.06	0.024066	4.59
NleB1	166	172	PEGKDSL	NleB1 alone	10 s (23 C)	1.23	0.030446	4.64
NleB1	166	172	PEGKDSL	NleB1 alone	100 s (23 C)	2.13	0.031446	4.62
NleB1	166	172	PEGKDSL	NleB1 alone	1,000 s (23 C)	2.61	0.028458	4.62
NleB1	166	172	PEGKDSL	NleB1 alone	10,000 s (23 C)	3.08	0.033959	4.63

NleB1	166	172	PEGKDSL	NleB1 alone	12 h (28 C)	3.10	0.011607	4.61
NleB1	167	172	EGKDSL	NleB1 +YM155	10 s (23 C)	0.90	0.011788	4.93
NleB1	167	172	EGKDSL	NleB1 +YM155	100 s (23 C)	1.28	0.039073	4.90
NleB1	167	172	EGKDSL	NleB1 +YM155	1,000 s (23 C)	1.72	0.019479	4.91
NleB1	167	172	EGKDSL	NleB1 +YM155	10,000 s (23 C)	1.95	0.033915	4.91
NleB1	167	172	EGKDSL	NleB1 +YM155	12 h (28 C)	1.93	0.013362	4.90
NleB1	167	172	EGKDSL	NleB1 alone	10 s (23 C)	0.81	0.014337	4.95
NleB1	167	172	EGKDSL	NleB1 alone	100 s (23 C)	1.28	0.031495	4.92
NleB1	167	172	EGKDSL	NleB1 alone	1,000 s (23 C)	1.72	0.049346	4.93
NleB1	167	172	EGKDSL	NleB1 alone	10,000 s (23 C)	1.99	0.036972	4.93
NleB1	167	172	EGKDSL	NleB1 alone	12 h (28 C)	1.97	0.014534	4.91
NleB1	171	187	SLIESYAEKREHTWFDF	NleB1 +YM155	10 s (23 C)	2.37	0.026097	7.82
NleB1	171	187	SLIESYAEKREHTWFDF	NleB1 +YM155	100 s (23 C)	4.27	0.076782	7.79
NleB1	171	187	SLIESYAEKREHTWFDF	NleB1 +YM155	1,000 s (23 C)	7.21	0.137223	7.80
NleB1	171	187	SLIESYAEKREHTWFDF	NleB1 +YM155	10,000 s (23 C)	9.65	0.074355	7.80
NleB1	171	187	SLIESYAEKREHTWFDF	NleB1 +YM155	12 h (28 C)	10.24	0.017689	7.79
NleB1	171	187	SLIESYAEKREHTWFDF	NleB1 alone	10 s (23 C)	2.40	0.094219	7.82
NleB1	171	187	SLIESYAEKREHTWFDF	NleB1 alone	100 s (23 C)	4.30	0.070455	7.80
NleB1	171	187	SLIESYAEKREHTWFDF	NleB1 alone	1,000 s (23 C)	6.73	0.015584	7.80
NleB1	171	187	SLIESYAEKREHTWFDF	NleB1 alone	10,000 s (23 C)	9.52	0.01348	7.80
NleB1	171	187	SLIESYAEKREHTWFDF	NleB1 alone	12 h (28 C)	10.12	0.1441	7.79
NleB1	173	185	IESYAEKREHTWF	NleB1 +YM155	10 s (23 C)	2.22	0.028844	6.18
NleB1	173	185	IESYAEKREHTWF	NleB1 +YM155	100 s (23 C)	3.70	0.054326	6.14
NleB1	173	185	IESYAEKREHTWF	NleB1 +YM155	1,000 s (23 C)	4.93	0.06662	6.15
NleB1	173	185	IESYAEKREHTWF	NleB1 +YM155	10,000 s (23 C)	5.63	0.065919	6.15
NleB1	173	185	IESYAEKREHTWF	NleB1 +YM155	12 h (28 C)	5.83	0.006307	6.15
NleB1	173	185	IESYAEKREHTWF	NleB1 alone	10 s (23 C)	2.04	0.022205	6.26
NleB1	173	185	IESYAEKREHTWF	NleB1 alone	100 s (23 C)	3.60	0.040232	6.23

NleB1	173	185	IESYAEKREHTWF	NleB1 alone	1,000 s (23 C)	4.81	0.055642	6.24
NleB1	173	185	IESYAEKREHTWF	NleB1 alone	10,000 s (23 C)	5.68	0.033957	6.25
NleB1	173	185	IESYAEKREHTWF	NleB1 alone	12 h (28 C)	5.84	0.145222	6.22
NleB1	173	187	IESYAEKREHTWFDF	NleB1 +YM155	10 s (23 C)	2.21	0.025559	7.51
NleB1	173	187	IESYAEKREHTWFDF	NleB1 +YM155	100 s (23 C)	3.87	0.048207	7.48
NleB1	173	187	IESYAEKREHTWFDF	NleB1 +YM155	1,000 s (23 C)	5.60	0.050502	7.49
NleB1	173	187	IESYAEKREHTWFDF	NleB1 +YM155	10,000 s (23 C)	6.68	0.038166	7.49
NleB1	173	187	IESYAEKREHTWFDF	NleB1 +YM155	12 h (28 C)	7.02	0.024208	7.48
NleB1	173	187	IESYAEKREHTWFDF	NleB1 alone	10 s (23 C)	2.01	0.017661	7.51
NleB1	173	187	IESYAEKREHTWFDF	NleB1 alone	100 s (23 C)	3.75	0.039564	7.48
NleB1	173	187	IESYAEKREHTWFDF	NleB1 alone	1,000 s (23 C)	5.43	0.03278	7.50
NleB1	173	187	IESYAEKREHTWFDF	NleB1 alone	10,000 s (23 C)	6.75	0.031679	7.50
NleB1	173	187	IESYAEKREHTWFDF	NleB1 alone	12 h (28 C)	6.98	0.124629	7.48
NleB1	174	187	ESYAEKREHTWFDF	NleB1 +YM155	10 s (23 C)	2.22	0.027996	7.51
NleB1	174	187	ESYAEKREHTWFDF	NleB1 +YM155	100 s (23 C)	3.67	0.03931	7.48
NleB1	174	187	ESYAEKREHTWFDF	NleB1 +YM155	1,000 s (23 C)	5.35	0.075492	7.49
NleB1	174	187	ESYAEKREHTWFDF	NleB1 +YM155	10,000 s (23 C)	6.37	0.027371	7.49
NleB1	174	187	ESYAEKREHTWFDF	NleB1 +YM155	12 h (28 C)	6.64	0.034103	7.48
NleB1	174	187	ESYAEKREHTWFDF	NleB1 alone	10 s (23 C)	2.01	0.049792	7.51
NleB1	174	187	ESYAEKREHTWFDF	NleB1 alone	100 s (23 C)	3.57	0.054825	7.49
NleB1	174	187	ESYAEKREHTWFDF	NleB1 alone	1,000 s (23 C)	5.16	0.048377	7.50
NleB1	174	187	ESYAEKREHTWFDF	NleB1 alone	10,000 s (23 C)	6.44	0.01371	7.50
NleB1	174	187	ESYAEKREHTWFDF	NleB1 alone	12 h (28 C)	6.51	0.052233	7.48
NleB1	175	187	SYAEKREHTWFDF	NleB1 +YM155	10 s (23 C)	1.80	0.041312	7.47
NleB1	175	187	SYAEKREHTWFDF	NleB1 +YM155	100 s (23 C)	3.18	0.030566	7.45
NleB1	175	187	SYAEKREHTWFDF	NleB1 +YM155	1,000 s (23 C)	4.58	0.043999	7.46
NleB1	175	187	SYAEKREHTWFDF	NleB1 +YM155	10,000 s (23 C)	5.33	0.048582	7.46
NleB1	175	187	SYAEKREHTWFDF	NleB1 +YM155	12 h (28 C)	5.54	0.050935	7.44

NleB1	175	187	SYAEKREHTWFDF	NleB1 alone	10 s (23 C)	1.59	0.017484	7.47
NleB1	175	187	SYAEKREHTWFDF	NleB1 alone	100 s (23 C)	3.06	0.056331	7.45
NleB1	175	187	SYAEKREHTWFDF	NleB1 alone	1,000 s (23 C)	4.47	0.025569	7.46
NleB1	175	187	SYAEKREHTWFDF	NleB1 alone	10,000 s (23 C)	5.39	0.011947	7.47
NleB1	175	187	SYAEKREHTWFDF	NleB1 alone	12 h (28 C)	5.58	0.105858	7.45
NleB1	176	187	YAEKREHTWFDF	NleB1 +YM155	10 s (23 C)	1.40	0.016318	7.44
NleB1	176	187	YAEKREHTWFDF	NleB1 +YM155	100 s (23 C)	2.36	0.04462	7.41
NleB1	176	187	YAEKREHTWFDF	NleB1 +YM155	1,000 s (23 C)	3.51	0.005891	7.42
NleB1	176	187	YAEKREHTWFDF	NleB1 +YM155	10,000 s (23 C)	3.97	0.020935	7.42
NleB1	176	187	YAEKREHTWFDF	NleB1 +YM155	12 h (28 C)	4.17	0.073268	7.42
NleB1	176	187	YAEKREHTWFDF	NleB1 alone	10 s (23 C)	1.17	0.016072	7.45
NleB1	176	187	YAEKREHTWFDF	NleB1 alone	100 s (23 C)	2.34	0.066054	7.41
NleB1	176	187	YAEKREHTWFDF	NleB1 alone	1,000 s (23 C)	3.50	0.018569	7.43
NleB1	176	187	YAEKREHTWFDF	NleB1 alone	10,000 s (23 C)	4.04	0.038463	7.43
NleB1	176	187	YAEKREHTWFDF	NleB1 alone	12 h (28 C)	4.19	0.061666	7.41
NleB1	187	194	FFRNLAIL	NleB1 +YM155	10 s (23 C)	0.49	0.019702	6.67
NleB1	187	194	FFRNLAIL	NleB1 +YM155	100 s (23 C)	1.70	0.05162	6.63
NleB1	187	194	FFRNLAIL	NleB1 +YM155	1,000 s (23 C)	2.58	0.02866	6.65
NleB1	187	194	FFRNLAIL	NleB1 +YM155	10,000 s (23 C)	3.10	0.037011	6.64
NleB1	187	194	FFRNLAIL	NleB1 +YM155	12 h (28 C)	3.82	0.032761	6.64
NleB1	187	194	FFRNLAIL	NleB1 alone	10 s (23 C)	0.40	0.016001	6.77
NleB1	187	194	FFRNLAIL	NleB1 alone	100 s (23 C)	1.71	0.038103	6.74
NleB1	187	194	FFRNLAIL	NleB1 alone	1,000 s (23 C)	2.86	0.028766	6.75
NleB1	187	194	FFRNLAIL	NleB1 alone	10,000 s (23 C)	3.51	0.019164	6.75
NleB1	187	194	FFRNLAIL	NleB1 alone	12 h (28 C)	4.38	0.027957	6.74
NleB1	190	202	NLAILKAGSLFTE	NleB1 +YM155	10 s (23 C)	0.58	0.031233	6.31
NleB1	190	202	NLAILKAGSLFTE	NleB1 +YM155	100 s (23 C)	0.80	0.02501	6.29
NleB1	190	202	NLAILKAGSLFTE	NleB1 +YM155	1,000 s (23 C)	1.04	0.007125	6.28

NleB1	190	202	NLAILKAGSLFTE	NleB1 +YM155	10,000 s (23 C)	1.05	0.045232	6.29
NleB1	190	202	NLAILKAGSLFTE	NleB1 +YM155	12 h (28 C)	3.11	0.052942	6.29
NleB1	190	202	NLAILKAGSLFTE	NleB1 alone	10 s (23 C)	0.52	0.027131	6.38
NleB1	190	202	NLAILKAGSLFTE	NleB1 alone	100 s (23 C)	0.79	0.011227	6.35
NleB1	190	202	NLAILKAGSLFTE	NleB1 alone	1,000 s (23 C)	1.02	0.024703	6.36
NleB1	190	202	NLAILKAGSLFTE	NleB1 alone	10,000 s (23 C)	1.07	0.024543	6.37
NleB1	190	202	NLAILKAGSLFTE	NleB1 alone	12 h (28 C)	3.22	0.005482	6.35
NleB1	194	199	LKAGSL	NleB1 +YM155	10 s (23 C)	0.33	0.039958	4.49
NleB1	194	199	LKAGSL	NleB1 +YM155	100 s (23 C)	0.40	0.026545	4.46
NleB1	194	199	LKAGSL	NleB1 +YM155	1,000 s (23 C)	0.83	0.028385	4.47
NleB1	194	199	LKAGSL	NleB1 +YM155	10,000 s (23 C)	1.16	0.026454	4.47
NleB1	194	199	LKAGSL	NleB1 +YM155	12 h (28 C)	1.98	0.069099	4.46
NleB1	194	199	LKAGSL	NleB1 alone	10 s (23 C)	0.22	0.007034	4.50
NleB1	194	199	LKAGSL	NleB1 alone	100 s (23 C)	0.32	0.010835	4.47
NleB1	194	199	LKAGSL	NleB1 alone	1,000 s (23 C)	0.64	0.011237	4.47
NleB1	194	199	LKAGSL	NleB1 alone	10,000 s (23 C)	0.98	0.014685	4.48
NleB1	194	199	LKAGSL	NleB1 alone	12 h (28 C)	2.00	0.043004	4.45
NleB1	195	199	KAGSL	NleB1 +YM155	10 s (23 C)	0.32	0.107658	3.26
NleB1	195	199	KAGSL	NleB1 +YM155	100 s (23 C)	0.43	0.025259	3.21
NleB1	195	199	KAGSL	NleB1 +YM155	1,000 s (23 C)	0.71	0.012905	3.24
NleB1	195	199	KAGSL	NleB1 +YM155	10,000 s (23 C)	0.99	0.025008	3.23
NleB1	195	199	KAGSL	NleB1 +YM155	12 h (28 C)	1.51	0.017401	3.22
NleB1	195	199	KAGSL	NleB1 alone	10 s (23 C)	0.17	0.005293	3.28
NleB1	195	199	KAGSL	NleB1 alone	100 s (23 C)	0.28	0.004586	3.23
NleB1	195	199	KAGSL	NleB1 alone	1,000 s (23 C)	0.54	0.038055	3.24
NleB1	195	199	KAGSL	NleB1 alone	10,000 s (23 C)	0.87	0.023298	3.24
NleB1	195	199	KAGSL	NleB1 alone	12 h (28 C)	1.48	0.017864	3.26
NleB1	200	216	FTETGKTGCHNISPCSG	NleB1 +YM155	10 s (23 C)	5.90	0.173701	4.23

NleB1	200	216	FTETGKTGCHNISPCSG	NleB1 +YM155	100 s (23 C)	7.02	0.090954	4.19
NleB1	200	216	FTETGKTGCHNISPCSG	NleB1 +YM155	1,000 s (23 C)	7.34	0.020311	4.20
NleB1	200	216	FTETGKTGCHNISPCSG	NleB1 +YM155	10,000 s (23 C)	7.25	0.050524	4.21
NleB1	200	216	FTETGKTGCHNISPCSG	NleB1 +YM155	12 h (28 C)	7.57	0.074254	4.19
NleB1	200	216	FTETGKTGCHNISPCSG	NleB1 alone	10 s (23 C)	5.97	0.040223	4.23
NleB1	200	216	FTETGKTGCHNISPCSG	NleB1 alone	100 s (23 C)	7.19	0.029697	4.20
NleB1	200	216	FTETGKTGCHNISPCSG	NleB1 alone	1,000 s (23 C)	7.52	0.018823	4.21
NleB1	200	216	FTETGKTGCHNISPCSG	NleB1 alone	10,000 s (23 C)	7.36	0.062096	4.22
NleB1	200	216	FTETGKTGCHNISPCSG	NleB1 alone	12 h (28 C)	7.57	0.136841	4.19
NleB1	200	217	FTETGKTGCHNISPCSGC	NleB1 +YM155	10 s (23 C)	5.66	0.153578	4.66
NleB1	200	217	FTETGKTGCHNISPCSGC	NleB1 +YM155	100 s (23 C)	6.65	0.071782	4.62
NleB1	200	217	FTETGKTGCHNISPCSGC	NleB1 +YM155	1,000 s (23 C)	6.82	0.028294	4.63
NleB1	200	217	FTETGKTGCHNISPCSGC	NleB1 +YM155	10,000 s (23 C)	6.83	0.054004	4.63
NleB1	200	217	FTETGKTGCHNISPCSGC	NleB1 +YM155	12 h (28 C)	7.36	0.056129	4.63
NleB1	200	217	FTETGKTGCHNISPCSGC	NleB1 alone	10 s (23 C)	5.72	0.01138	4.67
NleB1	200	217	FTETGKTGCHNISPCSGC	NleB1 alone	100 s (23 C)	6.90	0.064576	4.64
NleB1	200	217	FTETGKTGCHNISPCSGC	NleB1 alone	1,000 s (23 C)	7.08	0.017761	4.65
NleB1	200	217	FTETGKTGCHNISPCSGC	NleB1 alone	10,000 s (23 C)	6.99	0.032347	4.65
NleB1	200	217	FTETGKTGCHNISPCSGC	NleB1 alone	12 h (28 C)	7.47	0.102142	4.63
NleB1	200	219	FTETGKTGCHNISPCSGCIY	NleB1 +YM155	10 s (23 C)	5.61	0.181226	5.91
NleB1	200	219	FTETGKTGCHNISPCSGCIY	NleB1 +YM155	100 s (23 C)	6.52	0.054525	5.88
NleB1	200	219	FTETGKTGCHNISPCSGCIY	NleB1 +YM155	1,000 s (23 C)	6.95	0.070325	5.88
NleB1	200	219	FTETGKTGCHNISPCSGCIY	NleB1 +YM155	10,000 s (23 C)	6.94	0.09121	5.88
NleB1	200	219	FTETGKTGCHNISPCSGCIY	NleB1 +YM155	12 h (28 C)	7.79	0.003979	5.88
NleB1	200	219	FTETGKTGCHNISPCSGCIY	NleB1 alone	10 s (23 C)	5.52	0.05711	5.95
NleB1	200	219	FTETGKTGCHNISPCSGCIY	NleB1 alone	100 s (23 C)	6.72	0.037801	5.92
NleB1	200	219	FTETGKTGCHNISPCSGCIY	NleB1 alone	1,000 s (23 C)	7.00	0.014044	5.93
NleB1	200	219	FTETGKTGCHNISPCSGCIY	NleB1 alone	10,000 s (23 C)	6.97	0.045444	5.94

NleB1	200	219	FTETGKTGCHNISPCSGCIY	NleB1 alone	12 h (28 C)	7.93	0.131386	5.92
NleB1	202	217	ETGKTGCHNISPCSGC	NleB1 +YM155	10 s (23 C)	5.48	0.134986	4.66
NleB1	202	217	ETGKTGCHNISPCSGC	NleB1 +YM155	100 s (23 C)	6.36	0.120315	4.62
NleB1	202	217	ETGKTGCHNISPCSGC	NleB1 +YM155	1,000 s (23 C)	6.44	0.052359	4.63
NleB1	202	217	ETGKTGCHNISPCSGC	NleB1 +YM155	10,000 s (23 C)	6.34	0.07829	4.63
NleB1	202	217	ETGKTGCHNISPCSGC	NleB1 +YM155	12 h (28 C)	6.66	0.029279	4.62
NleB1	202	217	ETGKTGCHNISPCSGC	NleB1 alone	10 s (23 C)	5.46	0.089232	4.67
NleB1	202	217	ETGKTGCHNISPCSGC	NleB1 alone	100 s (23 C)	6.47	0.048491	4.64
NleB1	202	217	ETGKTGCHNISPCSGC	NleB1 alone	1,000 s (23 C)	6.57	0.078237	4.65
NleB1	202	217	ETGKTGCHNISPCSGC	NleB1 alone	10,000 s (23 C)	6.59	0.028489	4.65
NleB1	202	217	ETGKTGCHNISPCSGC	NleB1 alone	12 h (28 C)	6.85	0.142507	4.63
NleB1	218	222	IYLDA	NleB1 +YM155	10 s (23 C)	0.16	0.009316	6.02
NleB1	218	222	IYLDA	NleB1 +YM155	100 s (23 C)	0.27	0.027146	5.98
NleB1	218	222	IYLDA	NleB1 +YM155	1,000 s (23 C)	0.55	0.004589	5.99
NleB1	218	222	IYLDA	NleB1 +YM155	10,000 s (23 C)	0.69	0.033791	5.99
NleB1	218	222	IYLDA	NleB1 +YM155	12 h (28 C)	0.90	0.039458	5.99
NleB1	218	222	IYLDA	NleB1 alone	10 s (23 C)	0.18	0.027458	6.07
NleB1	218	222	IYLDA	NleB1 alone	100 s (23 C)	0.28	0.011127	6.03
NleB1	218	222	IYLDA	NleB1 alone	1,000 s (23 C)	0.56	0.006213	6.05
NleB1	218	222	IYLDA	NleB1 alone	10,000 s (23 C)	0.67	0.035578	6.05
NleB1	218	222	IYLDA	NleB1 alone	12 h (28 C)	1.07	0.018455	6.03
NleB1	218	223	IYLDAD	NleB1 +YM155	10 s (23 C)	0.17	0.024064	5.66
NleB1	218	223	IYLDAD	NleB1 +YM155	100 s (23 C)	0.24	0.033606	5.61
NleB1	218	223	IYLDAD	NleB1 +YM155	1,000 s (23 C)	0.83	0.011386	5.63
NleB1	218	223	IYLDAD	NleB1 +YM155	10,000 s (23 C)	1.32	0.025261	5.63
NleB1	218	223	IYLDAD	NleB1 +YM155	12 h (28 C)	1.58	0.004051	5.62
NleB1	218	223	IYLDAD	NleB1 alone	10 s (23 C)	0.16	0.033274	5.68
NleB1	218	223	IYLDAD	NleB1 alone	100 s (23 C)	0.28	0.035298	5.65

NleB1	218	223	IYLDAD	NleB1 alone	1,000 s (23 C)	0.86	0.012144	5.66
NleB1	218	223	IYLDAD	NleB1 alone	10,000 s (23 C)	1.37	0.027544	5.66
NleB1	218	223	IYLDAD	NleB1 alone	12 h (28 C)	1.75	0.003909	5.64
NleB1	218	224	IYLDADM	NleB1 +YM155	10 s (23 C)	0.16	0.021525	6.99
NleB1	218	224	IYLDADM	NleB1 +YM155	100 s (23 C)	0.25	0.037983	6.95
NleB1	218	224	IYLDADM	NleB1 +YM155	1,000 s (23 C)	0.84	0.001001	6.97
NleB1	218	224	IYLDADM	NleB1 +YM155	10,000 s (23 C)	1.40	0.020852	6.97
NleB1	218	224	IYLDADM	NleB1 +YM155	12 h (28 C)	2.01	0.057655	6.96
NleB1	218	224	IYLDADM	NleB1 alone	10 s (23 C)	0.19	0.018073	7.00
NleB1	218	224	IYLDADM	NleB1 alone	100 s (23 C)	0.34	0.010187	6.98
NleB1	218	224	IYLDADM	NleB1 alone	1,000 s (23 C)	0.86	0.004539	6.99
NleB1	218	224	IYLDADM	NleB1 alone	10,000 s (23 C)	1.41	0.025774	7.00
NleB1	218	224	IYLDADM	NleB1 alone	12 h (28 C)	2.22	0.034392	6.98
NleB1	223	230	DMIITDKL	NleB1 +YM155	10 s (23 C)	0.46	0.017742	6.55
NleB1	223	230	DMIITDKL	NleB1 +YM155	100 s (23 C)	1.10	0.056064	6.51
NleB1	223	230	DMIITDKL	NleB1 +YM155	1,000 s (23 C)	2.02	0.061498	6.50
NleB1	223	230	DMIITDKL	NleB1 +YM155	10,000 s (23 C)	3.12	0.020158	6.50
NleB1	223	230	DMIITDKL	NleB1 +YM155	12 h (28 C)	3.96	0.010276	6.50
NleB1	223	230	DMIITDKL	NleB1 alone	10 s (23 C)	0.37	0.011313	6.61
NleB1	223	230	DMIITDKL	NleB1 alone	100 s (23 C)	0.98	0.013956	6.59
NleB1	223	230	DMIITDKL	NleB1 alone	1,000 s (23 C)	1.89	0.024745	6.60
NleB1	223	230	DMIITDKL	NleB1 alone	10,000 s (23 C)	2.96	0.03296	6.60
NleB1	223	230	DMIITDKL	NleB1 alone	12 h (28 C)	3.99	0.040873	6.58
NleB1	223	233	DMIITDKLGVL	NleB1 +YM155	10 s (23 C)	0.61	0.002517	7.64
NleB1	223	233	DMIITDKLGVL	NleB1 +YM155	100 s (23 C)	1.67	0.05417	7.61
NleB1	223	233	DMIITDKLGVL	NleB1 +YM155	1,000 s (23 C)	3.27	0.055361	7.62
NleB1	223	233	DMIITDKLGVL	NleB1 +YM155	10,000 s (23 C)	4.51	0.050624	7.62
NleB1	223	233	DMIITDKLGVL	NleB1 +YM155	12 h (28 C)	5.73	0.00114	7.61

NleB1	223	233	DMIITDKLGVL	NleB1 alone	10 s (23 C)	0.48	0.020668	7.64
NleB1	223	233	DMIITDKLGVL	NleB1 alone	100 s (23 C)	1.57	0.08713	7.62
NleB1	223	233	DMIITDKLGVL	NleB1 alone	1,000 s (23 C)	3.04	0.013591	7.63
NleB1	223	233	DMIITDKLGVL	NleB1 alone	10,000 s (23 C)	4.33	0.017138	7.63
NleB1	223	233	DMIITDKLGVL	NleB1 alone	12 h (28 C)	5.68	0.064454	7.61
NleB1	224	230	MIITDKL	NleB1 +YM155	10 s (23 C)	0.51	0.045124	6.19
NleB1	224	230	MIITDKL	NleB1 +YM155	100 s (23 C)	1.19	0.068599	6.16
NleB1	224	230	MIITDKL	NleB1 +YM155	1,000 s (23 C)	2.02	0.037337	6.17
NleB1	224	230	MIITDKL	NleB1 +YM155	10,000 s (23 C)	2.70	0.034439	6.17
NleB1	224	230	MIITDKL	NleB1 +YM155	12 h (28 C)	3.40	0.002961	6.16
NleB1	224	230	MIITDKL	NleB1 alone	10 s (23 C)	0.31	0.003671	6.24
NleB1	224	230	MIITDKL	NleB1 alone	100 s (23 C)	1.10	0.032263	6.21
NleB1	224	230	MIITDKL	NleB1 alone	1,000 s (23 C)	1.89	0.010681	6.22
NleB1	224	230	MIITDKL	NleB1 alone	10,000 s (23 C)	2.66	0.026773	6.23
NleB1	224	230	MIITDKL	NleB1 alone	12 h (28 C)	3.45	0.006444	6.21
NleB1	225	230	IITDKL	NleB1 +YM155	10 s (23 C)	0.46	0.018376	5.27
NleB1	225	230	IITDKL	NleB1 +YM155	100 s (23 C)	0.92	0.040226	5.23
NleB1	225	230	IITDKL	NleB1 +YM155	1,000 s (23 C)	1.42	0.019893	5.24
NleB1	225	230	IITDKL	NleB1 +YM155	10,000 s (23 C)	1.66	0.024505	5.24
NleB1	225	230	IITDKL	NleB1 +YM155	12 h (28 C)	2.02	0.041495	5.24
NleB1	225	230	IITDKL	NleB1 alone	10 s (23 C)	0.33	0.006073	5.28
NleB1	225	230	IITDKL	NleB1 alone	100 s (23 C)	0.85	0.021763	5.25
NleB1	225	230	IITDKL	NleB1 alone	1,000 s (23 C)	1.37	0.025621	5.26
NleB1	225	230	IITDKL	NleB1 alone	10,000 s (23 C)	1.69	0.026902	5.27
NleB1	225	230	IITDKL	NleB1 alone	12 h (28 C)	1.87	0.021705	5.25
NleB1	225	232	IITDKLGV	NleB1 +YM155	10 s (23 C)	0.29	0.021026	7.64
NleB1	225	232	IITDKLGV	NleB1 +YM155	100 s (23 C)	1.01	0.047138	7.61
NleB1	225	232	IITDKLGV	NleB1 +YM155	1,000 s (23 C)	2.04	0.026847	7.62

NleB1	225	232	IITDKLGV	NleB1 +YM155	10,000 s (23 C)	2.95	0.039175	7.62
NleB1	225	232	IITDKLGV	NleB1 +YM155	12 h (28 C)	3.60	0.025341	7.61
NleB1	225	232	IITDKLGV	NleB1 alone	10 s (23 C)	0.27	0.04745	7.64
NleB1	225	232	IITDKLGV	NleB1 alone	100 s (23 C)	0.98	0.062657	7.61
NleB1	225	232	IITDKLGV	NleB1 alone	1,000 s (23 C)	2.01	0.045017	7.63
NleB1	225	232	IITDKLGV	NleB1 alone	10,000 s (23 C)	2.89	0.04038	7.63
NleB1	225	232	IITDKLGV	NleB1 alone	12 h (28 C)	3.58	0.024807	7.61
NleB1	225	233	IITDKLGVL	NleB1 +YM155	10 s (23 C)	0.57	0.023074	6.67
NleB1	225	233	IITDKLGVL	NleB1 +YM155	100 s (23 C)	1.91	0.05294	6.63
NleB1	225	233	IITDKLGVL	NleB1 +YM155	1,000 s (23 C)	3.32	0.03486	6.65
NleB1	225	233	IITDKLGVL	NleB1 +YM155	10,000 s (23 C)	4.02	0.04617	6.65
NleB1	225	233	IITDKLGVL	NleB1 +YM155	12 h (28 C)	4.79	0.01594	6.64
NleB1	225	233	IITDKLGVL	NleB1 alone	10 s (23 C)	0.48	0.023955	6.77
NleB1	225	233	IITDKLGVL	NleB1 alone	100 s (23 C)	1.80	0.020115	6.74
NleB1	225	233	IITDKLGVL	NleB1 alone	1,000 s (23 C)	3.25	0.013744	6.75
NleB1	225	233	IITDKLGVL	NleB1 alone	10,000 s (23 C)	4.03	0.015697	6.75
NleB1	225	233	IITDKLGVL	NleB1 alone	12 h (28 C)	4.82	0.050111	6.74
NleB1	225	240	IITDKLGVLYAPDGIA	NleB1 +YM155	10 s (23 C)	0.61	0.02502	7.21
NleB1	225	240	IITDKLGVLYAPDGIA	NleB1 +YM155	100 s (23 C)	2.12	0.032118	7.17
NleB1	225	240	IITDKLGVLYAPDGIA	NleB1 +YM155	1,000 s (23 C)	4.62	0.059351	7.19
NleB1	225	240	IITDKLGVLYAPDGIA	NleB1 +YM155	10,000 s (23 C)	7.02	0.058943	7.19
NleB1	225	240	IITDKLGVLYAPDGIA	NleB1 +YM155	12 h (28 C)	9.16	0.050271	7.18
NleB1	225	240	IITDKLGVLYAPDGIA	NleB1 alone	10 s (23 C)	0.51	0.012829	7.21
NleB1	225	240	IITDKLGVLYAPDGIA	NleB1 alone	100 s (23 C)	2.13	0.05482	7.18
NleB1	225	240	IITDKLGVLYAPDGIA	NleB1 alone	1,000 s (23 C)	4.38	0.018542	7.19
NleB1	225	240	IITDKLGVLYAPDGIA	NleB1 alone	10,000 s (23 C)	6.86	0.028493	7.20
NleB1	225	240	IITDKLGVLYAPDGIA	NleB1 alone	12 h (28 C)	9.10	0.085593	7.17
NleB1	225	241	IITDKLGVLYAPDGIAV	NleB1 +YM155	10 s (23 C)	0.69	0.04923	7.61

NleB1	225	241	IITDKLGVLYAPDGIAV	NleB1 +YM155	100 s (23 C)	2.38	0.078928	7.58
NleB1	225	241	IITDKLGVLYAPDGIAV	NleB1 +YM155	1,000 s (23 C)	5.26	0.068498	7.60
NleB1	225	241	IITDKLGVLYAPDGIAV	NleB1 +YM155	10,000 s (23 C)	8.10	0.052253	7.59
NleB1	225	241	IITDKLGVLYAPDGIAV	NleB1 +YM155	12 h (28 C)	10.23	0.034301	7.59
NleB1	225	241	IITDKLGVLYAPDGIAV	NleB1 alone	10 s (23 C)	0.58	0.021543	7.61
NleB1	225	241	IITDKLGVLYAPDGIAV	NleB1 alone	100 s (23 C)	2.35	0.016694	7.59
NleB1	225	241	IITDKLGVLYAPDGIAV	NleB1 alone	1,000 s (23 C)	5.09	0.025514	7.60
NleB1	225	241	IITDKLGVLYAPDGIAV	NleB1 alone	10,000 s (23 C)	7.94	0.042158	7.60
NleB1	225	241	IITDKLGVLYAPDGIAV	NleB1 alone	12 h (28 C)	10.25	0.085045	7.58
NleB1	225	247	IITDKLGVLYAPDGIAVHVDCND	NleB1 +YM155	10 s (23 C)	2.44	0.027985	6.90
NleB1	225	247	IITDKLGVLYAPDGIAVHVDCND	NleB1 +YM155	100 s (23 C)	4.66	0.048139	6.86
NleB1	225	247	IITDKLGVLYAPDGIAVHVDCND	NleB1 +YM155	1,000 s (23 C)	8.25	0.082486	6.87
NleB1	225	247	IITDKLGVLYAPDGIAVHVDCND	NleB1 +YM155	10,000 s (23 C)	11.76	0.134222	6.88
NleB1	225	247	IITDKLGVLYAPDGIAVHVDCND	NleB1 alone	10 s (23 C)	2.32	0.045418	6.91
NleB1	225	247	IITDKLGVLYAPDGIAVHVDCND	NleB1 alone	100 s (23 C)	4.89	0.102211	6.88
NleB1	225	247	IITDKLGVLYAPDGIAVHVDCND	NleB1 alone	1,000 s (23 C)	8.38	0.084893	6.89
NleB1	225	247	IITDKLGVLYAPDGIAVHVDCND	NleB1 alone	10,000 s (23 C)	11.65	0.090499	6.89
NleB1	225	247	IITDKLGVLYAPDGIAVHVDCND	NleB1 alone	12 h (28 C)	13.94	0.052826	6.87
NleB1	226	233	ITDKLGVL	NleB1 +YM155	10 s (23 C)	0.41	0.013052	7.36
NleB1	226	233	ITDKLGVL	NleB1 +YM155	100 s (23 C)	1.24	0.040321	7.33
NleB1	226	233	ITDKLGVL	NleB1 +YM155	1,000 s (23 C)	2.38	0.058189	7.34
NleB1	226	233	ITDKLGVL	NleB1 +YM155	10,000 s (23 C)	2.98	0.040217	7.35
NleB1	226	233	ITDKLGVL	NleB1 +YM155	12 h (28 C)	3.64	3.63E-05	7.34
NleB1	226	233	ITDKLGVL	NleB1 alone	10 s (23 C)	0.31	0.004619	7.37
NleB1	226	233	ITDKLGVL	NleB1 alone	100 s (23 C)	1.16	0.041621	7.34
NleB1	226	233	ITDKLGVL	NleB1 alone	1,000 s (23 C)	2.27	0.012422	7.35
NleB1	226	233	ITDKLGVL	NleB1 alone	10,000 s (23 C)	2.96	0.017393	7.35
NleB1	226	233	ITDKLGVL	NleB1 alone	12 h (28 C)	3.65	0.035633	7.33

NleB1	228	233	DKLGVL	NleB1 +YM155	10 s (23 C)	0.40	0.025521	6.67
NleB1	228	233	DKLGVL	NleB1 +YM155	100 s (23 C)	1.35	0.011566	6.63
NleB1	228	233	DKLGVL	NleB1 +YM155	1,000 s (23 C)	2.44	0.061806	6.65
NleB1	228	233	DKLGVL	NleB1 +YM155	10,000 s (23 C)	3.00	0.031984	6.65
NleB1	228	233	DKLGVL	NleB1 +YM155	12 h (28 C)	3.60	0.022809	6.64
NleB1	228	233	DKLGVL	NleB1 alone	10 s (23 C)	0.30	0.006099	6.77
NleB1	228	233	DKLGVL	NleB1 alone	100 s (23 C)	1.29	0.016618	6.74
NleB1	228	233	DKLGVL	NleB1 alone	1,000 s (23 C)	2.41	0.036457	6.75
NleB1	228	233	DKLGVL	NleB1 alone	10,000 s (23 C)	3.06	0.008616	6.75
NleB1	228	233	DKLGVL	NleB1 alone	12 h (28 C)	3.72	0.017373	6.73
NleB1	231	241	GVLYAPDGIAV	NleB1 +YM155	10 s (23 C)	0.17	0.017507	7.27
NleB1	231	241	GVLYAPDGIAV	NleB1 +YM155	100 s (23 C)	0.72	0.045565	7.24
NleB1	231	241	GVLYAPDGIAV	NleB1 +YM155	1,000 s (23 C)	2.19	0.049706	7.25
NleB1	231	241	GVLYAPDGIAV	NleB1 +YM155	10,000 s (23 C)	4.41	0.027883	7.25
NleB1	231	241	GVLYAPDGIAV	NleB1 +YM155	12 h (28 C)	6.12	0.067673	7.24
NleB1	231	241	GVLYAPDGIAV	NleB1 alone	10 s (23 C)	0.15	0.007014	7.27
NleB1	231	241	GVLYAPDGIAV	NleB1 alone	100 s (23 C)	0.72	0.014633	7.24
NleB1	231	241	GVLYAPDGIAV	NleB1 alone	1,000 s (23 C)	2.08	0.019203	7.26
NleB1	231	241	GVLYAPDGIAV	NleB1 alone	10,000 s (23 C)	4.19	0.035645	7.26
NleB1	231	241	GVLYAPDGIAV	NleB1 alone	12 h (28 C)	6.08	0.053177	7.24
NleB1	234	241	YAPDGIAV	NleB1 +YM155	10 s (23 C)	0.34	0.012287	6.07
NleB1	234	241	YAPDGIAV	NleB1 +YM155	100 s (23 C)	0.58	0.072466	6.03
NleB1	234	241	YAPDGIAV	NleB1 +YM155	1,000 s (23 C)	1.20	0.043997	6.05
NleB1	234	241	YAPDGIAV	NleB1 +YM155	10,000 s (23 C)	2.48	0.012677	6.05
NleB1	234	241	YAPDGIAV	NleB1 +YM155	12 h (28 C)	3.50	0.003128	6.04
NleB1	234	241	YAPDGIAV	NleB1 alone	10 s (23 C)	0.25	0.0117	6.12
NleB1	234	241	YAPDGIAV	NleB1 alone	100 s (23 C)	0.43	0.021725	6.09
NleB1	234	241	YAPDGIAV	NleB1 alone	1,000 s (23 C)	1.01	0.009919	6.10

NleB1	234	241	YAPDGIAV	NleB1 alone	10,000 s (23 C)	2.48	0.001522	6.11
NleB1	234	241	YAPDGIAV	NleB1 alone	12 h (28 C)	3.53	0.073858	6.08
NleB1	234	245	YAPDGIAVHVDC	NleB1 +YM155	10 s (23 C)	1.02	0.026022	5.93
NleB1	234	245	YAPDGIAVHVDC	NleB1 +YM155	100 s (23 C)	1.83	0.052843	5.90
NleB1	234	245	YAPDGIAVHVDC	NleB1 +YM155	1,000 s (23 C)	3.29	0.029036	5.90
NleB1	234	245	YAPDGIAVHVDC	NleB1 +YM155	10,000 s (23 C)	4.98	0.032257	5.90
NleB1	234	245	YAPDGIAVHVDC	NleB1 +YM155	12 h (28 C)	5.97	ND	5.90
NleB1	234	245	YAPDGIAVHVDC	NleB1 alone	10 s (23 C)	0.95	0.027303	5.97
NleB1	234	245	YAPDGIAVHVDC	NleB1 alone	100 s (23 C)	1.81	0.005013	5.94
NleB1	234	245	YAPDGIAVHVDC	NleB1 alone	1,000 s (23 C)	3.20	0.01226	5.95
NleB1	234	245	YAPDGIAVHVDC	NleB1 alone	10,000 s (23 C)	4.96	0.046887	5.95
NleB1	234	245	YAPDGIAVHVDC	NleB1 alone	12 h (28 C)	6.15	0.119153	5.93
NleB1	234	247	YAPDGIAVHVDCND	NleB1 +YM155	10 s (23 C)	2.12	0.068565	5.58
NleB1	234	247	YAPDGIAVHVDCND	NleB1 +YM155	100 s (23 C)	2.97	0.007583	5.54
NleB1	234	247	YAPDGIAVHVDCND	NleB1 +YM155	1,000 s (23 C)	4.28	0.020341	5.56
NleB1	234	247	YAPDGIAVHVDCND	NleB1 +YM155	10,000 s (23 C)	5.79	0.004626	5.56
NleB1	234	247	YAPDGIAVHVDCND	NleB1 +YM155	12 h (28 C)	6.70	0.062022	5.54
NleB1	234	247	YAPDGIAVHVDCND	NleB1 alone	10 s (23 C)	2.08	0.009419	5.61
NleB1	234	247	YAPDGIAVHVDCND	NleB1 alone	100 s (23 C)	2.96	0.047079	5.58
NleB1	234	247	YAPDGIAVHVDCND	NleB1 alone	1,000 s (23 C)	4.19	0.040573	5.59
NleB1	234	247	YAPDGIAVHVDCND	NleB1 alone	10,000 s (23 C)	5.84	0.0445	5.59
NleB1	234	247	YAPDGIAVHVDCND	NleB1 alone	12 h (28 C)	6.79	0.056062	5.57
NleB1	234	256	YAPDGIAVHVDCNDEIKSLENGA	NleB1 +YM155	10 s (23 C)	7.05	0.092511	6.61
NleB1	234	256	YAPDGIAVHVDCNDEIKSLENGA	NleB1 +YM155	100 s (23 C)	9.01	0.109816	6.58
NleB1	234	256	YAPDGIAVHVDCNDEIKSLENGA	NleB1 +YM155	1,000 s (23 C)	10.36	0.028664	6.57
NleB1	234	256	YAPDGIAVHVDCNDEIKSLENGA	NleB1 +YM155	10,000 s (23 C)	11.77	0.05603	6.58
NleB1	234	256	YAPDGIAVHVDCNDEIKSLENGA	NleB1 +YM155	12 h (28 C)	12.67	0.144498	6.57
NleB1	234	256	YAPDGIAVHVDCNDEIKSLENGA	NleB1 alone	10 s (23 C)	6.74	0.055752	6.67

NleB1	234	256	YAPDGIAVHVDCNDEIKSLENGA	NleB1 alone	100 s (23 C)	8.94	0.04648	6.64
NleB1	234	256	YAPDGIAVHVDCNDEIKSLENGA	NleB1 alone	1,000 s (23 C)	10.05	0.028484	6.65
NleB1	234	256	YAPDGIAVHVDCNDEIKSLENGA	NleB1 alone	10,000 s (23 C)	11.72	0.046911	6.65
NleB1	234	256	YAPDGIAVHVDCNDEIKSLENGA	NleB1 alone	12 h (28 C)	12.82	0.110244	6.63
NleB1	241	247	VHVDCND	NleB1 +YM155	10 s (23 C)	1.81	0.062906	3.41
NleB1	241	247	VHVDCND	NleB1 +YM155	100 s (23 C)	2.27	0.01416	3.39
NleB1	241	247	VHVDCND	NleB1 +YM155	1,000 s (23 C)	2.49	0.025548	3.41
NleB1	241	247	VHVDCND	NleB1 +YM155	10,000 s (23 C)	2.46	0.015479	3.40
NleB1	241	247	VHVDCND	NleB1 +YM155	12 h (28 C)	2.63	ND	3.39
NleB1	241	247	VHVDCND	NleB1 alone	10 s (23 C)	1.81	0.026365	3.43
NleB1	241	247	VHVDCND	NleB1 alone	100 s (23 C)	2.35	0.018223	3.40
NleB1	241	247	VHVDCND	NleB1 alone	1,000 s (23 C)	2.57	0.056508	3.03
NleB1	241	247	VHVDCND	NleB1 alone	10,000 s (23 C)	2.54	0.045443	3.41
NleB1	241	247	VHVDCND	NleB1 alone	12 h (28 C)	2.59	0.026171	2.71
NleB1	242	247	HVDCND	NleB1 +YM155	10 s (23 C)	1.76	0.049424	2.50
NleB1	242	247	HVDCND	NleB1 +YM155	100 s (23 C)	1.85	0.019353	2.46
NleB1	242	247	HVDCND	NleB1 +YM155	1,000 s (23 C)	1.79	0.006799	2.47
NleB1	242	247	HVDCND	NleB1 +YM155	10,000 s (23 C)	1.77	0.019814	2.48
NleB1	242	247	HVDCND	NleB1 +YM155	12 h (28 C)	1.70	0.083772	2.46
NleB1	242	247	HVDCND	NleB1 alone	10 s (23 C)	1.74	0.019689	2.50
NleB1	242	247	HVDCND	NleB1 alone	100 s (23 C)	1.85	0.037754	2.46
NleB1	242	247	HVDCND	NleB1 alone	1,000 s (23 C)	1.80	0.006792	2.47
NleB1	242	247	HVDCND	NleB1 alone	10,000 s (23 C)	1.79	0.016758	2.49
NleB1	242	247	HVDCND	NleB1 alone	12 h (28 C)	1.78	0.024164	2.46
NleB1	242	255	HVDCNDEIKSLENG	NleB1 +YM155	10 s (23 C)	5.96	0.104945	5.54
NleB1	242	255	HVDCNDEIKSLENG	NleB1 +YM155	100 s (23 C)	7.20	0.059158	5.49
NleB1	242	255	HVDCNDEIKSLENG	NleB1 +YM155	1,000 s (23 C)	7.11	0.040284	5.50
NleB1	242	255	HVDCNDEIKSLENG	NleB1 +YM155	10,000 s (23 C)	7.10	0.074022	5.51

NleB1	242	255	HVDCNDEIKSLENG	NleB1 alone	10 s (23 C)	5.95	0.02326	5.56
NleB1	242	255	HVDCNDEIKSLENG	NleB1 alone	100 s (23 C)	7.26	0.083537	5.52
NleB1	242	255	HVDCNDEIKSLENG	NleB1 alone	1,000 s (23 C)	7.16	0.059191	5.54
NleB1	242	255	HVDCNDEIKSLENG	NleB1 alone	10,000 s (23 C)	7.12	0.02916	5.54
NleB1	242	255	HVDCNDEIKSLENG	NleB1 alone	12 h (28 C)	7.21	0.044591	5.51
NleB1	242	256	HVDCNDEIKSLENGA	NleB1 +YM155	10 s (23 C)	6.52	0.159379	5.74
NleB1	242	256	HVDCNDEIKSLENGA	NleB1 +YM155	100 s (23 C)	7.85	0.029481	5.70
NleB1	242	256	HVDCNDEIKSLENGA	NleB1 +YM155	1,000 s (23 C)	7.79	0.004477	5.71
NleB1	242	256	HVDCNDEIKSLENGA	NleB1 +YM155	10,000 s (23 C)	7.73	0.063319	5.71
NleB1	242	256	HVDCNDEIKSLENGA	NleB1 +YM155	12 h (28 C)	8.02	0.009753	5.70
NleB1	242	256	HVDCNDEIKSLENGA	NleB1 alone	10 s (23 C)	6.44	0.040818	5.77
NleB1	242	256	HVDCNDEIKSLENGA	NleB1 alone	100 s (23 C)	7.90	0.024099	5.74
NleB1	242	256	HVDCNDEIKSLENGA	NleB1 alone	1,000 s (23 C)	7.82	0.029837	5.74
NleB1	242	256	HVDCNDEIKSLENGA	NleB1 alone	10,000 s (23 C)	7.80	0.04282	5.75
NleB1	242	256	HVDCNDEIKSLENGA	NleB1 alone	12 h (28 C)	7.88	0.080717	5.73
NleB1	242	267	HVDCNDEIKSLENGAIVVNRSNHPAL	NleB1 +YM155	10 s (23 C)	7.02	0.136647	6.26
NleB1	242	267	HVDCNDEIKSLENGAIVVNRSNHPAL	NleB1 +YM155	100 s (23 C)	8.85	0.033452	6.23
NleB1	242	267	HVDCNDEIKSLENGAIVVNRSNHPAL	NleB1 +YM155	1,000 s (23 C)	9.22	0.054902	6.23
NleB1	242	267	HVDCNDEIKSLENGAIVVNRSNHPAL	NleB1 +YM155	10,000 s (23 C)	9.83	0.006903	6.23
NleB1	242	267	HVDCNDEIKSLENGAIVVNRSNHPAL	NleB1 alone	10 s (23 C)	6.85	0.035111	6.30
NleB1	242	267	HVDCNDEIKSLENGAIVVNRSNHPAL	NleB1 alone	100 s (23 C)	8.96	0.034454	6.27
NleB1	242	267	HVDCNDEIKSLENGAIVVNRSNHPAL	NleB1 alone	1,000 s (23 C)	9.14	0.031129	6.28
NleB1	242	267	HVDCNDEIKSLENGAIVVNRSNHPAL	NleB1 alone	10,000 s (23 C)	9.66	0.050121	6.29
NleB1	242	267	HVDCNDEIKSLENGAIVVNRSNHPAL	NleB1 alone	12 h (28 C)	13.12	0.065475	6.26
NleB1	246	256	NDEIKSLENGA	NleB1 +YM155	10 s (23 C)	4.86	0.093698	5.21
NleB1	246	256	NDEIKSLENGA	NleB1 +YM155	100 s (23 C)	6.15	0.015587	5.16
NleB1	246	256	NDEIKSLENGA	NleB1 +YM155	1,000 s (23 C)	6.07	0.007818	5.17
NleB1	246	256	NDEIKSLENGA	NleB1 +YM155	10,000 s (23 C)	6.05	0.047082	5.18

NleB1	246	256	NDEIKSLENGA	NleB1 +YM155	12 h (28 C)	6.35	0.045635	5.16
NleB1	246	256	NDEIKSLENGA	NleB1 alone	10 s (23 C)	4.76	0.061191	5.23
NleB1	246	256	NDEIKSLENGA	NleB1 alone	100 s (23 C)	6.14	0.015029	5.20
NleB1	246	256	NDEIKSLENGA	NleB1 alone	1,000 s (23 C)	6.09	0.037644	5.20
NleB1	246	256	NDEIKSLENGA	NleB1 alone	10,000 s (23 C)	6.07	0.030165	5.22
NleB1	246	256	NDEIKSLENGA	NleB1 alone	12 h (28 C)	6.12	0.042023	5.19
NleB1	247	256	DEIKSLENGA	NleB1 +YM155	10 s (23 C)	4.55	0.069052	5.22
NleB1	247	256	DEIKSLENGA	NleB1 +YM155	100 s (23 C)	5.73	0.082235	5.18
NleB1	247	256	DEIKSLENGA	NleB1 +YM155	1,000 s (23 C)	5.68	0.036346	5.19
NleB1	247	256	DEIKSLENGA	NleB1 +YM155	10,000 s (23 C)	5.58	0.061675	5.20
NleB1	247	256	DEIKSLENGA	NleB1 +YM155	12 h (28 C)	5.67	0.024876	5.18
NleB1	247	256	DEIKSLENGA	NleB1 alone	10 s (23 C)	4.40	0.061001	5.24
NleB1	247	256	DEIKSLENGA	NleB1 alone	100 s (23 C)	5.71	0.03771	5.21
NleB1	247	256	DEIKSLENGA	NleB1 alone	1,000 s (23 C)	5.76	0.038552	5.22
NleB1	247	256	DEIKSLENGA	NleB1 alone	10,000 s (23 C)	5.62	0.031208	5.23
NleB1	247	256	DEIKSLENGA	NleB1 alone	12 h (28 C)	5.69	0.020323	5.20
NleB1	248	255	EIKSLENG	NleB1 +YM155	10 s (23 C)	3.27	0.083455	4.06
NleB1	248	255	EIKSLENG	NleB1 +YM155	100 s (23 C)	4.19	0.027914	4.02
NleB1	248	255	EIKSLENG	NleB1 +YM155	1,000 s (23 C)	4.21	0.033543	4.03
NleB1	248	255	EIKSLENG	NleB1 +YM155	10,000 s (23 C)	4.14	0.047739	4.04
NleB1	248	255	EIKSLENG	NleB1 alone	10 s (23 C)	3.28	0.030199	4.06
NleB1	248	255	EIKSLENG	NleB1 alone	100 s (23 C)	4.25	0.035726	4.02
NleB1	248	255	EIKSLENG	NleB1 alone	1,000 s (23 C)	4.26	0.029414	4.03
NleB1	248	255	EIKSLENG	NleB1 alone	10,000 s (23 C)	4.23	0.028131	4.05
NleB1	248	255	EIKSLENG	NleB1 alone	12 h (28 C)	4.21	0.013774	4.00
NleB1	248	256	EIKSLENGA	NleB1 +YM155	10 s (23 C)	3.69	0.084265	4.30
NleB1	248	256	EIKSLENGA	NleB1 +YM155	100 s (23 C)	4.84	0.03147	4.26
NleB1	248	256	EIKSLENGA	NleB1 +YM155	1,000 s (23 C)	4.76	0.020425	4.28

NleB1	248	256	EIKSLENGA	NleB1 +YM155	10,000 s (23 C)	4.68	0.024935	4.28
NleB1	248	256	EIKSLENGA	NleB1 +YM155	12 h (28 C)	4.67	0.022652	4.26
NleB1	248	256	EIKSLENGA	NleB1 alone	10 s (23 C)	3.73	0.043954	4.30
NleB1	248	256	EIKSLENGA	NleB1 alone	100 s (23 C)	4.94	0.030843	4.27
NleB1	248	256	EIKSLENGA	NleB1 alone	1,000 s (23 C)	4.89	0.010456	4.28
NleB1	248	256	EIKSLENGA	NleB1 alone	10,000 s (23 C)	4.84	0.022191	4.29
NleB1	248	256	EIKSLENGA	NleB1 alone	12 h (28 C)	4.89	0.005698	4.26
NleB1	248	267	EIKSLENGAIVVNRSNHPAL	NleB1 +YM155	10 s (23 C)	4.17	0.111918	5.33
NleB1	248	267	EIKSLENGAIVVNRSNHPAL	NleB1 +YM155	100 s (23 C)	5.76	0.017057	5.29
NleB1	248	267	EIKSLENGAIVVNRSNHPAL	NleB1 +YM155	1,000 s (23 C)	6.07	0.049338	5.30
NleB1	248	267	EIKSLENGAIVVNRSNHPAL	NleB1 +YM155	10,000 s (23 C)	6.73	0.086577	5.30
NleB1	248	267	EIKSLENGAIVVNRSNHPAL	NleB1 alone	10 s (23 C)	4.19	0.026792	5.35
NleB1	248	267	EIKSLENGAIVVNRSNHPAL	NleB1 alone	100 s (23 C)	5.81	0.023441	5.31
NleB1	248	267	EIKSLENGAIVVNRSNHPAL	NleB1 alone	1,000 s (23 C)	6.05	0.020211	5.33
NleB1	248	267	EIKSLENGAIVVNRSNHPAL	NleB1 alone	10,000 s (23 C)	6.63	0.01634	5.33
NleB1	248	267	EIKSLENGAIVVNRSNHPAL	NleB1 alone	12 h (28 C)	10.02	0.074193	5.31
NleB1	248	268	EIKSLENGAIVVNRSNHPALL	NleB1 +YM155	10 s (23 C)	4.08	0.095827	5.90
NleB1	248	268	EIKSLENGAIVVNRSNHPALL	NleB1 +YM155	100 s (23 C)	5.67	0.027491	5.86
NleB1	248	268	EIKSLENGAIVVNRSNHPALL	NleB1 +YM155	1,000 s (23 C)	5.94	0.030976	5.87
NleB1	248	268	EIKSLENGAIVVNRSNHPALL	NleB1 +YM155	10,000 s (23 C)	6.49	0.086048	5.87
NleB1	248	268	EIKSLENGAIVVNRSNHPALL	NleB1 +YM155	12 h (28 C)	9.94	0.159926	5.87
NleB1	248	268	EIKSLENGAIVVNRSNHPALL	NleB1 alone	10 s (23 C)	4.05	0.052593	5.93
NleB1	248	268	EIKSLENGAIVVNRSNHPALL	NleB1 alone	100 s (23 C)	5.70	0.026399	5.91
NleB1	248	268	EIKSLENGAIVVNRSNHPALL	NleB1 alone	1,000 s (23 C)	5.88	0.026348	5.91
NleB1	248	268	EIKSLENGAIVVNRSNHPALL	NleB1 alone	10,000 s (23 C)	6.45	0.016482	5.92
NleB1	248	268	EIKSLENGAIVVNRSNHPALL	NleB1 alone	12 h (28 C)	9.83	0.124558	5.90
NleB1	249	268	IKSLENGAIVVNRSNHPALL	NleB1 +YM155	10 s (23 C)	3.23	0.085342	5.71
NleB1	249	268	IKSLENGAIVVNRSNHPALL	NleB1 +YM155	100 s (23 C)	4.46	0.024611	5.68

NleB1	249	268	IKSLENGAIVVNRSNHPALL	NleB1 +YM155	1,000 s (23 C)	4.81	0.067906	5.69
NleB1	249	268	IKSLENGAIVVNRSNHPALL	NleB1 +YM155	10,000 s (23 C)	5.58	0.056771	5.69
NleB1	249	268	IKSLENGAIVVNRSNHPALL	NleB1 +YM155	12 h (28 C)	8.62	0.020251	5.68
NleB1	249	268	IKSLENGAIVVNRSNHPALL	NleB1 alone	10 s (23 C)	3.17	0.033032	5.75
NleB1	249	268	IKSLENGAIVVNRSNHPALL	NleB1 alone	100 s (23 C)	4.59	0.034984	5.72
NleB1	249	268	IKSLENGAIVVNRSNHPALL	NleB1 alone	1,000 s (23 C)	4.79	0.023697	5.73
NleB1	249	268	IKSLENGAIVVNRSNHPALL	NleB1 alone	10,000 s (23 C)	5.42	0.030394	5.73
NleB1	249	268	IKSLENGAIVVNRSNHPALL	NleB1 alone	12 h (28 C)	8.72	0.015757	5.71
NleB1	250	256	KSLENGA	NleB1 +YM155	10 s (23 C)	2.92	0.07046	4.30
NleB1	250	256	KSLENGA	NleB1 +YM155	100 s (23 C)	3.78	0.041807	4.26
NleB1	250	256	KSLENGA	NleB1 +YM155	1,000 s (23 C)	3.73	0.021756	4.28
NleB1	250	256	KSLENGA	NleB1 +YM155	10,000 s (23 C)	3.70	0.025317	4.29
NleB1	250	256	KSLENGA	NleB1 alone	10 s (23 C)	2.88	0.039366	4.30
NleB1	250	256	KSLENGA	NleB1 alone	100 s (23 C)	3.83	0.029206	4.27
NleB1	250	256	KSLENGA	NleB1 alone	1,000 s (23 C)	3.84	0.013361	4.27
NleB1	250	256	KSLENGA	NleB1 alone	10,000 s (23 C)	3.81	0.016801	4.29
NleB1	250	256	KSLENGA	NleB1 alone	12 h (28 C)	3.85	0.019361	4.25
NleB1	256	267	AIVVNRSNHPAL	NleB1 +YM155	10 s (23 C)	0.43	0.064421	4.61
NleB1	256	267	AIVVNRSNHPAL	NleB1 +YM155	100 s (23 C)	0.77	0.024645	4.58
NleB1	256	267	AIVVNRSNHPAL	NleB1 +YM155	1,000 s (23 C)	0.95	0.023215	4.59
NleB1	256	267	AIVVNRSNHPAL	NleB1 +YM155	10,000 s (23 C)	1.32	0.025356	4.59
NleB1	256	267	AIVVNRSNHPAL	NleB1 +YM155	12 h (28 C)	3.02	0.122413	4.57
NleB1	256	267	AIVVNRSNHPAL	NleB1 alone	10 s (23 C)	0.42	0.026609	4.62
NleB1	256	267	AIVVNRSNHPAL	NleB1 alone	100 s (23 C)	0.64	0.024887	4.59
NleB1	256	267	AIVVNRSNHPAL	NleB1 alone	1,000 s (23 C)	0.81	0.022453	4.60
NleB1	256	267	AIVVNRSNHPAL	NleB1 alone	10,000 s (23 C)	1.16	0.017101	4.61
NleB1	256	267	AIVVNRSNHPAL	NleB1 alone	12 h (28 C)	3.11	0.007105	4.58
NleB1	256	268	AIVVNRSNHPALL	NleB1 +YM155	10 s (23 C)	0.48	0.037902	5.52

NleB1	256	268	AIVVNRSNHPALL	NleB1 +YM155	100 s (23 C)	0.73	0.013596	5.48
NleB1	256	268	AIVVNRSNHPALL	NleB1 +YM155	1,000 s (23 C)	0.99	0.002395	5.49
NleB1	256	268	AIVVNRSNHPALL	NleB1 +YM155	10,000 s (23 C)	1.62	0.029755	5.49
NleB1	256	268	AIVVNRSNHPALL	NleB1 +YM155	12 h (28 C)	4.30	0.038969	5.48
NleB1	256	268	AIVVNRSNHPALL	NleB1 alone	10 s (23 C)	0.48	0.027836	5.56
NleB1	256	268	AIVVNRSNHPALL	NleB1 alone	100 s (23 C)	0.75	0.019138	5.53
NleB1	256	268	AIVVNRSNHPALL	NleB1 alone	1,000 s (23 C)	1.01	0.030058	5.54
NleB1	256	268	AIVVNRSNHPALL	NleB1 alone	10,000 s (23 C)	1.54	0.029062	5.54
NleB1	256	268	AIVVNRSNHPALL	NleB1 alone	12 h (28 C)	4.41	0.027768	5.51
NleB1	257	267	IVVNRSNHPAL	NleB1 +YM155	10 s (23 C)	0.45	0.050438	4.41
NleB1	257	267	IVVNRSNHPAL	NleB1 +YM155	100 s (23 C)	0.67	0.009433	4.37
NleB1	257	267	IVVNRSNHPAL	NleB1 +YM155	1,000 s (23 C)	1.09	0.014803	4.39
NleB1	257	267	IVVNRSNHPAL	NleB1 +YM155	10,000 s (23 C)	1.90	0.078717	4.39
NleB1	257	267	IVVNRSNHPAL	NleB1 +YM155	12 h (28 C)	3.65	0.02996	4.38
NleB1	257	267	IVVNRSNHPAL	NleB1 alone	10 s (23 C)	0.36	0.027194	4.42
NleB1	257	267	IVVNRSNHPAL	NleB1 alone	100 s (23 C)	0.65	0.039556	4.39
NleB1	257	267	IVVNRSNHPAL	NleB1 alone	1,000 s (23 C)	0.95	0.003969	4.39
NleB1	257	267	IVVNRSNHPAL	NleB1 alone	10,000 s (23 C)	1.61	0.030445	4.41
NleB1	257	267	IVVNRSNHPAL	NleB1 alone	12 h (28 C)	3.71	0.027244	4.38
NleB1	257	268	IVVNRSNHPALL	NleB1 +YM155	10 s (23 C)	0.37	0.028925	5.37
NleB1	257	268	IVVNRSNHPALL	NleB1 +YM155	100 s (23 C)	0.51	0.050386	5.33
NleB1	257	268	IVVNRSNHPALL	NleB1 +YM155	1,000 s (23 C)	0.91	0.033036	5.35
NleB1	257	268	IVVNRSNHPALL	NleB1 +YM155	10,000 s (23 C)	1.54	0.011097	5.35
NleB1	257	268	IVVNRSNHPALL	NleB1 +YM155	12 h (28 C)	3.67	0.018834	5.34
NleB1	257	268	IVVNRSNHPALL	NleB1 alone	10 s (23 C)	0.27	0.013458	5.40
NleB1	257	268	IVVNRSNHPALL	NleB1 alone	100 s (23 C)	0.51	0.039174	5.37
NleB1	257	268	IVVNRSNHPALL	NleB1 alone	1,000 s (23 C)	0.79	0.026383	5.38
NleB1	257	268	IVVNRSNHPALL	NleB1 alone	10,000 s (23 C)	1.35	0.02756	5.39

NleB1	257	268	IVVNRSNHPALL	NleB1 alone	12 h (28 C)	3.76	0.012076	5.37
NleB1	257	271	IVVNRSNHPALLAGL	NleB1 +YM155	10 s (23 C)	0.25	0.010327	6.58
NleB1	257	271	IVVNRSNHPALLAGL	NleB1 +YM155	100 s (23 C)	0.57	0.035919	6.56
NleB1	257	271	IVVNRSNHPALLAGL	NleB1 +YM155	1,000 s (23 C)	1.77	0.078637	6.54
NleB1	257	271	IVVNRSNHPALLAGL	NleB1 +YM155	10,000 s (23 C)	3.19	0.035566	6.54
NleB1	257	271	IVVNRSNHPALLAGL	NleB1 +YM155	12 h (28 C)	5.98	0.025004	6.54
NleB1	257	271	IVVNRSNHPALLAGL	NleB1 alone	10 s (23 C)	0.20	0.049466	6.69
NleB1	257	271	IVVNRSNHPALLAGL	NleB1 alone	100 s (23 C)	0.69	0.067419	6.66
NleB1	257	271	IVVNRSNHPALLAGL	NleB1 alone	1,000 s (23 C)	1.58	0.054443	6.67
NleB1	257	271	IVVNRSNHPALLAGL	NleB1 alone	10,000 s (23 C)	3.11	0.032753	6.67
NleB1	257	271	IVVNRSNHPALLAGL	NleB1 alone	12 h (28 C)	5.70	0.076177	6.66
NleB1	258	267	VVNRSNHPAL	NleB1 +YM155	10 s (23 C)	0.46	0.044924	4.41
NleB1	258	267	VVNRSNHPAL	NleB1 +YM155	100 s (23 C)	0.76	0.021927	4.38
NleB1	258	267	VVNRSNHPAL	NleB1 +YM155	1,000 s (23 C)	1.05	0.042906	4.39
NleB1	258	267	VVNRSNHPAL	NleB1 +YM155	10,000 s (23 C)	1.67	0.036468	4.39
NleB1	258	267	VVNRSNHPAL	NleB1 +YM155	12 h (28 C)	3.40	0.065605	4.37
NleB1	258	267	VVNRSNHPAL	NleB1 alone	10 s (23 C)	0.34	0.022064	4.42
NleB1	258	267	VVNRSNHPAL	NleB1 alone	100 s (23 C)	0.65	0.00447	4.39
NleB1	258	267	VVNRSNHPAL	NleB1 alone	1,000 s (23 C)	0.89	0.0097	4.39
NleB1	258	267	VVNRSNHPAL	NleB1 alone	10,000 s (23 C)	1.43	0.012323	4.40
NleB1	258	267	VVNRSNHPAL	NleB1 alone	12 h (28 C)	3.44	0.037339	4.37
NleB1	258	268	VVNRSNHPALL	NleB1 +YM155	10 s (23 C)	0.43	0.039012	5.37
NleB1	258	268	VVNRSNHPALL	NleB1 +YM155	100 s (23 C)	0.59	0.026169	5.47
NleB1	258	268	VVNRSNHPALL	NleB1 +YM155	1,000 s (23 C)	0.88	0.019172	5.49
NleB1	258	268	VVNRSNHPALL	NleB1 +YM155	10,000 s (23 C)	1.44	0.018356	5.49
NleB1	258	268	VVNRSNHPALL	NleB1 +YM155	12 h (28 C)	3.66	0.035278	5.48
NleB1	258	268	VVNRSNHPALL	NleB1 alone	10 s (23 C)	0.35	0.028381	5.40
NleB1	258	268	VVNRSNHPALL	NleB1 alone	100 s (23 C)	0.65	0.029837	5.37

NleB1	258	268	VVNRSNHPALL	NleB1 alone	1,000 s (23 C)	0.84	0.013588	5.38
NleB1	258	268	VVNRSNHPALL	NleB1 alone	10,000 s (23 C)	1.32	0.006203	5.39
NleB1	258	268	VVNRSNHPALL	NleB1 alone	12 h (28 C)	3.67	0.109005	5.44
NleB1	259	267	VNRSNHPAL	NleB1 +YM155	10 s (23 C)	0.46	0.020093	4.41
NleB1	259	267	VNRSNHPAL	NleB1 +YM155	100 s (23 C)	0.74	0.041469	4.38
NleB1	259	267	VNRSNHPAL	NleB1 +YM155	1,000 s (23 C)	1.00	0.028698	4.39
NleB1	259	267	VNRSNHPAL	NleB1 +YM155	10,000 s (23 C)	1.65	0.029383	4.39
NleB1	259	267	VNRSNHPAL	NleB1 +YM155	12 h (28 C)	3.27	0.011388	4.37
NleB1	259	267	VNRSNHPAL	NleB1 alone	10 s (23 C)	0.36	0.020169	4.42
NleB1	259	267	VNRSNHPAL	NleB1 alone	100 s (23 C)	0.64	0.034266	4.39
NleB1	259	267	VNRSNHPAL	NleB1 alone	1,000 s (23 C)	0.85	0.017219	4.39
NleB1	259	267	VNRSNHPAL	NleB1 alone	10,000 s (23 C)	1.39	0.028643	4.40
NleB1	259	267	VNRSNHPAL	NleB1 alone	12 h (28 C)	3.31	0.015756	4.37
NleB1	260	267	NRSNHPAL	NleB1 +YM155	10 s (23 C)	0.41	0.009432	4.41
NleB1	260	267	NRSNHPAL	NleB1 +YM155	100 s (23 C)	0.78	0.026196	4.39
NleB1	260	267	NRSNHPAL	NleB1 +YM155	1,000 s (23 C)	1.06	0.030194	4.39
NleB1	260	267	NRSNHPAL	NleB1 +YM155	10,000 s (23 C)	1.60	0.043942	4.39
NleB1	260	267	NRSNHPAL	NleB1 +YM155	12 h (28 C)	3.32	0.030386	4.38
NleB1	260	267	NRSNHPAL	NleB1 alone	10 s (23 C)	0.38	0.03246	4.42
NleB1	260	267	NRSNHPAL	NleB1 alone	100 s (23 C)	0.86	0.039358	4.39
NleB1	260	267	NRSNHPAL	NleB1 alone	1,000 s (23 C)	1.03	0.035356	4.40
NleB1	260	267	NRSNHPAL	NleB1 alone	10,000 s (23 C)	1.51	0.043639	4.40
NleB1	260	267	NRSNHPAL	NleB1 alone	12 h (28 C)	3.40	0.01181	4.38
NleB1	268	274	LAGLDIM	NleB1 +YM155	10 s (23 C)	0.35	0.04935	7.75
NleB1	268	274	LAGLDIM	NleB1 +YM155	100 s (23 C)	0.98	0.029503	7.70
NleB1	268	274	LAGLDIM	NleB1 +YM155	1,000 s (23 C)	2.53	0.020482	7.73
NleB1	268	274	LAGLDIM	NleB1 +YM155	10,000 s (23 C)	3.32	0.031252	7.73
NleB1	268	274	LAGLDIM	NleB1 +YM155	12 h (28 C)	3.52	0.001801	7.72

NleB1	268	274	LAGLDIM	NleB1 alone	10 s (23 C)	0.25	0.015419	7.75
NleB1	268	274	LAGLDIM	NleB1 alone	100 s (23 C)	0.89	0.024622	7.72
NleB1	268	274	LAGLDIM	NleB1 alone	1,000 s (23 C)	2.48	0.015083	7.73
NleB1	268	274	LAGLDIM	NleB1 alone	10,000 s (23 C)	3.35	0.042074	7.74
NleB1	268	274	LAGLDIM	NleB1 alone	12 h (28 C)	3.53	0.056454	7.72
NleB1	269	274	AGLDIM	NleB1 +YM155	10 s (23 C)	0.34	0.026888	7.13
NleB1	269	274	AGLDIM	NleB1 +YM155	100 s (23 C)	0.82	0.05547	7.09
NleB1	269	274	AGLDIM	NleB1 +YM155	1,000 s (23 C)	1.92	0.017272	7.10
NleB1	269	274	AGLDIM	NleB1 +YM155	10,000 s (23 C)	2.39	0.020275	7.11
NleB1	269	274	AGLDIM	NleB1 +YM155	12 h (28 C)	2.50	0.016873	7.10
NleB1	269	274	AGLDIM	NleB1 alone	10 s (23 C)	0.23	0.007705	7.13
NleB1	269	274	AGLDIM	NleB1 alone	100 s (23 C)	0.76	0.009075	7.10
NleB1	269	274	AGLDIM	NleB1 alone	1,000 s (23 C)	1.98	0.022082	7.11
NleB1	269	274	AGLDIM	NleB1 alone	10,000 s (23 C)	2.55	0.020986	7.12
NleB1	269	274	AGLDIM	NleB1 alone	12 h (28 C)	2.64	0.037205	7.10
NleB1	270	282	GLDIMKSKVDAHP	NleB1 +YM155	10 s (23 C)	0.96	0.01768	6.08
NleB1	270	282	GLDIMKSKVDAHP	NleB1 +YM155	100 s (23 C)	1.02	0.007546	6.04
NleB1	270	282	GLDIMKSKVDAHP	NleB1 +YM155	1,000 s (23 C)	1.59	0.024654	6.05
NleB1	270	282	GLDIMKSKVDAHP	NleB1 +YM155	10,000 s (23 C)	3.02	0.070654	6.05
NleB1	270	282	GLDIMKSKVDAHP	NleB1 +YM155	12 h (28 C)	4.09	0.012907	6.05
NleB1	270	282	GLDIMKSKVDAHP	NleB1 alone	10 s (23 C)	0.92	0.015988	6.12
NleB1	270	282	GLDIMKSKVDAHP	NleB1 alone	100 s (23 C)	1.02	0.013806	6.10
NleB1	270	282	GLDIMKSKVDAHP	NleB1 alone	1,000 s (23 C)	1.48	0.005198	6.10
NleB1	270	282	GLDIMKSKVDAHP	NleB1 alone	10,000 s (23 C)	2.82	0.026776	6.11
NleB1	270	282	GLDIMKSKVDAHP	NleB1 alone	12 h (28 C)	4.10	0.053688	6.08
NleB1	272	297	DIMKSKVDAHPYYDGLGKGIKRHFNY	NleB1 +YM155	10 s (23 C)	4.18	0.108461	5.29
NleB1	272	297	DIMKSKVDAHPYYDGLGKGIKRHFNY	NleB1 +YM155	100 s (23 C)	8.45	0.031387	5.24
NleB1	272	297	DIMKSKVDAHPYYDGLGKGIKRHFNY	NleB1 +YM155	1,000 s (23 C)	10.55	0.021682	5.24

NleB1	272	297	DIMKSKVDAHPYYDGLGKGIKRHFNY	NleB1 +YM155	10,000 s (23 C)	10.49	0.106367	5.25
NleB1	272	297	DIMKSKVDAHPYYDGLGKGIKRHFNY	NleB1 +YM155	12 h (28 C)	10.83	0.043907	5.24
NleB1	272	297	DIMKSKVDAHPYYDGLGKGIKRHFNY	NleB1 alone	10 s (23 C)	3.94	0.040152	5.30
NleB1	272	297	DIMKSKVDAHPYYDGLGKGIKRHFNY	NleB1 alone	100 s (23 C)	8.40	0.066851	5.26
NleB1	272	297	DIMKSKVDAHPYYDGLGKGIKRHFNY	NleB1 alone	1,000 s (23 C)	10.62	0.095374	5.26
NleB1	272	297	DIMKSKVDAHPYYDGLGKGIKRHFNY	NleB1 alone	10,000 s (23 C)	10.66	0.1237	5.27
NleB1	272	297	DIMKSKVDAHPYYDGLGKGIKRHFNY	NleB1 alone	12 h (28 C)	10.70	0.099656	5.25
NleB1	272	300	DIMKSKVDAHPYYDGLGKGIKRHFNYSSL	NleB1 +YM155	10 s (23 C)	7.47	0.180517	5.62
NleB1	272	300	DIMKSKVDAHPYYDGLGKGIKRHFNYSSL	NleB1 +YM155	100 s (23 C)	12.58	0.249552	5.58
NleB1	272	300	DIMKSKVDAHPYYDGLGKGIKRHFNYSSL	NleB1 +YM155	1,000 s (23 C)	15.14	0.037396	5.57
NleB1	272	300	DIMKSKVDAHPYYDGLGKGIKRHFNYSSL	NleB1 +YM155	10,000 s (23 C)	15.06	0.182251	5.58
NleB1	272	300	DIMKSKVDAHPYYDGLGKGIKRHFNYSSL	NleB1 +YM155	12 h (28 C)	15.57	0.003311	5.57
NleB1	272	300	DIMKSKVDAHPYYDGLGKGIKRHFNYSSL	NleB1 alone	10 s (23 C)	7.17	0.09472	5.63
NleB1	272	300	DIMKSKVDAHPYYDGLGKGIKRHFNYSSL	NleB1 alone	100 s (23 C)	12.47	0.118578	5.60
NleB1	272	300	DIMKSKVDAHPYYDGLGKGIKRHFNYSSL	NleB1 alone	1,000 s (23 C)	15.19	0.111563	5.60
NleB1	272	300	DIMKSKVDAHPYYDGLGKGIKRHFNYSSL	NleB1 alone	10,000 s (23 C)	15.28	0.082497	5.60
NleB1	272	300	DIMKSKVDAHPYYDGLGKGIKRHFNYSSL	NleB1 alone	12 h (28 C)	15.48	0.16231	5.58
NleB1	273	295	IMKSKVDAHPYYDGLGKGIKRHF	NleB1 +YM155	10 s (23 C)	2.79	0.094431	4.78
NleB1	273	295	IMKSKVDAHPYYDGLGKGIKRHF	NleB1 +YM155	100 s (23 C)	5.20	0.033314	4.73
NleB1	273	295	IMKSKVDAHPYYDGLGKGIKRHF	NleB1 +YM155	1,000 s (23 C)	6.74	0.021136	4.73
NleB1	273	295	IMKSKVDAHPYYDGLGKGIKRHF	NleB1 +YM155	10,000 s (23 C)	6.73	0.078476	4.74
NleB1	273	295	IMKSKVDAHPYYDGLGKGIKRHF	NleB1 +YM155	12 h (28 C)	6.96	0.054994	4.73
NleB1	273	295	IMKSKVDAHPYYDGLGKGIKRHF	NleB1 alone	10 s (23 C)	2.59	0.071353	4.79
NleB1	273	295	IMKSKVDAHPYYDGLGKGIKRHF	NleB1 alone	100 s (23 C)	5.11	0.051006	4.75
NleB1	273	295	IMKSKVDAHPYYDGLGKGIKRHF	NleB1 alone	1,000 s (23 C)	6.85	0.057593	4.75
NleB1	273	295	IMKSKVDAHPYYDGLGKGIKRHF	NleB1 alone	10,000 s (23 C)	6.81	0.01739	4.76
NleB1	273	295	IMKSKVDAHPYYDGLGKGIKRHF	NleB1 alone	12 h (28 C)	6.90	0.09683	4.74
NleB1	273	299	IMKSKVDAHPYYDGLGKGIKRHFNYSS	NleB1 +YM155	10 s (23 C)	4.91	0.142581	5.44

NleB1	273	299	IMKSKVDAHPYYDGLGKGIKRHFNYSS	NleB1 +YM155	100 s (23 C)	7.89	0.035356	5.40
NleB1	273	299	IMKSKVDAHPYYDGLGKGIKRHFNYSS	NleB1 +YM155	1,000 s (23 C)	9.76	0.023365	5.41
NleB1	273	299	IMKSKVDAHPYYDGLGKGIKRHFNYSS	NleB1 +YM155	10,000 s (23 C)	9.73	0.249838	5.41
NleB1	273	299	IMKSKVDAHPYYDGLGKGIKRHFNYSS	NleB1 +YM155	12 h (28 C)	10.21	0.018874	5.40
NleB1	273	299	IMKSKVDAHPYYDGLGKGIKRHFNYSS	NleB1 alone	10 s (23 C)	4.67	0.038439	5.47
NleB1	273	299	IMKSKVDAHPYYDGLGKGIKRHFNYSS	NleB1 alone	100 s (23 C)	8.00	0.021314	5.42
NleB1	273	299	IMKSKVDAHPYYDGLGKGIKRHFNYSS	NleB1 alone	1,000 s (23 C)	10.07	0.103751	5.42
NleB1	273	299	IMKSKVDAHPYYDGLGKGIKRHFNYSS	NleB1 alone	10,000 s (23 C)	10.04	0.129426	5.43
NleB1	273	299	IMKSKVDAHPYYDGLGKGIKRHFNYSS	NleB1 alone	12 h (28 C)	10.25	0.106769	5.41
NleB1	275	300	KSKVDAHPYYDGLGKGIKRHFNYSSL	NleB1 +YM155	10 s (23 C)	6.37	0.207711	5.38
NleB1	275	300	KSKVDAHPYYDGLGKGIKRHFNYSSL	NleB1 +YM155	100 s (23 C)	10.21	0.025198	5.33
NleB1	275	300	KSKVDAHPYYDGLGKGIKRHFNYSSL	NleB1 +YM155	1,000 s (23 C)	12.14	0.030058	5.33
NleB1	275	300	KSKVDAHPYYDGLGKGIKRHFNYSSL	NleB1 +YM155	10,000 s (23 C)	11.99	0.109363	5.35
NleB1	275	300	KSKVDAHPYYDGLGKGIKRHFNYSSL	NleB1 +YM155	12 h (28 C)	12.56	0.019105	5.33
NleB1	275	300	KSKVDAHPYYDGLGKGIKRHFNYSSL	NleB1 alone	10 s (23 C)	6.09	0.100016	5.40
NleB1	275	300	KSKVDAHPYYDGLGKGIKRHFNYSSL	NleB1 alone	100 s (23 C)	10.11	0.046845	5.36
NleB1	275	300	KSKVDAHPYYDGLGKGIKRHFNYSSL	NleB1 alone	1,000 s (23 C)	12.29	0.10615	5.36
NleB1	275	300	KSKVDAHPYYDGLGKGIKRHFNYSSL	NleB1 alone	10,000 s (23 C)	12.25	0.078771	5.37
NleB1	275	300	KSKVDAHPYYDGLGKGIKRHFNYSSL	NleB1 alone	12 h (28 C)	12.38	0.197713	5.35
NleB1	296	309	NYSSLHNYNAFCDF	NleB1 +YM155	10 s (23 C)	5.39	0.195326	4.23
NleB1	296	309	NYSSLHNYNAFCDF	NleB1 +YM155	100 s (23 C)	6.23	0.069294	4.20
NleB1	296	309	NYSSLHNYNAFCDF	NleB1 +YM155	1,000 s (23 C)	6.51	0.057513	4.21
NleB1	296	309	NYSSLHNYNAFCDF	NleB1 +YM155	10,000 s (23 C)	6.47	0.096527	4.22
NleB1	296	309	NYSSLHNYNAFCDF	NleB1 +YM155	12 h (28 C)	6.79	0.048251	4.20
NleB1	296	309	NYSSLHNYNAFCDF	NleB1 alone	10 s (23 C)	5.47	0.097955	4.24
NleB1	296	309	NYSSLHNYNAFCDF	NleB1 alone	100 s (23 C)	6.44	0.065921	4.21
NleB1	296	309	NYSSLHNYNAFCDF	NleB1 alone	1,000 s (23 C)	6.62	0.069024	4.21
NleB1	296	309	NYSSLHNYNAFCDF	NleB1 alone	10,000 s (23 C)	6.64	0.06118	4.23

NleB1	296	309	NYSSLHNYNAFCDF	NleB1 alone	12 h (28 C)	7.01	0.189903	4.20
NleB1	310	323	IEFKHENIIPNTSM	NleB1 +YM155	10 s (23 C)	3.99	0.099705	5.96
NleB1	310	323	IEFKHENIIPNTSM	NleB1 +YM155	100 s (23 C)	5.18	0.023122	5.92
NleB1	310	323	IEFKHENIIPNTSM	NleB1 +YM155	1,000 s (23 C)	5.86	0.011969	5.93
NleB1	310	323	IEFKHENIIPNTSM	NleB1 +YM155	10,000 s (23 C)	5.90	0.03833	5.93
NleB1	310	323	IEFKHENIIPNTSM	NleB1 +YM155	12 h (28 C)	6.14	0.038787	5.94
NleB1	310	323	IEFKHENIIPNTSM	NleB1 alone	10 s (23 C)	3.87	0.030005	6.00
NleB1	310	323	IEFKHENIIPNTSM	NleB1 alone	100 s (23 C)	5.18	0.051531	5.97
NleB1	310	323	IEFKHENIIPNTSM	NleB1 alone	1,000 s (23 C)	5.95	0.023312	5.98
NleB1	310	323	IEFKHENIIPNTSM	NleB1 alone	10,000 s (23 C)	5.96	0.02513	5.99
NleB1	310	323	IEFKHENIIPNTSM	NleB1 alone	12 h (28 C)	6.15	0.085588	5.97
NleB1	312	323	FKHENIIPNTSM	NleB1 +YM155	10 s (23 C)	3.93	0.095977	5.48
NleB1	312	323	FKHENIIPNTSM	NleB1 +YM155	100 s (23 C)	4.74	0.059635	5.45
NleB1	312	323	FKHENIIPNTSM	NleB1 +YM155	1,000 s (23 C)	4.83	0.025874	5.46
NleB1	312	323	FKHENIIPNTSM	NleB1 +YM155	10,000 s (23 C)	4.84	0.03608	5.45
NleB1	312	323	FKHENIIPNTSM	NleB1 +YM155	12 h (28 C)	5.02	0.02793	5.45
NleB1	312	323	FKHENIIPNTSM	NleB1 alone	10 s (23 C)	3.81	0.044515	5.51
NleB1	312	323	FKHENIIPNTSM	NleB1 alone	100 s (23 C)	4.76	0.047112	5.48
NleB1	312	323	FKHENIIPNTSM	NleB1 alone	1,000 s (23 C)	4.92	0.038216	5.49
NleB1	312	323	FKHENIIPNTSM	NleB1 alone	10,000 s (23 C)	4.89	0.023503	5.50
NleB1	312	323	FKHENIIPNTSM	NleB1 alone	12 h (28 C)	5.04	0.039095	5.48
NleB1	312	325	FKHENIIPNTSMYT	NleB1 +YM155	10 s (23 C)	4.01	0.082101	5.96
NleB1	312	325	FKHENIIPNTSMYT	NleB1 +YM155	100 s (23 C)	5.23	0.050084	5.92
NleB1	312	325	FKHENIIPNTSMYT	NleB1 +YM155	1,000 s (23 C)	5.93	0.021961	5.93
NleB1	312	325	FKHENIIPNTSMYT	NleB1 +YM155	10,000 s (23 C)	5.90	0.050303	5.93
NleB1	312	325	FKHENIIPNTSMYT	NleB1 +YM155	12 h (28 C)	6.25	0.03927	5.94
NleB1	312	325	FKHENIIPNTSMYT	NleB1 alone	10 s (23 C)	3.88	0.039039	6.00
NleB1	312	325	FKHENIIPNTSMYT	NleB1 alone	100 s (23 C)	5.18	0.020723	5.97

NleB1	312	325	FKHENIIPNTSMYT	NleB1 alone	1,000 s (23 C)	5.98	0.020453	5.98
NleB1	312	325	FKHENIIPNTSMYT	NleB1 alone	10,000 s (23 C)	5.93	0.036641	5.99
NleB1	312	325	FKHENIIPNTSMYT	NleB1 alone	12 h (28 C)	6.15	0.138167	5.97
NleB1	313	323	KHENIIPNTSM	NleB1 +YM155	10 s (23 C)	3.32	0.109444	5.08
NleB1	313	323	KHENIIPNTSM	NleB1 +YM155	100 s (23 C)	4.08	0.055815	5.04
NleB1	313	323	KHENIIPNTSM	NleB1 +YM155	1,000 s (23 C)	4.17	0.03145	5.06
NleB1	313	323	KHENIIPNTSM	NleB1 +YM155	10,000 s (23 C)	4.22	0.017643	5.06
NleB1	313	323	KHENIIPNTSM	NleB1 +YM155	12 h (28 C)	4.29	0.05192	5.06
NleB1	313	323	KHENIIPNTSM	NleB1 alone	10 s (23 C)	3.26	0.017516	5.10
NleB1	313	323	KHENIIPNTSM	NleB1 alone	100 s (23 C)	4.10	0.036581	5.08
NleB1	313	323	KHENIIPNTSM	NleB1 alone	1,000 s (23 C)	4.27	0.055357	5.08
NleB1	313	323	KHENIIPNTSM	NleB1 alone	10,000 s (23 C)	4.28	0.033299	5.09
NleB1	313	323	KHENIIPNTSM	NleB1 alone	12 h (28 C)	4.33	0.014751	5.07

Table S7. Deuterium uptake values, uptake standard deviation (SD) and retention time (RT) of the individual NleB1 peptides whose HDX was followed at every time point and in both states (apo- and YM155 bound NleB1).

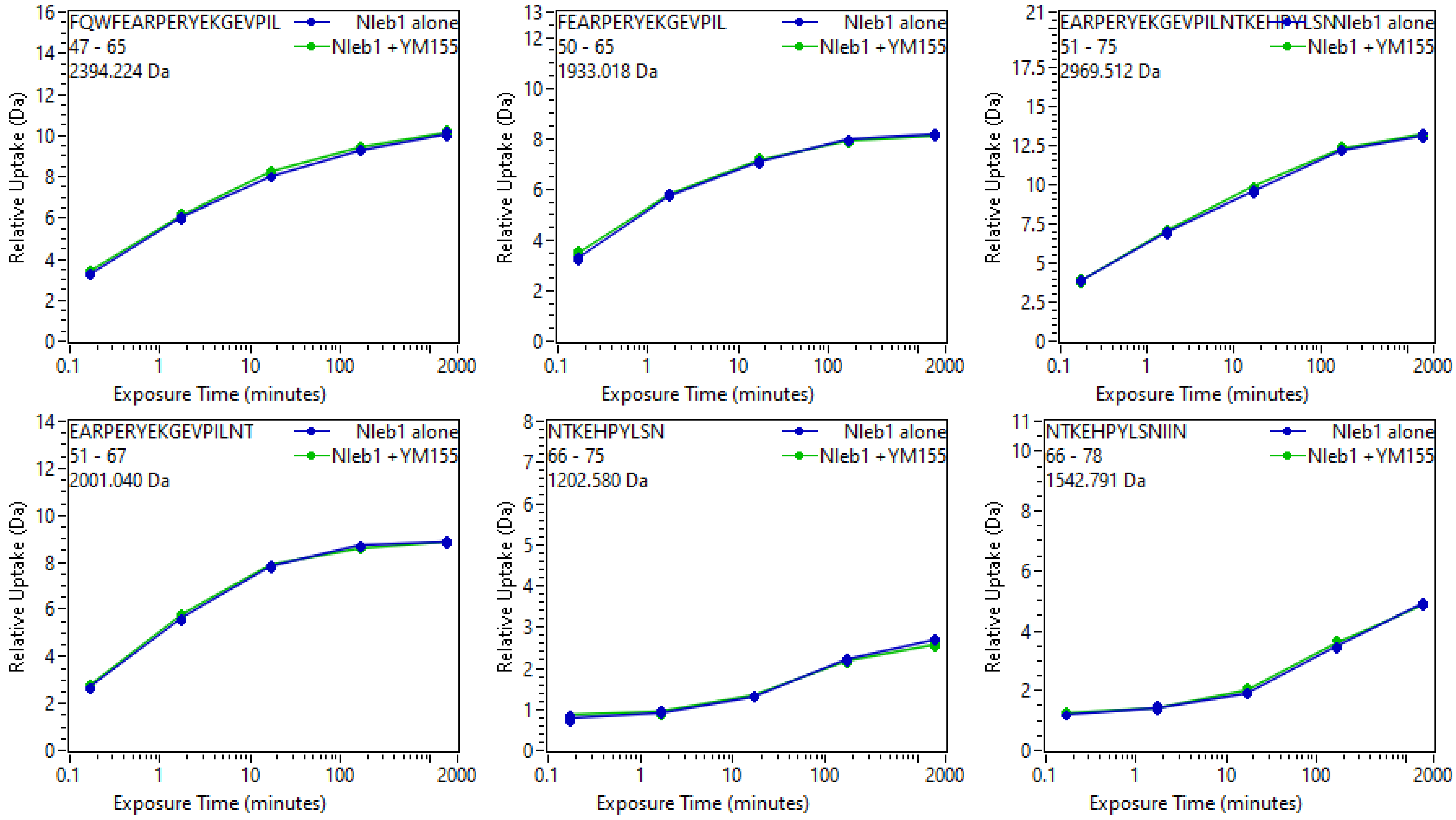

FQWFEARPERYEKGEVPIL
47 - 65
2394.224 Da
FEARPERYEKGEVPIL
50 - 65
1933.018 Da
EARPERYEKGEVPILNTKEHPYLSN
51 - 75
2969.512 Da
EARPERYEKGEVPILNT
51 - 67
2001.040 Da
NTKEHPYLSN
66 - 75
1202.580 Da
NTKEHPYLSNIIN
66 - 78
1542.791 Da
Nleb1 alone
Nleb1 +YM155
Relative Uptake (Da)
Exposure Time (minutes)

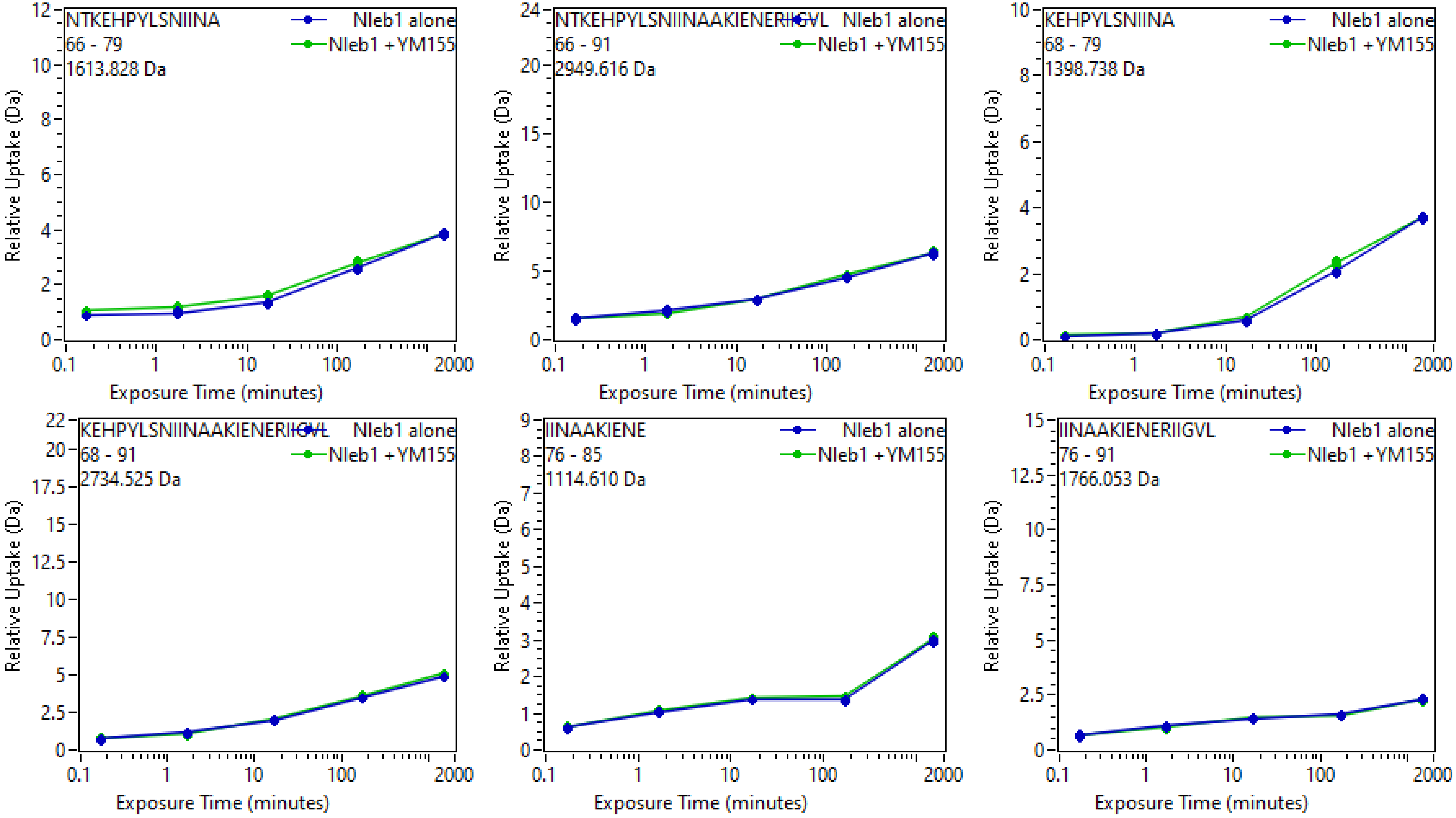
NTKEHPYLSNIINA
66 - 79
1613.828 Da
Nleb1 alone
Nleb1 +YM155
NTKEHPYLSNIINAAKIENERIIGVL
66 - 91
2949.616 Da
Nleb1 alone
Nleb1 +YM155
KEHPYLSNIINA
68 - 79
1398.738 Da
Nleb1 alone
Nleb1 +YM155
KEHPYLSNIINAAKIENERIIGVL
68 - 91
2734.525 Da
Nleb1 alone
Nleb1 +YM155
IINAAKIENE
76 - 85
1114.610 Da
Nleb1 alone
Nleb1 +YM155
IINAAKIENERIIGVL
76 - 91
1766.053 Da
Nleb1 alone
Nleb1 +YM155
Relative Uptake (Da)
Exposure Time (minutes)
0.1
1
10
100
2000

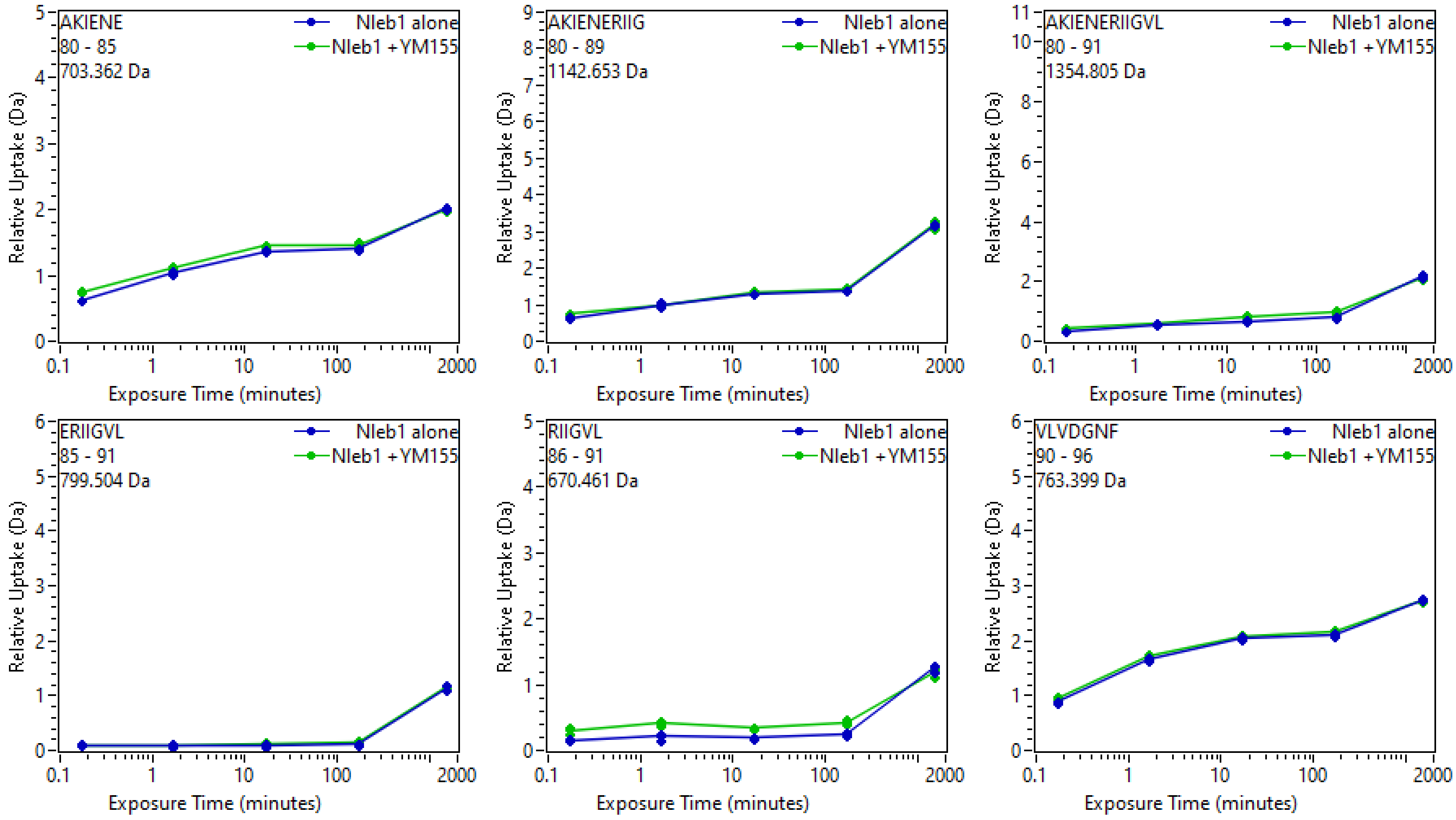
AKIENE
80 - 85
703.362 Da
AKIENERIIG
80 - 89
1142.653 Da
AKIENERIIGVL
80 - 91
1354.805 Da
ERIIGVL
85 - 91
799.504 Da
RIIGVL
86 - 91
670.461 Da
VLVDGNF
90 - 96
763.399 Da
Nleb1 alone
Nleb1 +YM155
Relative Uptake (Da)
Exposure Time (minutes)

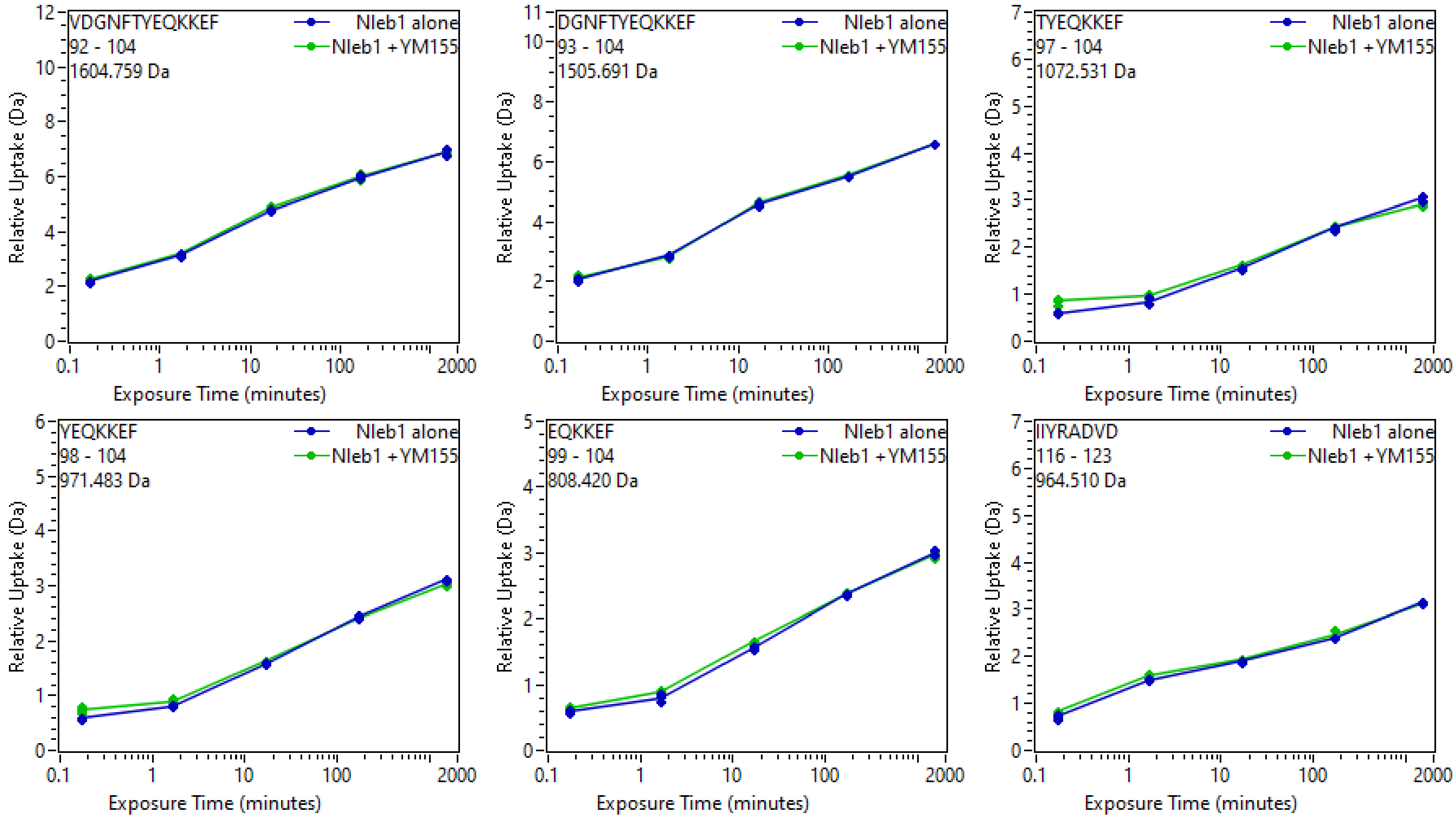
VDGNFTYEQKKEF
92 - 104
1604.759 Da
DGNFTYEQKKEF
93 - 104
1505.691 Da
TYEQKKEF
97 - 104
1072.531 Da
YEQKKEF
98 - 104
971.483 Da
EQKKEF
99 - 104
808.420 Da
IIYRADVD
116 - 123
964.510 Da
Nleb1 alone
Nleb1 +YM155
Relative Uptake (Da)
Exposure Time (minutes)
0.1
1
10
100
2000

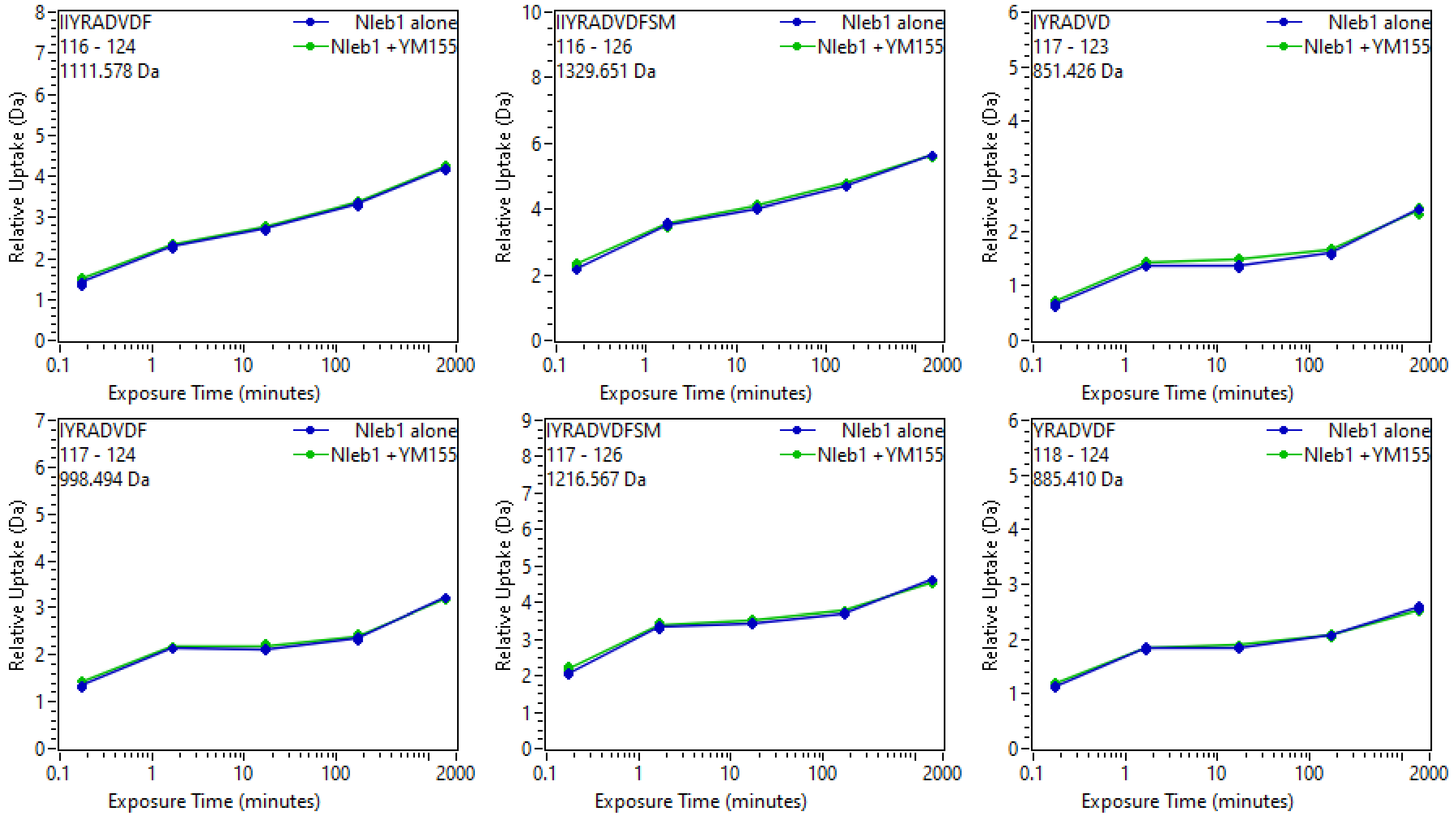
IIYRADVDF
116 - 124
1111.578 Da
IIYRADVDFSM
116 - 126
1329.651 Da
IYRADVD
117 - 123
851.426 Da
IYRADVDF
117 - 124
998.494 Da
IYRADVDFSM
117 - 126
1216.567 Da
YRADVDF
118 - 124
885.410 Da
Nleb1 alone
Nleb1 +YM155
Relative Uptake (Da)
Exposure Time (minutes)
0.1
1
10
100
2000

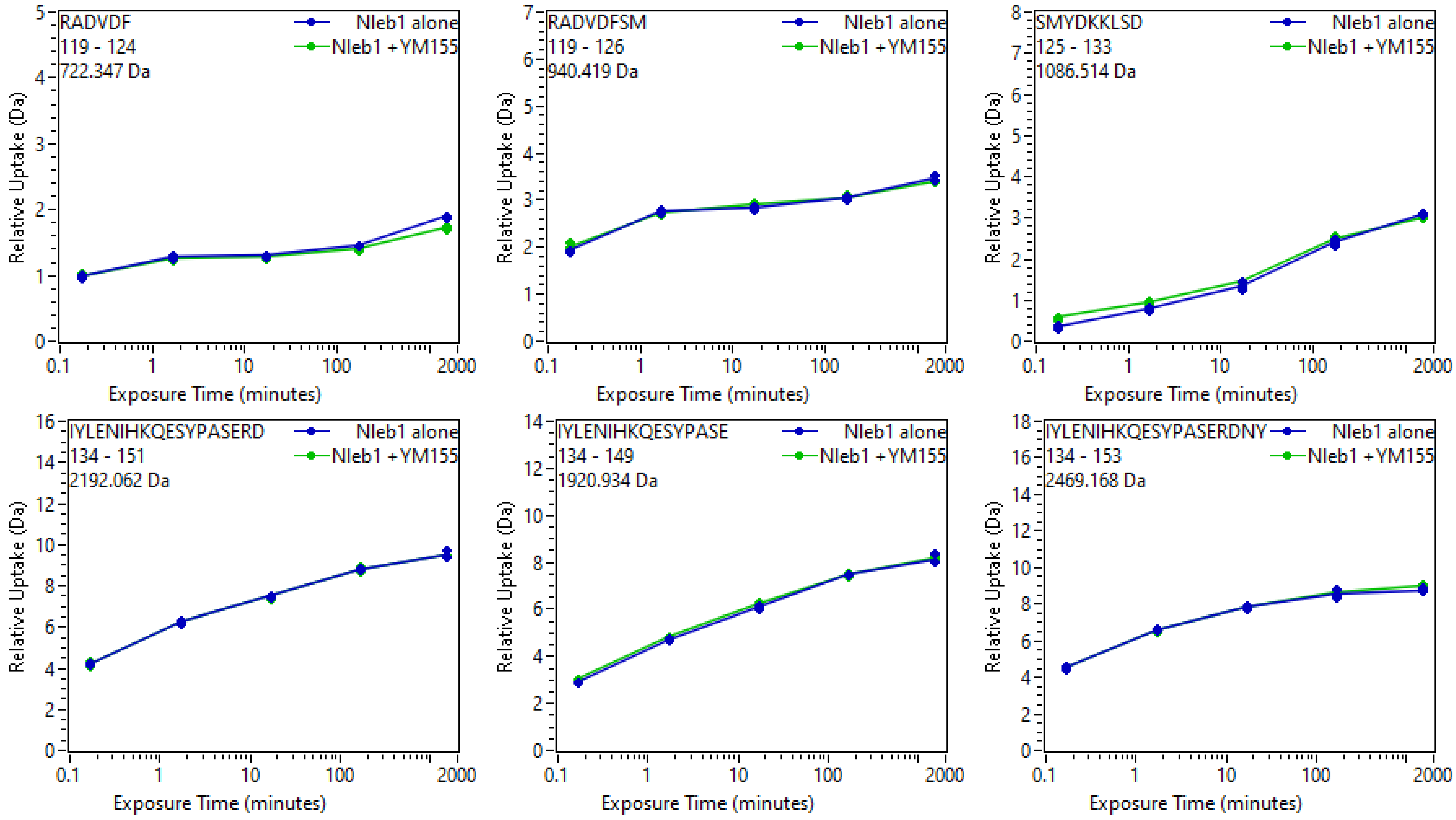
RADVDF
119 - 124
722.347 Da
Nleb1 alone
Nleb1 +YM155
RADVDFSM
119 - 126
940.419 Da
SMYDKKLSD
125 - 133
1086.514 Da
IYLENIHKQESYPASERD
134 - 151
2192.062 Da
IYLENIHKQESYPASE
134 - 149
1920.934 Da
IYLENIHKQESYPASERDNY
134 - 153
2469.168 Da
Relative Uptake (Da)
Exposure Time (minutes)
0.1
1
10
100
2000

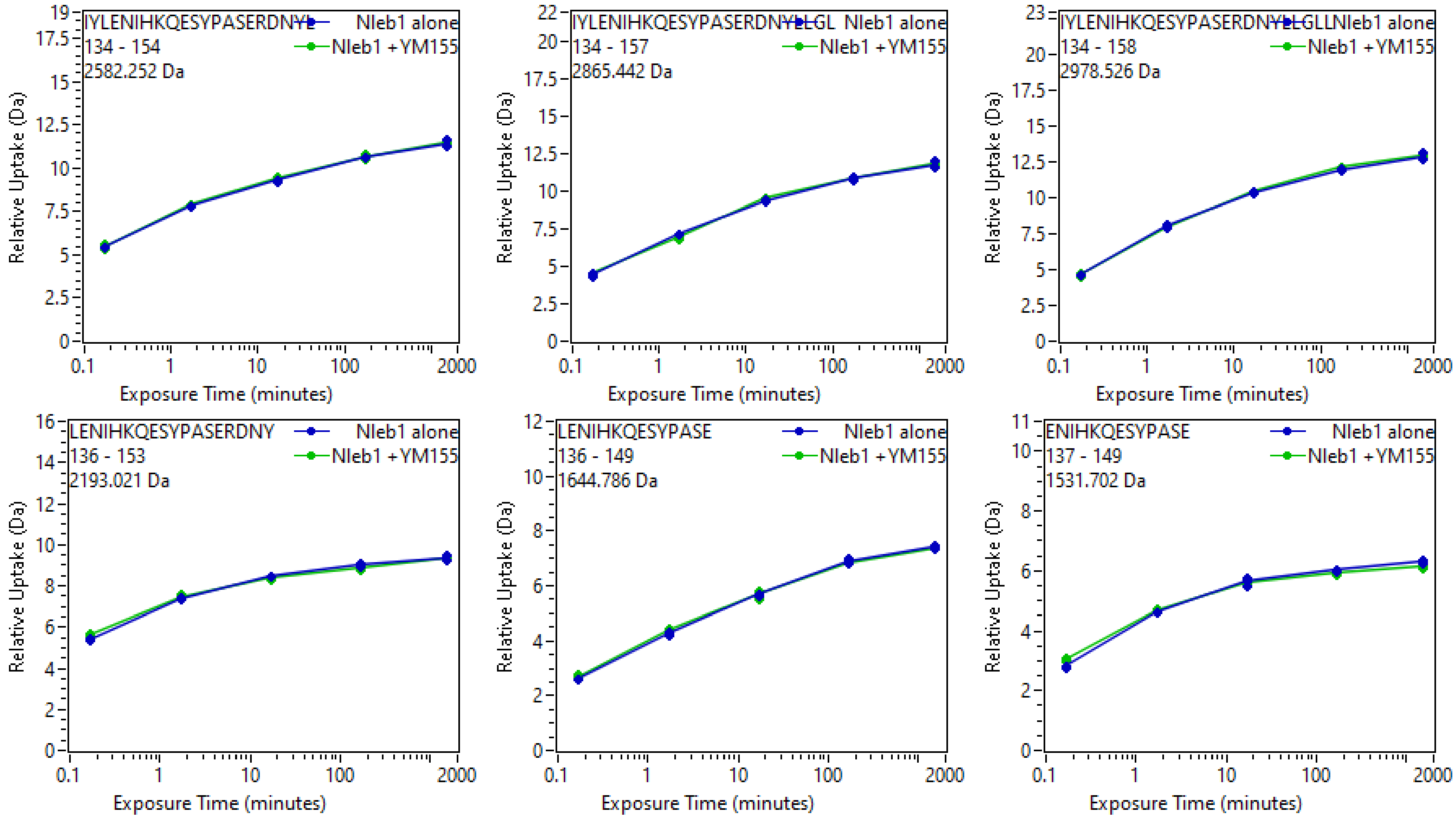
IYLENIHKQESYPASERDNYL
134 - 154
2582.252 Da
IYLENIHKQESYPASERDNYLLGL
134 - 157
2865.442 Da
IYLENIHKQESYPASERDNYLLGLL
134 - 158
2978.526 Da
LENIHKQESYPASERDNY
136 - 153
2193.021 Da
LENIHKQESYPASE
136 - 149
1644.786 Da
ENIHKQESYPASE
137 - 149
1531.702 Da
Nleb1 alone
Nleb1 +YM155
Relative Uptake (Da)
Exposure Time (minutes)
0.1
1
10
100
2000

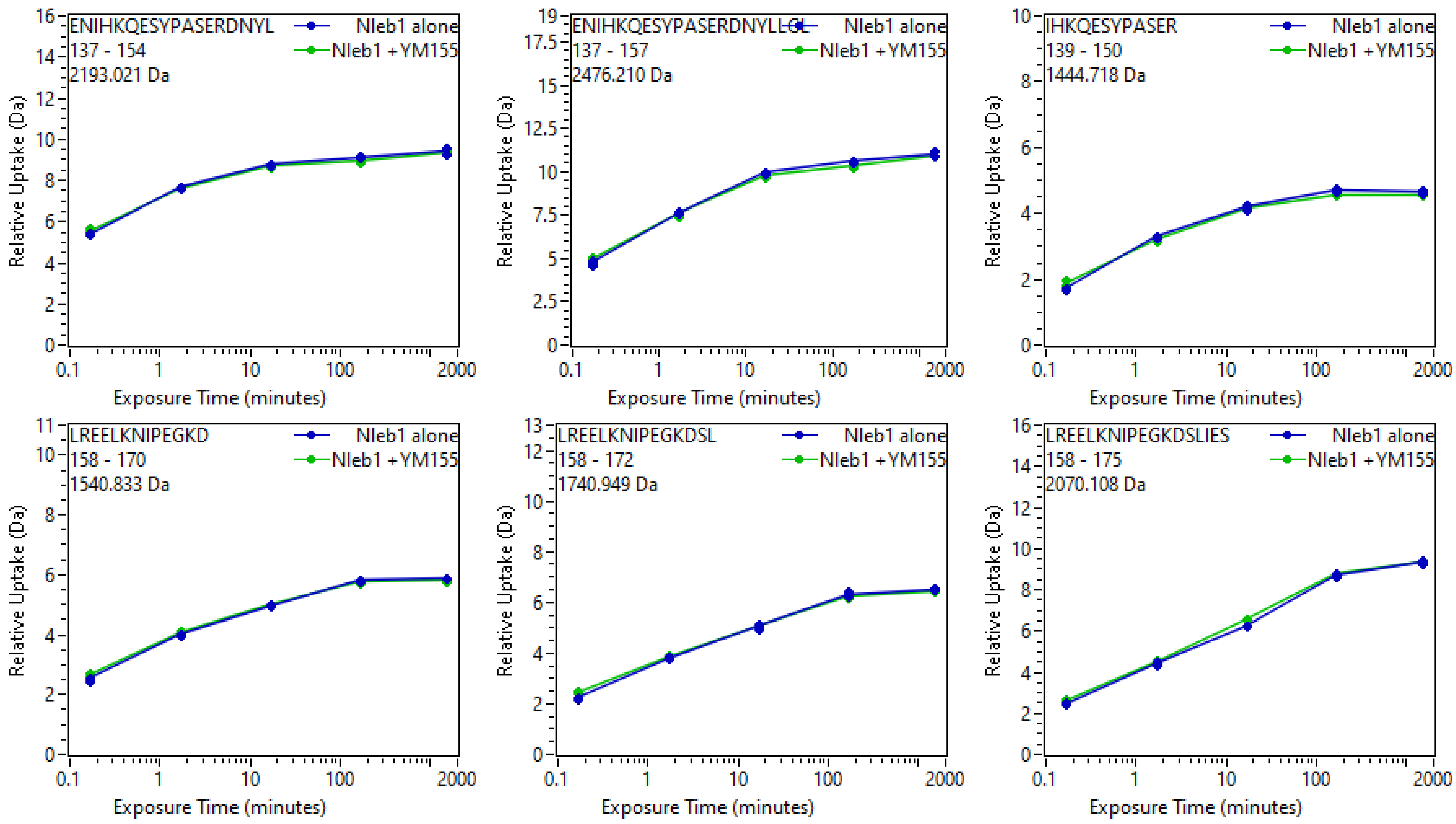
ENIHKQESYPASERDNYL
137 - 154
2193.021 Da
Nleb1 alone
Nleb1 +YM155
Relative Uptake (Da)
Exposure Time (minutes)
ENIHKQESYPASERDNYLLGL
137 - 157
2476.210 Da
IHKQESYPASER
139 - 150
1444.718 Da
LREELKNIPEGKD
158 - 170
1540.833 Da
LREELKNIPEGKDSL
158 - 172
1740.949 Da
LREELKNIPEGKDSLIES
158 - 175
2070.108 Da

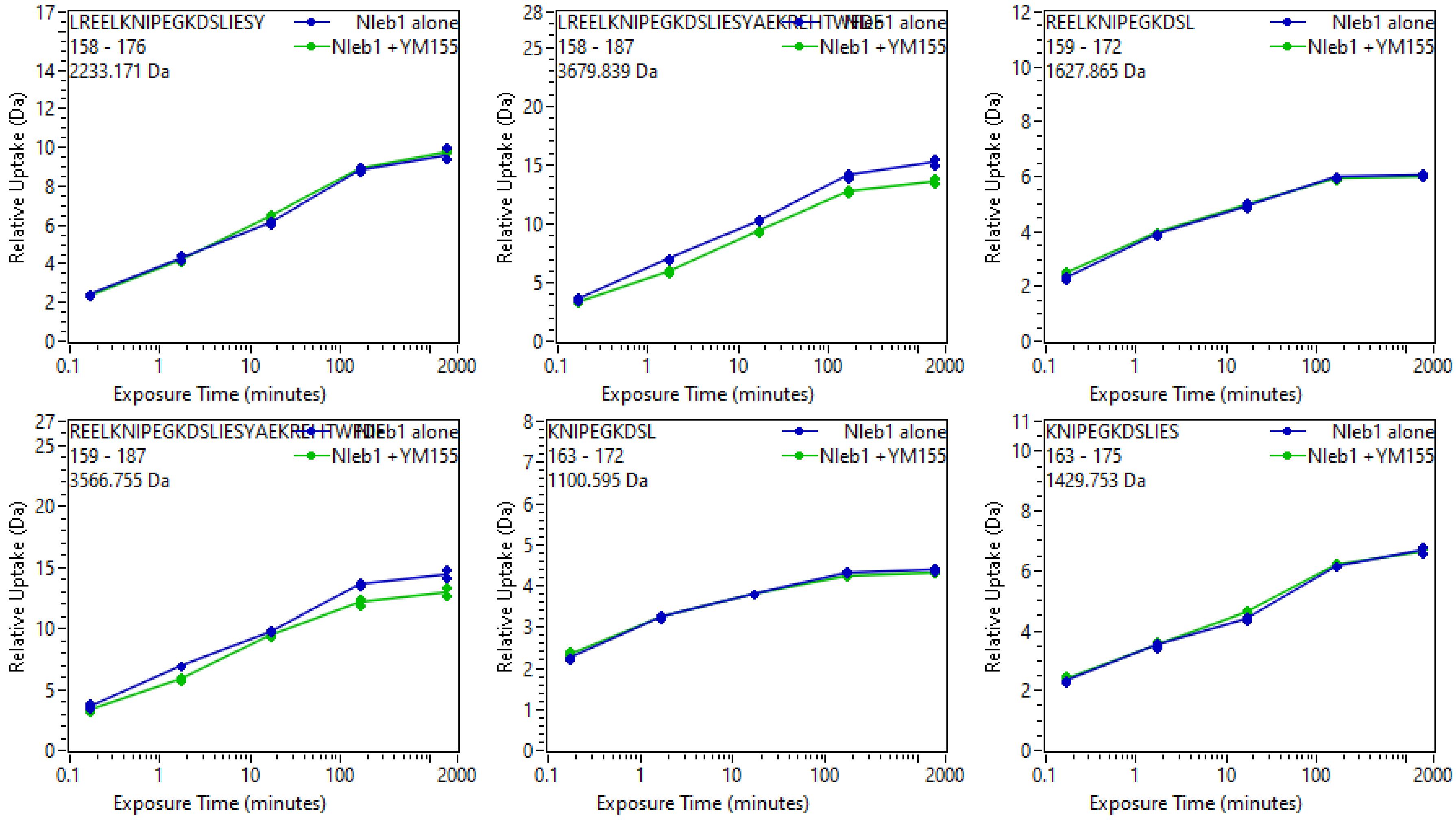
LREELKNIPEGKDSLIESY
158 - 176
2233.171 Da
Nleb1 alone
Nleb1 +YM155
Relative Uptake (Da)
Exposure Time (minutes)
LREELKNIPEGKDSLIESYAEKR
158 - 187
3679.839 Da
Nleb1 alone
Nleb1 +YM155
Relative Uptake (Da)
Exposure Time (minutes)
REELKNIPEGKDSL
159 - 172
1627.865 Da
Nleb1 alone
Nleb1 +YM155
Relative Uptake (Da)
Exposure Time (minutes)
REELKNIPEGKDSLIESYAEKR
159 - 187
3566.755 Da
Nleb1 alone
Nleb1 +YM155
Relative Uptake (Da)
Exposure Time (minutes)
KNIPEGKDSL
163 - 172
1100.595 Da
Nleb1 alone
Nleb1 +YM155
Relative Uptake (Da)
Exposure Time (minutes)
KNIPEGKDSLIES
163 - 175
1429.753 Da
Nleb1 alone
Nleb1 +YM155
Relative Uptake (Da)
Exposure Time (minutes)

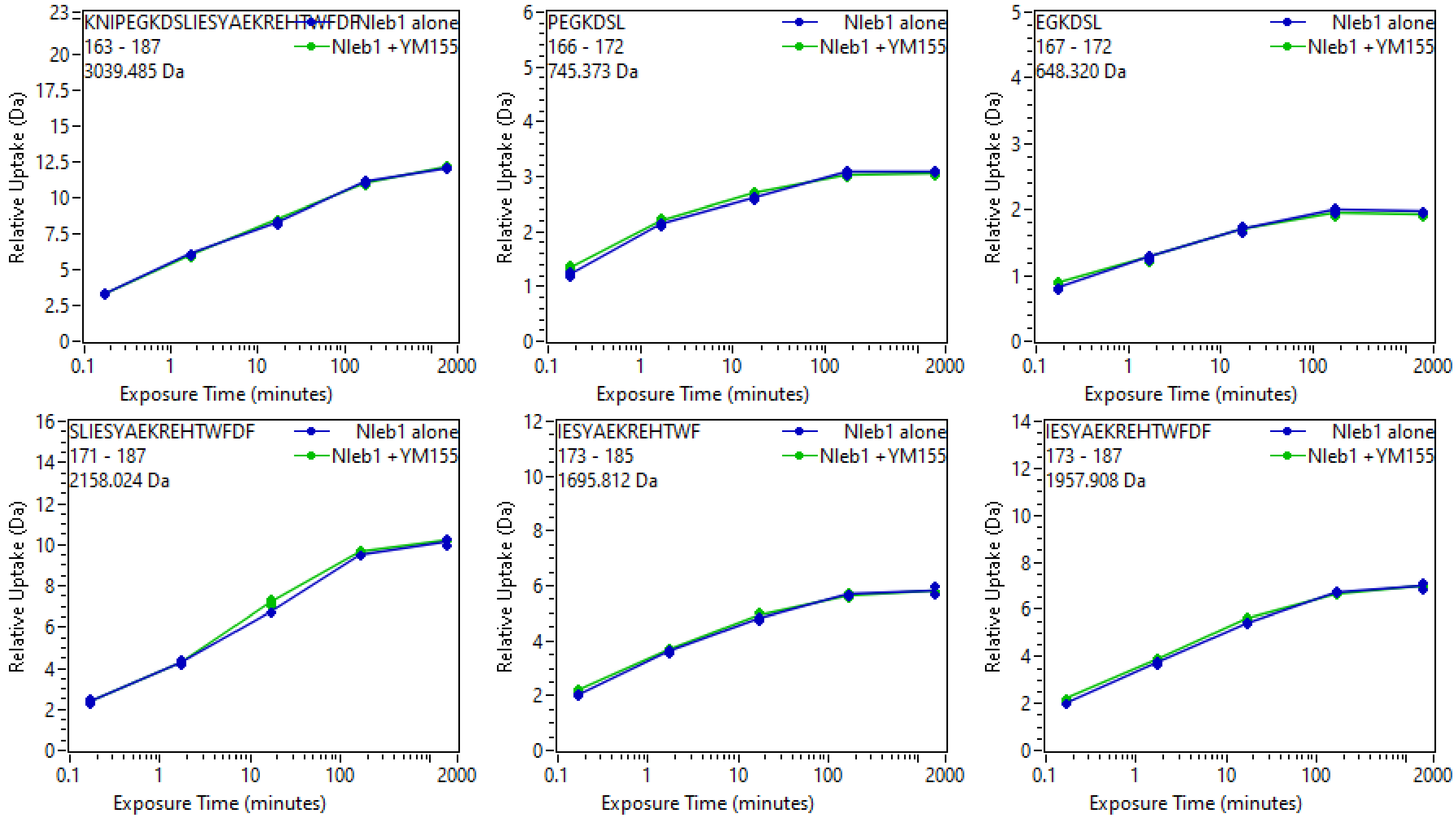
KNIPEGKDSLIESYAEKREHTWFDF
163 - 187
3039.485 Da
Nleb1 alone
Nleb1 +YM155
PEGKDSL
166 - 172
745.373 Da
EGKDSL
167 - 172
648.320 Da
SLIESYAEKREHTWFDF
171 - 187
2158.024 Da
IESYAEKREHTWF
173 - 185
1695.812 Da
IESYAEKREHTWFDF
173 - 187
1957.908 Da
Relative Uptake (Da)
Exposure Time (minutes)
0.1
1
10
100
2000

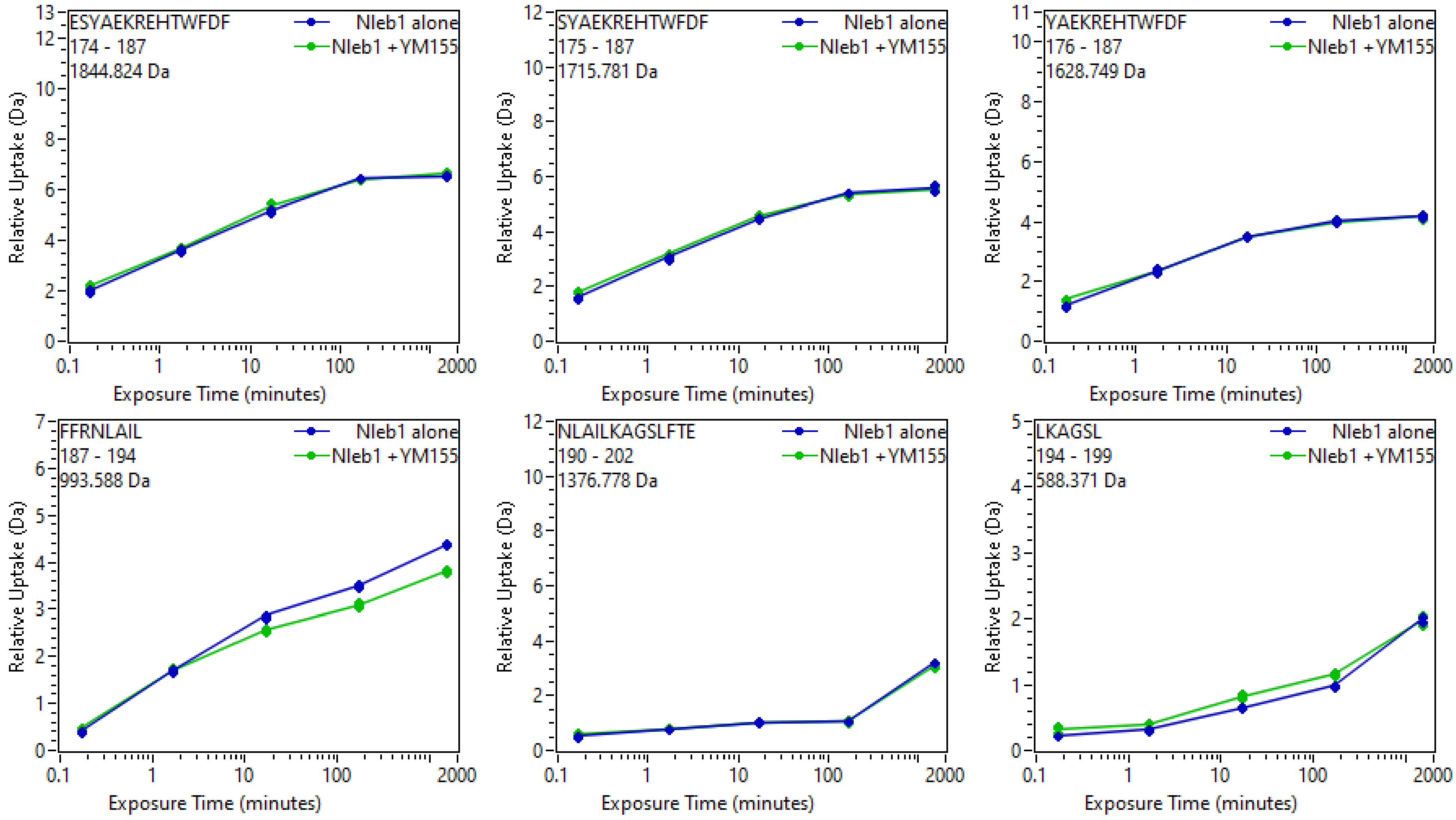
ESYAEKREHTWFDF
174 - 187
1844.824 Da
SYAEKREHTWFDF
175 - 187
1715.781 Da
YAEKREHTWFDF
176 - 187
1628.749 Da
FFRNLAIL
187 - 194
993.588 Da
NLAILKAGSLFTE
190 - 202
1376.778 Da
LKAGSL
194 - 199
588.371 Da
Nleb1 alone
Nleb1 +YM155
Relative Uptake (Da)
Exposure Time (minutes)
0.1
1
10
100
2000

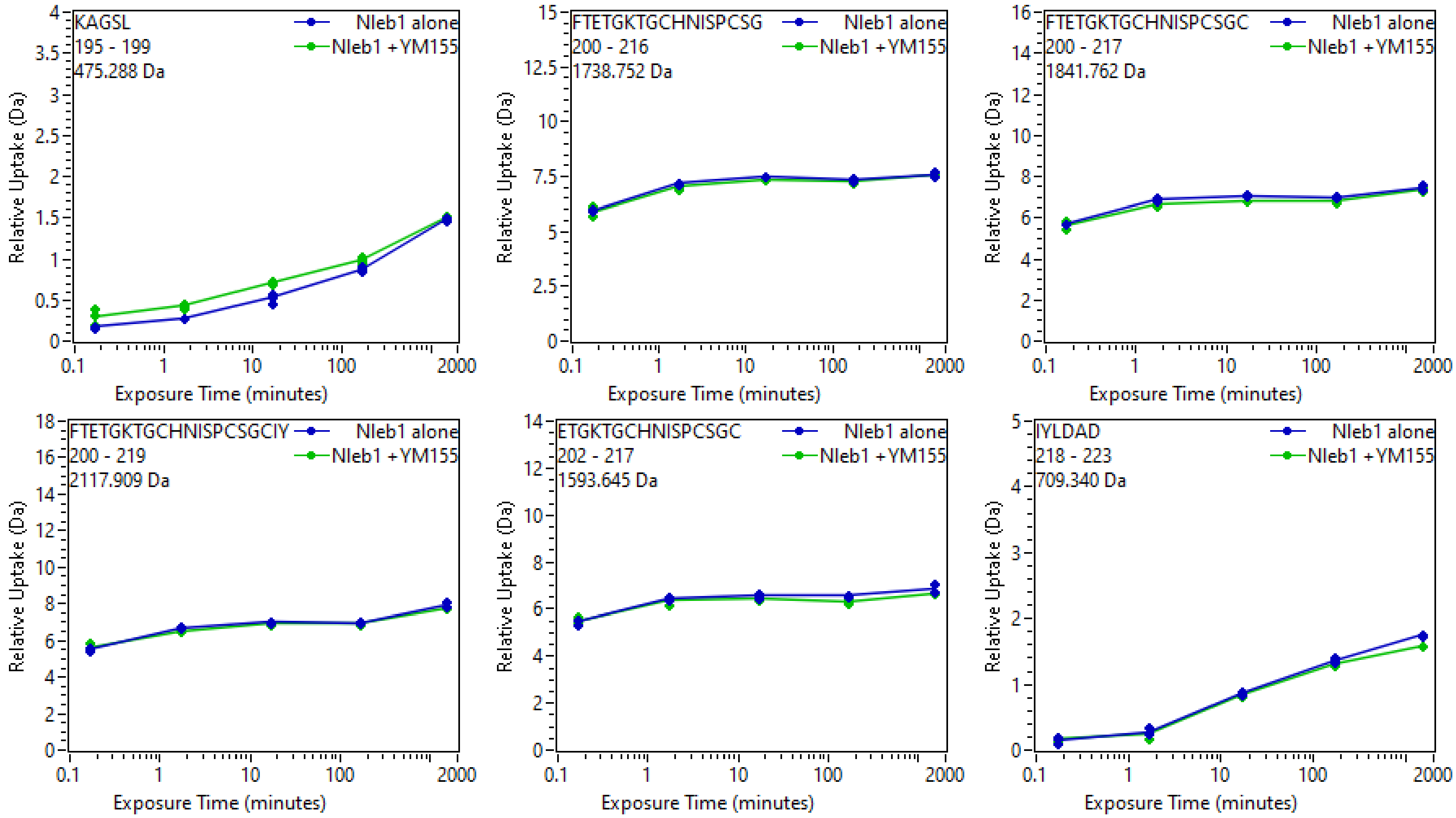

KAGSL
195 - 199
475.288 Da
Nleb1 alone
Nleb1 +YM155
FTETGKTGCHNISPCSG
200 - 216
1738.752 Da
Nleb1 alone
Nleb1 +YM155
FTETGKTGCHNISPCSGC
200 - 217
1841.762 Da
Nleb1 alone
Nleb1 +YM155
FTETGKTGCHNISPCSGCIY
200 - 219
2117.909 Da
Nleb1 alone
Nleb1 +YM155
ETGKTGCHNISPCSGC
202 - 217
1593.645 Da
Nleb1 alone
Nleb1 +YM155
IYLDAD
218 - 223
709.340 Da
Nleb1 alone
Nleb1 +YM155
Relative Uptake (Da)
Exposure Time (minutes)
0.1
1
10
100
2000

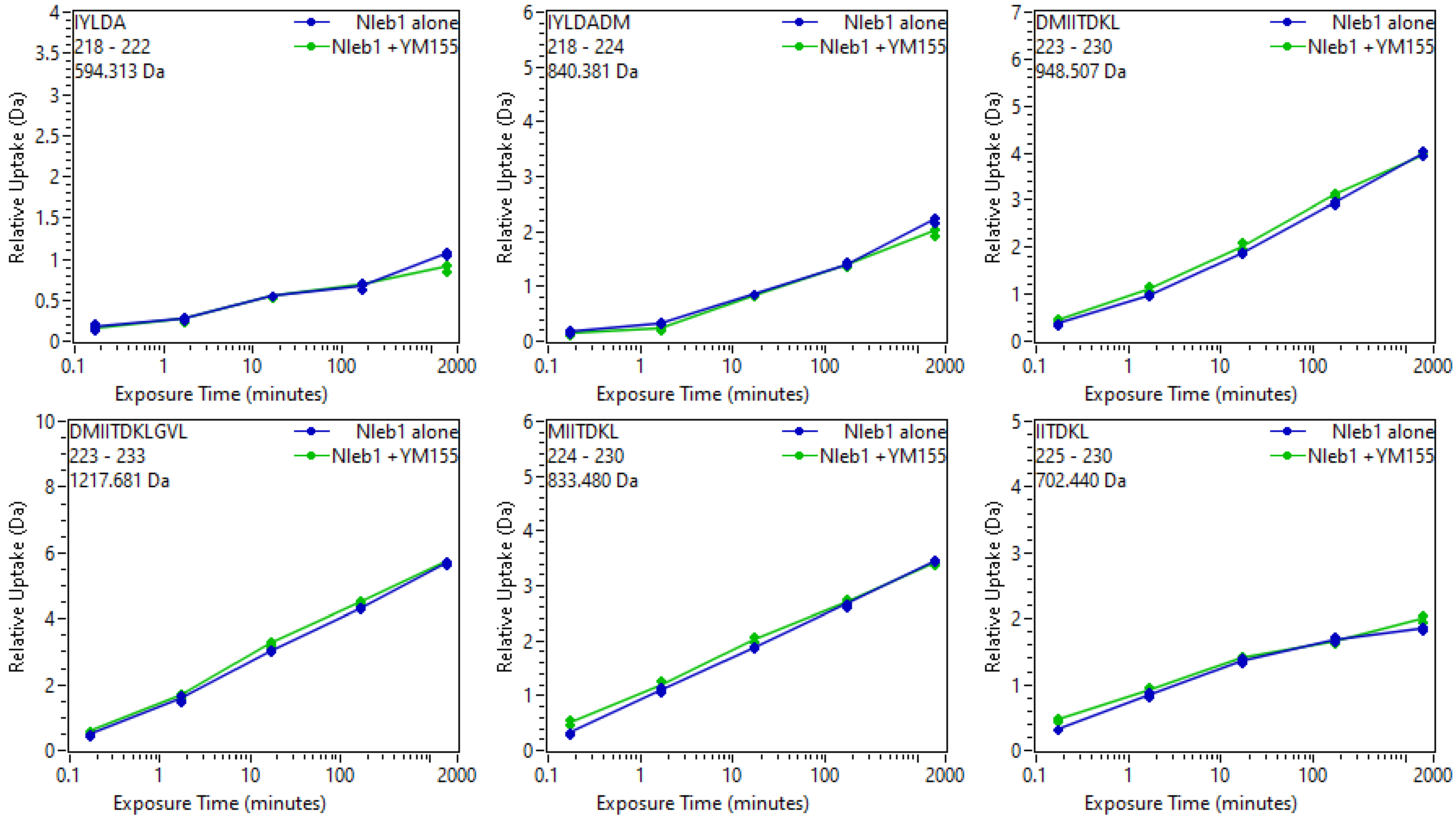
IYLDA
218 - 222
594.313 Da
IYLDADM
218 - 224
840.381 Da
DMIITDKL
223 - 230
948.507 Da
DMIITDKLGVL
223 - 233
1217.681 Da
MIITDKL
224 - 230
833.480 Da
IITDKL
225 - 230
702.440 Da
Nleb1 alone
Nleb1 +YM155
Relative Uptake (Da)
Exposure Time (minutes)
0.1
1
10
100
2000

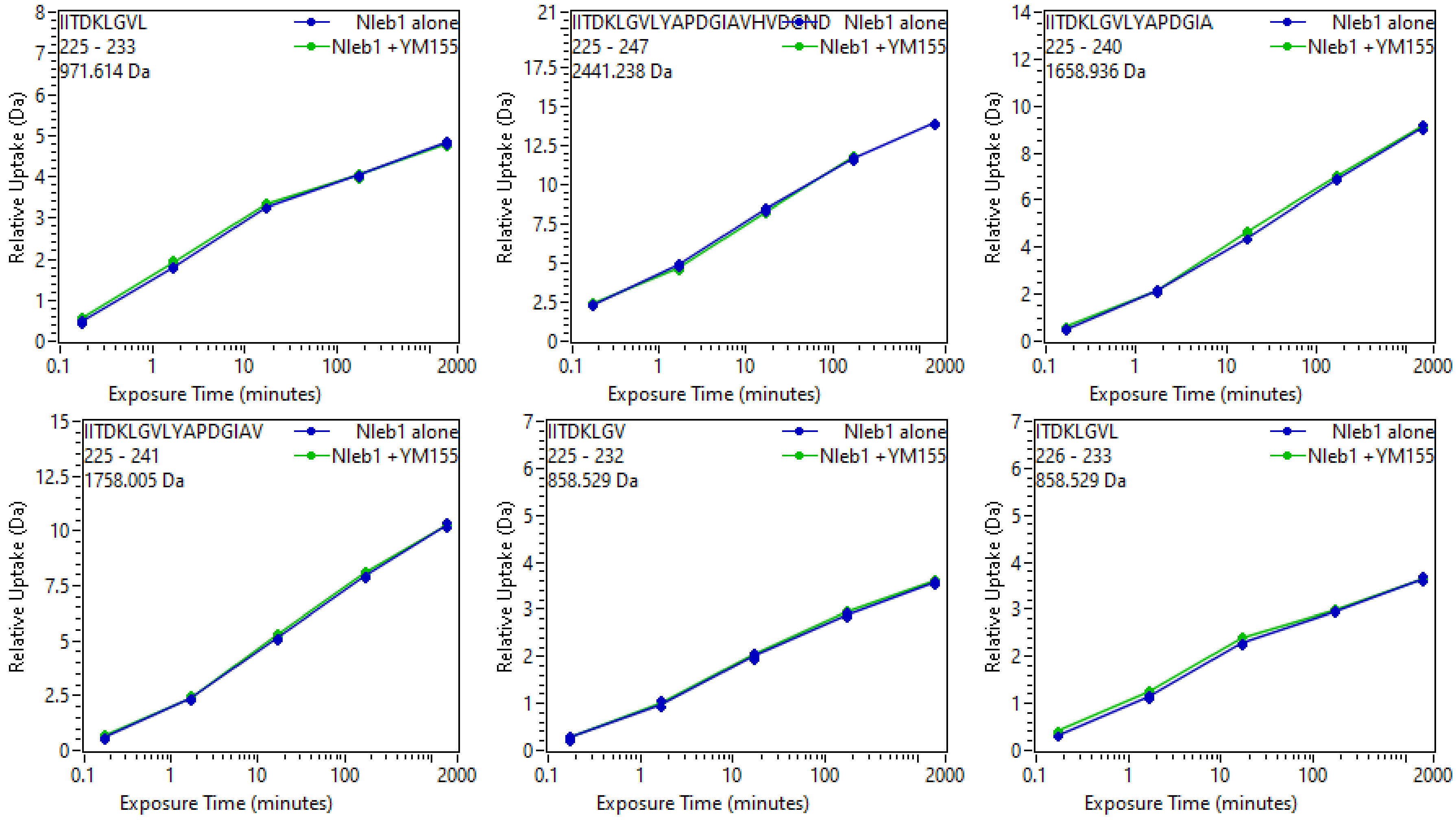
IITDKLGVL
225 - 233
971.614 Da
Nleb1 alone
Nleb1 +YM155
Relative Uptake (Da)
Exposure Time (minutes)
IITDKLGVLYAPDGIAVHVDGND
225 - 247
2441.238 Da
IITDKLGVLYAPDGIA
225 - 240
1658.936 Da
IITDKLGVLYAPDGIAV
225 - 241
1758.005 Da
IITDKLGV
225 - 232
858.529 Da
ITDKLGVL
226 - 233
858.529 Da

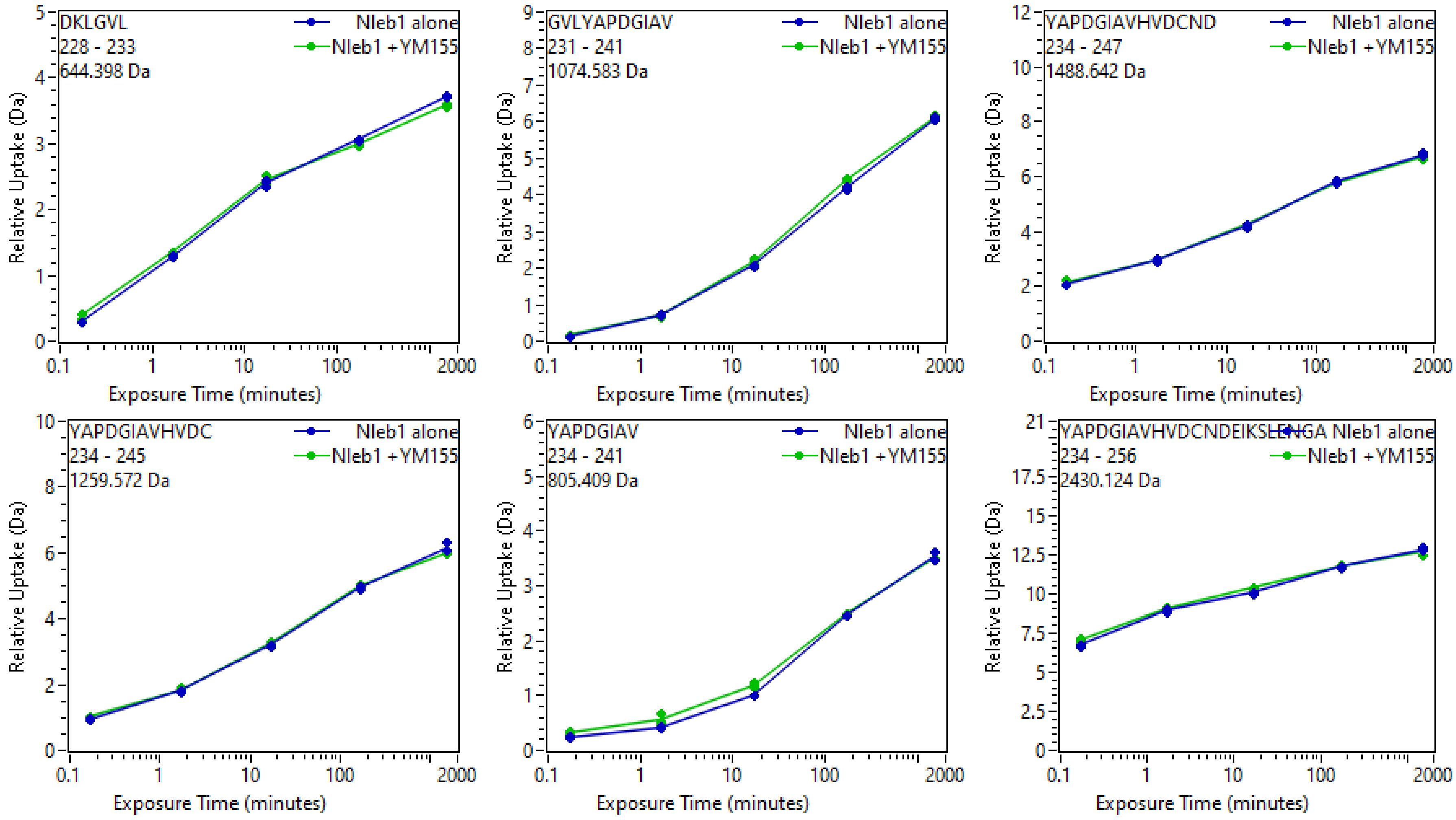
DKLGVL
228 - 233
644.398 Da
Nleb1 alone
Nleb1 +YM155
Relative Uptake (Da)
Exposure Time (minutes)
GVLYAPDGIAV
231 - 241
1074.583 Da
YAPDGIAVHVDCND
234 - 247
1488.642 Da
YAPDGIAVHVDC
234 - 245
1259.572 Da
YAPDGIAV
234 - 241
805.409 Da
YAPDGIAVHVDCNDEIKSLENGA
234 - 256
2430.124 Da

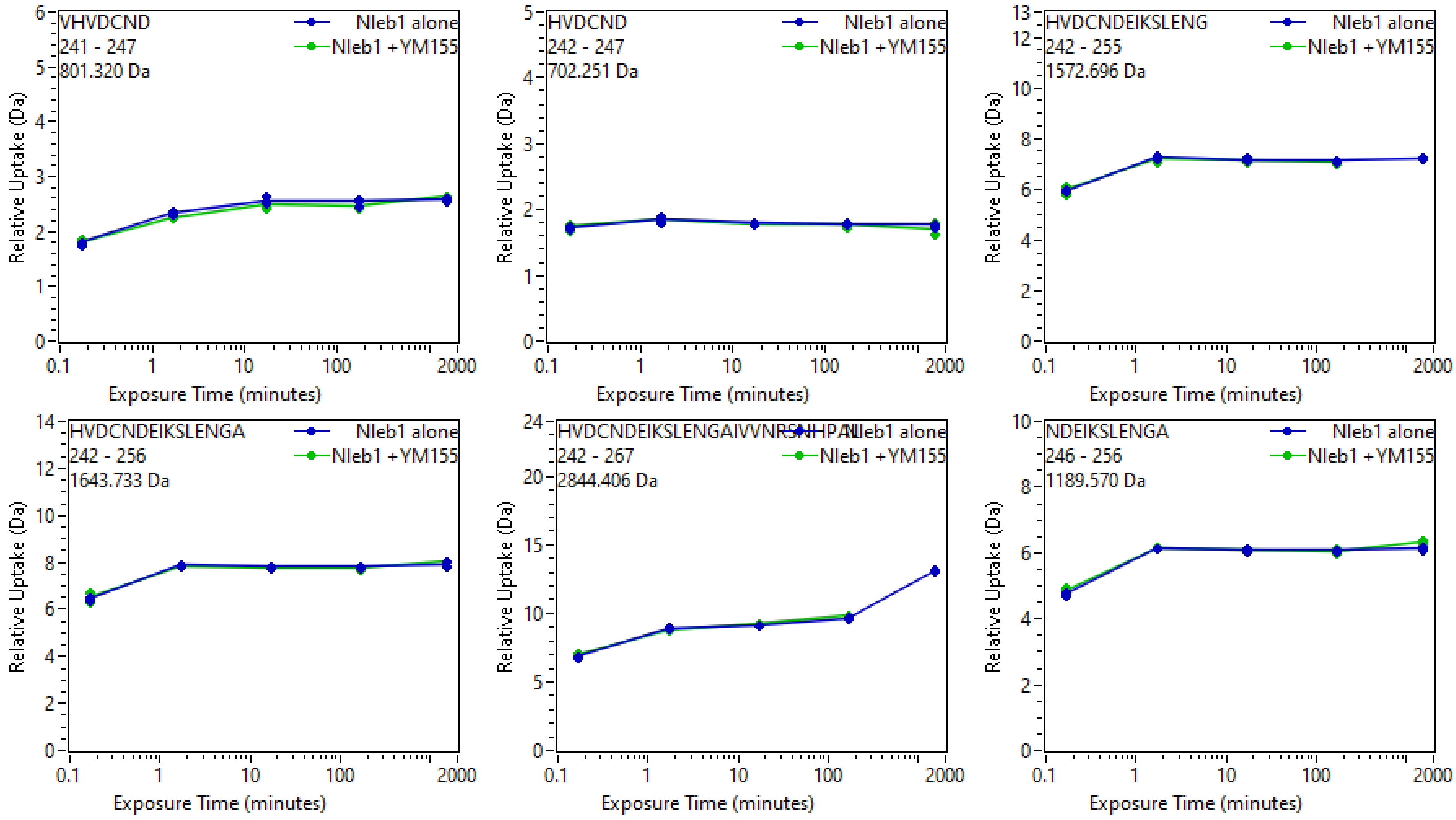
VHVDCND
241 - 247
801.320 Da
Nleb1 alone
Nleb1 +YM155
Relative Uptake (Da)
Exposure Time (minutes)
HVDCND
242 - 247
702.251 Da
HVDCNDEIKSLENG
242 - 255
1572.696 Da
HVDCNDEIKSLENGA
242 - 256
1643.733 Da
HVDCNDEIKSLENGAIVVNRSNHPAI
242 - 267
2844.406 Da
NDEIKSLENGA
246 - 256
1189.570 Da

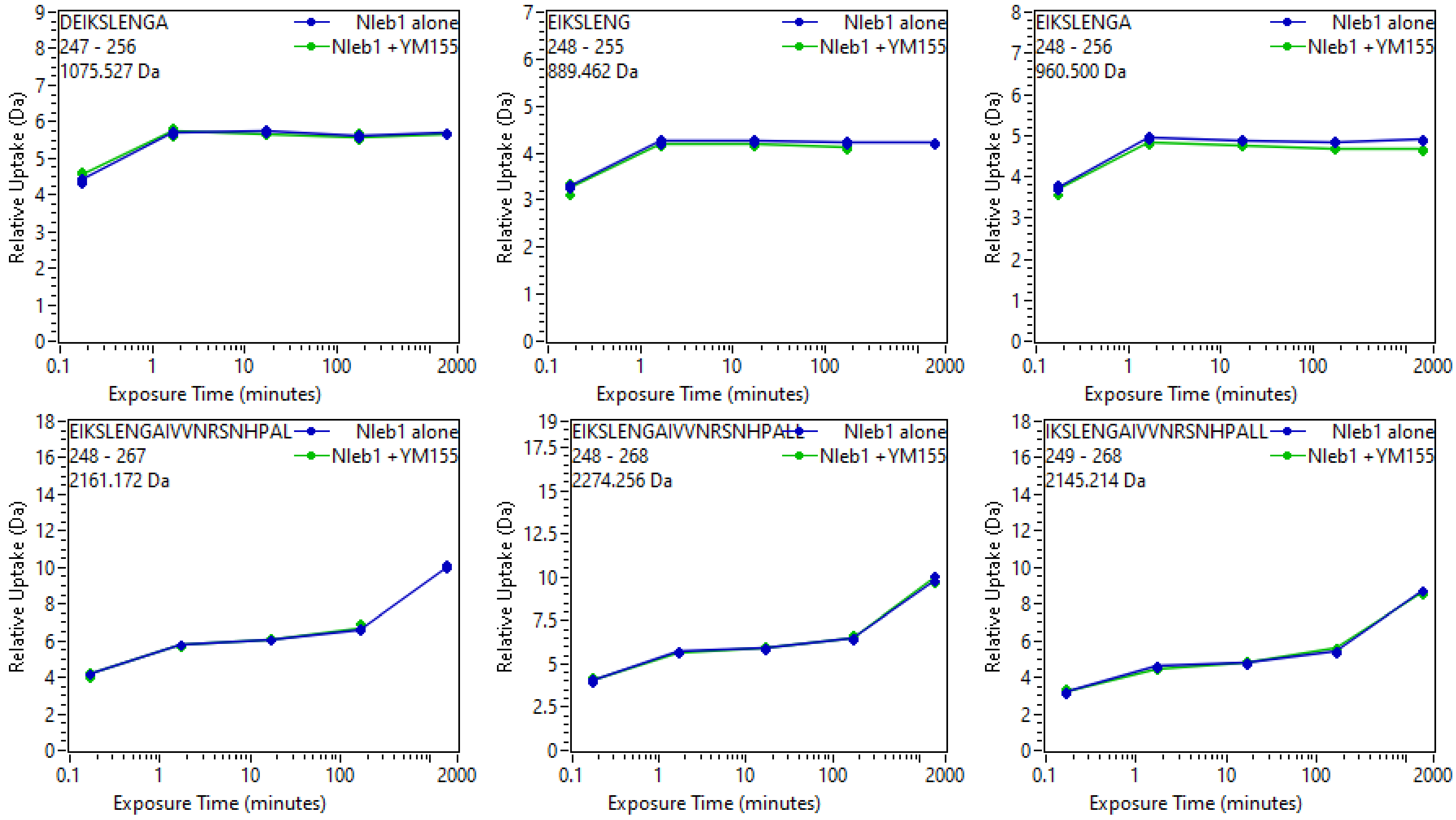
DEIKSLENGA
247 - 256
1075.527 Da
EIKSLENG
248 - 255
889.462 Da
EIKSLENGA
248 - 256
960.500 Da
EIKSLENGAIVVNRSNHPAL
248 - 267
2161.172 Da
EIKSLENGAIVVNRSNHPALL
248 - 268
2274.256 Da
IKSLENGAIVVNRSNHPALL
249 - 268
2145.214 Da
Nleb1 alone
Nleb1 +YM155
Relative Uptake (Da)
Exposure Time (minutes)

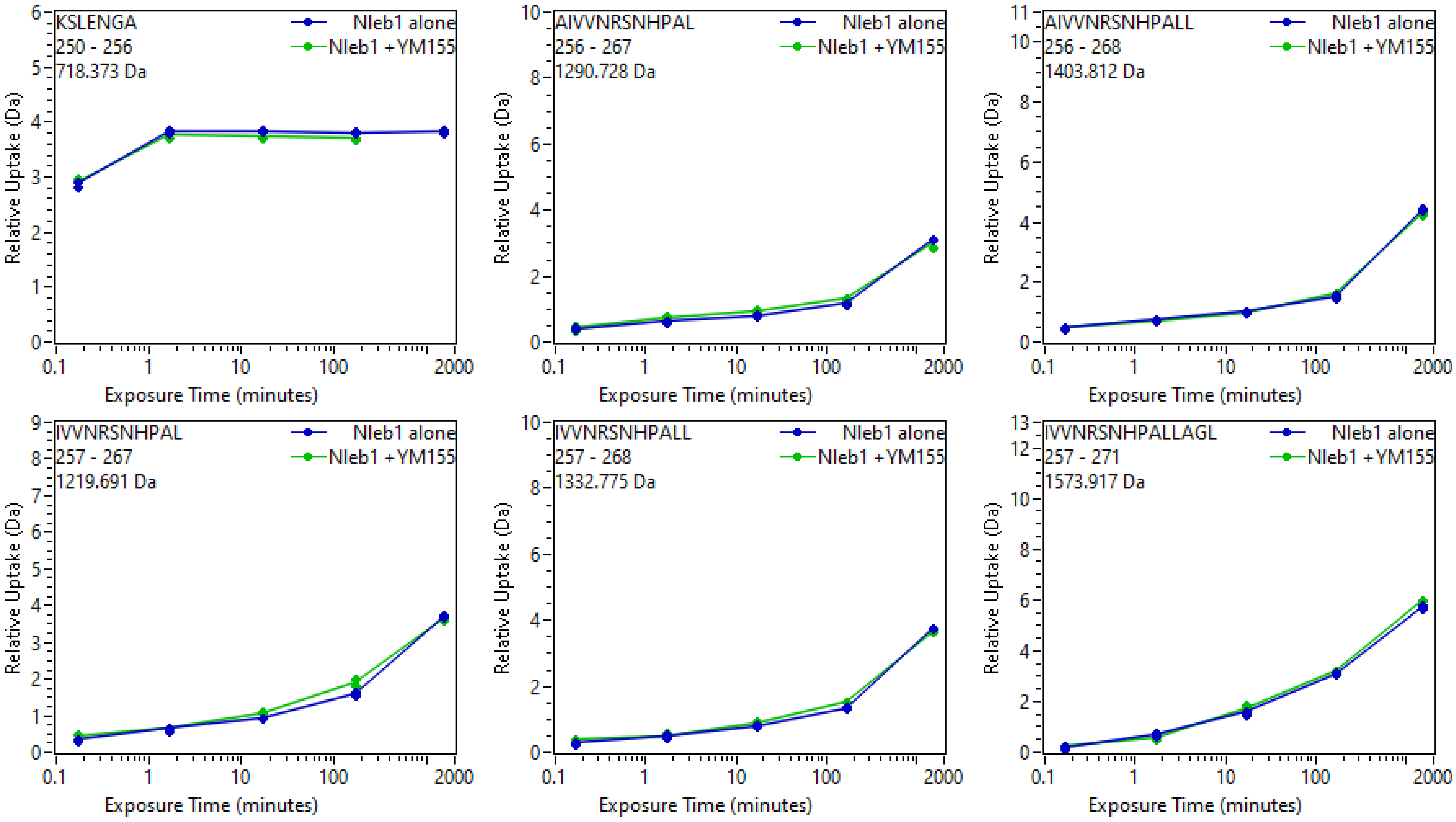
KSLENGA
250 - 256
718.373 Da
AIVVNRSNHPAL
256 - 267
1290.728 Da
AIVVNRSNHPALL
256 - 268
1403.812 Da
IVVNRSNHPAL
257 - 267
1219.691 Da
IVVNRSNHPALL
257 - 268
1332.775 Da
IVVNRSNHPALLAGL
257 - 271
1573.917 Da
Nleb1 alone
Nleb1 +YM155
Relative Uptake (Da)
Exposure Time (minutes)
0.1
1
10
100
2000

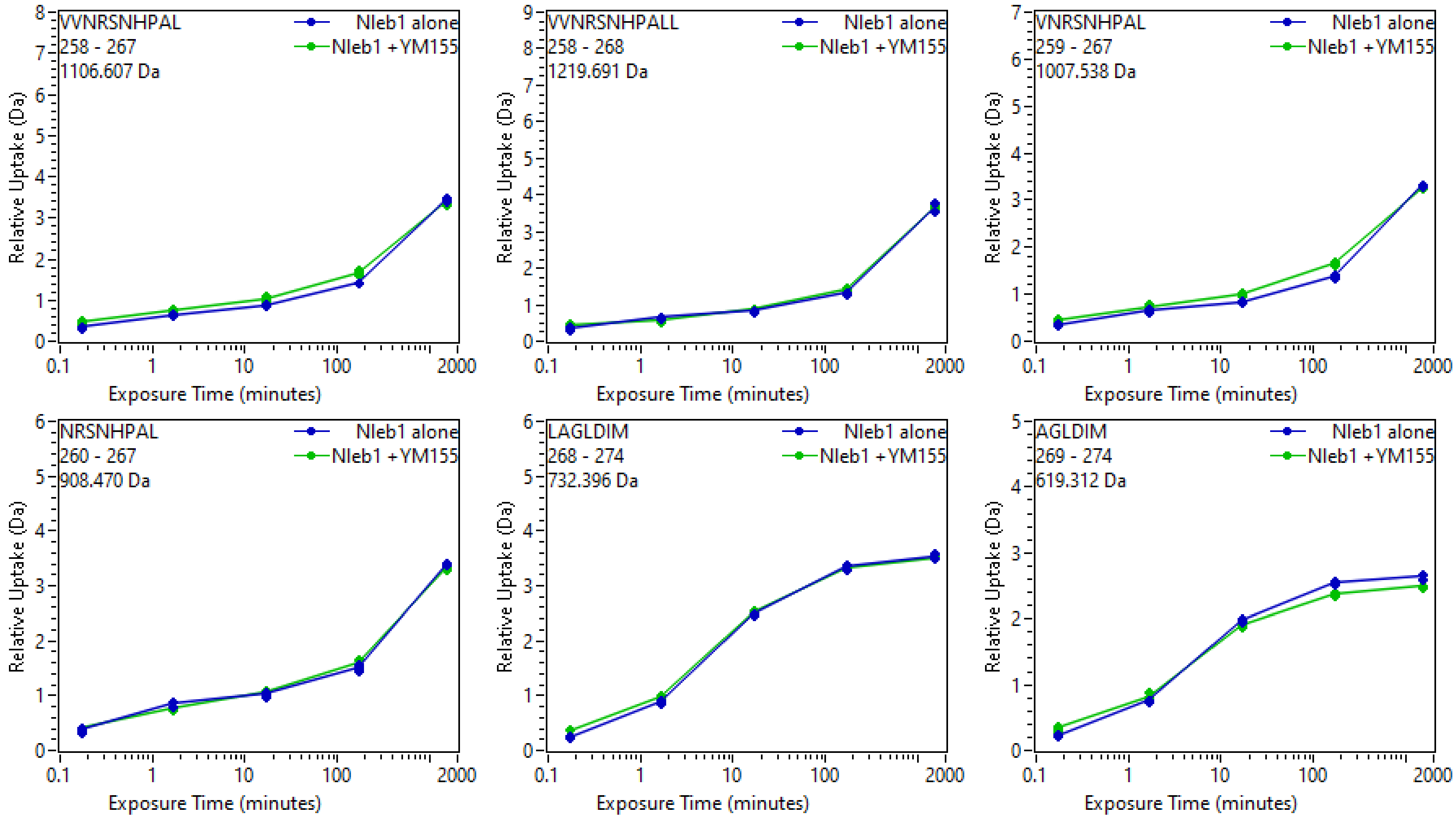
VVNRSNHPAL
258 - 267
1106.607 Da
VVNRSNHPALL
258 - 268
1219.691 Da
VNRSNHPAL
259 - 267
1007.538 Da
NRSNHPAL
260 - 267
908.470 Da
LAGLDIM
268 - 274
732.396 Da
AGLDIM
269 - 274
619.312 Da
Nleb1 alone
Nleb1 +YM155
Relative Uptake (Da)
Exposure Time (minutes)
0.1
1
10
100
2000

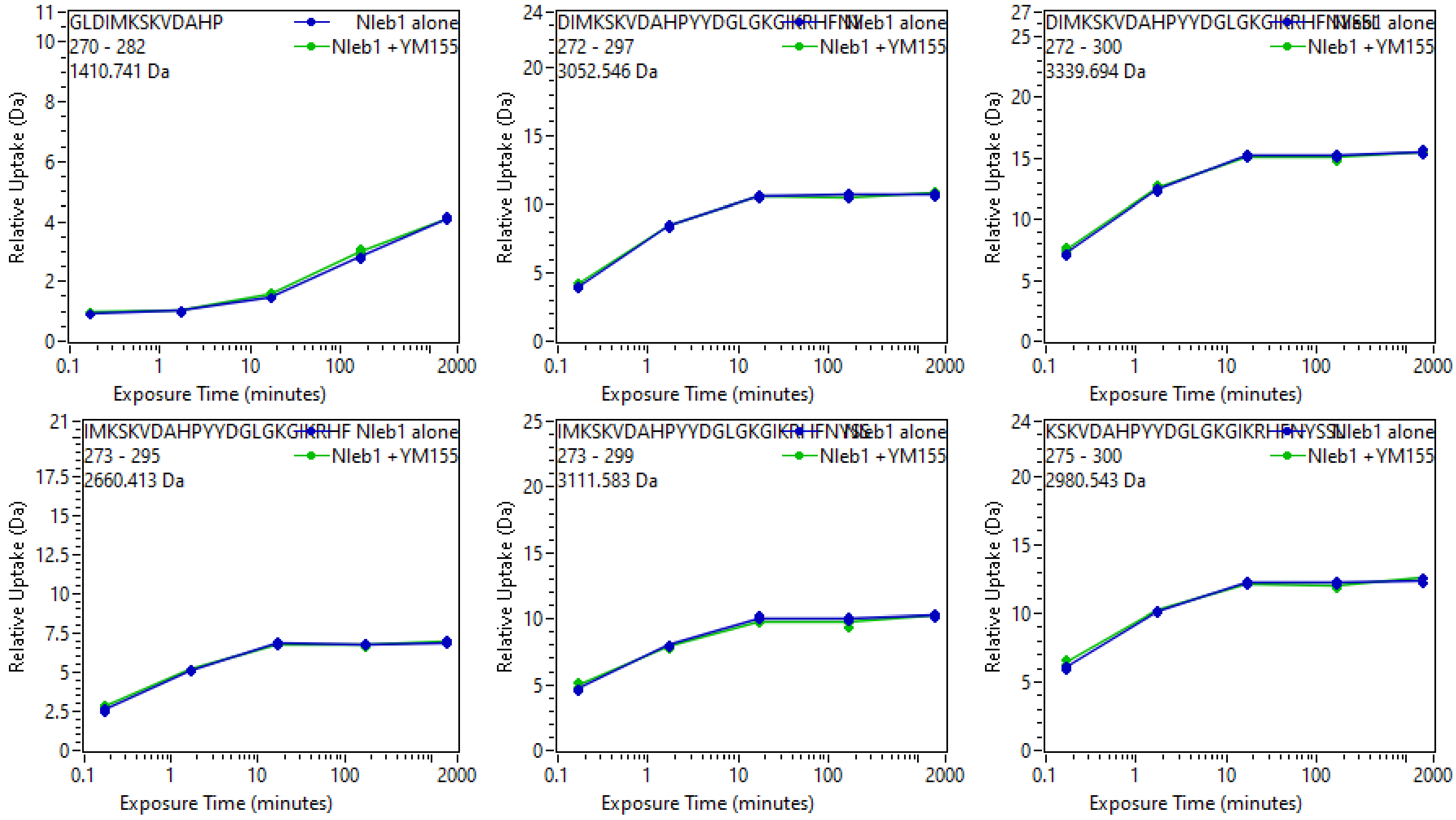
GLDIMKSKVDAHP
270 - 282
1410.741 Da
Nleb1 alone
Nleb1 +YM155
272 - 297
3052.546 Da
272 - 300
3339.694 Da
273 - 295
2660.413 Da
273 - 299
3111.583 Da
275 - 300
2980.543 Da
Relative Uptake (Da)
Exposure Time (minutes)
0.1
1
10
100
2000

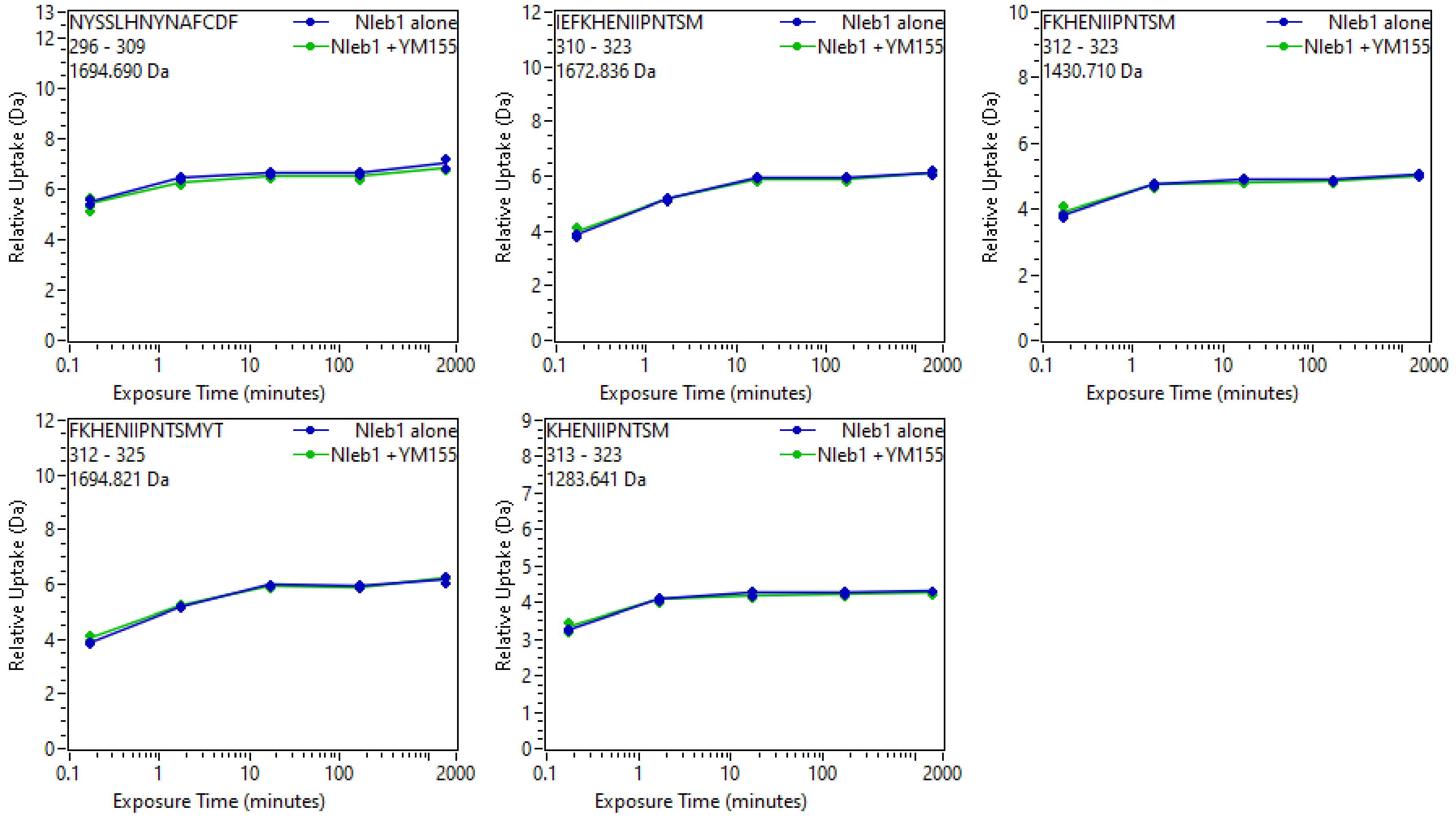

Figure S11. Deuterium uptake plot of each individual peptide whose HDX was followed for the NleB1 HDX-MS analysis.

Protein	Start	End	Sequence	State	Exposure	Uptake (Da)	Uptake SD	RT (min)
SseK2	46	53	AGIDYPLL	SseK2 + YM155	10 s (23 C)	0.39	0.058774	8.13
SseK2	46	53	AGIDYPLL	SseK2 + YM155	100 s (23 C)	0.70	0.00377	8.12
SseK2	46	53	AGIDYPLL	SseK2 + YM155	1,000 s (23 C)	1.54	0.025883	8.12
SseK2	46	53	AGIDYPLL	SseK2 + YM155	10,000 s (23 C)	2.86	0.028063	8.12
SseK2	46	53	AGIDYPLL	SseK2 + YM155	12 h (28 C)	3.37	0.013639	8.11
SseK2	46	53	AGIDYPLL	SseK2 alone	10 s (23 C)	0.37	0.012854	8.13
SseK2	46	53	AGIDYPLL	SseK2 alone	100 s (23 C)	0.77	0.057121	8.12
SseK2	46	53	AGIDYPLL	SseK2 alone	1,000 s (23 C)	1.61	0.158068	8.12
SseK2	46	53	AGIDYPLL	SseK2 alone	10,000 s (23 C)	2.92	0.031382	8.12
SseK2	46	53	AGIDYPLL	SseK2 alone	12 h (28 C)	3.37	0.036065	8.12
SseK2	46	62	AGIDYPLLPLNHQTPLV	SseK2 + YM155	10 s (23 C)	1.50	0.134804	7.63
SseK2	46	62	AGIDYPLLPLNHQTPLV	SseK2 + YM155	100 s (23 C)	1.95	0.044599	7.60
SseK2	46	62	AGIDYPLLPLNHQTPLV	SseK2 + YM155	1,000 s (23 C)	3.00	0.039326	7.62
SseK2	46	62	AGIDYPLLPLNHQTPLV	SseK2 + YM155	10,000 s (23 C)	5.07	0.024563	7.62
SseK2	46	62	AGIDYPLLPLNHQTPLV	SseK2 + YM155	12 h (28 C)	6.50	0.05685	7.61
SseK2	46	62	AGIDYPLLPLNHQTPLV	SseK2 alone	10 s (23 C)	1.46	0.048658	7.62
SseK2	46	62	AGIDYPLLPLNHQTPLV	SseK2 alone	100 s (23 C)	2.04	0.079223	7.62
SseK2	46	62	AGIDYPLLPLNHQTPLV	SseK2 alone	1,000 s (23 C)	3.06	0.136383	7.62
SseK2	46	62	AGIDYPLLPLNHQTPLV	SseK2 alone	10,000 s (23 C)	5.12	0.09776	7.62
SseK2	46	62	AGIDYPLLPLNHQTPLV	SseK2 alone	12 h (28 C)	6.51	0.001771	7.61
SseK2	46	63	AGIDYPLLPLNHQTPLVF	SseK2 + YM155	10 s (23 C)	1.43	0.022655	8.56
SseK2	46	63	AGIDYPLLPLNHQTPLVF	SseK2 + YM155	100 s (23 C)	1.90	0.010508	8.55
SseK2	46	63	AGIDYPLLPLNHQTPLVF	SseK2 + YM155	1,000 s (23 C)	2.99	0.08268	8.55
SseK2	46	63	AGIDYPLLPLNHQTPLVF	SseK2 + YM155	10,000 s (23 C)	5.00	0.030564	8.55
SseK2	46	63	AGIDYPLLPLNHQTPLVF	SseK2 + YM155	12 h (28 C)	6.62	0.013781	8.55
SseK2	46	63	AGIDYPLLPLNHQTPLVF	SseK2 alone	10 s (23 C)	1.46	0.04407	8.55
SseK2	46	63	AGIDYPLLPLNHQTPLVF	SseK2 alone	100 s (23 C)	2.00	0.087136	8.55

SseK2	46	63	AGIDYPLLPLNHQTPLVF	SseK2 alone	1,000 s (23 C)	3.04	0.186941	8.55
SseK2	46	63	AGIDYPLLPLNHQTPLVF	SseK2 alone	10,000 s (23 C)	5.06	0.050118	8.55
SseK2	46	63	AGIDYPLLPLNHQTPLVF	SseK2 alone	12 h (28 C)	6.60	0.050766	8.55
SseK2	46	64	AGIDYPLLPLNHQTPLVFQ	SseK2 + YM155	10 s (23 C)	1.47	0.046	8.00
SseK2	46	64	AGIDYPLLPLNHQTPLVFQ	SseK2 + YM155	100 s (23 C)	2.08	0.029152	8.13
SseK2	46	64	AGIDYPLLPLNHQTPLVFQ	SseK2 + YM155	1,000 s (23 C)	3.23	0.073804	7.99
SseK2	46	64	AGIDYPLLPLNHQTPLVFQ	SseK2 + YM155	10,000 s (23 C)	5.82	0.030967	8.13
SseK2	46	64	AGIDYPLLPLNHQTPLVFQ	SseK2 + YM155	12 h (28 C)	7.66	0.025428	8.13
SseK2	46	64	AGIDYPLLPLNHQTPLVFQ	SseK2 alone	10 s (23 C)	1.52	0.035622	8.16
SseK2	46	64	AGIDYPLLPLNHQTPLVFQ	SseK2 alone	100 s (23 C)	1.99	0.03433	8.14
SseK2	46	64	AGIDYPLLPLNHQTPLVFQ	SseK2 alone	1,000 s (23 C)	3.07	0.018608	7.99
SseK2	46	64	AGIDYPLLPLNHQTPLVFQ	SseK2 alone	10,000 s (23 C)	5.80	0.093273	8.16
SseK2	46	64	AGIDYPLLPLNHQTPLVFQ	SseK2 alone	12 h (28 C)	7.53	0.076237	8.16
SseK2	46	65	AGIDYPLLPLNHQTPLVFQW	SseK2 + YM155	10 s (23 C)	1.33	0.009179	9.02
SseK2	46	65	AGIDYPLLPLNHQTPLVFQW	SseK2 + YM155	100 s (23 C)	1.68	0.007594	9.04
SseK2	46	65	AGIDYPLLPLNHQTPLVFQW	SseK2 + YM155	1,000 s (23 C)	2.73	0.082864	9.04
SseK2	46	65	AGIDYPLLPLNHQTPLVFQW	SseK2 + YM155	10,000 s (23 C)	4.86	0.031141	9.01
SseK2	46	65	AGIDYPLLPLNHQTPLVFQW	SseK2 + YM155	12 h (28 C)	6.57	0.046614	9.02
SseK2	46	65	AGIDYPLLPLNHQTPLVFQW	SseK2 alone	10 s (23 C)	1.32	0.017147	9.03
SseK2	46	65	AGIDYPLLPLNHQTPLVFQW	SseK2 alone	100 s (23 C)	1.76	0.048683	9.02
SseK2	46	65	AGIDYPLLPLNHQTPLVFQW	SseK2 alone	1,000 s (23 C)	2.77	0.139288	9.04
SseK2	46	65	AGIDYPLLPLNHQTPLVFQW	SseK2 alone	10,000 s (23 C)	4.84	0.039914	9.02
SseK2	46	65	AGIDYPLLPLNHQTPLVFQW	SseK2 alone	12 h (28 C)	6.61	0.004134	9.02
SseK2	48	62	IDYPLLPLNHQTPLV	SseK2 + YM155	10 s (23 C)	1.18	0.051074	7.83
SseK2	48	62	IDYPLLPLNHQTPLV	SseK2 + YM155	100 s (23 C)	1.29	0.015507	7.82
SseK2	48	62	IDYPLLPLNHQTPLV	SseK2 + YM155	1,000 s (23 C)	1.93	0.039713	7.82
SseK2	48	62	IDYPLLPLNHQTPLV	SseK2 + YM155	10,000 s (23 C)	3.50	0.04297	7.82
SseK2	48	62	IDYPLLPLNHQTPLV	SseK2 + YM155	12 h (28 C)	5.04	0.024803	7.82

SseK2	48	62	IDYPLLPLNHQTPLV	SseK2 alone	10 s (23 C)	1.16	0.025803	7.83
SseK2	48	62	IDYPLLPLNHQTPLV	SseK2 alone	100 s (23 C)	1.36	0.024924	7.82
SseK2	48	62	IDYPLLPLNHQTPLV	SseK2 alone	1,000 s (23 C)	1.85	0.071944	7.82
SseK2	48	62	IDYPLLPLNHQTPLV	SseK2 alone	10,000 s (23 C)	3.53	0.043734	7.82
SseK2	48	62	IDYPLLPLNHQTPLV	SseK2 alone	12 h (28 C)	5.02	0.04237	7.82
SseK2	48	63	IDYPLLPLNHQTPLVF	SseK2 + YM155	10 s (23 C)	1.30	0.12303	8.56
SseK2	48	63	IDYPLLPLNHQTPLVF	SseK2 + YM155	100 s (23 C)	1.42	0.045341	8.55
SseK2	48	63	IDYPLLPLNHQTPLVF	SseK2 + YM155	1,000 s (23 C)	2.08	0.096155	8.55
SseK2	48	63	IDYPLLPLNHQTPLVF	SseK2 + YM155	10,000 s (23 C)	3.68	0.014147	8.55
SseK2	48	63	IDYPLLPLNHQTPLVF	SseK2 + YM155	12 h (28 C)	5.50	0.040614	8.55
SseK2	48	63	IDYPLLPLNHQTPLVF	SseK2 alone	10 s (23 C)	1.28	0.037538	8.56
SseK2	48	63	IDYPLLPLNHQTPLVF	SseK2 alone	100 s (23 C)	1.48	0.024599	8.55
SseK2	48	63	IDYPLLPLNHQTPLVF	SseK2 alone	1,000 s (23 C)	2.05	0.09218	8.55
SseK2	48	63	IDYPLLPLNHQTPLVF	SseK2 alone	10,000 s (23 C)	3.71	0.022643	8.55
SseK2	48	63	IDYPLLPLNHQTPLVF	SseK2 alone	12 h (28 C)	5.38	0.064011	8.55
SseK2	48	64	IDYPLLPLNHQTPLVFQ	SseK2 + YM155	10 s (23 C)	1.41	0.161948	8.16
SseK2	48	64	IDYPLLPLNHQTPLVFQ	SseK2 + YM155	100 s (23 C)	1.37	0.041704	8.15
SseK2	48	64	IDYPLLPLNHQTPLVFQ	SseK2 + YM155	1,000 s (23 C)	2.13	0.053482	8.15
SseK2	48	64	IDYPLLPLNHQTPLVFQ	SseK2 + YM155	10,000 s (23 C)	4.24	0.045117	8.15
SseK2	48	64	IDYPLLPLNHQTPLVFQ	SseK2 + YM155	12 h (28 C)	6.16	0.007146	8.14
SseK2	48	64	IDYPLLPLNHQTPLVFQ	SseK2 alone	10 s (23 C)	1.27	0.028538	8.15
SseK2	48	64	IDYPLLPLNHQTPLVFQ	SseK2 alone	100 s (23 C)	1.38	0.032911	8.15
SseK2	48	64	IDYPLLPLNHQTPLVFQ	SseK2 alone	1,000 s (23 C)	2.14	0.064941	8.15
SseK2	48	64	IDYPLLPLNHQTPLVFQ	SseK2 alone	10,000 s (23 C)	4.30	0.058279	8.15
SseK2	48	64	IDYPLLPLNHQTPLVFQ	SseK2 alone	12 h (28 C)	6.18	0.004183	8.14
SseK2	64	76	QWFERNPDRFGQN	SseK2 + YM155	10 s (23 C)	3.50	0.117382	5.85
SseK2	64	76	QWFERNPDRFGQN	SseK2 + YM155	100 s (23 C)	4.90	0.017944	5.84
SseK2	64	76	QWFERNPDRFGQN	SseK2 + YM155	1,000 s (23 C)	5.96	0.037038	5.83

SseK2	64	76	QWFERNPDRFGQN	SseK2 + YM155	10,000 s (23 C)	6.15	0.042309	5.83
SseK2	64	76	QWFERNPDRFGQN	SseK2 + YM155	12 h (28 C)	6.52	0.076793	5.83
SseK2	64	76	QWFERNPDRFGQN	SseK2 alone	10 s (23 C)	3.42	0.056719	5.89
SseK2	64	76	QWFERNPDRFGQN	SseK2 alone	100 s (23 C)	4.92	0.102542	5.88
SseK2	64	76	QWFERNPDRFGQN	SseK2 alone	1,000 s (23 C)	5.97	0.027796	5.89
SseK2	64	76	QWFERNPDRFGQN	SseK2 alone	10,000 s (23 C)	6.22	0.039591	5.89
SseK2	64	76	QWFERNPDRFGQN	SseK2 alone	12 h (28 C)	6.58	0.141941	5.88
SseK2	64	83	QWFERNPDRFGQNEIPIINT	SseK2 + YM155	10 s (23 C)	3.59	0.013395	6.96
SseK2	64	83	QWFERNPDRFGQNEIPIINT	SseK2 + YM155	100 s (23 C)	7.05	0.055473	6.94
SseK2	64	83	QWFERNPDRFGQNEIPIINT	SseK2 + YM155	1,000 s (23 C)	9.67	0.020574	6.94
SseK2	64	83	QWFERNPDRFGQNEIPIINT	SseK2 + YM155	10,000 s (23 C)	10.44	0.022452	6.94
SseK2	64	83	QWFERNPDRFGQNEIPIINT	SseK2 + YM155	12 h (28 C)	10.79	0.15768	6.94
SseK2	64	83	QWFERNPDRFGQNEIPIINT	SseK2 alone	10 s (23 C)	3.47	0.08903	6.97
SseK2	64	83	QWFERNPDRFGQNEIPIINT	SseK2 alone	100 s (23 C)	6.68	0.211604	6.96
SseK2	64	83	QWFERNPDRFGQNEIPIINT	SseK2 alone	1,000 s (23 C)	9.28	0.129286	6.96
SseK2	64	83	QWFERNPDRFGQNEIPIINT	SseK2 alone	10,000 s (23 C)	10.45	0.0466	6.96
SseK2	64	83	QWFERNPDRFGQNEIPIINT	SseK2 alone	12 h (28 C)	10.85	0.12274	6.95
SseK2	65	76	WFERNPDRFGQN	SseK2 + YM155	10 s (23 C)	3.40	0.142071	5.58
SseK2	65	76	WFERNPDRFGQN	SseK2 + YM155	100 s (23 C)	4.57	0.028032	5.55
SseK2	65	76	WFERNPDRFGQN	SseK2 + YM155	1,000 s (23 C)	5.12	0.022792	5.55
SseK2	65	76	WFERNPDRFGQN	SseK2 + YM155	10,000 s (23 C)	5.27	0.029394	5.56
SseK2	65	76	WFERNPDRFGQN	SseK2 + YM155	12 h (28 C)	5.54	0.11395	5.56
SseK2	65	76	WFERNPDRFGQN	SseK2 alone	10 s (23 C)	3.33	0.09159	5.61
SseK2	65	76	WFERNPDRFGQN	SseK2 alone	100 s (23 C)	4.57	0.052938	5.60
SseK2	65	76	WFERNPDRFGQN	SseK2 alone	1,000 s (23 C)	5.16	0.023869	5.60
SseK2	65	76	WFERNPDRFGQN	SseK2 alone	10,000 s (23 C)	5.36	0.02444	5.60
SseK2	65	76	WFERNPDRFGQN	SseK2 alone	12 h (28 C)	5.68	0.082878	5.59
SseK2	65	83	WFERNPDRFGQNEIPIINT	SseK2 + YM155	10 s (23 C)	3.52	0.02328	6.87

SseK2	65	83	WFERNPDRFGQNEIPIINT	SseK2 + YM155	100 s (23 C)	6.68	0.022526	6.85
SseK2	65	83	WFERNPDRFGQNEIPIINT	SseK2 + YM155	1,000 s (23 C)	8.83	0.014634	6.85
SseK2	65	83	WFERNPDRFGQNEIPIINT	SseK2 + YM155	10,000 s (23 C)	9.50	0.069376	6.85
SseK2	65	83	WFERNPDRFGQNEIPIINT	SseK2 + YM155	12 h (28 C)	9.74	0.124489	6.85
SseK2	65	83	WFERNPDRFGQNEIPIINT	SseK2 alone	10 s (23 C)	3.38	0.074172	6.89
SseK2	65	83	WFERNPDRFGQNEIPIINT	SseK2 alone	100 s (23 C)	6.27	0.169788	6.88
SseK2	65	83	WFERNPDRFGQNEIPIINT	SseK2 alone	1,000 s (23 C)	8.52	0.101836	6.88
SseK2	65	83	WFERNPDRFGQNEIPIINT	SseK2 alone	10,000 s (23 C)	9.57	0.044771	6.88
SseK2	65	83	WFERNPDRFGQNEIPIINT	SseK2 alone	12 h (28 C)	9.90	0.112917	6.87
SseK2	65	91	WFERNPDRFGQNEIPIINTQKNPYLNN	SseK2 + YM155	10 s (23 C)	5.53	0.015806	6.60
SseK2	65	91	WFERNPDRFGQNEIPIINTQKNPYLNN	SseK2 + YM155	100 s (23 C)	9.35	0.050014	6.59
SseK2	65	91	WFERNPDRFGQNEIPIINTQKNPYLNN	SseK2 + YM155	1,000 s (23 C)	12.27	0.027777	6.58
SseK2	65	91	WFERNPDRFGQNEIPIINTQKNPYLNN	SseK2 + YM155	10,000 s (23 C)	14.92	0.095124	6.58
SseK2	65	91	WFERNPDRFGQNEIPIINTQKNPYLNN	SseK2 + YM155	12 h (28 C)	15.62	0.170281	6.58
SseK2	65	91	WFERNPDRFGQNEIPIINTQKNPYLNN	SseK2 alone	10 s (23 C)	5.36	0.076166	6.67
SseK2	65	91	WFERNPDRFGQNEIPIINTQKNPYLNN	SseK2 alone	100 s (23 C)	8.86	0.160092	6.66
SseK2	65	91	WFERNPDRFGQNEIPIINTQKNPYLNN	SseK2 alone	1,000 s (23 C)	11.84	0.189234	6.66
SseK2	65	91	WFERNPDRFGQNEIPIINTQKNPYLNN	SseK2 alone	10,000 s (23 C)	15.04	0.122848	6.66
SseK2	65	91	WFERNPDRFGQNEIPIINTQKNPYLNN	SseK2 alone	12 h (28 C)	15.78	0.153987	6.65
SseK2	66	76	FERNPDRFGQN	SseK2 + YM155	10 s (23 C)	3.45	0.11235	4.23
SseK2	66	76	FERNPDRFGQN	SseK2 + YM155	100 s (23 C)	4.61	0.032484	4.21
SseK2	66	76	FERNPDRFGQN	SseK2 + YM155	1,000 s (23 C)	5.24	0.047501	4.21
SseK2	66	76	FERNPDRFGQN	SseK2 + YM155	10,000 s (23 C)	5.16	0.062073	4.22
SseK2	66	76	FERNPDRFGQN	SseK2 + YM155	12 h (28 C)	5.17	0.010364	4.21
SseK2	66	76	FERNPDRFGQN	SseK2 alone	10 s (23 C)	3.39	0.097888	4.22
SseK2	66	76	FERNPDRFGQN	SseK2 alone	100 s (23 C)	4.60	0.081551	4.21
SseK2	66	76	FERNPDRFGQN	SseK2 alone	1,000 s (23 C)	5.25	0.013996	4.21
SseK2	66	76	FERNPDRFGQN	SseK2 alone	10,000 s (23 C)	5.22	0.036732	4.21

SseK2	66	76	FERNPDRFGQN	SseK2 alone	12 h (28 C)	5.29	0.009945	4.20
SseK2	66	83	FERNPDRFGQNEIPIINT	SseK2 + YM155	10 s (23 C)	3.51	0.029639	6.26
SseK2	66	83	FERNPDRFGQNEIPIINT	SseK2 + YM155	100 s (23 C)	6.71	0.012162	6.25
SseK2	66	83	FERNPDRFGQNEIPIINT	SseK2 + YM155	1,000 s (23 C)	8.89	0.041015	6.23
SseK2	66	83	FERNPDRFGQNEIPIINT	SseK2 + YM155	10,000 s (23 C)	9.32	0.060978	6.23
SseK2	66	83	FERNPDRFGQNEIPIINT	SseK2 + YM155	12 h (28 C)	9.36	0.041063	6.23
SseK2	66	83	FERNPDRFGQNEIPIINT	SseK2 alone	10 s (23 C)	3.34	0.079852	6.31
SseK2	66	83	FERNPDRFGQNEIPIINT	SseK2 alone	100 s (23 C)	6.21	0.202409	6.31
SseK2	66	83	FERNPDRFGQNEIPIINT	SseK2 alone	1,000 s (23 C)	8.42	0.114978	6.30
SseK2	66	83	FERNPDRFGQNEIPIINT	SseK2 alone	10,000 s (23 C)	9.35	0.054593	6.30
SseK2	66	83	FERNPDRFGQNEIPIINT	SseK2 alone	12 h (28 C)	9.36	0.109794	6.30
SseK2	66	95	FERNPDRFGQNEIPIINTQKNPYLNNIINA	SseK2 + YM155	10 s (23 C)	5.09	0.015443	7.13
SseK2	66	95	FERNPDRFGQNEIPIINTQKNPYLNNIINA	SseK2 + YM155	100 s (23 C)	8.86	0.031632	7.11
SseK2	66	95	FERNPDRFGQNEIPIINTQKNPYLNNIINA	SseK2 + YM155	1,000 s (23 C)	11.85	0.064434	7.10
SseK2	66	95	FERNPDRFGQNEIPIINTQKNPYLNNIINA	SseK2 + YM155	10,000 s (23 C)	14.51	0.069772	7.09
SseK2	66	95	FERNPDRFGQNEIPIINTQKNPYLNNIINA	SseK2 + YM155	12 h (28 C)	15.78	0.064964	7.08
SseK2	66	95	FERNPDRFGQNEIPIINTQKNPYLNNIINA	SseK2 alone	10 s (23 C)	4.95	0.068945	7.11
SseK2	66	95	FERNPDRFGQNEIPIINTQKNPYLNNIINA	SseK2 alone	100 s (23 C)	8.40	0.163192	7.10
SseK2	66	95	FERNPDRFGQNEIPIINTQKNPYLNNIINA	SseK2 alone	1,000 s (23 C)	11.40	0.169089	7.10
SseK2	66	95	FERNPDRFGQNEIPIINTQKNPYLNNIINA	SseK2 alone	10,000 s (23 C)	14.61	0.112194	7.09
SseK2	66	95	FERNPDRFGQNEIPIINTQKNPYLNNIINA	SseK2 alone	12 h (28 C)	15.88	0.113068	7.08
SseK2	67	76	ERNPDRFGQN	SseK2 + YM155	10 s (23 C)	2.80	0.133901	3.60
SseK2	67	76	ERNPDRFGQN	SseK2 + YM155	100 s (23 C)	3.92	0.035762	3.59
SseK2	67	76	ERNPDRFGQN	SseK2 + YM155	1,000 s (23 C)	4.48	0.07254	3.59
SseK2	67	76	ERNPDRFGQN	SseK2 + YM155	10,000 s (23 C)	4.46	0.077306	3.60
SseK2	67	76	ERNPDRFGQN	SseK2 + YM155	12 h (28 C)	4.45	0.007087	3.60
SseK2	67	76	ERNPDRFGQN	SseK2 alone	10 s (23 C)	2.80	0.027401	3.61
SseK2	67	76	ERNPDRFGQN	SseK2 alone	100 s (23 C)	3.98	0.039906	3.59

SseK2	67	76	ERNPDRFGQN	SseK2 alone	1,000 s (23 C)	4.59	0.045378	3.59
SseK2	67	76	ERNPDRFGQN	SseK2 alone	10,000 s (23 C)	4.66	0.015154	3.57
SseK2	67	76	ERNPDRFGQN	SseK2 alone	12 h (28 C)	4.61	0.023625	3.59
SseK2	67	83	ERNPDRFGQNEIPIINT	SseK2 + YM155	10 s (23 C)	2.62	0.000516	6.15
SseK2	67	83	ERNPDRFGQNEIPIINT	SseK2 + YM155	100 s (23 C)	5.82	0.030708	6.12
SseK2	67	83	ERNPDRFGQNEIPIINT	SseK2 + YM155	1,000 s (23 C)	7.97	0.007941	6.12
SseK2	67	83	ERNPDRFGQNEIPIINT	SseK2 + YM155	10,000 s (23 C)	8.45	0.007938	6.11
SseK2	67	83	ERNPDRFGQNEIPIINT	SseK2 + YM155	12 h (28 C)	8.50	0.076875	6.12
SseK2	67	83	ERNPDRFGQNEIPIINT	SseK2 alone	10 s (23 C)	2.53	0.099013	6.18
SseK2	67	83	ERNPDRFGQNEIPIINT	SseK2 alone	100 s (23 C)	5.41	0.164898	6.17
SseK2	67	83	ERNPDRFGQNEIPIINT	SseK2 alone	1,000 s (23 C)	7.64	0.106906	6.16
SseK2	67	83	ERNPDRFGQNEIPIINT	SseK2 alone	10,000 s (23 C)	8.52	0.06301	6.16
SseK2	67	83	ERNPDRFGQNEIPIINT	SseK2 alone	12 h (28 C)	8.58	0.043967	6.16
SseK2	67	95	ERNPDRFGQNEIPIINTQKNPYLNNIINA	SseK2 + YM155	10 s (23 C)	4.34	0.023897	7.07
SseK2	67	95	ERNPDRFGQNEIPIINTQKNPYLNNIINA	SseK2 + YM155	100 s (23 C)	8.20	0.030193	7.05
SseK2	67	95	ERNPDRFGQNEIPIINTQKNPYLNNIINA	SseK2 + YM155	1,000 s (23 C)	11.19	0.058556	7.05
SseK2	67	95	ERNPDRFGQNEIPIINTQKNPYLNNIINA	SseK2 + YM155	10,000 s (23 C)	13.81	0.037004	7.04
SseK2	67	95	ERNPDRFGQNEIPIINTQKNPYLNNIINA	SseK2 + YM155	12 h (28 C)	15.10	0.009175	7.03
SseK2	67	95	ERNPDRFGQNEIPIINTQKNPYLNNIINA	SseK2 alone	10 s (23 C)	4.24	0.095101	7.07
SseK2	67	95	ERNPDRFGQNEIPIINTQKNPYLNNIINA	SseK2 alone	100 s (23 C)	7.66	0.147741	7.06
SseK2	67	95	ERNPDRFGQNEIPIINTQKNPYLNNIINA	SseK2 alone	1,000 s (23 C)	10.65	0.24119	7.06
SseK2	67	95	ERNPDRFGQNEIPIINTQKNPYLNNIINA	SseK2 alone	10,000 s (23 C)	13.92	0.161702	7.04
SseK2	67	95	ERNPDRFGQNEIPIINTQKNPYLNNIINA	SseK2 alone	12 h (28 C)	15.14	0.171902	7.03
SseK2	77	83	EIPIINT	SseK2 + YM155	10 s (23 C)	0.33	0.053974	6.12
SseK2	77	83	EIPIINT	SseK2 + YM155	100 s (23 C)	1.36	0.00989	6.10
SseK2	77	83	EIPIINT	SseK2 + YM155	1,000 s (23 C)	2.57	0.022785	6.09
SseK2	77	83	EIPIINT	SseK2 + YM155	10,000 s (23 C)	3.08	0.029068	6.09
SseK2	77	83	EIPIINT	SseK2 + YM155	12 h (28 C)	3.06	0.017151	6.09

SseK2	77	83	EIPIINT	SseK2 alone	10 s (23 C)	0.22	0.013551	6.17
SseK2	77	83	EIPIINT	SseK2 alone	100 s (23 C)	1.04	0.065012	6.15
SseK2	77	83	EIPIINT	SseK2 alone	1,000 s (23 C)	2.20	0.032984	6.15
SseK2	77	83	EIPIINT	SseK2 alone	10,000 s (23 C)	3.08	0.048334	6.15
SseK2	77	83	EIPIINT	SseK2 alone	12 h (28 C)	3.08	0.003107	6.15
SseK2	77	91	EIPIINTQKNPYLNN	SseK2 + YM155	10 s (23 C)	2.10	0.003113	6.20
SseK2	77	91	EIPIINTQKNPYLNN	SseK2 + YM155	100 s (23 C)	3.73	0.012473	6.17
SseK2	77	91	EIPIINTQKNPYLNN	SseK2 + YM155	1,000 s (23 C)	5.62	0.028327	6.17
SseK2	77	91	EIPIINTQKNPYLNN	SseK2 + YM155	10,000 s (23 C)	8.16	0.025222	6.17
SseK2	77	91	EIPIINTQKNPYLNN	SseK2 + YM155	12 h (28 C)	8.53	0.069751	6.17
SseK2	77	91	EIPIINTQKNPYLNN	SseK2 alone	10 s (23 C)	1.97	0.026736	6.23
SseK2	77	91	EIPIINTQKNPYLNN	SseK2 alone	100 s (23 C)	3.37	0.097034	6.22
SseK2	77	91	EIPIINTQKNPYLNN	SseK2 alone	1,000 s (23 C)	5.24	0.134408	6.21
SseK2	77	91	EIPIINTQKNPYLNN	SseK2 alone	10,000 s (23 C)	8.21	0.068541	6.21
SseK2	77	91	EIPIINTQKNPYLNN	SseK2 alone	12 h (28 C)	8.62	0.031995	6.21
SseK2	77	95	EIPIINTQKNPYLNNIINA	SseK2 + YM155	10 s (23 C)	2.12	0.016906	7.39
SseK2	77	95	EIPIINTQKNPYLNNIINA	SseK2 + YM155	100 s (23 C)	3.79	0.022227	7.36
SseK2	77	95	EIPIINTQKNPYLNNIINA	SseK2 + YM155	1,000 s (23 C)	5.75	0.044575	7.37
SseK2	77	95	EIPIINTQKNPYLNNIINA	SseK2 + YM155	10,000 s (23 C)	8.43	0.07837	7.36
SseK2	77	95	EIPIINTQKNPYLNNIINA	SseK2 + YM155	12 h (28 C)	9.55	0.005006	7.35
SseK2	77	95	EIPIINTQKNPYLNNIINA	SseK2 alone	10 s (23 C)	1.98	0.055773	7.38
SseK2	77	95	EIPIINTQKNPYLNNIINA	SseK2 alone	100 s (23 C)	3.34	0.097717	7.37
SseK2	77	95	EIPIINTQKNPYLNNIINA	SseK2 alone	1,000 s (23 C)	5.24	0.1176	7.37
SseK2	77	95	EIPIINTQKNPYLNNIINA	SseK2 alone	10,000 s (23 C)	8.44	0.085855	7.36
SseK2	77	95	EIPIINTQKNPYLNNIINA	SseK2 alone	12 h (28 C)	9.60	0.015172	7.35
SseK2	78	83	IPIINT	SseK2 + YM155	10 s (23 C)	0.28	0.031113	5.81
SseK2	78	83	IPIINT	SseK2 + YM155	100 s (23 C)	1.50	0.024738	5.78
SseK2	78	83	IPIINT	SseK2 + YM155	1,000 s (23 C)	2.68	0.039846	5.78

SseK2	78	83	IPIINT	SseK2 + YM155	10,000 s (23 C)	3.18	0.010186	5.78
SseK2	78	83	IPIINT	SseK2 + YM155	12 h (28 C)	3.19	0.016954	5.79
SseK2	78	83	IPIINT	SseK2 alone	10 s (23 C)	0.17	0.026351	5.84
SseK2	78	83	IPIINT	SseK2 alone	100 s (23 C)	1.00	0.054506	5.83
SseK2	78	83	IPIINT	SseK2 alone	1,000 s (23 C)	2.26	0.013234	5.83
SseK2	78	83	IPIINT	SseK2 alone	10,000 s (23 C)	3.21	0.033566	5.83
SseK2	78	83	IPIINT	SseK2 alone	12 h (28 C)	3.19	0.031083	5.83
SseK2	80	95	IINTQKNPYLNNIINA	SseK2 + YM155	10 s (23 C)	1.24	0.026613	8.17
SseK2	80	95	IINTQKNPYLNNIINA	SseK2 + YM155	100 s (23 C)	1.68	0.050678	8.16
SseK2	80	95	IINTQKNPYLNNIINA	SseK2 + YM155	1,000 s (23 C)	2.68	0.052163	8.16
SseK2	80	95	IINTQKNPYLNNIINA	SseK2 + YM155	10,000 s (23 C)	4.65	0.053659	8.16
SseK2	80	95	IINTQKNPYLNNIINA	SseK2 + YM155	12 h (28 C)	5.97	0.049757	8.15
SseK2	80	95	IINTQKNPYLNNIINA	SseK2 alone	10 s (23 C)	1.27	0.041359	8.17
SseK2	80	95	IINTQKNPYLNNIINA	SseK2 alone	100 s (23 C)	1.77	0.026125	8.16
SseK2	80	95	IINTQKNPYLNNIINA	SseK2 alone	1,000 s (23 C)	2.70	0.136367	8.16
SseK2	80	95	IINTQKNPYLNNIINA	SseK2 alone	10,000 s (23 C)	4.66	0.09538	8.16
SseK2	80	95	IINTQKNPYLNNIINA	SseK2 alone	12 h (28 C)	6.00	0.075341	8.16
SseK2	84	91	QKNPYLNN	SseK2 + YM155	10 s (23 C)	0.27	0.075912	4.31
SseK2	84	91	QKNPYLNN	SseK2 + YM155	100 s (23 C)	0.73	0.011561	4.29
SseK2	84	91	QKNPYLNN	SseK2 + YM155	1,000 s (23 C)	1.36	0.043301	4.29
SseK2	84	91	QKNPYLNN	SseK2 + YM155	10,000 s (23 C)	3.40	0.039521	4.30
SseK2	84	91	QKNPYLNN	SseK2 + YM155	12 h (28 C)	3.79	0.004184	4.29
SseK2	84	91	QKNPYLNN	SseK2 alone	10 s (23 C)	0.24	0.031031	4.30
SseK2	84	91	QKNPYLNN	SseK2 alone	100 s (23 C)	0.75	0.041138	4.29
SseK2	84	91	QKNPYLNN	SseK2 alone	1,000 s (23 C)	1.40	0.082245	4.29
SseK2	84	91	QKNPYLNN	SseK2 alone	10,000 s (23 C)	3.51	0.090705	4.29
SseK2	84	91	QKNPYLNN	SseK2 alone	12 h (28 C)	3.85	0.003535	4.28
SseK2	84	95	QKNPYLNNIINA	SseK2 + YM155	10 s (23 C)	0.30	0.06367	6.41

SseK2	84	95	QKNPYLNNIINA	SseK2 + YM155	100 s (23 C)	0.56	0.021526	6.40
SseK2	84	95	QKNPYLNNIINA	SseK2 + YM155	1,000 s (23 C)	1.19	0.044563	6.39
SseK2	84	95	QKNPYLNNIINA	SseK2 + YM155	10,000 s (23 C)	3.26	0.02545	6.38
SseK2	84	95	QKNPYLNNIINA	SseK2 + YM155	12 h (28 C)	4.32	0.000608	6.38
SseK2	84	95	QKNPYLNNIINA	SseK2 alone	10 s (23 C)	0.22	0.027527	6.47
SseK2	84	95	QKNPYLNNIINA	SseK2 alone	100 s (23 C)	0.52	0.016333	6.46
SseK2	84	95	QKNPYLNNIINA	SseK2 alone	1,000 s (23 C)	1.14	0.110497	6.47
SseK2	84	95	QKNPYLNNIINA	SseK2 alone	10,000 s (23 C)	3.25	0.066368	6.46
SseK2	84	95	QKNPYLNNIINA	SseK2 alone	12 h (28 C)	4.28	0.003896	6.45
SseK2	86	91	NPYLNN	SseK2 + YM155	10 s (23 C)	0.29	ND	4.32
SseK2	86	91	NPYLNN	SseK2 + YM155	100 s (23 C)	0.47	0.025021	4.29
SseK2	86	91	NPYLNN	SseK2 + YM155	1,000 s (23 C)	0.74	0.04553	4.29
SseK2	86	91	NPYLNN	SseK2 + YM155	10,000 s (23 C)	2.33	0.022976	4.29
SseK2	86	91	NPYLNN	SseK2 + YM155	12 h (28 C)	2.84	0.020198	4.29
SseK2	86	91	NPYLNN	SseK2 alone	10 s (23 C)	0.26	0.017442	4.31
SseK2	86	91	NPYLNN	SseK2 alone	100 s (23 C)	0.53	0.051803	4.29
SseK2	86	91	NPYLNN	SseK2 alone	1,000 s (23 C)	0.84	0.060162	4.29
SseK2	86	91	NPYLNN	SseK2 alone	10,000 s (23 C)	2.39	0.035477	4.29
SseK2	86	91	NPYLNN	SseK2 alone	12 h (28 C)	2.82	ND	4.28
SseK2	90	99	NNIINAAIIE	SseK2 + YM155	10 s (23 C)	0.35	0.069805	6.13
SseK2	90	99	NNIINAAIIE	SseK2 + YM155	100 s (23 C)	0.87	0.023299	6.10
SseK2	90	99	NNIINAAIIE	SseK2 + YM155	1,000 s (23 C)	3.65	0.006721	6.09
SseK2	90	99	NNIINAAIIE	SseK2 + YM155	10,000 s (23 C)	5.28	0.017355	6.08
SseK2	90	99	NNIINAAIIE	SseK2 + YM155	12 h (28 C)	5.31	0.058676	6.08
SseK2	90	99	NNIINAAIIE	SseK2 alone	10 s (23 C)	0.19	0.005232	6.17
SseK2	90	99	NNIINAAIIE	SseK2 alone	100 s (23 C)	0.99	0.149799	6.15
SseK2	90	99	NNIINAAIIE	SseK2 alone	1,000 s (23 C)	3.65	0.029901	6.13
SseK2	90	99	NNIINAAIIE	SseK2 alone	10,000 s (23 C)	5.42	0.076811	6.13

SseK2	90	99	NNIINAAIIE	SseK2 alone	12 h (28 C)	5.49	0.011067	6.12
SseK2	92	96	IINAA	SseK2 + YM155	10 s (23 C)	0.09	0.029588	3.74
SseK2	92	96	IINAA	SseK2 + YM155	100 s (23 C)	0.08	0.007379	3.73
SseK2	92	96	IINAA	SseK2 + YM155	1,000 s (23 C)	0.11	0.014545	3.72
SseK2	92	96	IINAA	SseK2 + YM155	10,000 s (23 C)	0.10	0.025245	3.74
SseK2	92	96	IINAA	SseK2 + YM155	12 h (28 C)	0.54	0.033326	3.73
SseK2	92	96	IINAA	SseK2 alone	10 s (23 C)	0.09	0.020441	3.74
SseK2	92	96	IINAA	SseK2 alone	100 s (23 C)	0.10	0.010024	3.73
SseK2	92	96	IINAA	SseK2 alone	1,000 s (23 C)	0.09	0.009108	3.73
SseK2	92	96	IINAA	SseK2 alone	10,000 s (23 C)	0.09	0.016922	3.72
SseK2	92	96	IINAA	SseK2 alone	12 h (28 C)	0.40	0.02103	3.73
SseK2	92	107	IINAAIIEKERIIGIF	SseK2 + YM155	10 s (23 C)	0.87	0.044665	7.76
SseK2	92	107	IINAAIIEKERIIGIF	SseK2 + YM155	100 s (23 C)	1.30	0.065805	7.74
SseK2	92	107	IINAAIIEKERIIGIF	SseK2 + YM155	1,000 s (23 C)	1.99	0.044675	7.75
SseK2	92	107	IINAAIIEKERIIGIF	SseK2 + YM155	10,000 s (23 C)	2.29	0.023499	7.75
SseK2	92	107	IINAAIIEKERIIGIF	SseK2 + YM155	12 h (28 C)	2.78	0.038569	7.75
SseK2	92	107	IINAAIIEKERIIGIF	SseK2 alone	10 s (23 C)	0.89	0.023897	7.76
SseK2	92	107	IINAAIIEKERIIGIF	SseK2 alone	100 s (23 C)	1.38	0.036906	7.75
SseK2	92	107	IINAAIIEKERIIGIF	SseK2 alone	1,000 s (23 C)	2.01	0.060475	7.75
SseK2	92	107	IINAAIIEKERIIGIF	SseK2 alone	10,000 s (23 C)	2.30	0.028845	7.76
SseK2	92	107	IINAAIIEKERIIGIF	SseK2 alone	12 h (28 C)	2.84	0.028086	7.76
SseK2	95	99	AAIIE	SseK2 + YM155	10 s (23 C)	0.08	0.010903	4.80
SseK2	95	99	AAIIE	SseK2 + YM155	100 s (23 C)	0.08	0.010744	4.77
SseK2	95	99	AAIIE	SseK2 + YM155	1,000 s (23 C)	0.09	0.024704	4.78
SseK2	95	99	AAIIE	SseK2 + YM155	10,000 s (23 C)	0.10	0.001419	4.78
SseK2	95	99	AAIIE	SseK2 + YM155	12 h (28 C)	0.23	0.01964	4.78
SseK2	95	99	AAIIE	SseK2 alone	10 s (23 C)	0.10	0.018141	4.79
SseK2	95	99	AAIIE	SseK2 alone	100 s (23 C)	0.10	0.03162	4.78

SseK2	95	99	AAIIE	SseK2 alone	1,000 s (23 C)	0.09	0.014263	4.78
SseK2	95	99	AAIIE	SseK2 alone	10,000 s (23 C)	0.10	0.009091	4.78
SseK2	95	99	AAIIE	SseK2 alone	12 h (28 C)	0.17	0.008405	4.78
SseK2	95	107	AAIIEKERIIGIF	SseK2 + YM155	10 s (23 C)	0.90	0.039425	7.30
SseK2	95	107	AAIIEKERIIGIF	SseK2 + YM155	100 s (23 C)	1.26	0.011587	7.27
SseK2	95	107	AAIIEKERIIGIF	SseK2 + YM155	1,000 s (23 C)	1.87	0.017069	7.29
SseK2	95	107	AAIIEKERIIGIF	SseK2 + YM155	10,000 s (23 C)	2.15	0.019464	7.29
SseK2	95	107	AAIIEKERIIGIF	SseK2 + YM155	12 h (28 C)	2.90	0.003746	7.28
SseK2	95	107	AAIIEKERIIGIF	SseK2 alone	10 s (23 C)	0.89	0.037212	7.29
SseK2	95	107	AAIIEKERIIGIF	SseK2 alone	100 s (23 C)	1.32	0.04857	7.28
SseK2	95	107	AAIIEKERIIGIF	SseK2 alone	1,000 s (23 C)	1.89	0.020031	7.29
SseK2	95	107	AAIIEKERIIGIF	SseK2 alone	10,000 s (23 C)	2.20	0.01948	7.29
SseK2	95	107	AAIIEKERIIGIF	SseK2 alone	12 h (28 C)	2.87	0.026766	7.28
SseK2	96	101	AIIEKE	SseK2 + YM155	10 s (23 C)	0.41	0.018716	3.58
SseK2	96	101	AIIEKE	SseK2 + YM155	100 s (23 C)	0.57	0.013157	3.58
SseK2	96	101	AIIEKE	SseK2 + YM155	1,000 s (23 C)	0.66	0.015358	3.57
SseK2	96	101	AIIEKE	SseK2 + YM155	10,000 s (23 C)	0.64	0.018096	3.59
SseK2	96	101	AIIEKE	SseK2 + YM155	12 h (28 C)	0.97	0.010225	3.58
SseK2	96	101	AIIEKE	SseK2 alone	10 s (23 C)	0.41	0.024667	3.59
SseK2	96	101	AIIEKE	SseK2 alone	100 s (23 C)	0.57	0.024776	3.57
SseK2	96	101	AIIEKE	SseK2 alone	1,000 s (23 C)	0.64	0.010782	3.57
SseK2	96	101	AIIEKE	SseK2 alone	10,000 s (23 C)	0.65	0.042673	3.55
SseK2	96	101	AIIEKE	SseK2 alone	12 h (28 C)	0.93	0.020064	3.57
SseK2	96	105	AIIEKERIIG	SseK2 + YM155	10 s (23 C)	0.75	0.032937	4.91
SseK2	96	105	AIIEKERIIG	SseK2 + YM155	100 s (23 C)	1.15	0.015144	4.89
SseK2	96	105	AIIEKERIIG	SseK2 + YM155	1,000 s (23 C)	1.78	0.035372	4.89
SseK2	96	105	AIIEKERIIG	SseK2 + YM155	10,000 s (23 C)	2.01	0.029275	4.89
SseK2	96	105	AIIEKERIIG	SseK2 + YM155	12 h (28 C)	3.20	0.057974	4.89

SseK2	96	105	AIIEKERIIG	SseK2 alone	10 s (23 C)	0.75	0.032739	4.92
SseK2	96	105	AIIEKERIIG	SseK2 alone	100 s (23 C)	1.20	0.041352	4.91
SseK2	96	105	AIIEKERIIG	SseK2 alone	1,000 s (23 C)	1.82	0.013796	4.91
SseK2	96	105	AIIEKERIIG	SseK2 alone	10,000 s (23 C)	2.08	0.025975	4.91
SseK2	96	105	AIIEKERIIG	SseK2 alone	12 h (28 C)	3.11	0.004394	4.91
SseK2	96	106	AIIEKERIIGI	SseK2 + YM155	10 s (23 C)	0.89	0.031668	6.06
SseK2	96	106	AIIEKERIIGI	SseK2 + YM155	100 s (23 C)	1.23	0.023158	6.03
SseK2	96	106	AIIEKERIIGI	SseK2 + YM155	1,000 s (23 C)	1.85	0.039971	6.03
SseK2	96	106	AIIEKERIIGI	SseK2 + YM155	10,000 s (23 C)	2.14	0.03101	6.02
SseK2	96	106	AIIEKERIIGI	SseK2 + YM155	12 h (28 C)	3.08	0.005233	6.03
SseK2	96	106	AIIEKERIIGI	SseK2 alone	10 s (23 C)	0.86	0.039863	6.10
SseK2	96	106	AIIEKERIIGI	SseK2 alone	100 s (23 C)	1.30	0.040235	6.09
SseK2	96	106	AIIEKERIIGI	SseK2 alone	1,000 s (23 C)	1.87	0.028096	6.09
SseK2	96	106	AIIEKERIIGI	SseK2 alone	10,000 s (23 C)	2.15	0.013853	6.10
SseK2	96	106	AIIEKERIIGI	SseK2 alone	12 h (28 C)	3.05	0.011807	6.09
SseK2	96	107	AIIEKERIIGIF	SseK2 + YM155	10 s (23 C)	0.72	0.071414	7.30
SseK2	96	107	AIIEKERIIGIF	SseK2 + YM155	100 s (23 C)	0.95	0.008368	7.27
SseK2	96	107	AIIEKERIIGIF	SseK2 + YM155	1,000 s (23 C)	1.57	0.057129	7.29
SseK2	96	107	AIIEKERIIGIF	SseK2 + YM155	10,000 s (23 C)	1.83	0.034334	7.29
SseK2	96	107	AIIEKERIIGIF	SseK2 + YM155	12 h (28 C)	3.70	0.013045	7.28
SseK2	96	107	AIIEKERIIGIF	SseK2 alone	10 s (23 C)	0.71	0.021978	7.29
SseK2	96	107	AIIEKERIIGIF	SseK2 alone	100 s (23 C)	1.00	0.034513	7.28
SseK2	96	107	AIIEKERIIGIF	SseK2 alone	1,000 s (23 C)	1.59	0.067021	7.28
SseK2	96	107	AIIEKERIIGIF	SseK2 alone	10,000 s (23 C)	1.86	0.071235	7.29
SseK2	96	107	AIIEKERIIGIF	SseK2 alone	12 h (28 C)	3.43	0.018871	7.28
SseK2	97	105	IIEKERIIG	SseK2 + YM155	10 s (23 C)	0.74	0.032361	4.91
SseK2	97	105	IIEKERIIG	SseK2 + YM155	100 s (23 C)	1.03	0.013304	4.89
SseK2	97	105	IIEKERIIG	SseK2 + YM155	1,000 s (23 C)	1.53	0.052405	4.89

SseK2	97	105	IIEKERIIG	SseK2 + YM155	10,000 s (23 C)	1.85	0.016917	4.89
SseK2	97	105	IIEKERIIG	SseK2 + YM155	12 h (28 C)	2.82	0.023838	4.89
SseK2	97	105	IIEKERIIG	SseK2 alone	10 s (23 C)	0.73	0.051521	4.92
SseK2	97	105	IIEKERIIG	SseK2 alone	100 s (23 C)	1.03	0.065158	4.90
SseK2	97	105	IIEKERIIG	SseK2 alone	1,000 s (23 C)	1.60	0.044144	4.90
SseK2	97	105	IIEKERIIG	SseK2 alone	10,000 s (23 C)	1.84	0.036054	4.91
SseK2	97	105	IIEKERIIG	SseK2 alone	12 h (28 C)	2.71	0.015686	4.90
SseK2	97	107	IIEKERIIGIF	SseK2 + YM155	10 s (23 C)	0.83	0.064133	7.30
SseK2	97	107	IIEKERIIGIF	SseK2 + YM155	100 s (23 C)	1.30	0.010117	7.18
SseK2	97	107	IIEKERIIGIF	SseK2 + YM155	1,000 s (23 C)	1.93	0.043939	7.19
SseK2	97	107	IIEKERIIGIF	SseK2 + YM155	10,000 s (23 C)	2.03	0.11587	7.25
SseK2	97	107	IIEKERIIGIF	SseK2 + YM155	12 h (28 C)	3.36	0.00269	7.18
SseK2	97	107	IIEKERIIGIF	SseK2 alone	10 s (23 C)	0.86	0.018776	7.28
SseK2	97	107	IIEKERIIGIF	SseK2 alone	100 s (23 C)	1.34	0.049257	7.19
SseK2	97	107	IIEKERIIGIF	SseK2 alone	1,000 s (23 C)	1.78	0.131232	7.24
SseK2	97	107	IIEKERIIGIF	SseK2 alone	10,000 s (23 C)	2.02	0.082553	7.27
SseK2	97	107	IIEKERIIGIF	SseK2 alone	12 h (28 C)	3.26	0.011921	7.18
SseK2	98	105	IEKERIIG	SseK2 + YM155	10 s (23 C)	0.60	0.022708	4.91
SseK2	98	105	IEKERIIG	SseK2 + YM155	100 s (23 C)	0.87	0.022618	4.89
SseK2	98	105	IEKERIIG	SseK2 + YM155	1,000 s (23 C)	1.44	0.013154	4.89
SseK2	98	105	IEKERIIG	SseK2 + YM155	10,000 s (23 C)	1.70	0.023208	4.89
SseK2	98	105	IEKERIIG	SseK2 + YM155	12 h (28 C)	2.71	0.01196	4.89
SseK2	98	105	IEKERIIG	SseK2 alone	10 s (23 C)	0.57	0.023549	4.92
SseK2	98	105	IEKERIIG	SseK2 alone	100 s (23 C)	0.93	0.025485	4.90
SseK2	98	105	IEKERIIG	SseK2 alone	1,000 s (23 C)	1.47	0.038484	4.91
SseK2	98	105	IEKERIIG	SseK2 alone	10,000 s (23 C)	1.72	0.050516	4.91
SseK2	98	105	IEKERIIG	SseK2 alone	12 h (28 C)	2.69	0.078772	4.90
SseK2	98	106	IEKERIIGI	SseK2 + YM155	10 s (23 C)	0.66	0.045434	6.06

SseK2	98	106	IEKERIIGI	SseK2 + YM155	100 s (23 C)	0.97	0.024529	6.03
SseK2	98	106	IEKERIIGI	SseK2 + YM155	1,000 s (23 C)	1.56	0.019204	6.03
SseK2	98	106	IEKERIIGI	SseK2 + YM155	10,000 s (23 C)	1.82	0.02022	6.03
SseK2	98	106	IEKERIIGI	SseK2 + YM155	12 h (28 C)	2.84	0.013233	6.03
SseK2	98	106	IEKERIIGI	SseK2 alone	10 s (23 C)	0.65	0.014393	6.10
SseK2	98	106	IEKERIIGI	SseK2 alone	100 s (23 C)	1.03	0.025708	6.09
SseK2	98	106	IEKERIIGI	SseK2 alone	1,000 s (23 C)	1.54	0.028995	6.09
SseK2	98	106	IEKERIIGI	SseK2 alone	10,000 s (23 C)	1.83	0.040581	6.09
SseK2	98	106	IEKERIIGI	SseK2 alone	12 h (28 C)	2.82	0.014146	6.08
SseK2	98	107	IEKERIIGIF	SseK2 + YM155	10 s (23 C)	0.55	0.061236	7.30
SseK2	98	107	IEKERIIGIF	SseK2 + YM155	100 s (23 C)	0.74	0.015417	7.27
SseK2	98	107	IEKERIIGIF	SseK2 + YM155	1,000 s (23 C)	1.20	0.014851	7.29
SseK2	98	107	IEKERIIGIF	SseK2 + YM155	10,000 s (23 C)	1.48	0.030648	7.29
SseK2	98	107	IEKERIIGIF	SseK2 + YM155	12 h (28 C)	2.69	0.00983	7.28
SseK2	98	107	IEKERIIGIF	SseK2 alone	10 s (23 C)	0.54	0.05199	7.29
SseK2	98	107	IEKERIIGIF	SseK2 alone	100 s (23 C)	0.79	0.03067	7.28
SseK2	98	107	IEKERIIGIF	SseK2 alone	1,000 s (23 C)	1.20	0.031664	7.29
SseK2	98	107	IEKERIIGIF	SseK2 alone	10,000 s (23 C)	1.48	0.036153	7.29
SseK2	98	107	IEKERIIGIF	SseK2 alone	12 h (28 C)	2.62	0.03831	7.29
SseK2	100	107	KERIIGIF	SseK2 + YM155	10 s (23 C)	0.11	0.017405	6.73
SseK2	100	107	KERIIGIF	SseK2 + YM155	100 s (23 C)	0.14	0.031862	6.69
SseK2	100	107	KERIIGIF	SseK2 + YM155	1,000 s (23 C)	0.47	0.036494	6.71
SseK2	100	107	KERIIGIF	SseK2 + YM155	10,000 s (23 C)	0.67	0.006461	6.71
SseK2	100	107	KERIIGIF	SseK2 + YM155	12 h (28 C)	1.61	0.02642	6.71
SseK2	100	107	KERIIGIF	SseK2 alone	10 s (23 C)	0.11	0.019788	6.80
SseK2	100	107	KERIIGIF	SseK2 alone	100 s (23 C)	0.19	0.021587	6.79
SseK2	100	107	KERIIGIF	SseK2 alone	1,000 s (23 C)	0.55	0.05534	6.80
SseK2	100	107	KERIIGIF	SseK2 alone	10,000 s (23 C)	0.75	0.018606	6.80

SseK2	100	107	KERIIGIF	SseK2 alone	12 h (28 C)	1.68	0.027834	6.79
SseK2	106	112	IFVDGDF	SseK2 + YM155	10 s (23 C)	0.69	0.081874	7.57
SseK2	106	112	IFVDGDF	SseK2 + YM155	100 s (23 C)	1.14	0.020055	7.55
SseK2	106	112	IFVDGDF	SseK2 + YM155	1,000 s (23 C)	1.81	0.01838	7.56
SseK2	106	112	IFVDGDF	SseK2 + YM155	10,000 s (23 C)	1.85	0.032619	7.56
SseK2	106	112	IFVDGDF	SseK2 + YM155	12 h (28 C)	2.27	0.008356	7.55
SseK2	106	112	IFVDGDF	SseK2 alone	10 s (23 C)	0.64	0.004965	7.56
SseK2	106	112	IFVDGDF	SseK2 alone	100 s (23 C)	1.19	0.113255	7.56
SseK2	106	112	IFVDGDF	SseK2 alone	1,000 s (23 C)	1.81	0.024109	7.56
SseK2	106	112	IFVDGDF	SseK2 alone	10,000 s (23 C)	1.84	0.022042	7.56
SseK2	106	112	IFVDGDF	SseK2 alone	12 h (28 C)	2.22	0.021016	7.55
SseK2	108	125	VDGDFSKGQRKALGKLEQ	SseK2 + YM155	10 s (23 C)	1.41	0.133351	4.49
SseK2	108	125	VDGDFSKGQRKALGKLEQ	SseK2 + YM155	100 s (23 C)	2.38	0.012065	4.47
SseK2	108	125	VDGDFSKGQRKALGKLEQ	SseK2 + YM155	1,000 s (23 C)	5.09	0.084089	4.45
SseK2	108	125	VDGDFSKGQRKALGKLEQ	SseK2 + YM155	10,000 s (23 C)	6.68	0.076311	4.45
SseK2	108	125	VDGDFSKGQRKALGKLEQ	SseK2 + YM155	12 h (28 C)	8.44	0.033356	4.44
SseK2	108	125	VDGDFSKGQRKALGKLEQ	SseK2 alone	10 s (23 C)	1.28	0.057274	4.49
SseK2	108	125	VDGDFSKGQRKALGKLEQ	SseK2 alone	100 s (23 C)	2.39	0.059534	4.48
SseK2	108	125	VDGDFSKGQRKALGKLEQ	SseK2 alone	1,000 s (23 C)	4.99	0.017675	4.47
SseK2	108	125	VDGDFSKGQRKALGKLEQ	SseK2 alone	10,000 s (23 C)	6.71	0.175995	4.46
SseK2	108	125	VDGDFSKGQRKALGKLEQ	SseK2 alone	12 h (28 C)	8.46	0.026968	4.44
SseK2	108	126	VDGDFSKGQRKALGKLEQN	SseK2 + YM155	10 s (23 C)	1.83	0.011232	4.49
SseK2	108	126	VDGDFSKGQRKALGKLEQN	SseK2 + YM155	100 s (23 C)	3.35	0.025216	4.46
SseK2	108	126	VDGDFSKGQRKALGKLEQN	SseK2 + YM155	1,000 s (23 C)	6.31	0.070533	4.45
SseK2	108	126	VDGDFSKGQRKALGKLEQN	SseK2 + YM155	10,000 s (23 C)	7.83	0.07711	4.45
SseK2	108	126	VDGDFSKGQRKALGKLEQN	SseK2 + YM155	12 h (28 C)	9.80	0.007688	4.44
SseK2	108	126	VDGDFSKGQRKALGKLEQN	SseK2 alone	10 s (23 C)	1.84	0.025688	4.48
SseK2	108	126	VDGDFSKGQRKALGKLEQN	SseK2 alone	100 s (23 C)	3.48	0.095928	4.47

SseK2	108	126	VDGDFSKGQRKALGKLEQN	SseK2 alone	1,000 s (23 C)	6.32	0.048615	4.47
SseK2	108	126	VDGDFSKGQRKALGKLEQN	SseK2 alone	10,000 s (23 C)	7.92	0.076392	4.46
SseK2	108	126	VDGDFSKGQRKALGKLEQN	SseK2 alone	12 h (28 C)	9.87	0.018848	4.44
SseK2	108	129	VDGDFSKGQRKALGKLEQNYRN	SseK2 + YM155	10 s (23 C)	2.70	0.088456	4.84
SseK2	108	129	VDGDFSKGQRKALGKLEQNYRN	SseK2 + YM155	100 s (23 C)	4.54	0.055736	4.82
SseK2	108	129	VDGDFSKGQRKALGKLEQNYRN	SseK2 + YM155	1,000 s (23 C)	8.32	0.069119	4.81
SseK2	108	129	VDGDFSKGQRKALGKLEQNYRN	SseK2 + YM155	10,000 s (23 C)	10.29	0.058756	4.80
SseK2	108	129	VDGDFSKGQRKALGKLEQNYRN	SseK2 + YM155	12 h (28 C)	12.26	0.096611	4.78
SseK2	108	129	VDGDFSKGQRKALGKLEQNYRN	SseK2 alone	10 s (23 C)	2.70	0.007054	4.85
SseK2	108	129	VDGDFSKGQRKALGKLEQNYRN	SseK2 alone	100 s (23 C)	4.55	0.121006	4.83
SseK2	108	129	VDGDFSKGQRKALGKLEQNYRN	SseK2 alone	1,000 s (23 C)	8.29	0.085946	4.82
SseK2	108	129	VDGDFSKGQRKALGKLEQNYRN	SseK2 alone	10,000 s (23 C)	10.25	0.055296	4.81
SseK2	108	129	VDGDFSKGQRKALGKLEQNYRN	SseK2 alone	12 h (28 C)	12.18	0.076891	4.79
SseK2	108	132	VDGDFSKGQRKALGKLEQNYRNIKV	SseK2 + YM155	10 s (23 C)	2.11	0.090258	5.80
SseK2	108	132	VDGDFSKGQRKALGKLEQNYRNIKV	SseK2 + YM155	100 s (23 C)	3.78	0.062306	5.76
SseK2	108	132	VDGDFSKGQRKALGKLEQNYRNIKV	SseK2 + YM155	1,000 s (23 C)	7.21	0.073849	5.77
SseK2	108	132	VDGDFSKGQRKALGKLEQNYRNIKV	SseK2 + YM155	10,000 s (23 C)	9.42	0.081469	5.76
SseK2	108	132	VDGDFSKGQRKALGKLEQNYRNIKV	SseK2 + YM155	12 h (28 C)	11.79	0.138917	5.74
SseK2	108	132	VDGDFSKGQRKALGKLEQNYRNIKV	SseK2 alone	10 s (23 C)	2.04	0.051966	5.82
SseK2	108	132	VDGDFSKGQRKALGKLEQNYRNIKV	SseK2 alone	100 s (23 C)	3.81	0.19573	5.80
SseK2	108	132	VDGDFSKGQRKALGKLEQNYRNIKV	SseK2 alone	1,000 s (23 C)	7.08	0.132465	5.80
SseK2	108	132	VDGDFSKGQRKALGKLEQNYRNIKV	SseK2 alone	10,000 s (23 C)	9.46	0.096232	5.78
SseK2	108	132	VDGDFSKGQRKALGKLEQNYRNIKV	SseK2 alone	12 h (28 C)	11.81	0.026691	5.76
SseK2	108	138	VDGDFSKGQRKALGKLEQNYRNIKVIYNSDL	SseK2 + YM155	10 s (23 C)	3.01	0.075709	6.43
SseK2	108	138	VDGDFSKGQRKALGKLEQNYRNIKVIYNSDL	SseK2 + YM155	100 s (23 C)	5.24	0.001261	6.41
SseK2	108	138	VDGDFSKGQRKALGKLEQNYRNIKVIYNSDL	SseK2 + YM155	1,000 s (23 C)	9.58	0.069168	6.39
SseK2	108	138	VDGDFSKGQRKALGKLEQNYRNIKVIYNSDL	SseK2 + YM155	10,000 s (23 C)	13.07	0.067635	6.37
SseK2	108	138	VDGDFSKGQRKALGKLEQNYRNIKVIYNSDL	SseK2 + YM155	12 h (28 C)	17.09	0.11664	6.36

SseK2	108	138	VDGDFSKGQRKALGKLEQNYRNIKVIYNSDL	SseK2 alone	10 s (23 C)	3.11	0.109032	6.46
SseK2	108	138	VDGDFSKGQRKALGKLEQNYRNIKVIYNSDL	SseK2 alone	100 s (23 C)	5.23	0.009374	6.45
SseK2	108	138	VDGDFSKGQRKALGKLEQNYRNIKVIYNSDL	SseK2 alone	1,000 s (23 C)	9.46	0.200811	6.43
SseK2	108	138	VDGDFSKGQRKALGKLEQNYRNIKVIYNSDL	SseK2 alone	10,000 s (23 C)	13.08	0.129122	6.42
SseK2	108	138	VDGDFSKGQRKALGKLEQNYRNIKVIYNSDL	SseK2 alone	12 h (28 C)	17.11	0.131678	6.40
SseK2	109	126	DGDFSKGQRKALGKLEQN	SseK2 + YM155	10 s (23 C)	1.55	0.102615	4.49
SseK2	109	126	DGDFSKGQRKALGKLEQN	SseK2 + YM155	100 s (23 C)	2.82	0.022181	4.46
SseK2	109	126	DGDFSKGQRKALGKLEQN	SseK2 + YM155	1,000 s (23 C)	5.43	0.096228	4.46
SseK2	109	126	DGDFSKGQRKALGKLEQN	SseK2 + YM155	10,000 s (23 C)	6.89	0.08561	4.45
SseK2	109	126	DGDFSKGQRKALGKLEQN	SseK2 + YM155	12 h (28 C)	8.58	0.03746	4.44
SseK2	109	126	DGDFSKGQRKALGKLEQN	SseK2 alone	10 s (23 C)	1.55	0.025862	4.49
SseK2	109	126	DGDFSKGQRKALGKLEQN	SseK2 alone	100 s (23 C)	2.96	0.052591	4.47
SseK2	109	126	DGDFSKGQRKALGKLEQN	SseK2 alone	1,000 s (23 C)	5.38	0.089635	4.47
SseK2	109	126	DGDFSKGQRKALGKLEQN	SseK2 alone	10,000 s (23 C)	6.96	0.128949	4.46
SseK2	109	126	DGDFSKGQRKALGKLEQN	SseK2 alone	12 h (28 C)	8.60	0.005805	4.44
SseK2	109	132	DGDFSKGQRKALGKLEQNYRNIKV	SseK2 + YM155	10 s (23 C)	2.30	0.066288	5.81
SseK2	109	132	DGDFSKGQRKALGKLEQNYRNIKV	SseK2 + YM155	100 s (23 C)	4.05	0.109247	5.77
SseK2	109	132	DGDFSKGQRKALGKLEQNYRNIKV	SseK2 + YM155	1,000 s (23 C)	7.45	0.108323	5.76
SseK2	109	132	DGDFSKGQRKALGKLEQNYRNIKV	SseK2 + YM155	10,000 s (23 C)	9.66	0.116577	5.75
SseK2	109	132	DGDFSKGQRKALGKLEQNYRNIKV	SseK2 + YM155	12 h (28 C)	11.82	0.112242	5.74
SseK2	109	132	DGDFSKGQRKALGKLEQNYRNIKV	SseK2 alone	10 s (23 C)	2.38	0.029514	5.82
SseK2	109	132	DGDFSKGQRKALGKLEQNYRNIKV	SseK2 alone	100 s (23 C)	4.17	0.161603	5.80
SseK2	109	132	DGDFSKGQRKALGKLEQNYRNIKV	SseK2 alone	1,000 s (23 C)	7.36	0.142385	5.79
SseK2	109	132	DGDFSKGQRKALGKLEQNYRNIKV	SseK2 alone	10,000 s (23 C)	9.63	0.237293	5.78
SseK2	109	132	DGDFSKGQRKALGKLEQNYRNIKV	SseK2 alone	12 h (28 C)	11.80	0.109481	5.76
SseK2	109	138	DGDFSKGQRKALGKLEQNYRNIKVIYNSDL	SseK2 + YM155	10 s (23 C)	2.85	0.105586	6.43
SseK2	109	138	DGDFSKGQRKALGKLEQNYRNIKVIYNSDL	SseK2 + YM155	100 s (23 C)	4.85	0.053935	6.41
SseK2	109	138	DGDFSKGQRKALGKLEQNYRNIKVIYNSDL	SseK2 + YM155	1,000 s (23 C)	9.04	0.157099	6.39

SseK2	109	138	DGDFSKGQRKALGKLEQNYRNIKVIYNSDL	SseK2 + YM155	10,000 s (23 C)	12.38	0.061656	6.37
SseK2	109	138	DGDFSKGQRKALGKLEQNYRNIKVIYNSDL	SseK2 + YM155	12 h (28 C)	16.31	0.2439	6.36
SseK2	109	138	DGDFSKGQRKALGKLEQNYRNIKVIYNSDL	SseK2 alone	10 s (23 C)	2.80	0.065782	6.46
SseK2	109	138	DGDFSKGQRKALGKLEQNYRNIKVIYNSDL	SseK2 alone	100 s (23 C)	4.78	0.002008	6.45
SseK2	109	138	DGDFSKGQRKALGKLEQNYRNIKVIYNSDL	SseK2 alone	1,000 s (23 C)	8.95	0.178885	6.43
SseK2	109	138	DGDFSKGQRKALGKLEQNYRNIKVIYNSDL	SseK2 alone	10,000 s (23 C)	12.51	0.201792	6.42
SseK2	109	138	DGDFSKGQRKALGKLEQNYRNIKVIYNSDL	SseK2 alone	12 h (28 C)	16.27	0.114266	6.40
SseK2	110	123	GDFSKGQRKALGKL	SseK2 + YM155	10 s (23 C)	0.97	0.103568	4.59
SseK2	110	123	GDFSKGQRKALGKL	SseK2 + YM155	100 s (23 C)	1.40	0.158235	4.56
SseK2	110	123	GDFSKGQRKALGKL	SseK2 + YM155	1,000 s (23 C)	3.04	0.134047	4.55
SseK2	110	123	GDFSKGQRKALGKL	SseK2 + YM155	10,000 s (23 C)	3.79	0.075468	4.56
SseK2	110	123	GDFSKGQRKALGKL	SseK2 + YM155	12 h (28 C)	4.65	ND	4.55
SseK2	110	123	GDFSKGQRKALGKL	SseK2 alone	10 s (23 C)	0.98	0.070184	4.58
SseK2	110	123	GDFSKGQRKALGKL	SseK2 alone	100 s (23 C)	1.41	0.070822	4.57
SseK2	110	123	GDFSKGQRKALGKL	SseK2 alone	1,000 s (23 C)	2.85	0.085293	4.57
SseK2	110	123	GDFSKGQRKALGKL	SseK2 alone	10,000 s (23 C)	3.81	0.094294	4.57
SseK2	110	123	GDFSKGQRKALGKL	SseK2 alone	12 h (28 C)	4.81	0.070524	4.55
SseK2	110	126	GDFSKGQRKALGKLEQN	SseK2 + YM155	10 s (23 C)	1.44	0.070483	4.49
SseK2	110	126	GDFSKGQRKALGKLEQN	SseK2 + YM155	100 s (23 C)	2.63	0.041405	4.46
SseK2	110	126	GDFSKGQRKALGKLEQN	SseK2 + YM155	1,000 s (23 C)	5.13	0.076262	4.45
SseK2	110	126	GDFSKGQRKALGKLEQN	SseK2 + YM155	10,000 s (23 C)	6.69	0.081713	4.45
SseK2	110	126	GDFSKGQRKALGKLEQN	SseK2 + YM155	12 h (28 C)	8.32	0.027733	4.44
SseK2	110	126	GDFSKGQRKALGKLEQN	SseK2 alone	10 s (23 C)	1.43	0.026813	4.49
SseK2	110	126	GDFSKGQRKALGKLEQN	SseK2 alone	100 s (23 C)	2.61	0.047349	4.47
SseK2	110	126	GDFSKGQRKALGKLEQN	SseK2 alone	1,000 s (23 C)	5.25	0.115437	4.47
SseK2	110	126	GDFSKGQRKALGKLEQN	SseK2 alone	10,000 s (23 C)	6.70	0.072392	4.46
SseK2	110	126	GDFSKGQRKALGKLEQN	SseK2 alone	12 h (28 C)	8.31	0.074792	4.44
SseK2	113	138	SKGQRKALGKLEQNYRNIKVIYNSDL	SseK2 + YM155	10 s (23 C)	1.94	0.062194	5.70

SseK2	113	138	SKGQRKALGKLEQNYRNIKVIYNSDL	SseK2 + YM155	100 s (23 C)	3.40	0.044752	5.68
SseK2	113	138	SKGQRKALGKLEQNYRNIKVIYNSDL	SseK2 + YM155	1,000 s (23 C)	6.60	0.046162	5.68
SseK2	113	138	SKGQRKALGKLEQNYRNIKVIYNSDL	SseK2 + YM155	10,000 s (23 C)	9.74	0.024397	5.66
SseK2	113	138	SKGQRKALGKLEQNYRNIKVIYNSDL	SseK2 + YM155	12 h (28 C)	13.58	0.095287	5.64
SseK2	113	138	SKGQRKALGKLEQNYRNIKVIYNSDL	SseK2 alone	10 s (23 C)	2.02	0.125106	5.72
SseK2	113	138	SKGQRKALGKLEQNYRNIKVIYNSDL	SseK2 alone	100 s (23 C)	3.59	0.169489	5.70
SseK2	113	138	SKGQRKALGKLEQNYRNIKVIYNSDL	SseK2 alone	1,000 s (23 C)	6.66	0.166515	5.70
SseK2	113	138	SKGQRKALGKLEQNYRNIKVIYNSDL	SseK2 alone	10,000 s (23 C)	9.86	0.145925	5.69
SseK2	113	138	SKGQRKALGKLEQNYRNIKVIYNSDL	SseK2 alone	12 h (28 C)	13.60	0.082668	5.67
SseK2	120	124	LGKLE	SseK2 + YM155	10 s (23 C)	0.14	0.026931	4.12
SseK2	120	124	LGKLE	SseK2 + YM155	100 s (23 C)	0.15	0.029139	4.09
SseK2	120	124	LGKLE	SseK2 + YM155	1,000 s (23 C)	0.48	0.030326	4.09
SseK2	120	124	LGKLE	SseK2 + YM155	10,000 s (23 C)	1.51	0.024537	4.10
SseK2	120	124	LGKLE	SseK2 + YM155	12 h (28 C)	1.94	0.00633	4.09
SseK2	120	124	LGKLE	SseK2 alone	10 s (23 C)	0.12	0.02501	4.11
SseK2	120	124	LGKLE	SseK2 alone	100 s (23 C)	0.16	0.013453	4.09
SseK2	120	124	LGKLE	SseK2 alone	1,000 s (23 C)	0.44	0.003408	4.10
SseK2	120	124	LGKLE	SseK2 alone	10,000 s (23 C)	1.49	0.043111	4.09
SseK2	120	124	LGKLE	SseK2 alone	12 h (28 C)	1.91	0.020905	4.09
SseK2	127	137	YRNIKVIYNSD	SseK2 + YM155	10 s (23 C)	0.73	0.153432	4.77
SseK2	127	137	YRNIKVIYNSD	SseK2 + YM155	100 s (23 C)	1.14	0.014269	4.75
SseK2	127	137	YRNIKVIYNSD	SseK2 + YM155	1,000 s (23 C)	2.06	0.05428	4.75
SseK2	127	137	YRNIKVIYNSD	SseK2 + YM155	10,000 s (23 C)	3.29	0.038057	4.75
SseK2	127	137	YRNIKVIYNSD	SseK2 + YM155	12 h (28 C)	4.50	0.033809	4.74
SseK2	127	137	YRNIKVIYNSD	SseK2 alone	10 s (23 C)	0.73	0.124745	4.78
SseK2	127	137	YRNIKVIYNSD	SseK2 alone	100 s (23 C)	1.26	0.142213	4.76
SseK2	127	137	YRNIKVIYNSD	SseK2 alone	1,000 s (23 C)	2.12	0.093575	4.76
SseK2	127	137	YRNIKVIYNSD	SseK2 alone	10,000 s (23 C)	3.38	0.077488	4.76

SseK2	127	137	YRNIKVIYNSD	SseK2 alone	12 h (28 C)	4.45	0.042261	4.75
SseK2	127	138	YRNIKVIYNSDL	SseK2 + YM155	10 s (23 C)	0.59	0.028559	5.75
SseK2	127	138	YRNIKVIYNSDL	SseK2 + YM155	100 s (23 C)	0.95	0.020879	5.73
SseK2	127	138	YRNIKVIYNSDL	SseK2 + YM155	1,000 s (23 C)	1.80	0.082528	5.73
SseK2	127	138	YRNIKVIYNSDL	SseK2 + YM155	10,000 s (23 C)	3.14	0.014503	5.73
SseK2	127	138	YRNIKVIYNSDL	SseK2 + YM155	12 h (28 C)	5.01	0.0077	5.73
SseK2	127	138	YRNIKVIYNSDL	SseK2 alone	10 s (23 C)	0.65	0.045125	5.78
SseK2	127	138	YRNIKVIYNSDL	SseK2 alone	100 s (23 C)	1.07	0.111869	5.77
SseK2	127	138	YRNIKVIYNSDL	SseK2 alone	1,000 s (23 C)	1.82	0.120361	5.77
SseK2	127	138	YRNIKVIYNSDL	SseK2 alone	10,000 s (23 C)	3.26	0.154534	5.78
SseK2	127	138	YRNIKVIYNSDL	SseK2 alone	12 h (28 C)	4.97	0.005461	5.77
SseK2	130	138	IKVIYNSDL	SseK2 + YM155	10 s (23 C)	0.60	0.144325	5.94
SseK2	130	138	IKVIYNSDL	SseK2 + YM155	100 s (23 C)	0.74	0.01412	5.92
SseK2	130	138	IKVIYNSDL	SseK2 + YM155	1,000 s (23 C)	1.29	0.054218	5.92
SseK2	130	138	IKVIYNSDL	SseK2 + YM155	10,000 s (23 C)	2.56	0.017503	5.92
SseK2	130	138	IKVIYNSDL	SseK2 + YM155	12 h (28 C)	4.14	0.051295	5.92
SseK2	130	138	IKVIYNSDL	SseK2 alone	10 s (23 C)	0.57	0.028135	5.98
SseK2	130	138	IKVIYNSDL	SseK2 alone	100 s (23 C)	0.80	0.029186	5.97
SseK2	130	138	IKVIYNSDL	SseK2 alone	1,000 s (23 C)	1.33	0.091505	5.98
SseK2	130	138	IKVIYNSDL	SseK2 alone	10,000 s (23 C)	2.71	0.135261	5.97
SseK2	130	138	IKVIYNSDL	SseK2 alone	12 h (28 C)	4.17	0.018446	5.97
SseK2	133	138	IYNSDL	SseK2 + YM155	10 s (23 C)	0.58	0.125033	5.55
SseK2	133	138	IYNSDL	SseK2 + YM155	100 s (23 C)	0.70	0.018412	5.51
SseK2	133	138	IYNSDL	SseK2 + YM155	1,000 s (23 C)	1.11	0.032029	5.52
SseK2	133	138	IYNSDL	SseK2 + YM155	10,000 s (23 C)	1.54	0.017795	5.52
SseK2	133	138	IYNSDL	SseK2 + YM155	12 h (28 C)	2.47	0.031872	5.52
SseK2	133	138	IYNSDL	SseK2 alone	10 s (23 C)	0.63	0.050208	5.56
SseK2	133	138	IYNSDL	SseK2 alone	100 s (23 C)	0.87	0.03559	5.55

SseK2	133	138	IYNSDL	SseK2 alone	1,000 s (23 C)	1.29	0.062436	5.55
SseK2	133	138	IYNSDL	SseK2 alone	10,000 s (23 C)	1.75	0.073649	5.55
SseK2	133	138	IYNSDL	SseK2 alone	12 h (28 C)	2.58	0.006022	5.54
SseK2	139	149	NYSMYDKKLTT	SseK2 + YM155	10 s (23 C)	1.54	0.008824	4.87
SseK2	139	149	NYSMYDKKLTT	SseK2 + YM155	100 s (23 C)	2.20	0.022432	4.85
SseK2	139	149	NYSMYDKKLTT	SseK2 + YM155	1,000 s (23 C)	4.13	0.046098	4.85
SseK2	139	149	NYSMYDKKLTT	SseK2 + YM155	10,000 s (23 C)	5.68	0.022219	4.85
SseK2	139	149	NYSMYDKKLTT	SseK2 + YM155	12 h (28 C)	5.81	0.054191	4.85
SseK2	139	149	NYSMYDKKLTT	SseK2 alone	10 s (23 C)	1.59	0.05843	4.88
SseK2	139	149	NYSMYDKKLTT	SseK2 alone	100 s (23 C)	2.30	0.060583	4.87
SseK2	139	149	NYSMYDKKLTT	SseK2 alone	1,000 s (23 C)	4.24	0.142366	4.87
SseK2	139	149	NYSMYDKKLTT	SseK2 alone	10,000 s (23 C)	5.72	0.048459	4.87
SseK2	139	149	NYSMYDKKLTT	SseK2 alone	12 h (28 C)	5.85	0.069863	4.86
SseK2	139	151	NYSMYDKKLTTIY	SseK2 + YM155	10 s (23 C)	1.73	0.129069	6.12
SseK2	139	151	NYSMYDKKLTTIY	SseK2 + YM155	100 s (23 C)	2.43	0.092327	6.10
SseK2	139	151	NYSMYDKKLTTIY	SseK2 + YM155	1,000 s (23 C)	4.62	0.031335	6.09
SseK2	139	151	NYSMYDKKLTTIY	SseK2 + YM155	10,000 s (23 C)	7.51	0.046568	6.08
SseK2	139	151	NYSMYDKKLTTIY	SseK2 + YM155	12 h (28 C)	7.88	0.0843	6.08
SseK2	139	151	NYSMYDKKLTTIY	SseK2 alone	10 s (23 C)	1.73	0.069815	6.16
SseK2	139	151	NYSMYDKKLTTIY	SseK2 alone	100 s (23 C)	2.56	0.117498	6.15
SseK2	139	151	NYSMYDKKLTTIY	SseK2 alone	1,000 s (23 C)	4.63	0.012141	6.15
SseK2	139	151	NYSMYDKKLTTIY	SseK2 alone	10,000 s (23 C)	7.47	0.070803	6.15
SseK2	139	151	NYSMYDKKLTTIY	SseK2 alone	12 h (28 C)	7.88	0.062313	6.14
SseK2	141	149	SMYDKKLTT	SseK2 + YM155	10 s (23 C)	0.79	0.134468	4.20
SseK2	141	149	SMYDKKLTT	SseK2 + YM155	100 s (23 C)	1.07	0.011708	4.18
SseK2	141	149	SMYDKKLTT	SseK2 + YM155	1,000 s (23 C)	2.68	0.017215	4.17
SseK2	141	149	SMYDKKLTT	SseK2 + YM155	10,000 s (23 C)	4.02	0.058048	4.18
SseK2	141	149	SMYDKKLTT	SseK2 + YM155	12 h (28 C)	4.00	0.014189	4.17

SseK2	141	149	SMYDKKLTT	SseK2 alone	10 s (23 C)	0.75	0.032348	4.19
SseK2	141	149	SMYDKKLTT	SseK2 alone	100 s (23 C)	1.15	0.034332	4.18
SseK2	141	149	SMYDKKLTT	SseK2 alone	1,000 s (23 C)	2.75	0.178005	4.18
SseK2	141	149	SMYDKKLTT	SseK2 alone	10,000 s (23 C)	4.05	0.056944	4.18
SseK2	141	149	SMYDKKLTT	SseK2 alone	12 h (28 C)	4.05	0.002156	4.17
SseK2	143	149	YDKKLTT	SseK2 + YM155	10 s (23 C)	0.59	0.104704	2.51
SseK2	143	149	YDKKLTT	SseK2 + YM155	100 s (23 C)	0.86	0.014781	2.48
SseK2	143	149	YDKKLTT	SseK2 + YM155	1,000 s (23 C)	2.30	0.028431	2.49
SseK2	143	149	YDKKLTT	SseK2 + YM155	10,000 s (23 C)	2.99	0.028864	2.50
SseK2	143	149	YDKKLTT	SseK2 + YM155	12 h (28 C)	3.03	0.000516	2.49
SseK2	143	149	YDKKLTT	SseK2 alone	10 s (23 C)	0.53	0.021155	2.50
SseK2	143	149	YDKKLTT	SseK2 alone	100 s (23 C)	0.89	0.05744	2.49
SseK2	143	149	YDKKLTT	SseK2 alone	1,000 s (23 C)	2.36	0.105955	2.49
SseK2	143	149	YDKKLTT	SseK2 alone	10,000 s (23 C)	3.03	0.006398	2.50
SseK2	143	149	YDKKLTT	SseK2 alone	12 h (28 C)	3.03	0.020559	2.48
SseK2	143	151	YDKKLTTIY	SseK2 + YM155	10 s (23 C)	0.88	0.093633	5.18
SseK2	143	151	YDKKLTTIY	SseK2 + YM155	100 s (23 C)	1.15	0.026895	5.15
SseK2	143	151	YDKKLTTIY	SseK2 + YM155	1,000 s (23 C)	2.93	0.048572	5.15
SseK2	143	151	YDKKLTTIY	SseK2 + YM155	10,000 s (23 C)	4.64	0.034295	5.15
SseK2	143	151	YDKKLTTIY	SseK2 + YM155	12 h (28 C)	4.98	0.051559	5.15
SseK2	143	151	YDKKLTTIY	SseK2 alone	10 s (23 C)	0.86	0.006141	5.19
SseK2	143	151	YDKKLTTIY	SseK2 alone	100 s (23 C)	1.18	0.040907	5.18
SseK2	143	151	YDKKLTTIY	SseK2 alone	1,000 s (23 C)	2.93	0.106845	5.18
SseK2	143	151	YDKKLTTIY	SseK2 alone	10,000 s (23 C)	4.68	0.032193	5.18
SseK2	143	151	YDKKLTTIY	SseK2 alone	12 h (28 C)	5.02	0.0264	5.17
SseK2	144	149	DKKLTT	SseK2 + YM155	10 s (23 C)	0.73	0.133652	2.50
SseK2	144	149	DKKLTT	SseK2 + YM155	100 s (23 C)	1.05	0.040189	2.47
SseK2	144	149	DKKLTT	SseK2 + YM155	1,000 s (23 C)	2.31	0.006396	2.48

SseK2	144	149	DKKLTT	SseK2 + YM155	10,000 s (23 C)	3.03	0.034351	2.49
SseK2	144	149	DKKLTT	SseK2 + YM155	12 h (28 C)	3.10	0.009652	2.48
SseK2	144	149	DKKLTT	SseK2 alone	10 s (23 C)	0.72	0.024931	2.49
SseK2	144	149	DKKLTT	SseK2 alone	100 s (23 C)	1.05	0.102919	2.48
SseK2	144	149	DKKLTT	SseK2 alone	1,000 s (23 C)	2.33	0.068407	2.48
SseK2	144	149	DKKLTT	SseK2 alone	10,000 s (23 C)	3.03	0.022283	2.48
SseK2	144	149	DKKLTT	SseK2 alone	12 h (28 C)	3.14	0.020854	2.47
SseK2	149	156	TIYLENIT	SseK2 + YM155	10 s (23 C)	1.32	0.090364	6.13
SseK2	149	156	TIYLENIT	SseK2 + YM155	100 s (23 C)	1.87	0.072976	6.11
SseK2	149	156	TIYLENIT	SseK2 + YM155	1,000 s (23 C)	2.29	0.023633	6.11
SseK2	149	156	TIYLENIT	SseK2 + YM155	10,000 s (23 C)	2.44	0.097482	6.11
SseK2	149	156	TIYLENIT	SseK2 + YM155	12 h (28 C)	2.51	0.063959	6.11
SseK2	149	156	TIYLENIT	SseK2 alone	10 s (23 C)	1.35	0.111834	6.20
SseK2	149	156	TIYLENIT	SseK2 alone	100 s (23 C)	1.87	0.055015	6.20
SseK2	149	156	TIYLENIT	SseK2 alone	1,000 s (23 C)	2.36	0.013229	6.21
SseK2	149	156	TIYLENIT	SseK2 alone	10,000 s (23 C)	2.43	0.030401	6.21
SseK2	149	156	TIYLENIT	SseK2 alone	12 h (28 C)	2.48	0.037427	6.20
SseK2	150	158	IYLENITKL	SseK2 + YM155	10 s (23 C)	0.25	0.045797	7.21
SseK2	150	158	IYLENITKL	SseK2 + YM155	100 s (23 C)	0.77	0.01943	7.19
SseK2	150	158	IYLENITKL	SseK2 + YM155	1,000 s (23 C)	3.41	0.042935	7.19
SseK2	150	158	IYLENITKL	SseK2 + YM155	10,000 s (23 C)	5.39	0.043193	7.18
SseK2	150	158	IYLENITKL	SseK2 + YM155	12 h (28 C)	5.53	0.041189	7.18
SseK2	150	158	IYLENITKL	SseK2 alone	10 s (23 C)	0.23	0.012078	7.20
SseK2	150	158	IYLENITKL	SseK2 alone	100 s (23 C)	0.82	0.108682	7.19
SseK2	150	158	IYLENITKL	SseK2 alone	1,000 s (23 C)	3.34	0.013369	7.19
SseK2	150	158	IYLENITKL	SseK2 alone	10,000 s (23 C)	5.45	0.020904	7.18
SseK2	150	158	IYLENITKL	SseK2 alone	12 h (28 C)	5.63	0.002968	7.18
SseK2	150	170	IYLENITKLEAQSASERDEVL	SseK2 + YM155	10 s (23 C)	6.30	0.014739	7.10

SseK2	150	170	IYLENITKLEAQSASERDEVL	SseK2 + YM155	100 s (23 C)	8.45	0.037144	7.07
SseK2	150	170	IYLENITKLEAQSASERDEVL	SseK2 + YM155	1,000 s (23 C)	12.31	0.04412	7.07
SseK2	150	170	IYLENITKLEAQSASERDEVL	SseK2 + YM155	10,000 s (23 C)	14.23	0.037358	7.06
SseK2	150	170	IYLENITKLEAQSASERDEVL	SseK2 + YM155	12 h (28 C)	14.37	0.11354	7.06
SseK2	150	170	IYLENITKLEAQSASERDEVL	SseK2 alone	10 s (23 C)	6.50	0.175549	7.08
SseK2	150	170	IYLENITKLEAQSASERDEVL	SseK2 alone	100 s (23 C)	8.58	0.031472	7.08
SseK2	150	170	IYLENITKLEAQSASERDEVL	SseK2 alone	1,000 s (23 C)	12.38	0.010522	7.07
SseK2	150	170	IYLENITKLEAQSASERDEVL	SseK2 alone	10,000 s (23 C)	14.46	0.043473	7.06
SseK2	150	170	IYLENITKLEAQSASERDEVL	SseK2 alone	12 h (28 C)	14.67	0.186034	7.06
SseK2	152	158	LENITKL	SseK2 + YM155	10 s (23 C)	0.20	0.069058	5.75
SseK2	152	158	LENITKL	SseK2 + YM155	100 s (23 C)	0.58	0.019523	5.72
SseK2	152	158	LENITKL	SseK2 + YM155	1,000 s (23 C)	2.66	0.010893	5.73
SseK2	152	158	LENITKL	SseK2 + YM155	10,000 s (23 C)	3.59	0.018407	5.72
SseK2	152	158	LENITKL	SseK2 + YM155	12 h (28 C)	3.58	0.035898	5.72
SseK2	152	158	LENITKL	SseK2 alone	10 s (23 C)	0.19	0.006027	5.78
SseK2	152	158	LENITKL	SseK2 alone	100 s (23 C)	0.59	0.000775	5.77
SseK2	152	158	LENITKL	SseK2 alone	1,000 s (23 C)	2.69	0.029535	5.77
SseK2	152	158	LENITKL	SseK2 alone	10,000 s (23 C)	3.71	0.026014	5.77
SseK2	152	158	LENITKL	SseK2 alone	12 h (28 C)	3.72	0.011119	5.77
SseK2	152	167	LENITKLEAQSASERD	SseK2 + YM155	10 s (23 C)	5.08	0.027446	5.14
SseK2	152	167	LENITKLEAQSASERD	SseK2 + YM155	100 s (23 C)	6.13	0.037344	5.12
SseK2	152	167	LENITKLEAQSASERD	SseK2 + YM155	1,000 s (23 C)	9.30	0.009356	5.11
SseK2	152	167	LENITKLEAQSASERD	SseK2 + YM155	10,000 s (23 C)	10.29	0.042479	5.11
SseK2	152	167	LENITKLEAQSASERD	SseK2 + YM155	12 h (28 C)	10.28	0.061435	5.11
SseK2	152	167	LENITKLEAQSASERD	SseK2 alone	10 s (23 C)	5.06	0.034601	5.16
SseK2	152	167	LENITKLEAQSASERD	SseK2 alone	100 s (23 C)	6.19	0.034299	5.14
SseK2	152	167	LENITKLEAQSASERD	SseK2 alone	1,000 s (23 C)	9.39	0.123614	5.13
SseK2	152	167	LENITKLEAQSASERD	SseK2 alone	10,000 s (23 C)	10.36	0.020781	5.13

SseK2	152	167	LENITKLEAQSASERD	SseK2 alone	12 h (28 C)	10.39	0.003417	5.13
SseK2	153	158	ENITKL	SseK2 + YM155	10 s (23 C)	0.17	0.079019	4.99
SseK2	153	158	ENITKL	SseK2 + YM155	100 s (23 C)	0.45	0.006628	4.97
SseK2	153	158	ENITKL	SseK2 + YM155	1,000 s (23 C)	1.95	0.04152	4.97
SseK2	153	158	ENITKL	SseK2 + YM155	10,000 s (23 C)	2.64	0.036716	4.97
SseK2	153	158	ENITKL	SseK2 + YM155	12 h (28 C)	2.64	0.013276	4.97
SseK2	153	158	ENITKL	SseK2 alone	10 s (23 C)	0.15	0.014844	4.99
SseK2	153	158	ENITKL	SseK2 alone	100 s (23 C)	0.51	0.058072	4.97
SseK2	153	158	ENITKL	SseK2 alone	1,000 s (23 C)	2.03	0.114397	4.97
SseK2	153	158	ENITKL	SseK2 alone	10,000 s (23 C)	2.70	0.044672	4.97
SseK2	153	158	ENITKL	SseK2 alone	12 h (28 C)	2.68	0.002959	4.97
SseK2	153	165	ENITKLEAQSASE	SseK2 + YM155	10 s (23 C)	4.01	0.03554	4.68
SseK2	153	165	ENITKLEAQSASE	SseK2 + YM155	100 s (23 C)	4.98	0.033893	4.66
SseK2	153	165	ENITKLEAQSASE	SseK2 + YM155	1,000 s (23 C)	7.81	0.051441	4.65
SseK2	153	165	ENITKLEAQSASE	SseK2 + YM155	10,000 s (23 C)	8.70	0.019467	4.65
SseK2	153	165	ENITKLEAQSASE	SseK2 + YM155	12 h (28 C)	8.63	0.051967	4.65
SseK2	153	165	ENITKLEAQSASE	SseK2 alone	10 s (23 C)	4.02	0.018756	4.68
SseK2	153	165	ENITKLEAQSASE	SseK2 alone	100 s (23 C)	5.00	0.03238	4.67
SseK2	153	165	ENITKLEAQSASE	SseK2 alone	1,000 s (23 C)	7.89	0.107168	4.66
SseK2	153	165	ENITKLEAQSASE	SseK2 alone	10,000 s (23 C)	8.78	0.022633	4.66
SseK2	153	165	ENITKLEAQSASE	SseK2 alone	12 h (28 C)	8.64	0.048947	4.65
SseK2	153	170	ENITKLEAQSASERDEVL	SseK2 + YM155	10 s (23 C)	6.46	0.007471	5.76
SseK2	153	170	ENITKLEAQSASERDEVL	SseK2 + YM155	100 s (23 C)	8.55	0.008831	5.73
SseK2	153	170	ENITKLEAQSASERDEVL	SseK2 + YM155	1,000 s (23 C)	11.64	0.015676	5.72
SseK2	153	170	ENITKLEAQSASERDEVL	SseK2 + YM155	10,000 s (23 C)	12.45	0.05947	5.72
SseK2	153	170	ENITKLEAQSASERDEVL	SseK2 + YM155	12 h (28 C)	12.52	0.058837	5.72
SseK2	153	170	ENITKLEAQSASERDEVL	SseK2 alone	10 s (23 C)	6.59	0.181625	5.78
SseK2	153	170	ENITKLEAQSASERDEVL	SseK2 alone	100 s (23 C)	8.56	0.016249	5.77

SseK2	153	170	ENITKLEAQSASERDEVL	SseK2 alone	1,000 s (23 C)	11.69	0.090354	5.76
SseK2	153	170	ENITKLEAQSASERDEVL	SseK2 alone	10,000 s (23 C)	12.54	0.047487	5.76
SseK2	153	170	ENITKLEAQSASERDEVL	SseK2 alone	12 h (28 C)	12.57	0.067509	5.76
SseK2	155	165	ITKLEAQSASE	SseK2 + YM155	10 s (23 C)	3.72	0.105376	3.74
SseK2	155	165	ITKLEAQSASE	SseK2 + YM155	100 s (23 C)	4.35	0.024439	3.73
SseK2	155	165	ITKLEAQSASE	SseK2 + YM155	1,000 s (23 C)	5.98	0.012693	3.72
SseK2	155	165	ITKLEAQSASE	SseK2 + YM155	10,000 s (23 C)	6.18	0.051591	3.73
SseK2	155	165	ITKLEAQSASE	SseK2 + YM155	12 h (28 C)	6.25	0.049453	3.73
SseK2	155	165	ITKLEAQSASE	SseK2 alone	10 s (23 C)	3.71	0.029324	3.74
SseK2	155	165	ITKLEAQSASE	SseK2 alone	100 s (23 C)	4.57	0.146947	3.72
SseK2	155	165	ITKLEAQSASE	SseK2 alone	1,000 s (23 C)	6.25	0.082994	3.72
SseK2	155	165	ITKLEAQSASE	SseK2 alone	10,000 s (23 C)	6.40	0.030578	3.71
SseK2	155	165	ITKLEAQSASE	SseK2 alone	12 h (28 C)	6.42	0.074699	3.72
SseK2	155	170	ITKLEAQSASERDEVL	SseK2 + YM155	10 s (23 C)	6.21	0.037294	5.24
SseK2	155	170	ITKLEAQSASERDEVL	SseK2 + YM155	100 s (23 C)	7.97	0.027327	5.22
SseK2	155	170	ITKLEAQSASERDEVL	SseK2 + YM155	1,000 s (23 C)	9.87	0.028177	5.22
SseK2	155	170	ITKLEAQSASERDEVL	SseK2 + YM155	10,000 s (23 C)	10.05	0.06053	5.22
SseK2	155	170	ITKLEAQSASERDEVL	SseK2 + YM155	12 h (28 C)	10.10	0.118031	5.21
SseK2	155	170	ITKLEAQSASERDEVL	SseK2 alone	10 s (23 C)	6.23	0.03484	5.27
SseK2	155	170	ITKLEAQSASERDEVL	SseK2 alone	100 s (23 C)	8.03	0.016175	5.25
SseK2	155	170	ITKLEAQSASERDEVL	SseK2 alone	1,000 s (23 C)	10.06	0.138408	5.25
SseK2	155	170	ITKLEAQSASERDEVL	SseK2 alone	10,000 s (23 C)	10.23	0.059596	5.25
SseK2	155	170	ITKLEAQSASERDEVL	SseK2 alone	12 h (28 C)	10.27	0.129041	5.25
SseK2	159	170	EAQSASERDEVL	SseK2 + YM155	10 s (23 C)	4.90	0.111242	5.04
SseK2	159	170	EAQSASERDEVL	SseK2 + YM155	100 s (23 C)	5.82	0.023212	5.00
SseK2	159	170	EAQSASERDEVL	SseK2 + YM155	1,000 s (23 C)	6.15	0.030725	5.01
SseK2	159	170	EAQSASERDEVL	SseK2 + YM155	10,000 s (23 C)	6.11	0.030189	5.01
SseK2	159	170	EAQSASERDEVL	SseK2 + YM155	12 h (28 C)	6.13	0.065374	5.01

SseK2	159	170	EAQSASERDEVL	SseK2 alone	10 s (23 C)	5.05	0.162113	5.04
SseK2	159	170	EAQSASERDEVL	SseK2 alone	100 s (23 C)	6.08	0.069348	5.01
SseK2	159	170	EAQSASERDEVL	SseK2 alone	1,000 s (23 C)	6.32	0.032824	5.02
SseK2	159	170	EAQSASERDEVL	SseK2 alone	10,000 s (23 C)	6.29	0.021368	5.02
SseK2	159	170	EAQSASERDEVL	SseK2 alone	12 h (28 C)	6.32	0.080625	5.01
SseK2	164	170	SERDEVL	SseK2 + YM155	10 s (23 C)	2.03	0.078811	4.98
SseK2	164	170	SERDEVL	SseK2 + YM155	100 s (23 C)	2.93	0.041702	4.95
SseK2	164	170	SERDEVL	SseK2 + YM155	1,000 s (23 C)	3.21	0.018036	4.96
SseK2	164	170	SERDEVL	SseK2 + YM155	10,000 s (23 C)	3.15	0.059153	4.96
SseK2	164	170	SERDEVL	SseK2 + YM155	12 h (28 C)	3.12	0.003181	4.96
SseK2	164	170	SERDEVL	SseK2 alone	10 s (23 C)	2.12	0.006452	4.99
SseK2	164	170	SERDEVL	SseK2 alone	100 s (23 C)	3.05	0.078845	4.95
SseK2	164	170	SERDEVL	SseK2 alone	1,000 s (23 C)	3.40	0.070657	4.96
SseK2	164	170	SERDEVL	SseK2 alone	10,000 s (23 C)	3.27	0.049975	4.96
SseK2	164	170	SERDEVL	SseK2 alone	12 h (28 C)	3.15	0.068245	4.95
SseK2	166	190	RDEVLLNGVKKSLEDVLKNNPEETL	SseK2 + YM155	10 s (23 C)	2.10	0.027905	7.48
SseK2	166	190	RDEVLLNGVKKSLEDVLKNNPEETL	SseK2 + YM155	100 s (23 C)	6.03	0.042214	7.44
SseK2	166	190	RDEVLLNGVKKSLEDVLKNNPEETL	SseK2 + YM155	1,000 s (23 C)	12.55	0.036846	7.42
SseK2	166	190	RDEVLLNGVKKSLEDVLKNNPEETL	SseK2 + YM155	10,000 s (23 C)	15.88	0.041346	7.40
SseK2	166	190	RDEVLLNGVKKSLEDVLKNNPEETL	SseK2 + YM155	12 h (28 C)	16.05	0.092946	7.40
SseK2	166	190	RDEVLLNGVKKSLEDVLKNNPEETL	SseK2 alone	10 s (23 C)	2.14	0.033984	7.47
SseK2	166	190	RDEVLLNGVKKSLEDVLKNNPEETL	SseK2 alone	100 s (23 C)	6.04	0.025932	7.45
SseK2	166	190	RDEVLLNGVKKSLEDVLKNNPEETL	SseK2 alone	1,000 s (23 C)	12.37	0.038179	7.42
SseK2	166	190	RDEVLLNGVKKSLEDVLKNNPEETL	SseK2 alone	10,000 s (23 C)	15.96	0.096107	7.40
SseK2	166	190	RDEVLLNGVKKSLEDVLKNNPEETL	SseK2 alone	12 h (28 C)	16.15	0.076208	7.40
SseK2	168	179	EVLLNGVKKSLE	SseK2 + YM155	10 s (23 C)	0.64	0.091241	5.51
SseK2	168	179	EVLLNGVKKSLE	SseK2 + YM155	100 s (23 C)	2.82	0.029886	5.48
SseK2	168	179	EVLLNGVKKSLE	SseK2 + YM155	1,000 s (23 C)	6.93	0.029875	5.46

SseK2	168	179	EVLLNGVKKSLE	SseK2 + YM155	10,000 s (23 C)	7.82	0.019056	5.46
SseK2	168	179	EVLLNGVKKSLE	SseK2 + YM155	12 h (28 C)	7.80	0.064339	5.46
SseK2	168	179	EVLLNGVKKSLE	SseK2 alone	10 s (23 C)	0.64	0.05698	5.54
SseK2	168	179	EVLLNGVKKSLE	SseK2 alone	100 s (23 C)	2.84	0.042683	5.52
SseK2	168	179	EVLLNGVKKSLE	SseK2 alone	1,000 s (23 C)	6.94	0.03226	5.50
SseK2	168	179	EVLLNGVKKSLE	SseK2 alone	10,000 s (23 C)	7.87	0.038161	5.49
SseK2	168	179	EVLLNGVKKSLE	SseK2 alone	12 h (28 C)	7.80	0.05752	5.48
SseK2	168	182	EVLLNGVKKSLEDVL	SseK2 + YM155	10 s (23 C)	0.73	0.114618	7.50
SseK2	168	182	EVLLNGVKKSLEDVL	SseK2 + YM155	100 s (23 C)	2.99	0.024742	7.47
SseK2	168	182	EVLLNGVKKSLEDVL	SseK2 + YM155	1,000 s (23 C)	7.98	0.073787	7.44
SseK2	168	182	EVLLNGVKKSLEDVL	SseK2 + YM155	10,000 s (23 C)	10.18	0.024392	7.43
SseK2	168	182	EVLLNGVKKSLEDVL	SseK2 + YM155	12 h (28 C)	10.35	0.054252	7.43
SseK2	168	182	EVLLNGVKKSLEDVL	SseK2 alone	10 s (23 C)	0.73	0.093569	7.49
SseK2	168	182	EVLLNGVKKSLEDVL	SseK2 alone	100 s (23 C)	2.98	0.007469	7.48
SseK2	168	182	EVLLNGVKKSLEDVL	SseK2 alone	1,000 s (23 C)	7.87	0.076363	7.44
SseK2	168	182	EVLLNGVKKSLEDVL	SseK2 alone	10,000 s (23 C)	10.22	0.075168	7.43
SseK2	168	182	EVLLNGVKKSLEDVL	SseK2 alone	12 h (28 C)	10.38	0.03527	7.43
SseK2	168	190	EVLLNGVKKSLEDVLKNNPEETL	SseK2 + YM155	10 s (23 C)	1.87	0.008245	7.55
SseK2	168	190	EVLLNGVKKSLEDVLKNNPEETL	SseK2 + YM155	100 s (23 C)	5.41	0.044295	7.51
SseK2	168	190	EVLLNGVKKSLEDVLKNNPEETL	SseK2 + YM155	1,000 s (23 C)	11.96	0.066705	7.49
SseK2	168	190	EVLLNGVKKSLEDVLKNNPEETL	SseK2 + YM155	10,000 s (23 C)	15.37	0.045489	7.48
SseK2	168	190	EVLLNGVKKSLEDVLKNNPEETL	SseK2 + YM155	12 h (28 C)	15.59	0.077811	7.48
SseK2	168	190	EVLLNGVKKSLEDVLKNNPEETL	SseK2 alone	10 s (23 C)	1.86	0.026829	7.55
SseK2	168	190	EVLLNGVKKSLEDVLKNNPEETL	SseK2 alone	100 s (23 C)	5.32	0.004804	7.53
SseK2	168	190	EVLLNGVKKSLEDVLKNNPEETL	SseK2 alone	1,000 s (23 C)	11.81	0.112316	7.49
SseK2	168	190	EVLLNGVKKSLEDVLKNNPEETL	SseK2 alone	10,000 s (23 C)	15.43	0.005751	7.48
SseK2	168	190	EVLLNGVKKSLEDVLKNNPEETL	SseK2 alone	12 h (28 C)	15.64	0.105185	7.47
SseK2	171	179	LNGVKKSLE	SseK2 + YM155	10 s (23 C)	0.46	0.125968	3.12

SseK2	171	179	LNGVKKSLE	SseK2 + YM155	100 s (23 C)	1.05	0.03602	3.40
SseK2	171	179	LNGVKKSLE	SseK2 + YM155	1,000 s (23 C)	3.38	0.049109	3.37
SseK2	171	179	LNGVKKSLE	SseK2 + YM155	10,000 s (23 C)	4.13	0.049753	3.40
SseK2	171	179	LNGVKKSLE	SseK2 + YM155	12 h (28 C)	4.08	0.001469	3.40
SseK2	171	179	LNGVKKSLE	SseK2 alone	10 s (23 C)	0.38	0.037849	3.41
SseK2	171	179	LNGVKKSLE	SseK2 alone	100 s (23 C)	1.19	0.136404	3.39
SseK2	171	179	LNGVKKSLE	SseK2 alone	1,000 s (23 C)	3.59	0.207965	3.37
SseK2	171	179	LNGVKKSLE	SseK2 alone	10,000 s (23 C)	4.20	0.012116	3.38
SseK2	171	179	LNGVKKSLE	SseK2 alone	12 h (28 C)	4.25	0.057834	3.38
SseK2	171	180	LNGVKKSLED	SseK2 + YM155	10 s (23 C)	0.37	0.059752	3.24
SseK2	171	180	LNGVKKSLED	SseK2 + YM155	100 s (23 C)	0.97	0.041864	3.54
SseK2	171	180	LNGVKKSLED	SseK2 + YM155	1,000 s (23 C)	3.61	0.046971	3.52
SseK2	171	180	LNGVKKSLED	SseK2 + YM155	10,000 s (23 C)	4.46	0.054613	3.54
SseK2	171	180	LNGVKKSLED	SseK2 + YM155	12 h (28 C)	4.42	0.020678	3.53
SseK2	171	180	LNGVKKSLED	SseK2 alone	10 s (23 C)	0.39	0.014608	3.55
SseK2	171	180	LNGVKKSLED	SseK2 alone	100 s (23 C)	1.17	0.13131	3.53
SseK2	171	180	LNGVKKSLED	SseK2 alone	1,000 s (23 C)	3.74	0.050052	3.52
SseK2	171	180	LNGVKKSLED	SseK2 alone	10,000 s (23 C)	4.76	0.124313	3.18
SseK2	171	180	LNGVKKSLED	SseK2 alone	12 h (28 C)	4.68	0.043112	3.52
SseK2	171	182	LNGVKKSLEDVL	SseK2 + YM155	10 s (23 C)	0.36	0.031026	6.13
SseK2	171	182	LNGVKKSLEDVL	SseK2 + YM155	100 s (23 C)	1.07	0.033163	6.10
SseK2	171	182	LNGVKKSLEDVL	SseK2 + YM155	1,000 s (23 C)	4.34	0.045711	6.09
SseK2	171	182	LNGVKKSLEDVL	SseK2 + YM155	10,000 s (23 C)	6.29	0.026724	6.08
SseK2	171	182	LNGVKKSLEDVL	SseK2 + YM155	12 h (28 C)	6.50	0.057093	6.08
SseK2	171	182	LNGVKKSLEDVL	SseK2 alone	10 s (23 C)	0.26	0.080602	6.17
SseK2	171	182	LNGVKKSLEDVL	SseK2 alone	100 s (23 C)	0.98	0.034294	6.16
SseK2	171	182	LNGVKKSLEDVL	SseK2 alone	1,000 s (23 C)	4.37	0.003468	6.13
SseK2	171	182	LNGVKKSLEDVL	SseK2 alone	10,000 s (23 C)	6.45	0.020142	6.13

SseK2	171	182	LNGVKKSLEDVL	SseK2 alone	12 h (28 C)	6.72	0.038404	6.12
SseK2	171	188	LNGVKKSLEDVLKNNPEE	SseK2 + YM155	10 s (23 C)	1.55	0.149768	5.70
SseK2	171	188	LNGVKKSLEDVLKNNPEE	SseK2 + YM155	100 s (23 C)	3.06	0.052969	5.70
SseK2	171	188	LNGVKKSLEDVLKNNPEE	SseK2 + YM155	1,000 s (23 C)	7.53	0.056981	5.66
SseK2	171	188	LNGVKKSLEDVLKNNPEE	SseK2 + YM155	10,000 s (23 C)	10.52	0.06283	5.64
SseK2	171	188	LNGVKKSLEDVLKNNPEE	SseK2 + YM155	12 h (28 C)	10.74	0.098257	5.64
SseK2	171	188	LNGVKKSLEDVLKNNPEE	SseK2 alone	10 s (23 C)	1.52	0.164782	5.72
SseK2	171	188	LNGVKKSLEDVLKNNPEE	SseK2 alone	100 s (23 C)	3.31	0.109964	5.71
SseK2	171	188	LNGVKKSLEDVLKNNPEE	SseK2 alone	1,000 s (23 C)	7.42	0.025997	5.68
SseK2	171	188	LNGVKKSLEDVLKNNPEE	SseK2 alone	10,000 s (23 C)	10.55	0.093907	5.67
SseK2	171	188	LNGVKKSLEDVLKNNPEE	SseK2 alone	12 h (28 C)	10.73	0.037277	5.67
SseK2	171	190	LNGVKKSLEDVLKNNPEETL	SseK2 + YM155	10 s (23 C)	1.50	0.03128	6.32
SseK2	171	190	LNGVKKSLEDVLKNNPEETL	SseK2 + YM155	100 s (23 C)	3.48	0.031478	6.30
SseK2	171	190	LNGVKKSLEDVLKNNPEETL	SseK2 + YM155	1,000 s (23 C)	8.70	0.025075	6.27
SseK2	171	190	LNGVKKSLEDVLKNNPEETL	SseK2 + YM155	10,000 s (23 C)	11.98	0.061815	6.25
SseK2	171	190	LNGVKKSLEDVLKNNPEETL	SseK2 + YM155	12 h (28 C)	12.12	0.107256	6.26
SseK2	171	190	LNGVKKSLEDVLKNNPEETL	SseK2 alone	10 s (23 C)	1.63	0.18743	6.35
SseK2	171	190	LNGVKKSLEDVLKNNPEETL	SseK2 alone	100 s (23 C)	3.45	0.029252	6.35
SseK2	171	190	LNGVKKSLEDVLKNNPEETL	SseK2 alone	1,000 s (23 C)	8.44	0.033795	6.33
SseK2	171	190	LNGVKKSLEDVLKNNPEETL	SseK2 alone	10,000 s (23 C)	12.03	0.100097	6.31
SseK2	171	190	LNGVKKSLEDVLKNNPEETL	SseK2 alone	12 h (28 C)	12.25	0.073802	6.31
SseK2	172	179	NGVKKSLE	SseK2 + YM155	10 s (23 C)	0.39	0.098892	3.16
SseK2	172	179	NGVKKSLE	SseK2 + YM155	100 s (23 C)	1.07	0.029816	3.40
SseK2	172	179	NGVKKSLE	SseK2 + YM155	1,000 s (23 C)	3.50	0.037246	3.38
SseK2	172	179	NGVKKSLE	SseK2 + YM155	10,000 s (23 C)	4.24	0.025401	3.40
SseK2	172	179	NGVKKSLE	SseK2 + YM155	12 h (28 C)	4.22	0.037968	3.40
SseK2	172	179	NGVKKSLE	SseK2 alone	10 s (23 C)	0.41	0.01	3.41
SseK2	172	179	NGVKKSLE	SseK2 alone	100 s (23 C)	1.20	0.129233	3.39

SseK2	172	179	NGVKKSLE	SseK2 alone	1,000 s (23 C)	3.65	0.164228	3.37
SseK2	172	179	NGVKKSLE	SseK2 alone	10,000 s (23 C)	4.31	0.0023	3.38
SseK2	172	179	NGVKKSLE	SseK2 alone	12 h (28 C)	4.36	0.028256	3.38
SseK2	173	179	GVKKSLE	SseK2 + YM155	10 s (23 C)	0.23	0.019211	3.17
SseK2	173	179	GVKKSLE	SseK2 + YM155	100 s (23 C)	0.83	0.010372	3.40
SseK2	173	179	GVKKSLE	SseK2 + YM155	1,000 s (23 C)	3.22	0.07284	3.38
SseK2	173	179	GVKKSLE	SseK2 + YM155	10,000 s (23 C)	3.78	0.016276	3.40
SseK2	173	179	GVKKSLE	SseK2 + YM155	12 h (28 C)	3.97	0.115124	3.40
SseK2	173	179	GVKKSLE	SseK2 alone	10 s (23 C)	0.24	0.014891	3.42
SseK2	173	179	GVKKSLE	SseK2 alone	100 s (23 C)	0.83	0.048544	3.38
SseK2	173	179	GVKKSLE	SseK2 alone	1,000 s (23 C)	3.26	0.01775	3.37
SseK2	173	179	GVKKSLE	SseK2 alone	10,000 s (23 C)	4.04	0.063733	3.17
SseK2	173	179	GVKKSLE	SseK2 alone	12 h (28 C)	4.10	0.05172	3.38
SseK2	176	194	KSLEDVLKNNPEETLISSH	SseK2 + YM155	10 s (23 C)	4.02	0.154278	5.66
SseK2	176	194	KSLEDVLKNNPEETLISSH	SseK2 + YM155	100 s (23 C)	5.38	0.121459	5.62
SseK2	176	194	KSLEDVLKNNPEETLISSH	SseK2 + YM155	1,000 s (23 C)	6.17	0.015184	5.64
SseK2	176	194	KSLEDVLKNNPEETLISSH	SseK2 + YM155	10,000 s (23 C)	6.70	0.04668	5.64
SseK2	176	194	KSLEDVLKNNPEETLISSH	SseK2 + YM155	12 h (28 C)	7.45	0.164329	5.64
SseK2	176	194	KSLEDVLKNNPEETLISSH	SseK2 alone	10 s (23 C)	3.99	0.141926	5.68
SseK2	176	194	KSLEDVLKNNPEETLISSH	SseK2 alone	100 s (23 C)	5.50	0.076779	5.67
SseK2	176	194	KSLEDVLKNNPEETLISSH	SseK2 alone	1,000 s (23 C)	6.18	0.124089	5.67
SseK2	176	194	KSLEDVLKNNPEETLISSH	SseK2 alone	10,000 s (23 C)	6.79	0.069764	5.68
SseK2	176	194	KSLEDVLKNNPEETLISSH	SseK2 alone	12 h (28 C)	7.49	0.020738	5.67
SseK2	180	190	DVLKNNPEETL	SseK2 + YM155	10 s (23 C)	1.14	0.018601	5.53
SseK2	180	190	DVLKNNPEETL	SseK2 + YM155	100 s (23 C)	2.09	0.019497	5.50
SseK2	180	190	DVLKNNPEETL	SseK2 + YM155	1,000 s (23 C)	3.67	0.054206	5.50
SseK2	180	190	DVLKNNPEETL	SseK2 + YM155	10,000 s (23 C)	5.18	0.027451	5.50
SseK2	180	190	DVLKNNPEETL	SseK2 + YM155	12 h (28 C)	5.23	0.058927	5.50

SseK2	180	190	DVLKNNPEETL	SseK2 alone	10 s (23 C)	1.20	0.077367	5.56
SseK2	180	190	DVLKNNPEETL	SseK2 alone	100 s (23 C)	2.15	0.045791	5.55
SseK2	180	190	DVLKNNPEETL	SseK2 alone	1,000 s (23 C)	3.57	0.057247	5.55
SseK2	180	190	DVLKNNPEETL	SseK2 alone	10,000 s (23 C)	5.19	0.037408	5.55
SseK2	180	190	DVLKNNPEETL	SseK2 alone	12 h (28 C)	5.29	0.022062	5.54
SseK2	181	190	VLKNNPEETL	SseK2 + YM155	10 s (23 C)	1.00	0.101112	5.00
SseK2	181	190	VLKNNPEETL	SseK2 + YM155	100 s (23 C)	1.99	0.027931	4.98
SseK2	181	190	VLKNNPEETL	SseK2 + YM155	1,000 s (23 C)	3.51	0.046209	4.98
SseK2	181	190	VLKNNPEETL	SseK2 + YM155	10,000 s (23 C)	4.58	0.006615	4.98
SseK2	181	190	VLKNNPEETL	SseK2 + YM155	12 h (28 C)	4.57	0.041765	4.98
SseK2	181	190	VLKNNPEETL	SseK2 alone	10 s (23 C)	1.03	0.098693	5.01
SseK2	181	190	VLKNNPEETL	SseK2 alone	100 s (23 C)	2.11	0.050843	4.99
SseK2	181	190	VLKNNPEETL	SseK2 alone	1,000 s (23 C)	3.63	0.193137	4.99
SseK2	181	190	VLKNNPEETL	SseK2 alone	10,000 s (23 C)	4.67	0.063806	4.99
SseK2	181	190	VLKNNPEETL	SseK2 alone	12 h (28 C)	4.65	0.010189	4.99
SseK2	183	190	KNNPEETL	SseK2 + YM155	10 s (23 C)	0.90	0.007245	4.49
SseK2	183	190	KNNPEETL	SseK2 + YM155	100 s (23 C)	1.51	0.009379	4.47
SseK2	183	190	KNNPEETL	SseK2 + YM155	1,000 s (23 C)	2.66	0.005237	4.47
SseK2	183	190	KNNPEETL	SseK2 + YM155	10,000 s (23 C)	3.43	0.031526	4.47
SseK2	183	190	KNNPEETL	SseK2 + YM155	12 h (28 C)	3.44	0.03767	4.47
SseK2	183	190	KNNPEETL	SseK2 alone	10 s (23 C)	1.03	0.08906	4.48
SseK2	183	190	KNNPEETL	SseK2 alone	100 s (23 C)	1.63	0.048301	4.47
SseK2	183	190	KNNPEETL	SseK2 alone	1,000 s (23 C)	2.75	0.136256	4.47
SseK2	183	190	KNNPEETL	SseK2 alone	10,000 s (23 C)	3.56	0.011343	4.47
SseK2	183	190	KNNPEETL	SseK2 alone	12 h (28 C)	3.51	0.024533	4.46
SseK2	185	190	NPEETL	SseK2 + YM155	10 s (23 C)	0.51	0.106217	4.50
SseK2	185	190	NPEETL	SseK2 + YM155	100 s (23 C)	0.72	0.022569	4.47
SseK2	185	190	NPEETL	SseK2 + YM155	1,000 s (23 C)	1.63	0.049427	4.47

SseK2	185	190	NPEETL	SseK2 + YM155	10,000 s (23 C)	2.14	0.052768	4.48
SseK2	185	190	NPEETL	SseK2 + YM155	12 h (28 C)	2.16	0.009022	4.47
SseK2	185	190	NPEETL	SseK2 alone	10 s (23 C)	0.56	0.014213	4.48
SseK2	185	190	NPEETL	SseK2 alone	100 s (23 C)	0.77	0.024528	4.47
SseK2	185	190	NPEETL	SseK2 alone	1,000 s (23 C)	1.69	0.027663	4.47
SseK2	185	190	NPEETL	SseK2 alone	10,000 s (23 C)	2.18	0.030066	4.47
SseK2	185	190	NPEETL	SseK2 alone	12 h (28 C)	2.23	0.023269	4.46
SseK2	190	200	LISSHNKDKGH	SseK2 + YM155	10 s (23 C)	1.46	0.017427	3.44
SseK2	190	200	LISSHNKDKGH	SseK2 + YM155	100 s (23 C)	1.70	0.067553	3.40
SseK2	190	200	LISSHNKDKGH	SseK2 + YM155	1,000 s (23 C)	1.89	0.001065	3.41
SseK2	190	200	LISSHNKDKGH	SseK2 + YM155	10,000 s (23 C)	1.92	0.05135	3.41
SseK2	190	200	LISSHNKDKGH	SseK2 + YM155	12 h (28 C)	1.96	0.015781	3.41
SseK2	190	200	LISSHNKDKGH	SseK2 alone	10 s (23 C)	1.48	0.053545	3.42
SseK2	190	200	LISSHNKDKGH	SseK2 alone	100 s (23 C)	1.70	0.08243	3.39
SseK2	190	200	LISSHNKDKGH	SseK2 alone	1,000 s (23 C)	2.07	0.052868	3.40
SseK2	190	200	LISSHNKDKGH	SseK2 alone	10,000 s (23 C)	1.92	0.071299	3.39
SseK2	190	200	LISSHNKDKGH	SseK2 alone	12 h (28 C)	2.00	0.016338	3.39
SseK2	191	200	ISSHNKDKGH	SseK2 + YM155	10 s (23 C)	1.77	0.0301	2.49
SseK2	191	200	ISSHNKDKGH	SseK2 + YM155	100 s (23 C)	1.82	0.032594	2.47
SseK2	191	200	ISSHNKDKGH	SseK2 + YM155	1,000 s (23 C)	1.86	0.014719	2.47
SseK2	191	200	ISSHNKDKGH	SseK2 + YM155	10,000 s (23 C)	1.81	0.047002	2.48
SseK2	191	200	ISSHNKDKGH	SseK2 + YM155	12 h (28 C)	1.78	0.006507	2.47
SseK2	191	200	ISSHNKDKGH	SseK2 alone	10 s (23 C)	1.75	0.032785	2.48
SseK2	191	200	ISSHNKDKGH	SseK2 alone	100 s (23 C)	1.81	0.065063	2.47
SseK2	191	200	ISSHNKDKGH	SseK2 alone	1,000 s (23 C)	1.90	0.041425	2.47
SseK2	191	200	ISSHNKDKGH	SseK2 alone	10,000 s (23 C)	1.88	0.055626	2.48
SseK2	191	200	ISSHNKDKGH	SseK2 alone	12 h (28 C)	1.83	0.038362	2.47
SseK2	191	201	ISSHNKDKGHL	SseK2 + YM155	10 s (23 C)	2.36	0.027571	2.49

SseK2	191	201	ISSHNKDKGHL	SseK2 + YM155	100 s (23 C)	2.47	0.036107	2.47
SseK2	191	201	ISSHNKDKGHL	SseK2 + YM155	1,000 s (23 C)	2.45	0.016163	2.47
SseK2	191	201	ISSHNKDKGHL	SseK2 + YM155	10,000 s (23 C)	2.39	0.059418	2.48
SseK2	191	201	ISSHNKDKGHL	SseK2 + YM155	12 h (28 C)	2.41	0.021598	2.47
SseK2	191	201	ISSHNKDKGHL	SseK2 alone	10 s (23 C)	2.36	0.052323	2.48
SseK2	191	201	ISSHNKDKGHL	SseK2 alone	100 s (23 C)	2.49	0.017238	2.47
SseK2	191	201	ISSHNKDKGHL	SseK2 alone	1,000 s (23 C)	2.55	0.050476	2.47
SseK2	191	201	ISSHNKDKGHL	SseK2 alone	10,000 s (23 C)	2.50	0.055869	2.48
SseK2	191	201	ISSHNKDKGHL	SseK2 alone	12 h (28 C)	2.42	0.02666	2.47
SseK2	191	205	ISSHNKDKGHLWFDF	SseK2 + YM155	10 s (23 C)	1.80	0.081933	7.28
SseK2	191	205	ISSHNKDKGHLWFDF	SseK2 + YM155	100 s (23 C)	2.66	0.021195	7.25
SseK2	191	205	ISSHNKDKGHLWFDF	SseK2 + YM155	1,000 s (23 C)	3.41	0.014046	7.26
SseK2	191	205	ISSHNKDKGHLWFDF	SseK2 + YM155	10,000 s (23 C)	3.66	0.012736	7.26
SseK2	191	205	ISSHNKDKGHLWFDF	SseK2 + YM155	12 h (28 C)	3.76	0.099804	7.26
SseK2	191	205	ISSHNKDKGHLWFDF	SseK2 alone	10 s (23 C)	1.80	0.052587	7.27
SseK2	191	205	ISSHNKDKGHLWFDF	SseK2 alone	100 s (23 C)	2.75	0.106006	7.26
SseK2	191	205	ISSHNKDKGHLWFDF	SseK2 alone	1,000 s (23 C)	3.48	0.081682	7.26
SseK2	191	205	ISSHNKDKGHLWFDF	SseK2 alone	10,000 s (23 C)	3.80	0.030959	7.26
SseK2	191	205	ISSHNKDKGHLWFDF	SseK2 alone	12 h (28 C)	3.91	0.070597	7.26
SseK2	192	205	SSHNKDKGHLWFDF	SseK2 + YM155	10 s (23 C)	1.57	0.067011	7.29
SseK2	192	205	SSHNKDKGHLWFDF	SseK2 + YM155	100 s (23 C)	2.13	0.010744	7.25
SseK2	192	205	SSHNKDKGHLWFDF	SseK2 + YM155	1,000 s (23 C)	2.88	0.059506	7.26
SseK2	192	205	SSHNKDKGHLWFDF	SseK2 + YM155	10,000 s (23 C)	3.14	0.029107	7.26
SseK2	192	205	SSHNKDKGHLWFDF	SseK2 + YM155	12 h (28 C)	3.14	0.08838	7.26
SseK2	192	205	SSHNKDKGHLWFDF	SseK2 alone	10 s (23 C)	1.53	0.015494	7.26
SseK2	192	205	SSHNKDKGHLWFDF	SseK2 alone	100 s (23 C)	2.25	0.052178	7.26
SseK2	192	205	SSHNKDKGHLWFDF	SseK2 alone	1,000 s (23 C)	2.90	0.046634	7.26
SseK2	192	205	SSHNKDKGHLWFDF	SseK2 alone	10,000 s (23 C)	3.20	0.113822	7.26

SseK2	192	205	SSHNKDKGHLWFDF	SseK2 alone	12 h (28 C)	3.23	0.01284	7.26
SseK2	194	201	HNKDKGHL	SseK2 + YM155	10 s (23 C)	1.32	0.005757	2.49
SseK2	194	201	HNKDKGHL	SseK2 + YM155	100 s (23 C)	1.32	0.076227	2.47
SseK2	194	201	HNKDKGHL	SseK2 + YM155	1,000 s (23 C)	1.36	0.014863	2.47
SseK2	194	201	HNKDKGHL	SseK2 + YM155	10,000 s (23 C)	1.30	0.011221	2.48
SseK2	194	201	HNKDKGHL	SseK2 + YM155	12 h (28 C)	1.11	0.038694	2.46
SseK2	194	201	HNKDKGHL	SseK2 alone	10 s (23 C)	1.24	0.023384	2.49
SseK2	194	201	HNKDKGHL	SseK2 alone	100 s (23 C)	1.27	0.038284	2.47
SseK2	194	201	HNKDKGHL	SseK2 alone	1,000 s (23 C)	1.30	0.024665	2.47
SseK2	194	201	HNKDKGHL	SseK2 alone	10,000 s (23 C)	1.40	0.023091	2.48
SseK2	194	201	HNKDKGHL	SseK2 alone	12 h (28 C)	1.36	0	2.46
SseK2	206	210	YRNLF	SseK2 + YM155	10 s (23 C)	0.17	0.062804	6.58
SseK2	206	210	YRNLF	SseK2 + YM155	100 s (23 C)	0.17	0.019146	6.58
SseK2	206	210	YRNLF	SseK2 + YM155	1,000 s (23 C)	0.17	0.061251	6.55
SseK2	206	210	YRNLF	SseK2 + YM155	10,000 s (23 C)	0.15	0.013447	6.54
SseK2	206	210	YRNLF	SseK2 + YM155	12 h (28 C)	0.74	0.00096	6.56
SseK2	206	210	YRNLF	SseK2 alone	10 s (23 C)	0.17	0.009607	6.73
SseK2	206	210	YRNLF	SseK2 alone	100 s (23 C)	0.21	0.042961	6.72
SseK2	206	210	YRNLF	SseK2 alone	1,000 s (23 C)	0.20	0.019686	6.72
SseK2	206	210	YRNLF	SseK2 alone	10,000 s (23 C)	0.22	0.012883	6.72
SseK2	206	210	YRNLF	SseK2 alone	12 h (28 C)	0.69	0.004102	6.71
SseK2	206	211	YRNLFL	SseK2 + YM155	10 s (23 C)	0.18	0.058845	7.53
SseK2	206	211	YRNLFL	SseK2 + YM155	100 s (23 C)	0.18	0.015466	7.49
SseK2	206	211	YRNLFL	SseK2 + YM155	1,000 s (23 C)	0.19	0.049496	7.51
SseK2	206	211	YRNLFL	SseK2 + YM155	10,000 s (23 C)	0.21	0.030325	7.51
SseK2	206	211	YRNLFL	SseK2 + YM155	12 h (28 C)	0.89	0.057999	7.50
SseK2	206	211	YRNLFL	SseK2 alone	10 s (23 C)	0.16	0.006057	7.52
SseK2	206	211	YRNLFL	SseK2 alone	100 s (23 C)	0.18	0.038657	7.51

SseK2	206	211	YRNLFL	SseK2 alone	1,000 s (23 C)	0.19	0.034953	7.51
SseK2	206	211	YRNLFL	SseK2 alone	10,000 s (23 C)	0.21	0.032642	7.51
SseK2	206	211	YRNLFL	SseK2 alone	12 h (28 C)	0.78	0.033867	7.50
SseK2	206	217	YRNLFLLKGSDA	SseK2 + YM155	10 s (23 C)	0.23	0.064471	6.51
SseK2	206	217	YRNLFLLKGSDA	SseK2 + YM155	100 s (23 C)	0.22	0.029812	6.49
SseK2	206	217	YRNLFLLKGSDA	SseK2 + YM155	1,000 s (23 C)	0.34	0.027637	6.48
SseK2	206	217	YRNLFLLKGSDA	SseK2 + YM155	10,000 s (23 C)	0.69	0.04246	6.48
SseK2	206	217	YRNLFLLKGSDA	SseK2 + YM155	12 h (28 C)	1.65	0.000009	6.48
SseK2	206	217	YRNLFLLKGSDA	SseK2 alone	10 s (23 C)	0.20	0.003569	6.58
SseK2	206	217	YRNLFLLKGSDA	SseK2 alone	100 s (23 C)	0.21	0.009389	6.57
SseK2	206	217	YRNLFLLKGSDA	SseK2 alone	1,000 s (23 C)	0.28	0.022755	6.57
SseK2	206	217	YRNLFLLKGSDA	SseK2 alone	10,000 s (23 C)	0.71	0.020051	6.57
SseK2	206	217	YRNLFLLKGSDA	SseK2 alone	12 h (28 C)	1.65	0.039443	6.56
SseK2	210	217	FLLKGSDA	SseK2 + YM155	10 s (23 C)	0.29	0.002404	5.34
SseK2	210	217	FLLKGSDA	SseK2 + YM155	100 s (23 C)	0.30	0.008775	5.31
SseK2	210	217	FLLKGSDA	SseK2 + YM155	1,000 s (23 C)	0.37	0.022569	5.31
SseK2	210	217	FLLKGSDA	SseK2 + YM155	10,000 s (23 C)	0.72	0.036961	5.31
SseK2	210	217	FLLKGSDA	SseK2 + YM155	12 h (28 C)	1.75	0.060802	5.31
SseK2	210	217	FLLKGSDA	SseK2 alone	10 s (23 C)	0.29	0.053021	5.35
SseK2	210	217	FLLKGSDA	SseK2 alone	100 s (23 C)	0.33	0.034426	5.33
SseK2	210	217	FLLKGSDA	SseK2 alone	1,000 s (23 C)	0.35	0.01234	5.34
SseK2	210	217	FLLKGSDA	SseK2 alone	10,000 s (23 C)	0.70	0.010899	5.34
SseK2	210	217	FLLKGSDA	SseK2 alone	12 h (28 C)	1.70	0.022754	5.33
SseK2	210	218	FLLKGSDAF	SseK2 + YM155	10 s (23 C)	0.05	0.067498	6.89
SseK2	210	218	FLLKGSDAF	SseK2 + YM155	100 s (23 C)	0.03	0.010088	6.86
SseK2	210	218	FLLKGSDAF	SseK2 + YM155	1,000 s (23 C)	0.11	0.092091	6.88
SseK2	210	218	FLLKGSDAF	SseK2 + YM155	10,000 s (23 C)	0.52	0.014269	6.86
SseK2	210	218	FLLKGSDAF	SseK2 + YM155	12 h (28 C)	1.76	0.017509	6.87

SseK2	210	218	FLLKGSDAF	SseK2 alone	10 s (23 C)	0.17	0.014106	6.92
SseK2	210	218	FLLKGSDAF	SseK2 alone	100 s (23 C)	0.15	0.028188	6.91
SseK2	210	218	FLLKGSDAF	SseK2 alone	1,000 s (23 C)	0.20	0.020673	6.91
SseK2	210	218	FLLKGSDAF	SseK2 alone	10,000 s (23 C)	0.57	0.019466	6.91
SseK2	210	218	FLLKGSDAF	SseK2 alone	12 h (28 C)	1.78	0.01142	6.91
SseK2	210	219	FLLKGSDAFL	SseK2 + YM155	10 s (23 C)	0.17	0.012036	7.72
SseK2	210	219	FLLKGSDAFL	SseK2 + YM155	100 s (23 C)	0.17	0.015092	7.69
SseK2	210	219	FLLKGSDAFL	SseK2 + YM155	1,000 s (23 C)	0.42	0.059681	7.70
SseK2	210	219	FLLKGSDAFL	SseK2 + YM155	10,000 s (23 C)	1.30	0.018947	7.70
SseK2	210	219	FLLKGSDAFL	SseK2 + YM155	12 h (28 C)	2.66	0.047205	7.70
SseK2	210	219	FLLKGSDAFL	SseK2 alone	10 s (23 C)	0.20	0.044598	7.71
SseK2	210	219	FLLKGSDAFL	SseK2 alone	100 s (23 C)	0.21	0.017184	7.70
SseK2	210	219	FLLKGSDAFL	SseK2 alone	1,000 s (23 C)	0.38	0.019879	7.71
SseK2	210	219	FLLKGSDAFL	SseK2 alone	10,000 s (23 C)	1.28	0.007586	7.71
SseK2	210	219	FLLKGSDAFL	SseK2 alone	12 h (28 C)	2.62	0.029322	7.70
SseK2	211	216	LLKGSD	SseK2 + YM155	10 s (23 C)	0.12	0.042699	2.51
SseK2	211	216	LLKGSD	SseK2 + YM155	100 s (23 C)	0.17	0.026368	2.49
SseK2	211	216	LLKGSD	SseK2 + YM155	1,000 s (23 C)	0.31	0.046249	2.49
SseK2	211	216	LLKGSD	SseK2 + YM155	10,000 s (23 C)	0.66	0.024385	2.50
SseK2	211	216	LLKGSD	SseK2 + YM155	12 h (28 C)	1.47	0.008251	2.49
SseK2	211	216	LLKGSD	SseK2 alone	10 s (23 C)	0.18	0.03778	2.50
SseK2	211	216	LLKGSD	SseK2 alone	100 s (23 C)	0.21	0.03073	2.49
SseK2	211	216	LLKGSD	SseK2 alone	1,000 s (23 C)	0.27	0.021478	2.49
SseK2	211	216	LLKGSD	SseK2 alone	10,000 s (23 C)	0.65	0.033059	2.50
SseK2	211	216	LLKGSD	SseK2 alone	12 h (28 C)	1.44	0.00748	2.49
SseK2	211	217	LLKGSDA	SseK2 + YM155	10 s (23 C)	-0.04	0.010313	3.19
SseK2	211	217	LLKGSDA	SseK2 + YM155	100 s (23 C)	-0.10	0.011723	3.11
SseK2	211	217	LLKGSDA	SseK2 + YM155	1,000 s (23 C)	0.01	0.046825	3.14

SseK2	211	217	LLKGSDA	SseK2 + YM155	10,000 s (23 C)	0.42	0.021496	3.15
SseK2	211	217	LLKGSDA	SseK2 + YM155	12 h (28 C)	1.29	0.002759	3.14
SseK2	211	217	LLKGSDA	SseK2 alone	10 s (23 C)	-0.08	0.004488	3.14
SseK2	211	217	LLKGSDA	SseK2 alone	100 s (23 C)	-0.10	0.002497	3.13
SseK2	211	217	LLKGSDA	SseK2 alone	1,000 s (23 C)	-0.03	0.002027	3.11
SseK2	211	217	LLKGSDA	SseK2 alone	10,000 s (23 C)	0.42	0.036404	3.11
SseK2	211	217	LLKGSDA	SseK2 alone	12 h (28 C)	1.27	0.052971	3.11
SseK2	211	218	LLKGSDAF	SseK2 + YM155	10 s (23 C)	0.24	0.042738	5.62
SseK2	211	218	LLKGSDAF	SseK2 + YM155	100 s (23 C)	0.18	0.01335	5.58
SseK2	211	218	LLKGSDAF	SseK2 + YM155	1,000 s (23 C)	0.31	0.000189	5.60
SseK2	211	218	LLKGSDAF	SseK2 + YM155	10,000 s (23 C)	0.64	0.024514	5.60
SseK2	211	218	LLKGSDAF	SseK2 + YM155	12 h (28 C)	1.78	0.043633	5.60
SseK2	211	218	LLKGSDAF	SseK2 alone	10 s (23 C)	0.22	0.013909	5.64
SseK2	211	218	LLKGSDAF	SseK2 alone	100 s (23 C)	0.24	0.021053	5.63
SseK2	211	218	LLKGSDAF	SseK2 alone	1,000 s (23 C)	0.27	0.014002	5.63
SseK2	211	218	LLKGSDAF	SseK2 alone	10,000 s (23 C)	0.68	0.004697	5.64
SseK2	211	218	LLKGSDAF	SseK2 alone	12 h (28 C)	1.73	0.0292	5.63
SseK2	211	224	LLKGSDAFLEAGKP	SseK2 + YM155	10 s (23 C)	0.70	0.026248	7.31
SseK2	211	224	LLKGSDAFLEAGKP	SseK2 + YM155	100 s (23 C)	0.85	0.039067	7.28
SseK2	211	224	LLKGSDAFLEAGKP	SseK2 + YM155	1,000 s (23 C)	1.22	0.022998	7.29
SseK2	211	224	LLKGSDAFLEAGKP	SseK2 + YM155	10,000 s (23 C)	1.40	0.016527	7.29
SseK2	211	224	LLKGSDAFLEAGKP	SseK2 + YM155	12 h (28 C)	1.87	0.083147	7.29
SseK2	211	224	LLKGSDAFLEAGKP	SseK2 alone	10 s (23 C)	0.70	0.027674	7.30
SseK2	211	224	LLKGSDAFLEAGKP	SseK2 alone	100 s (23 C)	0.93	0.026962	7.29
SseK2	211	224	LLKGSDAFLEAGKP	SseK2 alone	1,000 s (23 C)	1.24	0.031916	7.29
SseK2	211	224	LLKGSDAFLEAGKP	SseK2 alone	10,000 s (23 C)	1.41	0.036714	7.29
SseK2	211	224	LLKGSDAFLEAGKP	SseK2 alone	12 h (28 C)	1.92	0	7.29
SseK2	212	217	LKGSDA	SseK2 + YM155	10 s (23 C)	0.17	0.053292	2.50

SseK2	212	217	LKGSDA	SseK2 + YM155	100 s (23 C)	0.17	0.015285	2.46
SseK2	212	217	LKGSDA	SseK2 + YM155	1,000 s (23 C)	0.22	0.039913	2.48
SseK2	212	217	LKGSDA	SseK2 + YM155	10,000 s (23 C)	0.56	0.020925	2.48
SseK2	212	217	LKGSDA	SseK2 + YM155	12 h (28 C)	1.22	0.016874	2.46
SseK2	212	217	LKGSDA	SseK2 alone	10 s (23 C)	0.14	0.026529	2.48
SseK2	212	217	LKGSDA	SseK2 alone	100 s (23 C)	0.17	0.006676	2.46
SseK2	212	217	LKGSDA	SseK2 alone	1,000 s (23 C)	0.22	0.007319	2.47
SseK2	212	217	LKGSDA	SseK2 alone	10,000 s (23 C)	0.53	0.080217	2.48
SseK2	212	217	LKGSDA	SseK2 alone	12 h (28 C)	1.20	0.018106	2.46
SseK2	212	218	LKGSDAF	SseK2 + YM155	10 s (23 C)	0.19	0.082218	4.98
SseK2	212	218	LKGSDAF	SseK2 + YM155	100 s (23 C)	0.20	0.015976	4.95
SseK2	212	218	LKGSDAF	SseK2 + YM155	1,000 s (23 C)	0.27	0.055768	4.96
SseK2	212	218	LKGSDAF	SseK2 + YM155	10,000 s (23 C)	0.55	0.032541	4.96
SseK2	212	218	LKGSDAF	SseK2 + YM155	12 h (28 C)	1.40	0.029272	4.96
SseK2	212	218	LKGSDAF	SseK2 alone	10 s (23 C)	0.15	0.018911	4.98
SseK2	212	218	LKGSDAF	SseK2 alone	100 s (23 C)	0.23	0.02285	4.96
SseK2	212	218	LKGSDAF	SseK2 alone	1,000 s (23 C)	0.25	0.01189	4.97
SseK2	212	218	LKGSDAF	SseK2 alone	10,000 s (23 C)	0.57	0.027707	4.97
SseK2	212	218	LKGSDAF	SseK2 alone	12 h (28 C)	1.46	0.000927	4.96
SseK2	212	219	LKGSDAFL	SseK2 + YM155	10 s (23 C)	0.15	0.02294	6.29
SseK2	212	219	LKGSDAFL	SseK2 + YM155	100 s (23 C)	0.16	0.018904	6.28
SseK2	212	219	LKGSDAFL	SseK2 + YM155	1,000 s (23 C)	0.39	0.047294	6.26
SseK2	212	219	LKGSDAFL	SseK2 + YM155	10,000 s (23 C)	1.32	0.028132	6.26
SseK2	212	219	LKGSDAFL	SseK2 + YM155	12 h (28 C)	2.37	0.02174	6.26
SseK2	212	219	LKGSDAFL	SseK2 alone	10 s (23 C)	0.14	0.01927	6.38
SseK2	212	219	LKGSDAFL	SseK2 alone	100 s (23 C)	0.18	0.022816	6.37
SseK2	212	219	LKGSDAFL	SseK2 alone	1,000 s (23 C)	0.39	0.040274	6.38
SseK2	212	219	LKGSDAFL	SseK2 alone	10,000 s (23 C)	1.36	0.040037	6.38

SseK2	212	219	LKGSDAFL	SseK2 alone	12 h (28 C)	2.40	0.006426	6.37
SseK2	213	219	KGSDAFL	SseK2 + YM155	10 s (23 C)	0.16	0.038669	5.62
SseK2	213	219	KGSDAFL	SseK2 + YM155	100 s (23 C)	0.08	0.01214	5.59
SseK2	213	219	KGSDAFL	SseK2 + YM155	1,000 s (23 C)	0.11	0.001292	5.59
SseK2	213	219	KGSDAFL	SseK2 + YM155	10,000 s (23 C)	0.48	0.025117	5.60
SseK2	213	219	KGSDAFL	SseK2 + YM155	12 h (28 C)	1.24	0.039922	5.60
SseK2	213	219	KGSDAFL	SseK2 alone	10 s (23 C)	0.13	0.024862	5.64
SseK2	213	219	KGSDAFL	SseK2 alone	100 s (23 C)	0.08	0.007441	5.63
SseK2	213	219	KGSDAFL	SseK2 alone	1,000 s (23 C)	0.12	0.004124	5.63
SseK2	213	219	KGSDAFL	SseK2 alone	10,000 s (23 C)	0.47	0.017341	5.63
SseK2	213	219	KGSDAFL	SseK2 alone	12 h (28 C)	1.24	0.034597	5.62
SseK2	217	237	AFLEAGKPGCHHLQPGGGCIY	SseK2 + YM155	10 s (23 C)	2.22	0.021338	5.76
SseK2	217	237	AFLEAGKPGCHHLQPGGGCIY	SseK2 + YM155	100 s (23 C)	4.53	0.012104	5.73
SseK2	217	237	AFLEAGKPGCHHLQPGGGCIY	SseK2 + YM155	1,000 s (23 C)	5.79	0.066546	5.74
SseK2	217	237	AFLEAGKPGCHHLQPGGGCIY	SseK2 + YM155	10,000 s (23 C)	7.06	0.059659	5.74
SseK2	217	237	AFLEAGKPGCHHLQPGGGCIY	SseK2 + YM155	12 h (28 C)	7.70	0.014842	5.74
SseK2	217	237	AFLEAGKPGCHHLQPGGGCIY	SseK2 alone	10 s (23 C)	2.28	0.13514	5.79
SseK2	217	237	AFLEAGKPGCHHLQPGGGCIY	SseK2 alone	100 s (23 C)	4.60	0.099016	5.77
SseK2	217	237	AFLEAGKPGCHHLQPGGGCIY	SseK2 alone	1,000 s (23 C)	5.81	0.137875	5.78
SseK2	217	237	AFLEAGKPGCHHLQPGGGCIY	SseK2 alone	10,000 s (23 C)	7.11	0.035134	5.78
SseK2	217	237	AFLEAGKPGCHHLQPGGGCIY	SseK2 alone	12 h (28 C)	7.66	0.029761	5.78
SseK2	218	234	FLEAGKPGCHHLQPGGG	SseK2 + YM155	10 s (23 C)	2.07	0.00577	4.14
SseK2	218	234	FLEAGKPGCHHLQPGGG	SseK2 + YM155	100 s (23 C)	4.14	0.036323	4.12
SseK2	218	234	FLEAGKPGCHHLQPGGG	SseK2 + YM155	1,000 s (23 C)	5.13	0.028639	4.12
SseK2	218	234	FLEAGKPGCHHLQPGGG	SseK2 + YM155	10,000 s (23 C)	5.64	0.080306	4.12
SseK2	218	234	FLEAGKPGCHHLQPGGG	SseK2 + YM155	12 h (28 C)	5.64	0.063983	4.11
SseK2	218	234	FLEAGKPGCHHLQPGGG	SseK2 alone	10 s (23 C)	2.16	0.05854	4.13
SseK2	218	234	FLEAGKPGCHHLQPGGG	SseK2 alone	100 s (23 C)	4.26	0.040374	4.12

SseK2	218	234	FLEAGKPGCHHLQPGGG	SseK2 alone	1,000 s (23 C)	5.17	0.076582	4.12
SseK2	218	234	FLEAGKPGCHHLQPGGG	SseK2 alone	10,000 s (23 C)	5.74	0.045902	4.12
SseK2	218	234	FLEAGKPGCHHLQPGGG	SseK2 alone	12 h (28 C)	5.71	0.030544	4.12
SseK2	218	237	FLEAGKPGCHHLQPGGGCIY	SseK2 + YM155	10 s (23 C)	2.03	0.009156	5.48
SseK2	218	237	FLEAGKPGCHHLQPGGGCIY	SseK2 + YM155	100 s (23 C)	4.13	0.033465	5.45
SseK2	218	237	FLEAGKPGCHHLQPGGGCIY	SseK2 + YM155	1,000 s (23 C)	5.16	0.024954	5.45
SseK2	218	237	FLEAGKPGCHHLQPGGGCIY	SseK2 + YM155	10,000 s (23 C)	5.71	0.010983	5.45
SseK2	218	237	FLEAGKPGCHHLQPGGGCIY	SseK2 + YM155	12 h (28 C)	6.08	0.070561	5.46
SseK2	218	237	FLEAGKPGCHHLQPGGGCIY	SseK2 alone	10 s (23 C)	2.12	0.113727	5.50
SseK2	218	237	FLEAGKPGCHHLQPGGGCIY	SseK2 alone	100 s (23 C)	4.30	0.110063	5.49
SseK2	218	237	FLEAGKPGCHHLQPGGGCIY	SseK2 alone	1,000 s (23 C)	5.18	0.104635	5.49
SseK2	218	237	FLEAGKPGCHHLQPGGGCIY	SseK2 alone	10,000 s (23 C)	5.76	0.070399	5.49
SseK2	218	237	FLEAGKPGCHHLQPGGGCIY	SseK2 alone	12 h (28 C)	6.20	0.090936	5.48
SseK2	218	238	FLEAGKPGCHHLQPGGGCIYL	SseK2 + YM155	10 s (23 C)	2.00	0.026046	6.35
SseK2	218	238	FLEAGKPGCHHLQPGGGCIYL	SseK2 + YM155	100 s (23 C)	4.23	0.035611	6.33
SseK2	218	238	FLEAGKPGCHHLQPGGGCIYL	SseK2 + YM155	1,000 s (23 C)	5.31	0.069287	6.31
SseK2	218	238	FLEAGKPGCHHLQPGGGCIYL	SseK2 + YM155	10,000 s (23 C)	5.89	0.040388	6.32
SseK2	218	238	FLEAGKPGCHHLQPGGGCIYL	SseK2 + YM155	12 h (28 C)	6.41	0.030721	6.32
SseK2	218	238	FLEAGKPGCHHLQPGGGCIYL	SseK2 alone	10 s (23 C)	2.10	0.112205	6.41
SseK2	218	238	FLEAGKPGCHHLQPGGGCIYL	SseK2 alone	100 s (23 C)	4.35	0.112396	6.40
SseK2	218	238	FLEAGKPGCHHLQPGGGCIYL	SseK2 alone	1,000 s (23 C)	5.28	0.06622	6.40
SseK2	218	238	FLEAGKPGCHHLQPGGGCIYL	SseK2 alone	10,000 s (23 C)	5.93	0.074192	6.40
SseK2	218	238	FLEAGKPGCHHLQPGGGCIYL	SseK2 alone	12 h (28 C)	6.49	0.051634	6.40
SseK2	220	234	EAGKPGCHHLQPGGG	SseK2 + YM155	10 s (23 C)	2.37	ND	2.48
SseK2	220	234	EAGKPGCHHLQPGGG	SseK2 + YM155	100 s (23 C)	4.34	0.048793	2.46
SseK2	220	234	EAGKPGCHHLQPGGG	SseK2 + YM155	1,000 s (23 C)	4.76	0.035225	2.47
SseK2	220	234	EAGKPGCHHLQPGGG	SseK2 + YM155	10,000 s (23 C)	5.10	0.053635	2.48
SseK2	220	234	EAGKPGCHHLQPGGG	SseK2 + YM155	12 h (28 C)	5.23	0.035044	2.46

SseK2	220	234	EAGKPGCHHLQPGGG	SseK2 alone	10 s (23 C)	2.49	0.061157	2.48
SseK2	220	234	EAGKPGCHHLQPGGG	SseK2 alone	100 s (23 C)	4.55	0.054474	2.46
SseK2	220	234	EAGKPGCHHLQPGGG	SseK2 alone	1,000 s (23 C)	5.04	0.042672	2.46
SseK2	220	234	EAGKPGCHHLQPGGG	SseK2 alone	10,000 s (23 C)	5.32	0.019743	2.47
SseK2	220	234	EAGKPGCHHLQPGGG	SseK2 alone	12 h (28 C)	5.34	0.031849	2.47
SseK2	220	235	EAGKPGCHHLQPGGGC	SseK2 + YM155	10 s (23 C)	2.23	0.031978	2.51
SseK2	220	235	EAGKPGCHHLQPGGGC	SseK2 + YM155	100 s (23 C)	4.15	0.032008	2.48
SseK2	220	235	EAGKPGCHHLQPGGGC	SseK2 + YM155	1,000 s (23 C)	4.66	0.020317	2.49
SseK2	220	235	EAGKPGCHHLQPGGGC	SseK2 + YM155	10,000 s (23 C)	5.01	0.038695	2.50
SseK2	220	235	EAGKPGCHHLQPGGGC	SseK2 + YM155	12 h (28 C)	5.18	0.01126	2.49
SseK2	220	235	EAGKPGCHHLQPGGGC	SseK2 alone	10 s (23 C)	2.43	0.132652	2.50
SseK2	220	235	EAGKPGCHHLQPGGGC	SseK2 alone	100 s (23 C)	4.41	0.087751	2.49
SseK2	220	235	EAGKPGCHHLQPGGGC	SseK2 alone	1,000 s (23 C)	4.90	0.059727	2.49
SseK2	220	235	EAGKPGCHHLQPGGGC	SseK2 alone	10,000 s (23 C)	5.31	0.016326	2.50
SseK2	220	235	EAGKPGCHHLQPGGGC	SseK2 alone	12 h (28 C)	5.43	0.005465	2.48
SseK2	220	238	EAGKPGCHHLQPGGGCIYL	SseK2 + YM155	10 s (23 C)	1.95	0.030608	5.93
SseK2	220	238	EAGKPGCHHLQPGGGCIYL	SseK2 + YM155	100 s (23 C)	3.88	0.027179	5.91
SseK2	220	238	EAGKPGCHHLQPGGGCIYL	SseK2 + YM155	1,000 s (23 C)	4.28	0.102937	5.91
SseK2	220	238	EAGKPGCHHLQPGGGCIYL	SseK2 + YM155	10,000 s (23 C)	4.68	0.053483	5.91
SseK2	220	238	EAGKPGCHHLQPGGGCIYL	SseK2 + YM155	12 h (28 C)	5.29	0.025492	5.91
SseK2	220	238	EAGKPGCHHLQPGGGCIYL	SseK2 alone	10 s (23 C)	2.09	0.078313	5.99
SseK2	220	238	EAGKPGCHHLQPGGGCIYL	SseK2 alone	100 s (23 C)	3.97	0.100517	5.98
SseK2	220	238	EAGKPGCHHLQPGGGCIYL	SseK2 alone	1,000 s (23 C)	4.41	0.101325	5.98
SseK2	220	238	EAGKPGCHHLQPGGGCIYL	SseK2 alone	10,000 s (23 C)	4.81	0.090148	5.98
SseK2	220	238	EAGKPGCHHLQPGGGCIYL	SseK2 alone	12 h (28 C)	5.42	0.119683	5.97
SseK2	221	230	AGKPGCHHLQ	SseK2 + YM155	10 s (23 C)	0.36	0.091645	3.44
SseK2	221	230	AGKPGCHHLQ	SseK2 + YM155	100 s (23 C)	1.02	0.059633	3.40
SseK2	221	230	AGKPGCHHLQ	SseK2 + YM155	1,000 s (23 C)	3.50	0.048954	3.38

SseK2	221	230	AGKPGCHHLQ	SseK2 + YM155	10,000 s (23 C)	4.19	0.018961	3.40
SseK2	221	230	AGKPGCHHLQ	SseK2 + YM155	12 h (28 C)	4.12	0.009048	3.40
SseK2	221	230	AGKPGCHHLQ	SseK2 alone	10 s (23 C)	0.34	0.04841	3.41
SseK2	221	230	AGKPGCHHLQ	SseK2 alone	100 s (23 C)	1.14	0.086308	3.39
SseK2	221	230	AGKPGCHHLQ	SseK2 alone	1,000 s (23 C)	3.57	0.105504	3.37
SseK2	221	230	AGKPGCHHLQ	SseK2 alone	10,000 s (23 C)	4.16	0.002402	3.38
SseK2	221	230	AGKPGCHHLQ	SseK2 alone	12 h (28 C)	4.17	0.001987	3.38
SseK2	236	240	IYLDA	SseK2 + YM155	10 s (23 C)	0.14	0.025509	6.01
SseK2	236	240	IYLDA	SseK2 + YM155	100 s (23 C)	0.24	0.008133	5.98
SseK2	236	240	IYLDA	SseK2 + YM155	1,000 s (23 C)	0.51	0.006783	5.98
SseK2	236	240	IYLDA	SseK2 + YM155	10,000 s (23 C)	0.54	0.033184	5.98
SseK2	236	240	IYLDA	SseK2 + YM155	12 h (28 C)	0.73	0.023326	5.99
SseK2	236	240	IYLDA	SseK2 alone	10 s (23 C)	0.16	0.023526	6.05
SseK2	236	240	IYLDA	SseK2 alone	100 s (23 C)	0.26	0.038345	6.03
SseK2	236	240	IYLDA	SseK2 alone	1,000 s (23 C)	0.51	0.010321	6.04
SseK2	236	240	IYLDA	SseK2 alone	10,000 s (23 C)	0.55	0.016463	6.04
SseK2	236	240	IYLDA	SseK2 alone	12 h (28 C)	0.74	0.030008	6.03
SseK2	236	242	IYLDADM	SseK2 + YM155	10 s (23 C)	0.17	0.050926	6.98
SseK2	236	242	IYLDADM	SseK2 + YM155	100 s (23 C)	0.34	0.033729	6.96
SseK2	236	242	IYLDADM	SseK2 + YM155	1,000 s (23 C)	1.16	0.043707	6.96
SseK2	236	242	IYLDADM	SseK2 + YM155	10,000 s (23 C)	1.40	0.03408	6.96
SseK2	236	242	IYLDADM	SseK2 + YM155	12 h (28 C)	1.88	0.051081	6.96
SseK2	236	242	IYLDADM	SseK2 alone	10 s (23 C)	0.13	0.017523	6.99
SseK2	236	242	IYLDADM	SseK2 alone	100 s (23 C)	0.35	0.063788	6.98
SseK2	236	242	IYLDADM	SseK2 alone	1,000 s (23 C)	1.17	0.068795	6.98
SseK2	236	242	IYLDADM	SseK2 alone	10,000 s (23 C)	1.39	0.047586	6.98
SseK2	236	242	IYLDADM	SseK2 alone	12 h (28 C)	1.91	0.022344	6.98
SseK2	238	243	LDADML	SseK2 + YM155	10 s (23 C)	0.07	0.03568	6.70

SseK2	238	243	LDADML	SseK2 + YM155	100 s (23 C)	0.27	0.021901	6.68
SseK2	238	243	LDADML	SseK2 + YM155	1,000 s (23 C)	1.14	0.01811	6.68
SseK2	238	243	LDADML	SseK2 + YM155	10,000 s (23 C)	1.78	0.010001	6.68
SseK2	238	243	LDADML	SseK2 + YM155	12 h (28 C)	2.28	0.009587	6.68
SseK2	238	243	LDADML	SseK2 alone	10 s (23 C)	0.12	0.015232	6.79
SseK2	238	243	LDADML	SseK2 alone	100 s (23 C)	0.34	0.063068	6.78
SseK2	238	243	LDADML	SseK2 alone	1,000 s (23 C)	1.20	0.037802	6.78
SseK2	238	243	LDADML	SseK2 alone	10,000 s (23 C)	1.86	0.015803	6.78
SseK2	238	243	LDADML	SseK2 alone	12 h (28 C)	2.32	0.009064	6.77
SseK2	239	244	DADMLL	SseK2 + YM155	10 s (23 C)	0.18	0.038588	7.41
SseK2	239	244	DADMLL	SseK2 + YM155	100 s (23 C)	0.23	0.025113	7.38
SseK2	239	244	DADMLL	SseK2 + YM155	1,000 s (23 C)	0.53	0.037802	7.39
SseK2	239	244	DADMLL	SseK2 + YM155	10,000 s (23 C)	1.26	0.033018	7.39
SseK2	239	244	DADMLL	SseK2 + YM155	12 h (28 C)	2.10	0.015374	7.39
SseK2	239	244	DADMLL	SseK2 alone	10 s (23 C)	0.16	0.016182	7.41
SseK2	239	244	DADMLL	SseK2 alone	100 s (23 C)	0.25	0.026168	7.39
SseK2	239	244	DADMLL	SseK2 alone	1,000 s (23 C)	0.58	0.057994	7.40
SseK2	239	244	DADMLL	SseK2 alone	10,000 s (23 C)	1.28	0.044447	7.40
SseK2	239	244	DADMLL	SseK2 alone	12 h (28 C)	2.05	0.006479	7.39
SseK2	241	256	DMLLTDKLGTLYLPDG	SseK2 + YM155	10 s (23 C)	1.50	0.045633	7.97
SseK2	241	256	DMLLTDKLGTLYLPDG	SseK2 + YM155	100 s (23 C)	3.41	0.025677	7.96
SseK2	241	256	DMLLTDKLGTLYLPDG	SseK2 + YM155	1,000 s (23 C)	5.01	0.043283	7.96
SseK2	241	256	DMLLTDKLGTLYLPDG	SseK2 + YM155	10,000 s (23 C)	6.91	0.049769	7.95
SseK2	241	256	DMLLTDKLGTLYLPDG	SseK2 + YM155	12 h (28 C)	8.47	0.042389	7.95
SseK2	241	256	DMLLTDKLGTLYLPDG	SseK2 alone	10 s (23 C)	1.57	0.07808	7.96
SseK2	241	256	DMLLTDKLGTLYLPDG	SseK2 alone	100 s (23 C)	3.48	0.031808	7.96
SseK2	241	256	DMLLTDKLGTLYLPDG	SseK2 alone	1,000 s (23 C)	5.02	0.051516	7.96
SseK2	241	256	DMLLTDKLGTLYLPDG	SseK2 alone	10,000 s (23 C)	6.90	0.101851	7.95

SseK2	241	256	DMLLTDKLGTLYLPDG	SseK2 alone	12 h (28 C)	8.47	0.040771	7.95
SseK2	242	256	MLLTDKLGTLYLPDG	SseK2 + YM155	10 s (23 C)	1.52	0.135287	7.80
SseK2	242	256	MLLTDKLGTLYLPDG	SseK2 + YM155	100 s (23 C)	3.45	0.064706	7.77
SseK2	242	256	MLLTDKLGTLYLPDG	SseK2 + YM155	1,000 s (23 C)	5.05	0.020093	7.78
SseK2	242	256	MLLTDKLGTLYLPDG	SseK2 + YM155	10,000 s (23 C)	6.49	0.076358	7.78
SseK2	242	256	MLLTDKLGTLYLPDG	SseK2 + YM155	12 h (28 C)	8.05	0.079288	7.78
SseK2	242	256	MLLTDKLGTLYLPDG	SseK2 alone	10 s (23 C)	1.57	0.102976	7.79
SseK2	242	256	MLLTDKLGTLYLPDG	SseK2 alone	100 s (23 C)	3.45	0.02503	7.78
SseK2	242	256	MLLTDKLGTLYLPDG	SseK2 alone	1,000 s (23 C)	5.03	0.054163	7.78
SseK2	242	256	MLLTDKLGTLYLPDG	SseK2 alone	10,000 s (23 C)	6.52	0.070854	7.78
SseK2	242	256	MLLTDKLGTLYLPDG	SseK2 alone	12 h (28 C)	7.97	0.007029	7.78
SseK2	243	248	LLTDKL	SseK2 + YM155	10 s (23 C)	0.16	0.033424	5.67
SseK2	243	248	LLTDKL	SseK2 + YM155	100 s (23 C)	0.56	ND	5.64
SseK2	243	248	LLTDKL	SseK2 + YM155	1,000 s (23 C)	1.70	0.041347	5.65
SseK2	243	248	LLTDKL	SseK2 + YM155	10,000 s (23 C)	2.61	0.025976	5.64
SseK2	243	248	LLTDKL	SseK2 + YM155	12 h (28 C)	2.78	0.021063	5.65
SseK2	243	248	LLTDKL	SseK2 alone	10 s (23 C)	0.16	0.013087	5.70
SseK2	243	248	LLTDKL	SseK2 alone	100 s (23 C)	0.61	0.031656	5.68
SseK2	243	248	LLTDKL	SseK2 alone	1,000 s (23 C)	1.73	0.04404	5.68
SseK2	243	248	LLTDKL	SseK2 alone	10,000 s (23 C)	2.66	0.022104	5.69
SseK2	243	248	LLTDKL	SseK2 alone	12 h (28 C)	2.82	0.035334	5.68
SseK2	243	250	LLTDKLGT	SseK2 + YM155	10 s (23 C)	0.60	0.008759	5.15
SseK2	243	250	LLTDKLGT	SseK2 + YM155	100 s (23 C)	1.40	0.006947	5.12
SseK2	243	250	LLTDKLGT	SseK2 + YM155	1,000 s (23 C)	2.34	0.016365	5.13
SseK2	243	250	LLTDKLGT	SseK2 + YM155	10,000 s (23 C)	2.99	0.037348	5.12
SseK2	243	250	LLTDKLGT	SseK2 + YM155	12 h (28 C)	3.21	0.023333	5.12
SseK2	243	250	LLTDKLGT	SseK2 alone	10 s (23 C)	0.62	0.048383	5.16
SseK2	243	250	LLTDKLGT	SseK2 alone	100 s (23 C)	1.47	0.027997	5.14

SseK2	243	250	LLTDKLGT	SseK2 alone	1,000 s (23 C)	2.37	0.044491	5.14
SseK2	243	250	LLTDKLGT	SseK2 alone	10,000 s (23 C)	3.07	0.028416	5.14
SseK2	243	250	LLTDKLGT	SseK2 alone	12 h (28 C)	3.21	0.023815	5.14
SseK2	243	251	LLTDKLGTL	SseK2 + YM155	10 s (23 C)	1.22	0.117492	6.49
SseK2	243	251	LLTDKLGTL	SseK2 + YM155	100 s (23 C)	2.36	0.019842	6.48
SseK2	243	251	LLTDKLGTL	SseK2 + YM155	1,000 s (23 C)	3.70	0.019801	6.47
SseK2	243	251	LLTDKLGTL	SseK2 + YM155	10,000 s (23 C)	4.51	0.010394	6.46
SseK2	243	251	LLTDKLGTL	SseK2 + YM155	12 h (28 C)	4.73	0.015362	6.47
SseK2	243	251	LLTDKLGTL	SseK2 alone	10 s (23 C)	1.16	0.02802	6.57
SseK2	243	251	LLTDKLGTL	SseK2 alone	100 s (23 C)	2.41	0.035001	6.56
SseK2	243	251	LLTDKLGTL	SseK2 alone	1,000 s (23 C)	3.73	0.045131	6.56
SseK2	243	251	LLTDKLGTL	SseK2 alone	10,000 s (23 C)	4.56	0.045564	6.56
SseK2	243	251	LLTDKLGTL	SseK2 alone	12 h (28 C)	4.77	0.037337	6.55
SseK2	243	256	LLTDKLGTLYLPDG	SseK2 + YM155	10 s (23 C)	1.42	0.024912	7.29
SseK2	243	256	LLTDKLGTLYLPDG	SseK2 + YM155	100 s (23 C)	3.27	0.012685	7.27
SseK2	243	256	LLTDKLGTLYLPDG	SseK2 + YM155	1,000 s (23 C)	4.72	0.060384	7.27
SseK2	243	256	LLTDKLGTLYLPDG	SseK2 + YM155	10,000 s (23 C)	5.88	0.02421	7.27
SseK2	243	256	LLTDKLGTLYLPDG	SseK2 + YM155	12 h (28 C)	6.97	0.035343	7.27
SseK2	243	256	LLTDKLGTLYLPDG	SseK2 alone	10 s (23 C)	1.48	0.080618	7.28
SseK2	243	256	LLTDKLGTLYLPDG	SseK2 alone	100 s (23 C)	3.33	0.066394	7.27
SseK2	243	256	LLTDKLGTLYLPDG	SseK2 alone	1,000 s (23 C)	4.71	0.035414	7.27
SseK2	243	256	LLTDKLGTLYLPDG	SseK2 alone	10,000 s (23 C)	5.93	0.021159	7.27
SseK2	243	256	LLTDKLGTLYLPDG	SseK2 alone	12 h (28 C)	6.96	0.038875	7.27
SseK2	243	258	LLTDKLGTLYLPDGIA	SseK2 + YM155	10 s (23 C)	1.61	0.009345	7.79
SseK2	243	258	LLTDKLGTLYLPDGIA	SseK2 + YM155	100 s (23 C)	3.47	0.017369	7.77
SseK2	243	258	LLTDKLGTLYLPDGIA	SseK2 + YM155	1,000 s (23 C)	4.95	0.0462	7.78
SseK2	243	258	LLTDKLGTLYLPDGIA	SseK2 + YM155	10,000 s (23 C)	6.16	0.01493	7.77
SseK2	243	258	LLTDKLGTLYLPDGIA	SseK2 + YM155	12 h (28 C)	7.60	0.022299	7.77

SseK2	243	258	LLTDKLGTLYLPDGIA	SseK2 alone	10 s (23 C)	1.66	0.08097	7.78
SseK2	243	258	LLTDKLGTLYLPDGIA	SseK2 alone	100 s (23 C)	3.55	0.050667	7.77
SseK2	243	258	LLTDKLGTLYLPDGIA	SseK2 alone	1,000 s (23 C)	4.95	0.06451	7.78
SseK2	243	258	LLTDKLGTLYLPDGIA	SseK2 alone	10,000 s (23 C)	6.19	0.032852	7.78
SseK2	243	258	LLTDKLGTLYLPDGIA	SseK2 alone	12 h (28 C)	7.57	0.018524	7.77
SseK2	244	250	LTDKLGT	SseK2 + YM155	10 s (23 C)	0.97	0.101972	4.18
SseK2	244	250	LTDKLGT	SseK2 + YM155	100 s (23 C)	1.33	0.020824	4.16
SseK2	244	250	LTDKLGT	SseK2 + YM155	1,000 s (23 C)	1.94	0.016086	4.16
SseK2	244	250	LTDKLGT	SseK2 + YM155	10,000 s (23 C)	2.69	0.035958	4.17
SseK2	244	250	LTDKLGT	SseK2 + YM155	12 h (28 C)	2.83	0.030825	4.16
SseK2	244	250	LTDKLGT	SseK2 alone	10 s (23 C)	0.88	0.029378	4.18
SseK2	244	250	LTDKLGT	SseK2 alone	100 s (23 C)	1.38	0.007908	4.16
SseK2	244	250	LTDKLGT	SseK2 alone	1,000 s (23 C)	1.98	0.057083	4.16
SseK2	244	250	LTDKLGT	SseK2 alone	10,000 s (23 C)	2.77	0.034037	4.16
SseK2	244	250	LTDKLGT	SseK2 alone	12 h (28 C)	2.91	0.020131	4.16
SseK2	244	251	LTDKLGTL	SseK2 + YM155	10 s (23 C)	1.07	0.104414	5.84
SseK2	244	251	LTDKLGTL	SseK2 + YM155	100 s (23 C)	1.86	0.025755	5.82
SseK2	244	251	LTDKLGTL	SseK2 + YM155	1,000 s (23 C)	2.78	0.01972	5.82
SseK2	244	251	LTDKLGTL	SseK2 + YM155	10,000 s (23 C)	3.47	0.026759	5.82
SseK2	244	251	LTDKLGTL	SseK2 + YM155	12 h (28 C)	3.72	0.037457	5.82
SseK2	244	251	LTDKLGTL	SseK2 alone	10 s (23 C)	1.06	0.03155	5.89
SseK2	244	251	LTDKLGTL	SseK2 alone	100 s (23 C)	1.95	0.022725	5.87
SseK2	244	251	LTDKLGTL	SseK2 alone	1,000 s (23 C)	2.87	0.059505	5.88
SseK2	244	251	LTDKLGTL	SseK2 alone	10,000 s (23 C)	3.61	0.053566	5.88
SseK2	244	251	LTDKLGTL	SseK2 alone	12 h (28 C)	3.81	0.004795	5.87
SseK2	244	256	LTDKLGTLYLPDG	SseK2 + YM155	10 s (23 C)	1.18	0.030206	6.85
SseK2	244	256	LTDKLGTLYLPDG	SseK2 + YM155	100 s (23 C)	2.62	0.005036	6.83
SseK2	244	256	LTDKLGTLYLPDG	SseK2 + YM155	1,000 s (23 C)	3.61	0.031076	6.83

SseK2	244	256	LTDKLGTLYLPDG	SseK2 + YM155	10,000 s (23 C)	4.63	0.049341	6.83
SseK2	244	256	LTDKLGTLYLPDG	SseK2 + YM155	12 h (28 C)	5.70	0.014119	6.83
SseK2	244	256	LTDKLGTLYLPDG	SseK2 alone	10 s (23 C)	1.27	0.087776	6.88
SseK2	244	256	LTDKLGTLYLPDG	SseK2 alone	100 s (23 C)	2.71	0.045527	6.87
SseK2	244	256	LTDKLGTLYLPDG	SseK2 alone	1,000 s (23 C)	3.64	0.034147	6.87
SseK2	244	256	LTDKLGTLYLPDG	SseK2 alone	10,000 s (23 C)	4.75	0.023565	6.87
SseK2	244	256	LTDKLGTLYLPDG	SseK2 alone	12 h (28 C)	5.76	0.020012	6.86
SseK2	244	258	LTDKLGTLYLPDGIA	SseK2 + YM155	10 s (23 C)	1.44	0.009788	7.46
SseK2	244	258	LTDKLGTLYLPDGIA	SseK2 + YM155	100 s (23 C)	2.90	0.017225	7.44
SseK2	244	258	LTDKLGTLYLPDGIA	SseK2 + YM155	1,000 s (23 C)	3.92	0.070842	7.44
SseK2	244	258	LTDKLGTLYLPDGIA	SseK2 + YM155	10,000 s (23 C)	5.03	0.013367	7.44
SseK2	244	258	LTDKLGTLYLPDGIA	SseK2 + YM155	12 h (28 C)	6.48	0.025588	7.44
SseK2	244	258	LTDKLGTLYLPDGIA	SseK2 alone	10 s (23 C)	1.52	0.049357	7.45
SseK2	244	258	LTDKLGTLYLPDGIA	SseK2 alone	100 s (23 C)	2.95	0.056237	7.44
SseK2	244	258	LTDKLGTLYLPDGIA	SseK2 alone	1,000 s (23 C)	3.94	0.087371	7.45
SseK2	244	258	LTDKLGTLYLPDGIA	SseK2 alone	10,000 s (23 C)	5.07	0.042018	7.44
SseK2	244	258	LTDKLGTLYLPDGIA	SseK2 alone	12 h (28 C)	6.42	0.041796	7.44
SseK2	244	259	LTDKLGTLYLPDGIAI	SseK2 + YM155	10 s (23 C)	1.54	0.002052	8.40
SseK2	244	259	LTDKLGTLYLPDGIAI	SseK2 + YM155	100 s (23 C)	3.11	0.029421	8.39
SseK2	244	259	LTDKLGTLYLPDGIAI	SseK2 + YM155	1,000 s (23 C)	4.21	0.022328	8.39
SseK2	244	259	LTDKLGTLYLPDGIAI	SseK2 + YM155	10,000 s (23 C)	5.45	0.045439	8.39
SseK2	244	259	LTDKLGTLYLPDGIAI	SseK2 + YM155	12 h (28 C)	7.18	0.024603	8.39
SseK2	244	259	LTDKLGTLYLPDGIAI	SseK2 alone	10 s (23 C)	1.65	0.159039	8.39
SseK2	244	259	LTDKLGTLYLPDGIAI	SseK2 alone	100 s (23 C)	3.21	0.113517	8.39
SseK2	244	259	LTDKLGTLYLPDGIAI	SseK2 alone	1,000 s (23 C)	4.21	0.057267	8.39
SseK2	244	259	LTDKLGTLYLPDGIAI	SseK2 alone	10,000 s (23 C)	5.42	0.051723	8.39
SseK2	244	259	LTDKLGTLYLPDGIAI	SseK2 alone	12 h (28 C)	7.04	0.042795	8.39
SseK2	245	250	TDKLGT	SseK2 + YM155	10 s (23 C)	0.61	0.065118	5.15

SseK2	245	250	TDKLGT	SseK2 + YM155	100 s (23 C)	0.96	0.030966	5.12
SseK2	245	250	TDKLGT	SseK2 + YM155	1,000 s (23 C)	1.55	0.043764	5.13
SseK2	245	250	TDKLGT	SseK2 + YM155	10,000 s (23 C)	2.03	0.035948	5.13
SseK2	245	250	TDKLGT	SseK2 + YM155	12 h (28 C)	2.01	0.039061	5.12
SseK2	245	250	TDKLGT	SseK2 alone	10 s (23 C)	0.59	0.00664	5.16
SseK2	245	250	TDKLGT	SseK2 alone	100 s (23 C)	0.99	0.0079	5.14
SseK2	245	250	TDKLGT	SseK2 alone	1,000 s (23 C)	1.53	0.082017	5.14
SseK2	245	250	TDKLGT	SseK2 alone	10,000 s (23 C)	2.04	0.129267	5.14
SseK2	245	250	TDKLGT	SseK2 alone	12 h (28 C)	2.11	0.042554	5.14
SseK2	245	256	TDKLGTLYLPDG	SseK2 + YM155	10 s (23 C)	1.29	0.060214	6.55
SseK2	245	256	TDKLGTLYLPDG	SseK2 + YM155	100 s (23 C)	2.74	0.015334	6.53
SseK2	245	256	TDKLGTLYLPDG	SseK2 + YM155	1,000 s (23 C)	3.53	0.049454	6.51
SseK2	245	256	TDKLGTLYLPDG	SseK2 + YM155	10,000 s (23 C)	4.44	0.028722	6.50
SseK2	245	256	TDKLGTLYLPDG	SseK2 + YM155	12 h (28 C)	5.50	0.034313	6.52
SseK2	245	256	TDKLGTLYLPDG	SseK2 alone	10 s (23 C)	1.33	0.05113	6.61
SseK2	245	256	TDKLGTLYLPDG	SseK2 alone	100 s (23 C)	2.80	0.03771	6.60
SseK2	245	256	TDKLGTLYLPDG	SseK2 alone	1,000 s (23 C)	3.54	0.072921	6.60
SseK2	245	256	TDKLGTLYLPDG	SseK2 alone	10,000 s (23 C)	4.43	0.046391	6.60
SseK2	245	256	TDKLGTLYLPDG	SseK2 alone	12 h (28 C)	5.44	0.056832	6.60
SseK2	246	251	DKLGTL	SseK2 + YM155	10 s (23 C)	0.81	0.038739	5.84
SseK2	246	251	DKLGTL	SseK2 + YM155	100 s (23 C)	1.70	0.028396	5.81
SseK2	246	251	DKLGTL	SseK2 + YM155	1,000 s (23 C)	2.34	0.003717	5.82
SseK2	246	251	DKLGTL	SseK2 + YM155	10,000 s (23 C)	2.91	0.035565	5.82
SseK2	246	251	DKLGTL	SseK2 + YM155	12 h (28 C)	3.23	0.062018	5.82
SseK2	246	251	DKLGTL	SseK2 alone	10 s (23 C)	0.79	0.027455	5.89
SseK2	246	251	DKLGTL	SseK2 alone	100 s (23 C)	1.74	0.037905	5.87
SseK2	246	251	DKLGTL	SseK2 alone	1,000 s (23 C)	2.38	0.057362	5.88
SseK2	246	251	DKLGTL	SseK2 alone	10,000 s (23 C)	2.90	0.055954	5.88

SseK2	246	251	DKLGTL	SseK2 alone	12 h (28 C)	3.31	0.077573	5.87
SseK2	251	256	LYLPDG	SseK2 + YM155	10 s (23 C)	0.16	0.025994	6.12
SseK2	251	256	LYLPDG	SseK2 + YM155	100 s (23 C)	0.22	0.012226	6.09
SseK2	251	256	LYLPDG	SseK2 + YM155	1,000 s (23 C)	0.26	0.014365	6.09
SseK2	251	256	LYLPDG	SseK2 + YM155	10,000 s (23 C)	0.54	0.041822	6.09
SseK2	251	256	LYLPDG	SseK2 + YM155	12 h (28 C)	1.37	0.000931	6.09
SseK2	251	256	LYLPDG	SseK2 alone	10 s (23 C)	0.20	0.011808	6.17
SseK2	251	256	LYLPDG	SseK2 alone	100 s (23 C)	0.32	0.018312	6.16
SseK2	251	256	LYLPDG	SseK2 alone	1,000 s (23 C)	0.33	0.015461	6.16
SseK2	251	256	LYLPDG	SseK2 alone	10,000 s (23 C)	0.61	0.01139	6.16
SseK2	251	256	LYLPDG	SseK2 alone	12 h (28 C)	1.35	0.032391	6.15
SseK2	251	258	LYLPDGIA	SseK2 + YM155	10 s (23 C)	0.29	0.056023	7.23
SseK2	251	258	LYLPDGIA	SseK2 + YM155	100 s (23 C)	0.31	0.014368	7.21
SseK2	251	258	LYLPDGIA	SseK2 + YM155	1,000 s (23 C)	0.39	0.018327	7.22
SseK2	251	258	LYLPDGIA	SseK2 + YM155	10,000 s (23 C)	0.76	0.025566	7.21
SseK2	251	258	LYLPDGIA	SseK2 + YM155	12 h (28 C)	1.96	0.019208	7.21
SseK2	251	258	LYLPDGIA	SseK2 alone	10 s (23 C)	0.28	0.019006	7.22
SseK2	251	258	LYLPDGIA	SseK2 alone	100 s (23 C)	0.33	0.008926	7.21
SseK2	251	258	LYLPDGIA	SseK2 alone	1,000 s (23 C)	0.36	0.011943	7.22
SseK2	251	258	LYLPDGIA	SseK2 alone	10,000 s (23 C)	0.75	0.025095	7.22
SseK2	251	258	LYLPDGIA	SseK2 alone	12 h (28 C)	1.95	0.006329	7.21
SseK2	257	270	IAIHVSRKDNHVSL	SseK2 + YM155	10 s (23 C)	3.42	0.126662	4.62
SseK2	257	270	IAIHVSRKDNHVSL	SseK2 + YM155	100 s (23 C)	4.29	0.046665	4.59
SseK2	257	270	IAIHVSRKDNHVSL	SseK2 + YM155	1,000 s (23 C)	4.62	0.023134	4.59
SseK2	257	270	IAIHVSRKDNHVSL	SseK2 + YM155	10,000 s (23 C)	4.80	0.075601	4.60
SseK2	257	270	IAIHVSRKDNHVSL	SseK2 + YM155	12 h (28 C)	5.23	0.093194	4.59
SseK2	257	270	IAIHVSRKDNHVSL	SseK2 alone	10 s (23 C)	3.45	0.100407	4.61
SseK2	257	270	IAIHVSRKDNHVSL	SseK2 alone	100 s (23 C)	4.35	0.03033	4.60

SseK2	257	270	IAIHVSRKDNHVSL	SseK2 alone	1,000 s (23 C)	4.71	0.102296	4.60
SseK2	257	270	IAIHVSRKDNHVSL	SseK2 alone	10,000 s (23 C)	4.90	0.088493	4.61
SseK2	257	270	IAIHVSRKDNHVSL	SseK2 alone	12 h (28 C)	5.26	0.002755	4.60
SseK2	257	273	IAIHVSRKDNHVSLENG	SseK2 + YM155	10 s (23 C)	4.81	0.05463	4.62
SseK2	257	273	IAIHVSRKDNHVSLENG	SseK2 + YM155	100 s (23 C)	6.55	0.096314	4.60
SseK2	257	273	IAIHVSRKDNHVSLENG	SseK2 + YM155	1,000 s (23 C)	7.09	0.045162	4.61
SseK2	257	273	IAIHVSRKDNHVSLENG	SseK2 + YM155	10,000 s (23 C)	7.23	0.090469	4.61
SseK2	257	273	IAIHVSRKDNHVSLENG	SseK2 + YM155	12 h (28 C)	7.96	0.023949	4.61
SseK2	257	273	IAIHVSRKDNHVSLENG	SseK2 alone	10 s (23 C)	4.97	0.121783	4.62
SseK2	257	273	IAIHVSRKDNHVSLENG	SseK2 alone	100 s (23 C)	6.61	0.119757	4.61
SseK2	257	273	IAIHVSRKDNHVSLENG	SseK2 alone	1,000 s (23 C)	7.18	0.050189	4.62
SseK2	257	273	IAIHVSRKDNHVSLENG	SseK2 alone	10,000 s (23 C)	7.43	0.021962	4.62
SseK2	257	273	IAIHVSRKDNHVSLENG	SseK2 alone	12 h (28 C)	7.93	0.049837	4.62
SseK2	257	275	IAIHVSRKDNHVSLENGII	SseK2 + YM155	10 s (23 C)	4.22	0.020955	5.65
SseK2	257	275	IAIHVSRKDNHVSLENGII	SseK2 + YM155	1,000 s (23 C)	6.67	0.030791	5.64
SseK2	257	275	IAIHVSRKDNHVSLENGII	SseK2 + YM155	10,000 s (23 C)	7.24	0.077425	5.63
SseK2	257	275	IAIHVSRKDNHVSLENGII	SseK2 + YM155	12 h (28 C)	8.13	0.050065	5.64
SseK2	257	275	IAIHVSRKDNHVSLENGII	SseK2 alone	10 s (23 C)	4.22	0.009087	5.69
SseK2	257	275	IAIHVSRKDNHVSLENGII	SseK2 alone	100 s (23 C)	5.99	0.107461	5.67
SseK2	257	275	IAIHVSRKDNHVSLENGII	SseK2 alone	1,000 s (23 C)	6.72	0.139966	5.67
SseK2	257	275	IAIHVSRKDNHVSLENGII	SseK2 alone	10,000 s (23 C)	7.36	0.086805	5.68
SseK2	257	275	IAIHVSRKDNHVSLENGII	SseK2 alone	12 h (28 C)	8.06	0.091246	5.67
SseK2	257	276	IAIHVSRKDNHVSLENGIIA	SseK2 + YM155	10 s (23 C)	4.50	0.002987	5.40
SseK2	257	276	IAIHVSRKDNHVSLENGIIA	SseK2 + YM155	100 s (23 C)	6.15	0.038758	5.38
SseK2	257	276	IAIHVSRKDNHVSLENGIIA	SseK2 + YM155	1,000 s (23 C)	6.93	0.031468	5.38
SseK2	257	276	IAIHVSRKDNHVSLENGIIA	SseK2 + YM155	10,000 s (23 C)	7.57	0.03235	5.38
SseK2	257	276	IAIHVSRKDNHVSLENGIIA	SseK2 + YM155	12 h (28 C)	8.59	0.09274	5.38
SseK2	257	276	IAIHVSRKDNHVSLENGIIA	SseK2 alone	10 s (23 C)	4.48	0.010493	5.42

SseK2	257	276	IAIHVSRKDNHVSLENGIIA	SseK2 alone	100 s (23 C)	6.28	0.117485	5.41
SseK2	257	276	IAIHVSRKDNHVSLENGIIA	SseK2 alone	1,000 s (23 C)	7.03	0.139174	5.41
SseK2	257	276	IAIHVSRKDNHVSLENGIIA	SseK2 alone	10,000 s (23 C)	7.68	0.107782	5.41
SseK2	257	276	IAIHVSRKDNHVSLENGIIA	SseK2 alone	12 h (28 C)	8.63	0.102703	5.40
SseK2	257	277	IAIHVSRKDNHVSLENGIIAV	SseK2 + YM155	10 s (23 C)	4.40	0.009108	6.02
SseK2	257	277	IAIHVSRKDNHVSLENGIIAV	SseK2 + YM155	100 s (23 C)	6.15	0.032043	6.00
SseK2	257	277	IAIHVSRKDNHVSLENGIIAV	SseK2 + YM155	1,000 s (23 C)	6.99	0.030228	6.00
SseK2	257	277	IAIHVSRKDNHVSLENGIIAV	SseK2 + YM155	10,000 s (23 C)	7.61	0.072767	5.99
SseK2	257	277	IAIHVSRKDNHVSLENGIIAV	SseK2 + YM155	12 h (28 C)	8.84	0.087628	5.99
SseK2	257	277	IAIHVSRKDNHVSLENGIIAV	SseK2 alone	10 s (23 C)	4.39	0.014219	6.06
SseK2	257	277	IAIHVSRKDNHVSLENGIIAV	SseK2 alone	100 s (23 C)	6.20	0.079936	6.05
SseK2	257	277	IAIHVSRKDNHVSLENGIIAV	SseK2 alone	1,000 s (23 C)	6.96	0.07366	6.05
SseK2	257	277	IAIHVSRKDNHVSLENGIIAV	SseK2 alone	10,000 s (23 C)	7.65	0.065132	6.05
SseK2	257	277	IAIHVSRKDNHVSLENGIIAV	SseK2 alone	12 h (28 C)	8.84	0.117626	6.04
SseK2	257	278	IAIHVSRKDNHVSLENGIIAVN	SseK2 + YM155	10 s (23 C)	5.10	0.099548	5.71
SseK2	257	278	IAIHVSRKDNHVSLENGIIAVN	SseK2 + YM155	100 s (23 C)	6.90	0.095901	5.70
SseK2	257	278	IAIHVSRKDNHVSLENGIIAVN	SseK2 + YM155	1,000 s (23 C)	7.69	0.056188	5.70
SseK2	257	278	IAIHVSRKDNHVSLENGIIAVN	SseK2 + YM155	10,000 s (23 C)	8.39	0.097857	5.70
SseK2	257	278	IAIHVSRKDNHVSLENGIIAVN	SseK2 + YM155	12 h (28 C)	9.88	0.001077	5.70
SseK2	257	278	IAIHVSRKDNHVSLENGIIAVN	SseK2 alone	10 s (23 C)	5.09	0.036457	5.74
SseK2	257	278	IAIHVSRKDNHVSLENGIIAVN	SseK2 alone	100 s (23 C)	6.95	0.053869	5.72
SseK2	257	278	IAIHVSRKDNHVSLENGIIAVN	SseK2 alone	1,000 s (23 C)	7.70	0.103075	5.73
SseK2	257	278	IAIHVSRKDNHVSLENGIIAVN	SseK2 alone	10,000 s (23 C)	8.50	0.121911	5.73
SseK2	257	278	IAIHVSRKDNHVSLENGIIAVN	SseK2 alone	12 h (28 C)	9.80	0.027916	5.72
SseK2	257	285	IAIHVSRKDNHVSLENGIIAVNRSEHPAL	SseK2 + YM155	10 s (23 C)	4.58	0.04322	5.50
SseK2	257	285	IAIHVSRKDNHVSLENGIIAVNRSEHPAL	SseK2 + YM155	100 s (23 C)	6.19	0.075781	5.48
SseK2	257	285	IAIHVSRKDNHVSLENGIIAVNRSEHPAL	SseK2 + YM155	1,000 s (23 C)	7.28	0.053101	5.48
SseK2	257	285	IAIHVSRKDNHVSLENGIIAVNRSEHPAL	SseK2 + YM155	10,000 s (23 C)	8.75	0.15255	5.48

SseK2	257	285	IAIHVSRKDNHVSLENGIIAVNRSEHPAL	SseK2 + YM155	12 h (28 C)	11.46	0.084584	5.48
SseK2	257	285	IAIHVSRKDNHVSLENGIIAVNRSEHPAL	SseK2 alone	10 s (23 C)	4.50	0.010877	5.52
SseK2	257	285	IAIHVSRKDNHVSLENGIIAVNRSEHPAL	SseK2 alone	100 s (23 C)	6.34	0.123145	5.50
SseK2	257	285	IAIHVSRKDNHVSLENGIIAVNRSEHPAL	SseK2 alone	1,000 s (23 C)	7.25	0.13364	5.50
SseK2	257	285	IAIHVSRKDNHVSLENGIIAVNRSEHPAL	SseK2 alone	10,000 s (23 C)	8.76	0.089031	5.50
SseK2	257	285	IAIHVSRKDNHVSLENGIIAVNRSEHPAL	SseK2 alone	12 h (28 C)	11.43	0.132567	5.50
SseK2	258	270	AIHVSRKDNHVSL	SseK2 + YM155	10 s (23 C)	3.25	0.062771	4.61
SseK2	258	270	AIHVSRKDNHVSL	SseK2 + YM155	100 s (23 C)	4.05	0.054441	4.59
SseK2	258	270	AIHVSRKDNHVSL	SseK2 + YM155	1,000 s (23 C)	4.45	0.032978	4.59
SseK2	258	270	AIHVSRKDNHVSL	SseK2 + YM155	10,000 s (23 C)	4.57	0.084077	4.60
SseK2	258	270	AIHVSRKDNHVSL	SseK2 + YM155	12 h (28 C)	5.02	0.06893	4.60
SseK2	258	270	AIHVSRKDNHVSL	SseK2 alone	10 s (23 C)	3.26	0.059995	4.62
SseK2	258	270	AIHVSRKDNHVSL	SseK2 alone	100 s (23 C)	4.04	0.054492	4.60
SseK2	258	270	AIHVSRKDNHVSL	SseK2 alone	1,000 s (23 C)	4.48	0.038838	4.60
SseK2	258	270	AIHVSRKDNHVSL	SseK2 alone	10,000 s (23 C)	4.64	0.082149	4.61
SseK2	258	270	AIHVSRKDNHVSL	SseK2 alone	12 h (28 C)	5.03	0.025194	4.60
SseK2	259	275	IHVSRKDNHVSLENGII	SseK2 + YM155	10 s (23 C)	3.88	0.009557	5.38
SseK2	259	275	IHVSRKDNHVSLENGII	SseK2 + YM155	100 s (23 C)	5.26	0.026726	5.35
SseK2	259	275	IHVSRKDNHVSLENGII	SseK2 + YM155	1,000 s (23 C)	5.76	0.027529	5.36
SseK2	259	275	IHVSRKDNHVSLENGII	SseK2 + YM155	10,000 s (23 C)	6.16	0.036426	5.36
SseK2	259	275	IHVSRKDNHVSLENGII	SseK2 + YM155	12 h (28 C)	6.49	0.089889	5.36
SseK2	259	275	IHVSRKDNHVSLENGII	SseK2 alone	10 s (23 C)	3.96	0.000095	5.41
SseK2	259	275	IHVSRKDNHVSLENGII	SseK2 alone	100 s (23 C)	5.47	0.115162	5.39
SseK2	259	275	IHVSRKDNHVSLENGII	SseK2 alone	1,000 s (23 C)	5.98	0.067323	5.40
SseK2	259	275	IHVSRKDNHVSLENGII	SseK2 alone	10,000 s (23 C)	6.32	0.059562	5.40
SseK2	259	275	IHVSRKDNHVSLENGII	SseK2 alone	12 h (28 C)	6.62	0.154918	5.39
SseK2	259	276	IHVSRKDNHVSLENGIIA	SseK2 + YM155	10 s (23 C)	3.90	0.022427	5.07
SseK2	259	276	IHVSRKDNHVSLENGIIA	SseK2 + YM155	100 s (23 C)	5.29	0.03031	5.05

SseK2	259	276	IHVSRKDNHVSLENGIIA	SseK2 + YM155	1,000 s (23 C)	5.81	0.017704	5.05
SseK2	259	276	IHVSRKDNHVSLENGIIA	SseK2 + YM155	10,000 s (23 C)	6.19	0.070086	5.05
SseK2	259	276	IHVSRKDNHVSLENGIIA	SseK2 + YM155	12 h (28 C)	6.76	0.044065	5.05
SseK2	259	276	IHVSRKDNHVSLENGIIA	SseK2 alone	10 s (23 C)	3.96	0.009628	5.09
SseK2	259	276	IHVSRKDNHVSLENGIIA	SseK2 alone	100 s (23 C)	5.50	0.090885	5.07
SseK2	259	276	IHVSRKDNHVSLENGIIA	SseK2 alone	1,000 s (23 C)	5.99	0.09269	5.08
SseK2	259	276	IHVSRKDNHVSLENGIIA	SseK2 alone	10,000 s (23 C)	6.40	0.060542	5.08
SseK2	259	276	IHVSRKDNHVSLENGIIA	SseK2 alone	12 h (28 C)	6.91	0.133795	5.07
SseK2	259	277	IHVSRKDNHVSLENGIIAV	SseK2 + YM155	10 s (23 C)	3.85	0.032236	5.71
SseK2	259	277	IHVSRKDNHVSLENGIIAV	SseK2 + YM155	100 s (23 C)	5.29	0.078486	5.71
SseK2	259	277	IHVSRKDNHVSLENGIIAV	SseK2 + YM155	1,000 s (23 C)	5.89	0.083296	5.70
SseK2	259	277	IHVSRKDNHVSLENGIIAV	SseK2 + YM155	10,000 s (23 C)	6.32	0.040667	5.69
SseK2	259	277	IHVSRKDNHVSLENGIIAV	SseK2 + YM155	12 h (28 C)	7.05	0.042466	5.70
SseK2	259	277	IHVSRKDNHVSLENGIIAV	SseK2 alone	10 s (23 C)	3.82	0.009448	5.75
SseK2	259	277	IHVSRKDNHVSLENGIIAV	SseK2 alone	100 s (23 C)	5.49	0.090138	5.73
SseK2	259	277	IHVSRKDNHVSLENGIIAV	SseK2 alone	1,000 s (23 C)	5.94	0.180848	5.73
SseK2	259	277	IHVSRKDNHVSLENGIIAV	SseK2 alone	10,000 s (23 C)	6.44	0.145038	5.74
SseK2	259	277	IHVSRKDNHVSLENGIIAV	SseK2 alone	12 h (28 C)	7.14	0.016677	5.73
SseK2	259	285	IHVSRKDNHVSLENGIIAVNRSEHPAL	SseK2 + YM155	10 s (23 C)	3.69	0.030138	5.37
SseK2	259	285	IHVSRKDNHVSLENGIIAVNRSEHPAL	SseK2 + YM155	100 s (23 C)	5.11	0.025988	5.34
SseK2	259	285	IHVSRKDNHVSLENGIIAVNRSEHPAL	SseK2 + YM155	1,000 s (23 C)	5.89	0.041909	5.35
SseK2	259	285	IHVSRKDNHVSLENGIIAVNRSEHPAL	SseK2 + YM155	10,000 s (23 C)	7.27	0.10154	5.35
SseK2	259	285	IHVSRKDNHVSLENGIIAVNRSEHPAL	SseK2 + YM155	12 h (28 C)	9.59	0.077239	5.34
SseK2	259	285	IHVSRKDNHVSLENGIIAVNRSEHPAL	SseK2 alone	10 s (23 C)	3.79	0.172051	5.38
SseK2	259	285	IHVSRKDNHVSLENGIIAVNRSEHPAL	SseK2 alone	100 s (23 C)	5.17	0.030327	5.37
SseK2	259	285	IHVSRKDNHVSLENGIIAVNRSEHPAL	SseK2 alone	1,000 s (23 C)	5.84	0.093135	5.37
SseK2	259	285	IHVSRKDNHVSLENGIIAVNRSEHPAL	SseK2 alone	10,000 s (23 C)	7.24	0.022823	5.37
SseK2	259	285	IHVSRKDNHVSLENGIIAVNRSEHPAL	SseK2 alone	12 h (28 C)	9.51	0.063415	5.36

SseK2	261	280	VSRKDNHVSLENGIIAVNRS	SseK2 + YM155	10 s (23 C)	4.41	0.015727	5.40
SseK2	261	280	VSRKDNHVSLENGIIAVNRS	SseK2 + YM155	100 s (23 C)	6.20	0.041848	5.38
SseK2	261	280	VSRKDNHVSLENGIIAVNRS	SseK2 + YM155	1,000 s (23 C)	6.92	0.023419	5.38
SseK2	261	280	VSRKDNHVSLENGIIAVNRS	SseK2 + YM155	10,000 s (23 C)	7.53	0.089429	5.38
SseK2	261	280	VSRKDNHVSLENGIIAVNRS	SseK2 + YM155	12 h (28 C)	8.54	0.124179	5.38
SseK2	261	280	VSRKDNHVSLENGIIAVNRS	SseK2 alone	10 s (23 C)	4.38	0.04765	5.42
SseK2	261	280	VSRKDNHVSLENGIIAVNRS	SseK2 alone	100 s (23 C)	6.21	0.142554	5.41
SseK2	261	280	VSRKDNHVSLENGIIAVNRS	SseK2 alone	1,000 s (23 C)	7.01	0.138844	5.41
SseK2	261	280	VSRKDNHVSLENGIIAVNRS	SseK2 alone	10,000 s (23 C)	7.62	0.1077	5.41
SseK2	261	280	VSRKDNHVSLENGIIAVNRS	SseK2 alone	12 h (28 C)	8.64	0.152161	5.40
SseK2	265	271	DNHVSLE	SseK2 + YM155	10 s (23 C)	0.72	0.122374	4.49
SseK2	265	271	DNHVSLE	SseK2 + YM155	100 s (23 C)	1.16	0.014667	4.47
SseK2	265	271	DNHVSLE	SseK2 + YM155	1,000 s (23 C)	1.98	0.019238	4.47
SseK2	265	271	DNHVSLE	SseK2 + YM155	10,000 s (23 C)	2.60	0.020001	4.48
SseK2	265	271	DNHVSLE	SseK2 + YM155	12 h (28 C)	2.52	0.01823	4.47
SseK2	265	271	DNHVSLE	SseK2 alone	10 s (23 C)	0.74	0.077654	4.48
SseK2	265	271	DNHVSLE	SseK2 alone	100 s (23 C)	1.24	0.039682	4.47
SseK2	265	271	DNHVSLE	SseK2 alone	1,000 s (23 C)	2.06	0.092968	4.47
SseK2	265	271	DNHVSLE	SseK2 alone	10,000 s (23 C)	2.63	0.057383	4.47
SseK2	265	271	DNHVSLE	SseK2 alone	12 h (28 C)	2.57	0.022202	4.46
SseK2	272	285	NGIIAVNRSEHPAL	SseK2 + YM155	10 s (23 C)	0.41	0.089903	5.27
SseK2	272	285	NGIIAVNRSEHPAL	SseK2 + YM155	100 s (23 C)	0.44	0.018227	5.25
SseK2	272	285	NGIIAVNRSEHPAL	SseK2 + YM155	1,000 s (23 C)	0.96	0.039039	5.25
SseK2	272	285	NGIIAVNRSEHPAL	SseK2 + YM155	10,000 s (23 C)	2.37	0.034614	5.25
SseK2	272	285	NGIIAVNRSEHPAL	SseK2 + YM155	12 h (28 C)	4.32	0.114307	5.25
SseK2	272	285	NGIIAVNRSEHPAL	SseK2 alone	10 s (23 C)	0.42	0.037046	5.29
SseK2	272	285	NGIIAVNRSEHPAL	SseK2 alone	100 s (23 C)	0.47	0.01826	5.28
SseK2	272	285	NGIIAVNRSEHPAL	SseK2 alone	1,000 s (23 C)	1.00	0.036809	5.28

SseK2	272	285	NGIIAVNRSEHPAL	SseK2 alone	10,000 s (23 C)	2.40	0.023439	5.29
SseK2	272	285	NGIIAVNRSEHPAL	SseK2 alone	12 h (28 C)	4.29	0.062536	5.28
SseK2	274	285	IIAVNRSEHPAL	SseK2 + YM155	10 s (23 C)	0.32	0.010023	4.88
SseK2	274	285	IIAVNRSEHPAL	SseK2 + YM155	100 s (23 C)	0.36	0.017564	4.85
SseK2	274	285	IIAVNRSEHPAL	SseK2 + YM155	1,000 s (23 C)	0.64	0.073323	4.86
SseK2	274	285	IIAVNRSEHPAL	SseK2 + YM155	10,000 s (23 C)	1.50	0.026017	4.86
SseK2	274	285	IIAVNRSEHPAL	SseK2 + YM155	12 h (28 C)	3.26	0.040384	4.85
SseK2	274	285	IIAVNRSEHPAL	SseK2 alone	10 s (23 C)	0.36	0.037857	4.88
SseK2	274	285	IIAVNRSEHPAL	SseK2 alone	100 s (23 C)	0.41	0.015729	4.87
SseK2	274	285	IIAVNRSEHPAL	SseK2 alone	1,000 s (23 C)	0.63	0.028229	4.87
SseK2	274	285	IIAVNRSEHPAL	SseK2 alone	10,000 s (23 C)	1.51	0.035822	4.88
SseK2	274	285	IIAVNRSEHPAL	SseK2 alone	12 h (28 C)	3.19	0.01011	4.87
SseK2	275	284	IAVNRSEHPA	SseK2 + YM155	10 s (23 C)	0.31	0.013132	4.87
SseK2	275	284	IAVNRSEHPA	SseK2 + YM155	100 s (23 C)	0.30	0.008516	4.85
SseK2	275	284	IAVNRSEHPA	SseK2 + YM155	1,000 s (23 C)	0.54	0.066733	4.86
SseK2	275	284	IAVNRSEHPA	SseK2 + YM155	10,000 s (23 C)	1.29	0.037381	4.86
SseK2	275	284	IAVNRSEHPA	SseK2 + YM155	12 h (28 C)	2.88	0.061425	4.85
SseK2	275	284	IAVNRSEHPA	SseK2 alone	10 s (23 C)	0.29	0.028282	4.88
SseK2	275	284	IAVNRSEHPA	SseK2 alone	100 s (23 C)	0.36	0.020917	4.87
SseK2	275	284	IAVNRSEHPA	SseK2 alone	1,000 s (23 C)	0.55	0.017128	4.87
SseK2	275	284	IAVNRSEHPA	SseK2 alone	10,000 s (23 C)	1.33	0.005346	4.88
SseK2	275	284	IAVNRSEHPA	SseK2 alone	12 h (28 C)	2.87	0.028225	4.87
SseK2	275	285	IAVNRSEHPAL	SseK2 + YM155	10 s (23 C)	0.31	0.002718	4.88
SseK2	275	285	IAVNRSEHPAL	SseK2 + YM155	100 s (23 C)	0.31	0.021614	4.85
SseK2	275	285	IAVNRSEHPAL	SseK2 + YM155	1,000 s (23 C)	0.55	0.011938	4.86
SseK2	275	285	IAVNRSEHPAL	SseK2 + YM155	10,000 s (23 C)	1.41	0.04587	4.86
SseK2	275	285	IAVNRSEHPAL	SseK2 + YM155	12 h (28 C)	3.02	0.003643	4.85
SseK2	275	285	IAVNRSEHPAL	SseK2 alone	10 s (23 C)	0.34	0.037342	4.89

SseK2	275	285	IAVNRSEHPAL	SseK2 alone	100 s (23 C)	0.35	0.02884	4.87
SseK2	275	285	IAVNRSEHPAL	SseK2 alone	1,000 s (23 C)	0.61	0.049997	4.87
SseK2	275	285	IAVNRSEHPAL	SseK2 alone	10,000 s (23 C)	1.43	0.01166	4.87
SseK2	275	285	IAVNRSEHPAL	SseK2 alone	12 h (28 C)	2.98	0.018641	4.86
SseK2	276	285	AVNRSEHPAL	SseK2 + YM155	10 s (23 C)	0.40	0.025619	3.96
SseK2	276	285	AVNRSEHPAL	SseK2 + YM155	100 s (23 C)	0.37	0.02287	3.95
SseK2	276	285	AVNRSEHPAL	SseK2 + YM155	1,000 s (23 C)	0.60	0.01506	3.94
SseK2	276	285	AVNRSEHPAL	SseK2 + YM155	10,000 s (23 C)	1.38	0.025671	3.95
SseK2	276	285	AVNRSEHPAL	SseK2 + YM155	12 h (28 C)	2.64	0.064567	3.94
SseK2	276	285	AVNRSEHPAL	SseK2 alone	10 s (23 C)	0.42	0.024657	3.96
SseK2	276	285	AVNRSEHPAL	SseK2 alone	100 s (23 C)	0.44	0.01647	3.94
SseK2	276	285	AVNRSEHPAL	SseK2 alone	1,000 s (23 C)	0.65	0.04081	3.94
SseK2	276	285	AVNRSEHPAL	SseK2 alone	10,000 s (23 C)	1.46	0.043922	3.94
SseK2	276	285	AVNRSEHPAL	SseK2 alone	12 h (28 C)	2.70	0.084488	3.94
SseK2	276	286	AVNRSEHPALI	SseK2 + YM155	10 s (23 C)	0.66	0.01809	5.50
SseK2	276	286	AVNRSEHPALI	SseK2 + YM155	100 s (23 C)	0.81	0.023422	5.48
SseK2	276	286	AVNRSEHPALI	SseK2 + YM155	1,000 s (23 C)	1.18	0.021839	5.48
SseK2	276	286	AVNRSEHPALI	SseK2 + YM155	10,000 s (23 C)	2.27	0.052483	5.48
SseK2	276	286	AVNRSEHPALI	SseK2 + YM155	12 h (28 C)	3.70	0.018932	5.48
SseK2	276	286	AVNRSEHPALI	SseK2 alone	10 s (23 C)	0.66	0.012465	5.52
SseK2	276	286	AVNRSEHPALI	SseK2 alone	100 s (23 C)	0.83	0.021135	5.50
SseK2	276	286	AVNRSEHPALI	SseK2 alone	1,000 s (23 C)	1.25	0.028277	5.50
SseK2	276	286	AVNRSEHPALI	SseK2 alone	10,000 s (23 C)	2.25	0.006624	5.50
SseK2	276	286	AVNRSEHPALI	SseK2 alone	12 h (28 C)	3.78	0.014181	5.49
SseK2	276	288	AVNRSEHPALIKG	SseK2 + YM155	10 s (23 C)	0.30	0.061561	3.61
SseK2	276	288	AVNRSEHPALIKG	SseK2 + YM155	100 s (23 C)	0.32	0.030805	3.60
SseK2	276	288	AVNRSEHPALIKG	SseK2 + YM155	1,000 s (23 C)	0.87	0.068214	3.60
SseK2	276	288	AVNRSEHPALIKG	SseK2 + YM155	10,000 s (23 C)	2.63	0.063328	3.61

SseK2	276	288	AVNRSEHPALIKG	SseK2 + YM155	12 h (28 C)	4.17	0.079695	3.61
SseK2	276	288	AVNRSEHPALIKG	SseK2 alone	10 s (23 C)	0.33	0.003076	3.62
SseK2	276	288	AVNRSEHPALIKG	SseK2 alone	100 s (23 C)	0.38	0.014328	3.60
SseK2	276	288	AVNRSEHPALIKG	SseK2 alone	1,000 s (23 C)	0.89	0.032455	3.60
SseK2	276	288	AVNRSEHPALIKG	SseK2 alone	10,000 s (23 C)	2.67	0.022138	3.36
SseK2	276	288	AVNRSEHPALIKG	SseK2 alone	12 h (28 C)	4.12	0.012428	3.60
SseK2	277	285	VNRSEHPAL	SseK2 + YM155	10 s (23 C)	0.40	0.002191	3.87
SseK2	277	285	VNRSEHPAL	SseK2 + YM155	100 s (23 C)	0.40	0.027152	3.86
SseK2	277	285	VNRSEHPAL	SseK2 + YM155	1,000 s (23 C)	0.58	0.057029	3.86
SseK2	277	285	VNRSEHPAL	SseK2 + YM155	10,000 s (23 C)	1.22	0.039692	3.87
SseK2	277	285	VNRSEHPAL	SseK2 + YM155	12 h (28 C)	2.24	0.005722	3.86
SseK2	277	285	VNRSEHPAL	SseK2 alone	10 s (23 C)	0.41	0.037507	3.87
SseK2	277	285	VNRSEHPAL	SseK2 alone	100 s (23 C)	0.46	0.045759	3.86
SseK2	277	285	VNRSEHPAL	SseK2 alone	1,000 s (23 C)	0.59	0.0199	3.86
SseK2	277	285	VNRSEHPAL	SseK2 alone	10,000 s (23 C)	1.29	0.032236	3.85
SseK2	277	285	VNRSEHPAL	SseK2 alone	12 h (28 C)	2.31	0.003542	3.86
SseK2	278	285	NRSEHPAL	SseK2 + YM155	10 s (23 C)	0.30	0.026208	3.87
SseK2	278	285	NRSEHPAL	SseK2 + YM155	100 s (23 C)	0.31	0.040327	3.86
SseK2	278	285	NRSEHPAL	SseK2 + YM155	1,000 s (23 C)	0.52	0.009419	3.85
SseK2	278	285	NRSEHPAL	SseK2 + YM155	10,000 s (23 C)	1.14	0.037834	3.86
SseK2	278	285	NRSEHPAL	SseK2 + YM155	12 h (28 C)	2.12	0.032285	3.86
SseK2	278	285	NRSEHPAL	SseK2 alone	10 s (23 C)	0.31	0.037823	3.87
SseK2	278	285	NRSEHPAL	SseK2 alone	100 s (23 C)	0.36	0.006461	3.85
SseK2	278	285	NRSEHPAL	SseK2 alone	1,000 s (23 C)	0.54	0.017879	3.86
SseK2	278	285	NRSEHPAL	SseK2 alone	10,000 s (23 C)	1.20	0.027559	3.85
SseK2	278	285	NRSEHPAL	SseK2 alone	12 h (28 C)	2.13	0.04025	3.85
SseK2	279	285	RSEHPAL	SseK2 + YM155	10 s (23 C)	0.24	0.089454	3.67
SseK2	279	285	RSEHPAL	SseK2 + YM155	100 s (23 C)	0.14	0.016192	3.65

SseK2	279	285	RSEHPAL	SseK2 + YM155	1,000 s (23 C)	0.24	0.055903	3.65
SseK2	279	285	RSEHPAL	SseK2 + YM155	10,000 s (23 C)	0.50	0.034951	3.66
SseK2	279	285	RSEHPAL	SseK2 + YM155	12 h (28 C)	1.18	0.035928	3.66
SseK2	279	285	RSEHPAL	SseK2 alone	10 s (23 C)	0.20	0.012664	3.67
SseK2	279	285	RSEHPAL	SseK2 alone	100 s (23 C)	0.15	0.014321	3.65
SseK2	279	285	RSEHPAL	SseK2 alone	1,000 s (23 C)	0.18	0.019243	3.65
SseK2	279	285	RSEHPAL	SseK2 alone	10,000 s (23 C)	0.50	0.027434	3.64
SseK2	279	285	RSEHPAL	SseK2 alone	12 h (28 C)	1.10	0.015778	3.65
SseK2	286	292	IKGLEIM	SseK2 + YM155	10 s (23 C)	0.20	0.022956	6.50
SseK2	286	292	IKGLEIM	SseK2 + YM155	100 s (23 C)	0.25	0.008263	6.49
SseK2	286	292	IKGLEIM	SseK2 + YM155	1,000 s (23 C)	1.02	0.03402	6.47
SseK2	286	292	IKGLEIM	SseK2 + YM155	10,000 s (23 C)	2.67	0.011027	6.46
SseK2	286	292	IKGLEIM	SseK2 + YM155	12 h (28 C)	3.13	0.032267	6.48
SseK2	286	292	IKGLEIM	SseK2 alone	10 s (23 C)	0.19	0.013088	6.60
SseK2	286	292	IKGLEIM	SseK2 alone	100 s (23 C)	0.29	0.017915	6.59
SseK2	286	292	IKGLEIM	SseK2 alone	1,000 s (23 C)	0.97	0.007434	6.59
SseK2	286	292	IKGLEIM	SseK2 alone	10,000 s (23 C)	2.69	0.041132	6.59
SseK2	286	292	IKGLEIM	SseK2 alone	12 h (28 C)	3.18	0.012927	6.58
SseK2	286	304	IKGLEIMHSKPYGDPYNDW	SseK2 + YM155	10 s (23 C)	3.01	0.123672	6.58
SseK2	286	304	IKGLEIMHSKPYGDPYNDW	SseK2 + YM155	100 s (23 C)	4.32	0.047479	6.58
SseK2	286	304	IKGLEIMHSKPYGDPYNDW	SseK2 + YM155	1,000 s (23 C)	5.05	0.092483	6.57
SseK2	286	304	IKGLEIMHSKPYGDPYNDW	SseK2 + YM155	10,000 s (23 C)	6.40	0.128577	6.57
SseK2	286	304	IKGLEIMHSKPYGDPYNDW	SseK2 + YM155	12 h (28 C)	7.09	0.196481	6.57
SseK2	286	304	IKGLEIMHSKPYGDPYNDW	SseK2 alone	10 s (23 C)	3.16	0.062215	6.67
SseK2	286	304	IKGLEIMHSKPYGDPYNDW	SseK2 alone	100 s (23 C)	4.43	0.059578	6.66
SseK2	286	304	IKGLEIMHSKPYGDPYNDW	SseK2 alone	1,000 s (23 C)	5.34	0.077648	6.67
SseK2	286	304	IKGLEIMHSKPYGDPYNDW	SseK2 alone	10,000 s (23 C)	6.75	0.07792	6.67
SseK2	286	304	IKGLEIMHSKPYGDPYNDW	SseK2 alone	12 h (28 C)	7.44	0.046172	6.66

SseK2	289	303	LEIMHSKPYGDPYND	SseK2 + YM155	10 s (23 C)	3.16	0.022964	5.45
SseK2	289	303	LEIMHSKPYGDPYND	SseK2 + YM155	100 s (23 C)	3.89	0.022587	5.44
SseK2	289	303	LEIMHSKPYGDPYND	SseK2 + YM155	1,000 s (23 C)	4.60	0.010557	5.43
SseK2	289	303	LEIMHSKPYGDPYND	SseK2 + YM155	10,000 s (23 C)	5.28	0.033238	5.43
SseK2	289	303	LEIMHSKPYGDPYND	SseK2 + YM155	12 h (28 C)	5.35	0.063233	5.43
SseK2	289	303	LEIMHSKPYGDPYND	SseK2 alone	10 s (23 C)	3.15	0.039329	5.47
SseK2	289	303	LEIMHSKPYGDPYND	SseK2 alone	100 s (23 C)	3.95	0.035157	5.47
SseK2	289	303	LEIMHSKPYGDPYND	SseK2 alone	1,000 s (23 C)	4.63	0.049303	5.46
SseK2	289	303	LEIMHSKPYGDPYND	SseK2 alone	10,000 s (23 C)	5.36	0.039894	5.47
SseK2	289	303	LEIMHSKPYGDPYND	SseK2 alone	12 h (28 C)	5.44	0.05175	5.45
SseK2	289	304	LEIMHSKPYGDPYNDW	SseK2 + YM155	10 s (23 C)	2.94	0.008333	6.80
SseK2	289	304	LEIMHSKPYGDPYNDW	SseK2 + YM155	100 s (23 C)	4.02	0.015324	6.77
SseK2	289	304	LEIMHSKPYGDPYNDW	SseK2 + YM155	1,000 s (23 C)	4.81	0.030342	6.78
SseK2	289	304	LEIMHSKPYGDPYNDW	SseK2 + YM155	10,000 s (23 C)	5.56	0.020155	6.78
SseK2	289	304	LEIMHSKPYGDPYNDW	SseK2 + YM155	12 h (28 C)	5.62	0.061134	6.78
SseK2	289	304	LEIMHSKPYGDPYNDW	SseK2 alone	10 s (23 C)	2.97	0.033465	6.84
SseK2	289	304	LEIMHSKPYGDPYNDW	SseK2 alone	100 s (23 C)	4.15	0.048905	6.82
SseK2	289	304	LEIMHSKPYGDPYNDW	SseK2 alone	1,000 s (23 C)	4.92	0.057016	6.83
SseK2	289	304	LEIMHSKPYGDPYNDW	SseK2 alone	10,000 s (23 C)	5.66	0.032337	6.83
SseK2	289	304	LEIMHSKPYGDPYNDW	SseK2 alone	12 h (28 C)	5.77	0.086798	6.82
SseK2	289	305	LEIMHSKPYGDPYNDWL	SseK2 + YM155	10 s (23 C)	3.06	0.044177	7.60
SseK2	289	305	LEIMHSKPYGDPYNDWL	SseK2 + YM155	100 s (23 C)	4.44	0.034759	7.57
SseK2	289	305	LEIMHSKPYGDPYNDWL	SseK2 + YM155	1,000 s (23 C)	5.67	0.031148	7.58
SseK2	289	305	LEIMHSKPYGDPYNDWL	SseK2 + YM155	10,000 s (23 C)	6.39	0.046308	7.58
SseK2	289	305	LEIMHSKPYGDPYNDWL	SseK2 + YM155	12 h (28 C)	6.56	0.072196	7.58
SseK2	289	305	LEIMHSKPYGDPYNDWL	SseK2 alone	10 s (23 C)	3.11	0.061443	7.58
SseK2	289	305	LEIMHSKPYGDPYNDWL	SseK2 alone	100 s (23 C)	4.49	0.068965	7.58
SseK2	289	305	LEIMHSKPYGDPYNDWL	SseK2 alone	1,000 s (23 C)	5.72	0.026521	7.58

SseK2	289	305	LEIMHSKPYGDPYNDWL	SseK2 alone	10,000 s (23 C)	6.49	0.034417	7.58
SseK2	289	305	LEIMHSKPYGDPYNDWL	SseK2 alone	12 h (28 C)	6.60	0.094663	7.58
SseK2	289	316	LEIMHSKPYGDPYNDWLSKGLRHYFDGS	SseK2 + YM155	10 s (23 C)	4.44	0.077732	7.43
SseK2	289	316	LEIMHSKPYGDPYNDWLSKGLRHYFDGS	SseK2 + YM155	100 s (23 C)	7.52	0.007309	7.39
SseK2	289	316	LEIMHSKPYGDPYNDWLSKGLRHYFDGS	SseK2 + YM155	1,000 s (23 C)	10.02	0.060864	7.39
SseK2	289	316	LEIMHSKPYGDPYNDWLSKGLRHYFDGS	SseK2 + YM155	10,000 s (23 C)	12.02	0.052155	7.39
SseK2	289	316	LEIMHSKPYGDPYNDWLSKGLRHYFDGS	SseK2 + YM155	12 h (28 C)	12.95	0.147607	7.38
SseK2	289	316	LEIMHSKPYGDPYNDWLSKGLRHYFDGS	SseK2 alone	10 s (23 C)	4.45	0.122775	7.43
SseK2	289	316	LEIMHSKPYGDPYNDWLSKGLRHYFDGS	SseK2 alone	100 s (23 C)	7.53	0.131457	7.41
SseK2	289	316	LEIMHSKPYGDPYNDWLSKGLRHYFDGS	SseK2 alone	1,000 s (23 C)	9.96	0.126961	7.40
SseK2	289	316	LEIMHSKPYGDPYNDWLSKGLRHYFDGS	SseK2 alone	10,000 s (23 C)	12.01	0.172561	7.39
SseK2	289	316	LEIMHSKPYGDPYNDWLSKGLRHYFDGS	SseK2 alone	12 h (28 C)	13.06	0.088548	7.38
SseK2	291	304	IMHSKPYGDPYNDW	SseK2 + YM155	10 s (23 C)	2.74	0.124829	6.32
SseK2	291	304	IMHSKPYGDPYNDW	SseK2 + YM155	100 s (23 C)	3.63	0.054496	6.31
SseK2	291	304	IMHSKPYGDPYNDW	SseK2 + YM155	1,000 s (23 C)	3.92	0.026421	6.29
SseK2	291	304	IMHSKPYGDPYNDW	SseK2 + YM155	10,000 s (23 C)	3.93	0.069698	6.30
SseK2	291	304	IMHSKPYGDPYNDW	SseK2 + YM155	12 h (28 C)	3.96	0.059974	6.29
SseK2	291	304	IMHSKPYGDPYNDW	SseK2 alone	10 s (23 C)	2.70	0.023967	6.38
SseK2	291	304	IMHSKPYGDPYNDW	SseK2 alone	100 s (23 C)	3.67	0.048678	6.37
SseK2	291	304	IMHSKPYGDPYNDW	SseK2 alone	1,000 s (23 C)	3.98	0.055192	6.37
SseK2	291	304	IMHSKPYGDPYNDW	SseK2 alone	10,000 s (23 C)	3.98	0.026767	6.38
SseK2	291	304	IMHSKPYGDPYNDW	SseK2 alone	12 h (28 C)	4.04	0.117808	6.37
SseK2	291	320	IMHSKPYGDPYNDWLSKGLRHYFDGSHIQD	SseK2 + YM155	10 s (23 C)	4.70	0.242401	7.05
SseK2	291	320	IMHSKPYGDPYNDWLSKGLRHYFDGSHIQD	SseK2 + YM155	100 s (23 C)	6.75	0.104282	7.02
SseK2	291	320	IMHSKPYGDPYNDWLSKGLRHYFDGSHIQD	SseK2 + YM155	1,000 s (23 C)	8.23	0.033954	7.01
SseK2	291	320	IMHSKPYGDPYNDWLSKGLRHYFDGSHIQD	SseK2 + YM155	10,000 s (23 C)	9.10	0.037423	7.00
SseK2	291	320	IMHSKPYGDPYNDWLSKGLRHYFDGSHIQD	SseK2 + YM155	12 h (28 C)	9.94	0.176737	7.00
SseK2	291	320	IMHSKPYGDPYNDWLSKGLRHYFDGSHIQD	SseK2 alone	10 s (23 C)	5.60	0.179804	7.04

SseK2	291	320	IMHSKPYGDPYNDWLSKGLRHYFDGSHIQD	SseK2 alone	100 s (23 C)	8.47	0.12832	7.01
SseK2	291	320	IMHSKPYGDPYNDWLSKGLRHYFDGSHIQD	SseK2 alone	1,000 s (23 C)	10.18	0.187857	7.01
SseK2	291	320	IMHSKPYGDPYNDWLSKGLRHYFDGSHIQD	SseK2 alone	10,000 s (23 C)	11.21	0.120989	7.00
SseK2	291	320	IMHSKPYGDPYNDWLSKGLRHYFDGSHIQD	SseK2 alone	12 h (28 C)	12.38	0.111514	7.00
SseK2	293	304	HSKPYGDPYNDW	SseK2 + YM155	10 s (23 C)	2.60	0.075715	6.17
SseK2	293	304	HSKPYGDPYNDW	SseK2 + YM155	100 s (23 C)	3.15	0.059034	6.14
SseK2	293	304	HSKPYGDPYNDW	SseK2 + YM155	1,000 s (23 C)	3.35	0.02089	6.15
SseK2	293	304	HSKPYGDPYNDW	SseK2 + YM155	10,000 s (23 C)	3.31	0.019625	6.15
SseK2	293	304	HSKPYGDPYNDW	SseK2 + YM155	12 h (28 C)	3.33	0.079385	6.15
SseK2	293	304	HSKPYGDPYNDW	SseK2 alone	10 s (23 C)	2.61	0.030852	6.21
SseK2	293	304	HSKPYGDPYNDW	SseK2 alone	100 s (23 C)	3.27	0.034247	6.19
SseK2	293	304	HSKPYGDPYNDW	SseK2 alone	1,000 s (23 C)	3.45	0.061565	6.20
SseK2	293	304	HSKPYGDPYNDW	SseK2 alone	10,000 s (23 C)	3.41	0.046611	6.20
SseK2	293	304	HSKPYGDPYNDW	SseK2 alone	12 h (28 C)	3.47	0.049361	6.19
SseK2	293	316	HSKPYGDPYNDWLSKGLRHYFDGS	SseK2 + YM155	10 s (23 C)	4.48	0.090601	7.27
SseK2	293	316	HSKPYGDPYNDWLSKGLRHYFDGS	SseK2 + YM155	100 s (23 C)	7.47	0.026066	7.23
SseK2	293	316	HSKPYGDPYNDWLSKGLRHYFDGS	SseK2 + YM155	1,000 s (23 C)	9.49	0.064835	7.23
SseK2	293	316	HSKPYGDPYNDWLSKGLRHYFDGS	SseK2 + YM155	10,000 s (23 C)	10.86	0.057007	7.22
SseK2	293	316	HSKPYGDPYNDWLSKGLRHYFDGS	SseK2 + YM155	12 h (28 C)	11.82	0.092985	7.21
SseK2	293	316	HSKPYGDPYNDWLSKGLRHYFDGS	SseK2 alone	10 s (23 C)	4.60	0.046969	7.24
SseK2	293	316	HSKPYGDPYNDWLSKGLRHYFDGS	SseK2 alone	100 s (23 C)	7.64	0.129594	7.23
SseK2	293	316	HSKPYGDPYNDWLSKGLRHYFDGS	SseK2 alone	1,000 s (23 C)	9.61	0.090035	7.22
SseK2	293	316	HSKPYGDPYNDWLSKGLRHYFDGS	SseK2 alone	10,000 s (23 C)	10.89	0.090974	7.22
SseK2	293	316	HSKPYGDPYNDWLSKGLRHYFDGS	SseK2 alone	12 h (28 C)	11.92	0.079556	7.21
SseK2	293	320	HSKPYGDPYNDWLSKGLRHYFDGSHIQD	SseK2 + YM155	10 s (23 C)	6.09	0.014839	7.00
SseK2	293	320	HSKPYGDPYNDWLSKGLRHYFDGSHIQD	SseK2 + YM155	100 s (23 C)	9.04	0.063145	6.96
SseK2	293	320	HSKPYGDPYNDWLSKGLRHYFDGSHIQD	SseK2 + YM155	1,000 s (23 C)	10.87	0.134702	6.96
SseK2	293	320	HSKPYGDPYNDWLSKGLRHYFDGSHIQD	SseK2 + YM155	10,000 s (23 C)	12.11	0.120501	6.95

SseK2	293	320	HSKPYGDPYNDWLSKGLRHYFDGSHIQD	SseK2 + YM155	12 h (28 C)	12.95	0.31217	6.94
SseK2	293	320	HSKPYGDPYNDWLSKGLRHYFDGSHIQD	SseK2 alone	10 s (23 C)	6.39	0.017421	7.00
SseK2	293	320	HSKPYGDPYNDWLSKGLRHYFDGSHIQD	SseK2 alone	100 s (23 C)	9.52	0.133583	6.97
SseK2	293	320	HSKPYGDPYNDWLSKGLRHYFDGSHIQD	SseK2 alone	1,000 s (23 C)	11.51	0.137536	6.97
SseK2	293	320	HSKPYGDPYNDWLSKGLRHYFDGSHIQD	SseK2 alone	10,000 s (23 C)	12.83	0.131685	6.96
SseK2	293	320	HSKPYGDPYNDWLSKGLRHYFDGSHIQD	SseK2 alone	12 h (28 C)	13.91	0.171845	6.95
SseK2	293	321	HSKPYGDPYNDWLSKGLRHYFDGSHIQDY	SseK2 + YM155	10 s (23 C)	6.73	0.079767	7.19
SseK2	293	321	HSKPYGDPYNDWLSKGLRHYFDGSHIQDY	SseK2 + YM155	100 s (23 C)	9.36	0.230711	7.17
SseK2	293	321	HSKPYGDPYNDWLSKGLRHYFDGSHIQDY	SseK2 + YM155	1,000 s (23 C)	11.56	0.242033	7.16
SseK2	293	321	HSKPYGDPYNDWLSKGLRHYFDGSHIQDY	SseK2 + YM155	10,000 s (23 C)	12.09	0.362537	7.16
SseK2	293	321	HSKPYGDPYNDWLSKGLRHYFDGSHIQDY	SseK2 + YM155	12 h (28 C)	13.09	ND	7.16
SseK2	293	321	HSKPYGDPYNDWLSKGLRHYFDGSHIQDY	SseK2 alone	10 s (23 C)	6.77	0.096138	7.18
SseK2	293	321	HSKPYGDPYNDWLSKGLRHYFDGSHIQDY	SseK2 alone	100 s (23 C)	9.63	0.084369	7.16
SseK2	293	321	HSKPYGDPYNDWLSKGLRHYFDGSHIQDY	SseK2 alone	1,000 s (23 C)	12.10	0.00253	7.15
SseK2	293	321	HSKPYGDPYNDWLSKGLRHYFDGSHIQDY	SseK2 alone	10,000 s (23 C)	12.77	0.121632	7.14
SseK2	293	321	HSKPYGDPYNDWLSKGLRHYFDGSHIQDY	SseK2 alone	12 h (28 C)	13.85	0.172319	7.14
SseK2	304	320	WLSKGLRHYFDGSHIQD	SseK2 + YM155	10 s (23 C)	3.15	0.027141	5.88
SseK2	304	320	WLSKGLRHYFDGSHIQD	SseK2 + YM155	100 s (23 C)	4.34	0.022132	5.87
SseK2	304	320	WLSKGLRHYFDGSHIQD	SseK2 + YM155	1,000 s (23 C)	5.38	0.062179	5.85
SseK2	304	320	WLSKGLRHYFDGSHIQD	SseK2 + YM155	10,000 s (23 C)	6.44	0.089448	5.84
SseK2	304	320	WLSKGLRHYFDGSHIQD	SseK2 + YM155	12 h (28 C)	7.25	0.179387	5.84
SseK2	304	320	WLSKGLRHYFDGSHIQD	SseK2 alone	10 s (23 C)	3.19	0.118262	5.90
SseK2	304	320	WLSKGLRHYFDGSHIQD	SseK2 alone	100 s (23 C)	4.49	0.049148	5.89
SseK2	304	320	WLSKGLRHYFDGSHIQD	SseK2 alone	1,000 s (23 C)	5.38	0.113874	5.89
SseK2	304	320	WLSKGLRHYFDGSHIQD	SseK2 alone	10,000 s (23 C)	6.51	0.097404	5.88
SseK2	304	320	WLSKGLRHYFDGSHIQD	SseK2 alone	12 h (28 C)	7.45	0.075352	5.87
SseK2	305	320	LSKGLRHYFDGSHIQD	SseK2 + YM155	10 s (23 C)	3.12	0.026656	5.01
SseK2	305	320	LSKGLRHYFDGSHIQD	SseK2 + YM155	100 s (23 C)	4.13	0.041808	4.99

SseK2	305	320	LSKGLRHYFDGSHIQD	SseK2 + YM155	1,000 s (23 C)	5.03	0.033988	4.99
SseK2	305	320	LSKGLRHYFDGSHIQD	SseK2 + YM155	10,000 s (23 C)	6.07	0.064645	4.99
SseK2	305	320	LSKGLRHYFDGSHIQD	SseK2 + YM155	12 h (28 C)	6.89	0.074639	4.98
SseK2	305	320	LSKGLRHYFDGSHIQD	SseK2 alone	10 s (23 C)	3.18	0.101769	5.03
SseK2	305	320	LSKGLRHYFDGSHIQD	SseK2 alone	100 s (23 C)	4.20	0.047986	5.01
SseK2	305	320	LSKGLRHYFDGSHIQD	SseK2 alone	1,000 s (23 C)	5.14	0.081815	5.01
SseK2	305	320	LSKGLRHYFDGSHIQD	SseK2 alone	10,000 s (23 C)	6.20	0.05194	5.01
SseK2	305	320	LSKGLRHYFDGSHIQD	SseK2 alone	12 h (28 C)	7.10	0.105145	5.00
SseK2	305	323	LSKGLRHYFDGSHIQDYDA	SseK2 + YM155	10 s (23 C)	4.05	0.237404	5.44
SseK2	305	323	LSKGLRHYFDGSHIQDYDA	SseK2 + YM155	100 s (23 C)	5.30	0.026038	5.42
SseK2	305	323	LSKGLRHYFDGSHIQDYDA	SseK2 + YM155	1,000 s (23 C)	6.45	0.049391	5.42
SseK2	305	323	LSKGLRHYFDGSHIQDYDA	SseK2 + YM155	10,000 s (23 C)	7.31	0.152422	5.42
SseK2	305	323	LSKGLRHYFDGSHIQDYDA	SseK2 + YM155	12 h (28 C)	8.09	0.078117	5.41
SseK2	305	323	LSKGLRHYFDGSHIQDYDA	SseK2 alone	10 s (23 C)	4.01	0.108975	5.46
SseK2	305	323	LSKGLRHYFDGSHIQDYDA	SseK2 alone	100 s (23 C)	5.39	0.106641	5.45
SseK2	305	323	LSKGLRHYFDGSHIQDYDA	SseK2 alone	1,000 s (23 C)	6.46	0.063843	5.45
SseK2	305	323	LSKGLRHYFDGSHIQDYDA	SseK2 alone	10,000 s (23 C)	7.27	0.0695	5.44
SseK2	305	323	LSKGLRHYFDGSHIQDYDA	SseK2 alone	12 h (28 C)	8.36	0.085501	5.43
SseK2	306	320	SKGLRHYFDGSHIQD	SseK2 + YM155	10 s (23 C)	2.50	0.13729	5.02
SseK2	306	320	SKGLRHYFDGSHIQD	SseK2 + YM155	100 s (23 C)	3.17	0.036692	5.00
SseK2	306	320	SKGLRHYFDGSHIQD	SseK2 + YM155	1,000 s (23 C)	4.00	0.061097	4.99
SseK2	306	320	SKGLRHYFDGSHIQD	SseK2 + YM155	10,000 s (23 C)	4.83	0.060037	4.99
SseK2	306	320	SKGLRHYFDGSHIQD	SseK2 + YM155	12 h (28 C)	5.41	0.106677	4.99
SseK2	306	320	SKGLRHYFDGSHIQD	SseK2 alone	10 s (23 C)	2.45	0.027124	5.04
SseK2	306	320	SKGLRHYFDGSHIQD	SseK2 alone	100 s (23 C)	3.21	0.022028	5.02
SseK2	306	320	SKGLRHYFDGSHIQD	SseK2 alone	1,000 s (23 C)	4.03	0.103668	5.02
SseK2	306	320	SKGLRHYFDGSHIQD	SseK2 alone	10,000 s (23 C)	4.87	0.11038	5.02
SseK2	306	320	SKGLRHYFDGSHIQD	SseK2 alone	12 h (28 C)	5.56	0.021765	5.01

SseK2	327	337	FIEFKHENIIM	SseK2 + YM155	10 s (23 C)	1.92	0.010312	6.76
SseK2	327	337	FIEFKHENIIM	SseK2 + YM155	100 s (23 C)	2.88	0.026541	6.73
SseK2	327	337	FIEFKHENIIM	SseK2 + YM155	1,000 s (23 C)	3.53	0.022092	6.74
SseK2	327	337	FIEFKHENIIM	SseK2 + YM155	10,000 s (23 C)	4.03	0.025843	6.74
SseK2	327	337	FIEFKHENIIM	SseK2 + YM155	12 h (28 C)	4.75	0.083999	6.74
SseK2	327	337	FIEFKHENIIM	SseK2 alone	10 s (23 C)	2.01	0.057021	6.80
SseK2	327	337	FIEFKHENIIM	SseK2 alone	100 s (23 C)	2.96	0.073086	6.79
SseK2	327	337	FIEFKHENIIM	SseK2 alone	1,000 s (23 C)	3.60	0.055403	6.80
SseK2	327	337	FIEFKHENIIM	SseK2 alone	10,000 s (23 C)	4.14	0.053933	6.80
SseK2	327	337	FIEFKHENIIM	SseK2 alone	12 h (28 C)	4.78	0.112442	6.79
SseK2	328	348	IEFKHENIIMNTSSLTASSWR	SseK2 + YM155	10 s (23 C)	9.68	0.185363	6.53
SseK2	328	348	IEFKHENIIMNTSSLTASSWR	SseK2 + YM155	100 s (23 C)	10.71	0.097587	6.51
SseK2	328	348	IEFKHENIIMNTSSLTASSWR	SseK2 + YM155	1,000 s (23 C)	11.47	0.066422	6.50
SseK2	328	348	IEFKHENIIMNTSSLTASSWR	SseK2 + YM155	10,000 s (23 C)	11.73	0.052325	6.49
SseK2	328	348	IEFKHENIIMNTSSLTASSWR	SseK2 + YM155	12 h (28 C)	12.29	0.046397	6.50
SseK2	328	348	IEFKHENIIMNTSSLTASSWR	SseK2 alone	10 s (23 C)	9.71	0.096233	6.58
SseK2	328	348	IEFKHENIIMNTSSLTASSWR	SseK2 alone	100 s (23 C)	10.80	0.086703	6.57
SseK2	328	348	IEFKHENIIMNTSSLTASSWR	SseK2 alone	1,000 s (23 C)	11.55	0.163341	6.57
SseK2	328	348	IEFKHENIIMNTSSLTASSWR	SseK2 alone	10,000 s (23 C)	11.88	0.194312	6.57
SseK2	328	348	IEFKHENIIMNTSSLTASSWR	SseK2 alone	12 h (28 C)	12.35	0.179742	6.57
SseK2	329	337	EFKHENIIM	SseK2 + YM155	10 s (23 C)	1.89	0.037812	6.13
SseK2	329	337	EFKHENIIM	SseK2 + YM155	100 s (23 C)	2.85	0.019979	6.11
SseK2	329	337	EFKHENIIM	SseK2 + YM155	1,000 s (23 C)	3.36	0.020748	6.11
SseK2	329	337	EFKHENIIM	SseK2 + YM155	10,000 s (23 C)	3.64	0.064769	6.11
SseK2	329	337	EFKHENIIM	SseK2 + YM155	12 h (28 C)	3.94	0.014086	6.11
SseK2	329	337	EFKHENIIM	SseK2 alone	10 s (23 C)	1.92	0.023158	6.21
SseK2	329	337	EFKHENIIM	SseK2 alone	100 s (23 C)	2.74	0.073778	6.21
SseK2	329	337	EFKHENIIM	SseK2 alone	1,000 s (23 C)	3.33	0.096604	6.22

SseK2	329	337	EFKHENIIM	SseK2 alone	10,000 s (23 C)	3.66	0.049812	6.22
SseK2	329	337	EFKHENIIM	SseK2 alone	12 h (28 C)	3.86	0.062777	6.22
SseK2	331	337	KHENIIM	SseK2 + YM155	10 s (23 C)	0.36	0.016006	4.84
SseK2	331	337	KHENIIM	SseK2 + YM155	100 s (23 C)	0.62	0.006839	4.81
SseK2	331	337	KHENIIM	SseK2 + YM155	1,000 s (23 C)	0.69	0.046584	4.81
SseK2	331	337	KHENIIM	SseK2 + YM155	10,000 s (23 C)	0.57	0.032906	4.81
SseK2	331	337	KHENIIM	SseK2 + YM155	12 h (28 C)	0.69	0.013907	4.81
SseK2	331	337	KHENIIM	SseK2 alone	10 s (23 C)	0.35	0.017055	4.83
SseK2	331	337	KHENIIM	SseK2 alone	100 s (23 C)	0.64	0.019762	4.81
SseK2	331	337	KHENIIM	SseK2 alone	1,000 s (23 C)	0.72	0.041965	4.81
SseK2	331	337	KHENIIM	SseK2 alone	10,000 s (23 C)	0.58	0.020881	4.82
SseK2	331	337	KHENIIM	SseK2 alone	12 h (28 C)	0.59	0.016523	4.81
SseK2	338	342	NTSSL	SseK2 + YM155	10 s (23 C)	1.81	0.012463	4.05
SseK2	338	342	NTSSL	SseK2 + YM155	100 s (23 C)	1.82	0.008785	4.02
SseK2	338	342	NTSSL	SseK2 + YM155	1,000 s (23 C)	1.81	0.018759	4.02
SseK2	338	342	NTSSL	SseK2 + YM155	10,000 s (23 C)	1.81	0.024009	4.03
SseK2	338	342	NTSSL	SseK2 + YM155	12 h (28 C)	1.82	0.007456	4.02
SseK2	338	342	NTSSL	SseK2 alone	10 s (23 C)	1.85	0.02313	4.05
SseK2	338	342	NTSSL	SseK2 alone	100 s (23 C)	1.83	0.007965	4.03
SseK2	338	342	NTSSL	SseK2 alone	1,000 s (23 C)	1.85	0.047813	4.03
SseK2	338	342	NTSSL	SseK2 alone	10,000 s (23 C)	1.84	0.038277	4.03
SseK2	338	342	NTSSL	SseK2 alone	12 h (28 C)	1.78	0.010101	4.02
SseK2	338	348	NTSSLTASSWR	SseK2 + YM155	10 s (23 C)	5.80	0.001311	5.44
SseK2	338	348	NTSSLTASSWR	SseK2 + YM155	100 s (23 C)	5.92	0.032524	5.42
SseK2	338	348	NTSSLTASSWR	SseK2 + YM155	1,000 s (23 C)	5.99	0.047026	5.42
SseK2	338	348	NTSSLTASSWR	SseK2 + YM155	10,000 s (23 C)	5.94	0.023361	5.42
SseK2	338	348	NTSSLTASSWR	SseK2 + YM155	12 h (28 C)	6.00	0.09132	5.42
SseK2	338	348	NTSSLTASSWR	SseK2 alone	10 s (23 C)	5.94	0.064684	5.47

SseK2	338	348	NTSSLTASSWR	SseK2 alone	100 s (23 C)	6.12	0.015276	5.46
SseK2	338	348	NTSSLTASSWR	SseK2 alone	1,000 s (23 C)	6.18	0.063805	5.46
SseK2	338	348	NTSSLTASSWR	SseK2 alone	10,000 s (23 C)	6.18	0.075504	5.46
SseK2	338	348	NTSSLTASSWR	SseK2 alone	12 h (28 C)	6.24	0.023976	5.45
SseK2	343	348	TASSWR	SseK2 + YM155	10 s (23 C)	2.87	0.04015	4.12
SseK2	343	348	TASSWR	SseK2 + YM155	100 s (23 C)	3.11	0.010415	4.09
SseK2	343	348	TASSWR	SseK2 + YM155	1,000 s (23 C)	3.13	0.0042	4.09
SseK2	343	348	TASSWR	SseK2 + YM155	10,000 s (23 C)	3.07	0.040204	4.10
SseK2	343	348	TASSWR	SseK2 + YM155	12 h (28 C)	3.12	0.014093	4.09
SseK2	343	348	TASSWR	SseK2 alone	10 s (23 C)	2.90	0.031235	4.11
SseK2	343	348	TASSWR	SseK2 alone	100 s (23 C)	3.13	0.024035	4.09
SseK2	343	348	TASSWR	SseK2 alone	1,000 s (23 C)	3.17	0.030373	4.10
SseK2	343	348	TASSWR	SseK2 alone	10,000 s (23 C)	3.13	0.055206	4.10
SseK2	343	348	TASSWR	SseK2 alone	12 h (28 C)	3.08	0.037267	4.09

Table S8. Deuterium uptake values, uptake standard deviation (SD) and retention time (RT) of the individual SseK2 peptides whose HDX was followed at every time point and in both states (apo- and YM155 bound SseK2).

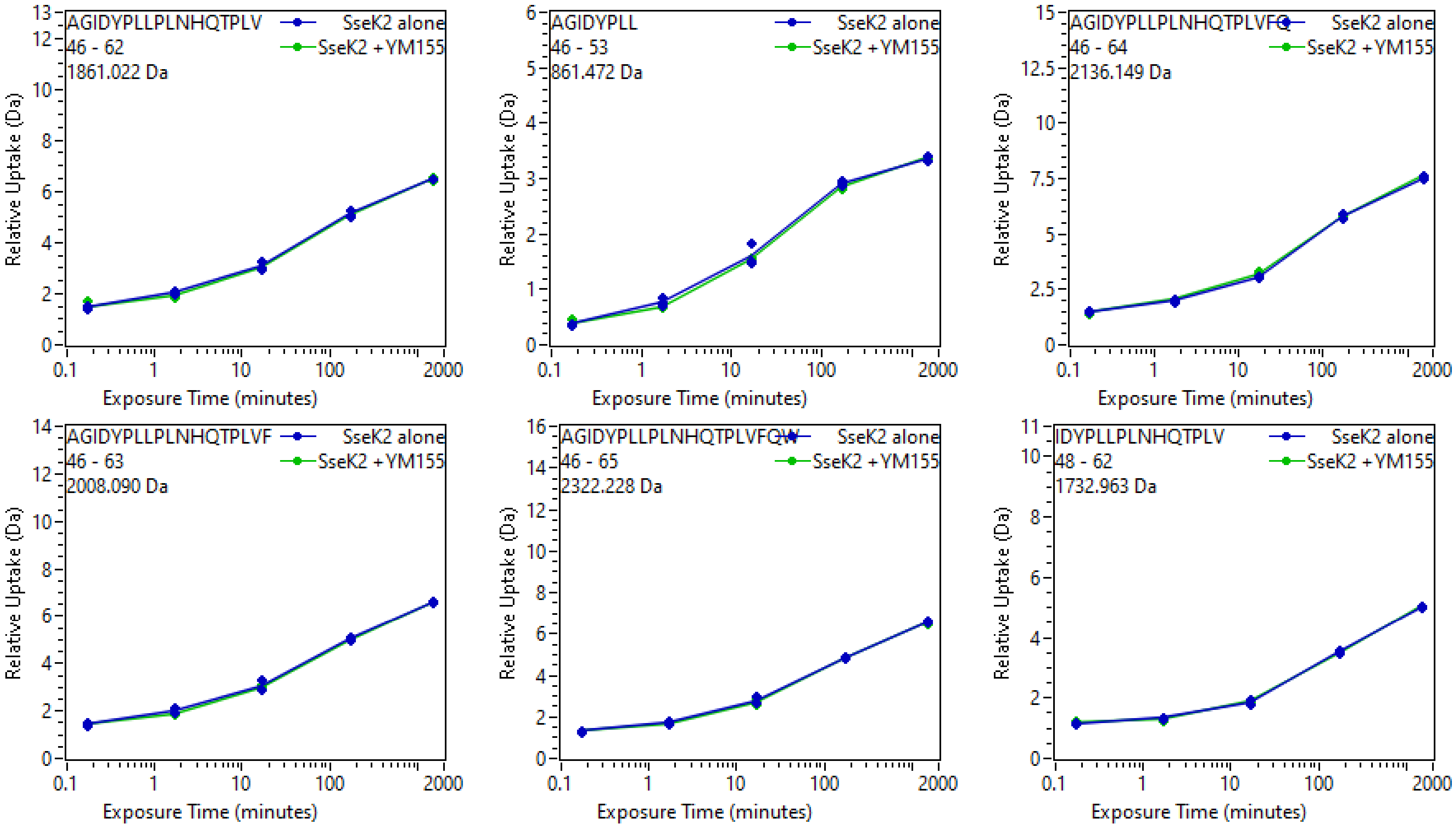
AGIDYPLLPLNHQTPLV
46 - 62
1861.022 Da
SseK2 alone
SseK2 +YM155
AGIDYPLL
46 - 53
861.472 Da
AGIDYPLLPLNHQTPLVFQ
46 - 64
2136.149 Da
AGIDYPLLPLNHQTPLVF
46 - 63
2008.090 Da
AGIDYPLLPLNHQTPLVFQW
46 - 65
2322.228 Da
IDYPLLPLNHQTPLV
48 - 62
1732.963 Da
Relative Uptake (Da)
Exposure Time (minutes)
0.1
1
10
100
2000

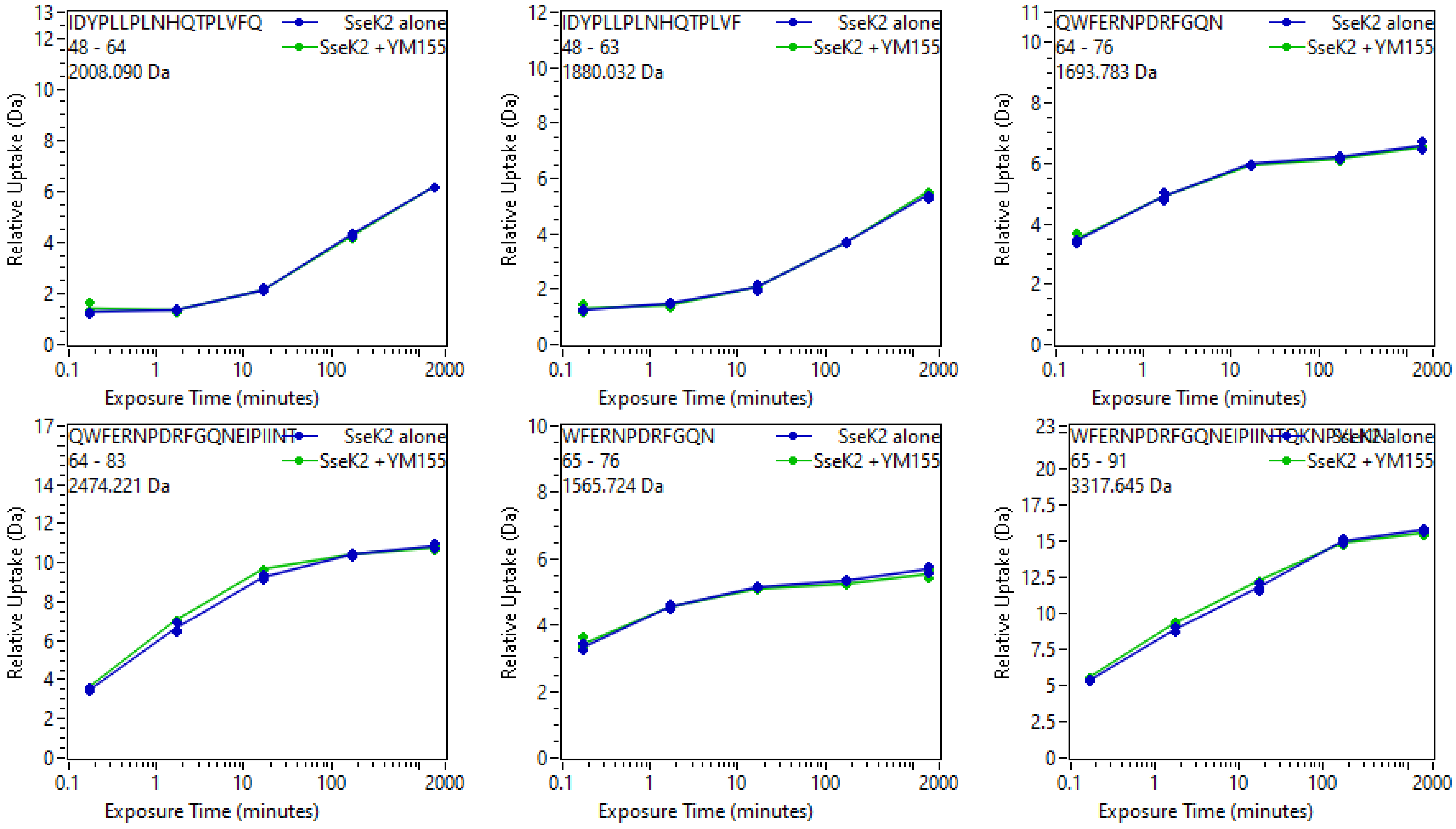
IDYPLLPLNHQTPLVFQ
48 - 64
2008.090 Da
IDYPLLPLNHQTPLVF
48 - 63
1880.032 Da
QWFERNPDRFGQN
64 - 76
1693.783 Da
QWFERNPDRFGQNEIPIINT
64 - 83
2474.221 Da
WFERNPDRFGQN
65 - 76
1565.724 Da
WFERNPDRFGQNEIPIINTQKNPSLKL
65 - 91
3317.645 Da
SseK2 alone
SseK2 +YM155
Relative Uptake (Da)
Exposure Time (minutes)

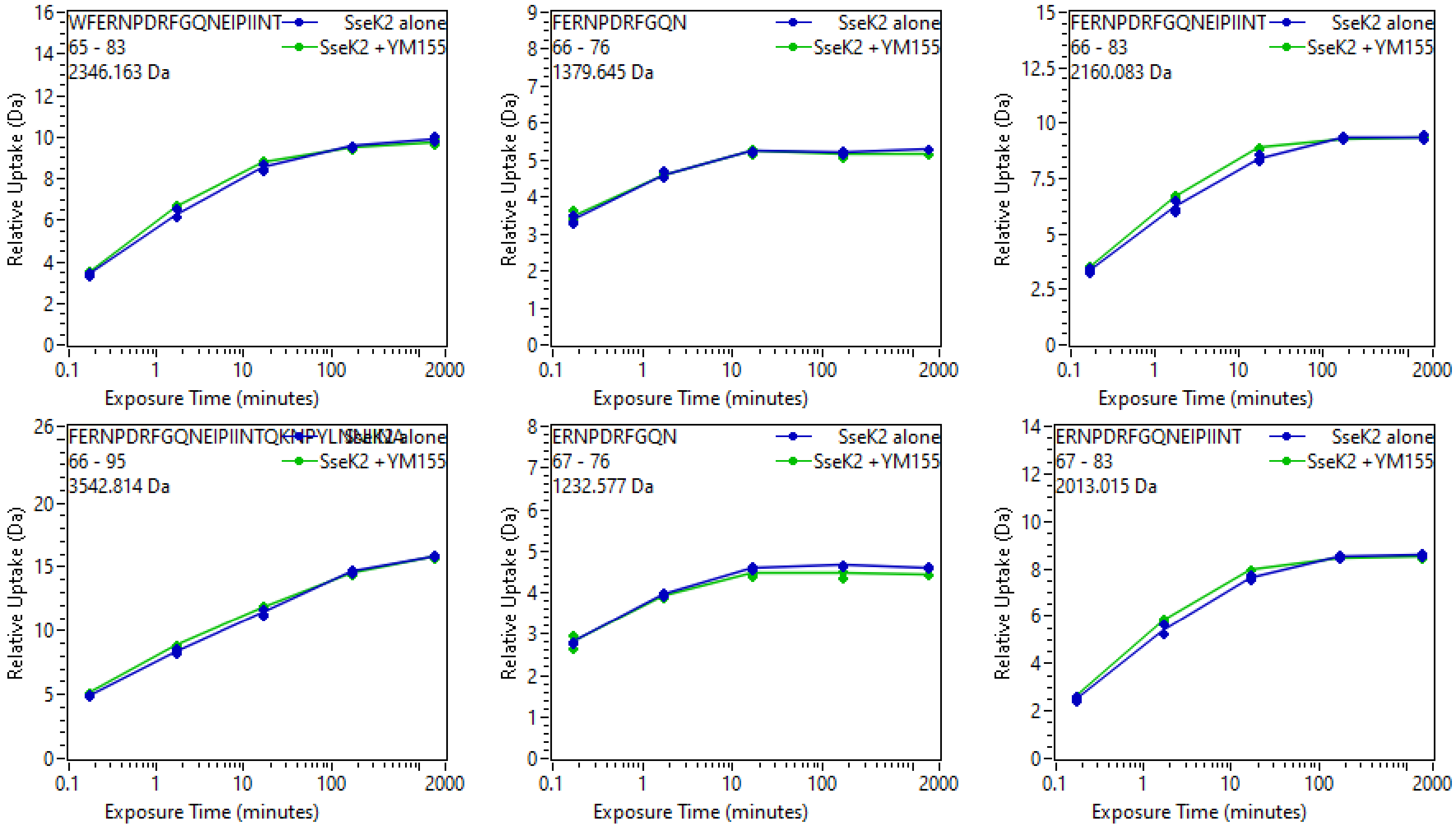
WFERNPDRFGQNEIPIINT
65 - 83
2346.163 Da
SseK2 alone
SseK2 +YM155
FERNPDRFGQN
66 - 76
1379.645 Da
SseK2 alone
SseK2 +YM155
FERNPDRFGQNEIPIINT
66 - 83
2160.083 Da
SseK2 alone
SseK2 +YM155
FERNPDRFGQNEIPIINTQK
66 - 95
3542.814 Da
SseK2 alone
SseK2 +YM155
ERNPDRFGQN
67 - 76
1232.577 Da
SseK2 alone
SseK2 +YM155
ERNPDRFGQNEIPIINT
67 - 83
2013.015 Da
SseK2 alone
SseK2 +YM155
Relative Uptake (Da)
Exposure Time (minutes)

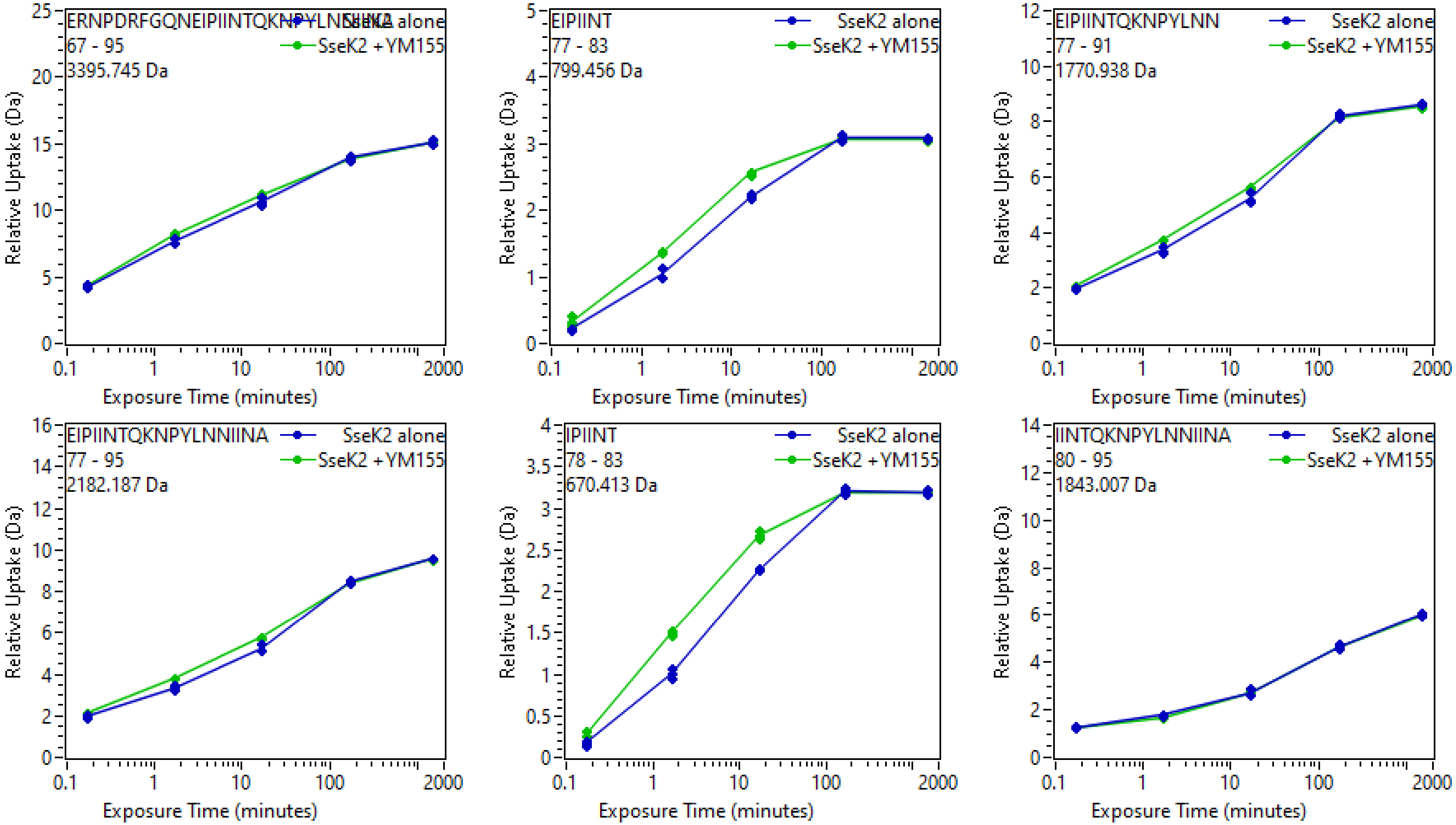
ERNPDRFGQNEIPIINTQKNPYLN
67 - 95
3395.745 Da
SseK2 alone
SseK2 +YM155
EIPIINT
77 - 83
799.456 Da
EIPIINTQKNPYLNN
77 - 91
1770.938 Da
EIPIINTQKNPYLNNIINA
77 - 95
2182.187 Da
IPIINT
78 - 83
670.413 Da
IINTQKNPYLNNIINA
80 - 95
1843.007 Da
Relative Uptake (Da)
Exposure Time (minutes)
0.1
1
10
100
2000

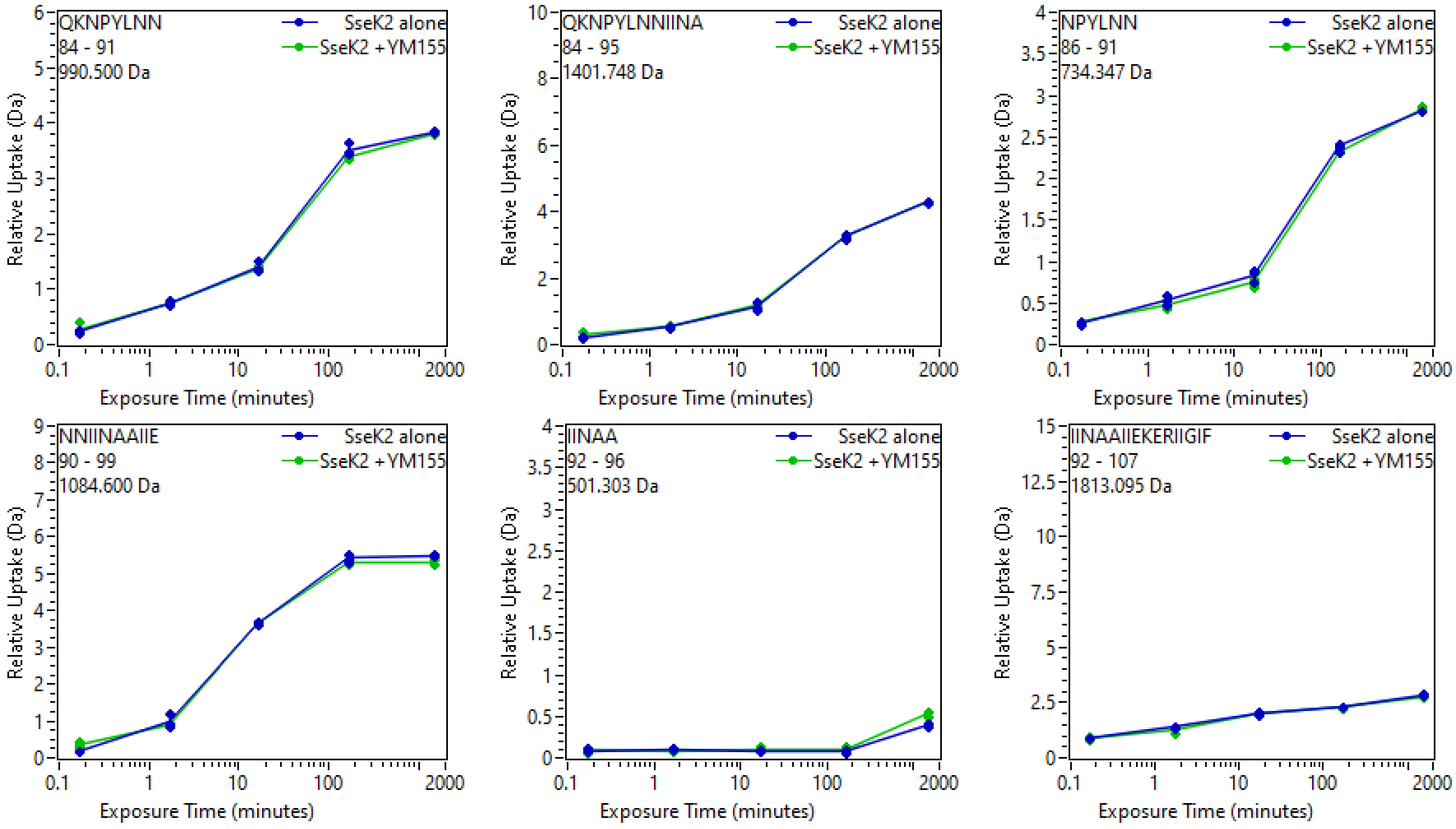

QKNPYLNN
84 - 91
990.500 Da
SseK2 alone
SseK2 +YM155
Relative Uptake (Da)
Exposure Time (minutes)
QKNPYLNNIINA
84 - 95
1401.748 Da
NPYLNN
86 - 91
734.347 Da
NNIINAAIIE
90 - 99
1084.600 Da
IINAA
92 - 96
501.303 Da
IINAAIIEKERIIGIF
92 - 107
1813.095 Da

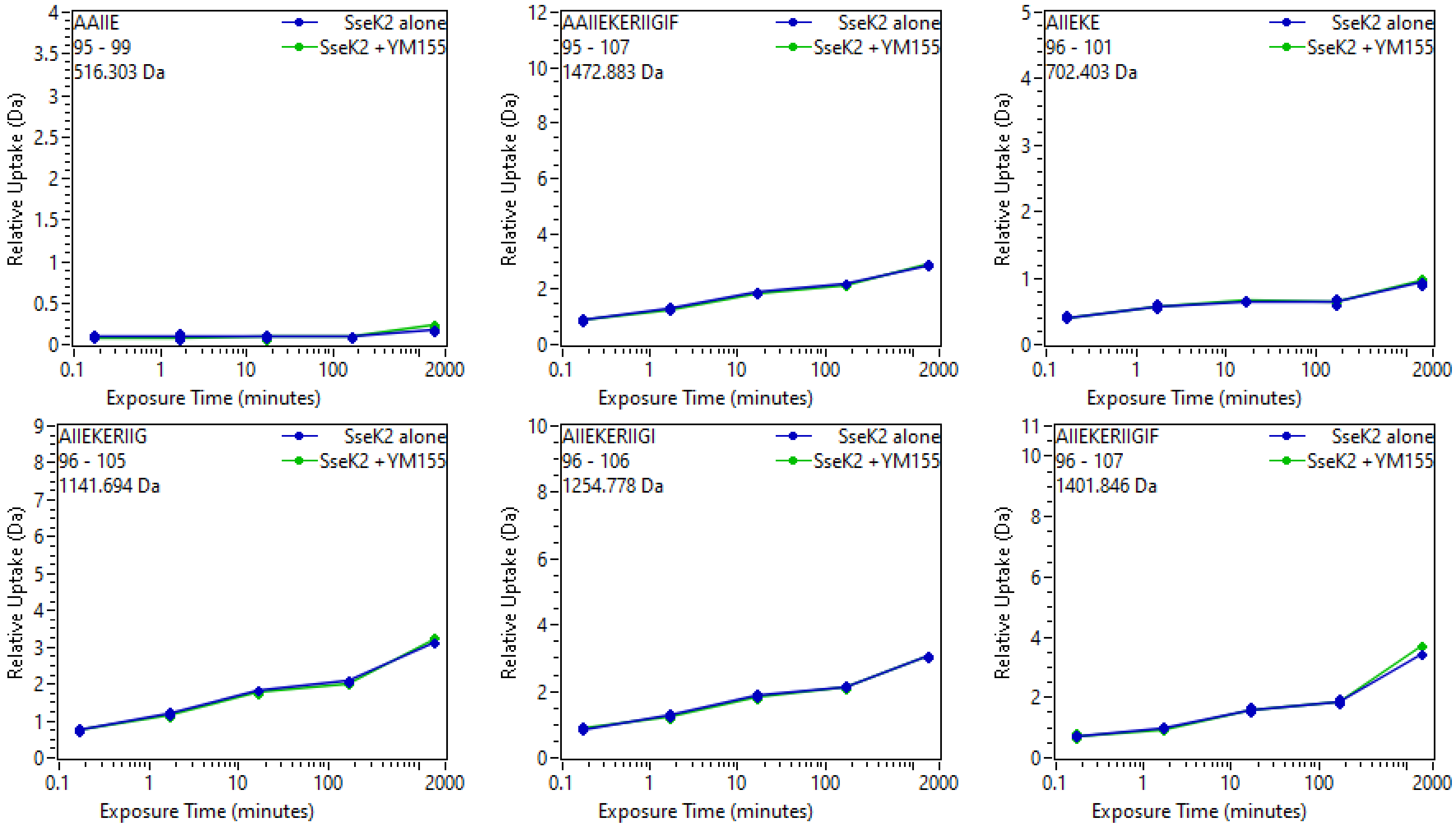
AAIIE
95 - 99
516.303 Da
AAIIEKERIIGIF
95 - 107
1472.883 Da
AIIEKE
96 - 101
702.403 Da
AIIEKERIIG
96 - 105
1141.694 Da
AIIEKERIIGI
96 - 106
1254.778 Da
AIIEKERIIGIF
96 - 107
1401.846 Da
SseK2 alone
SseK2 +YM155
Relative Uptake (Da)
Exposure Time (minutes)
0.1
1
10
100
2000

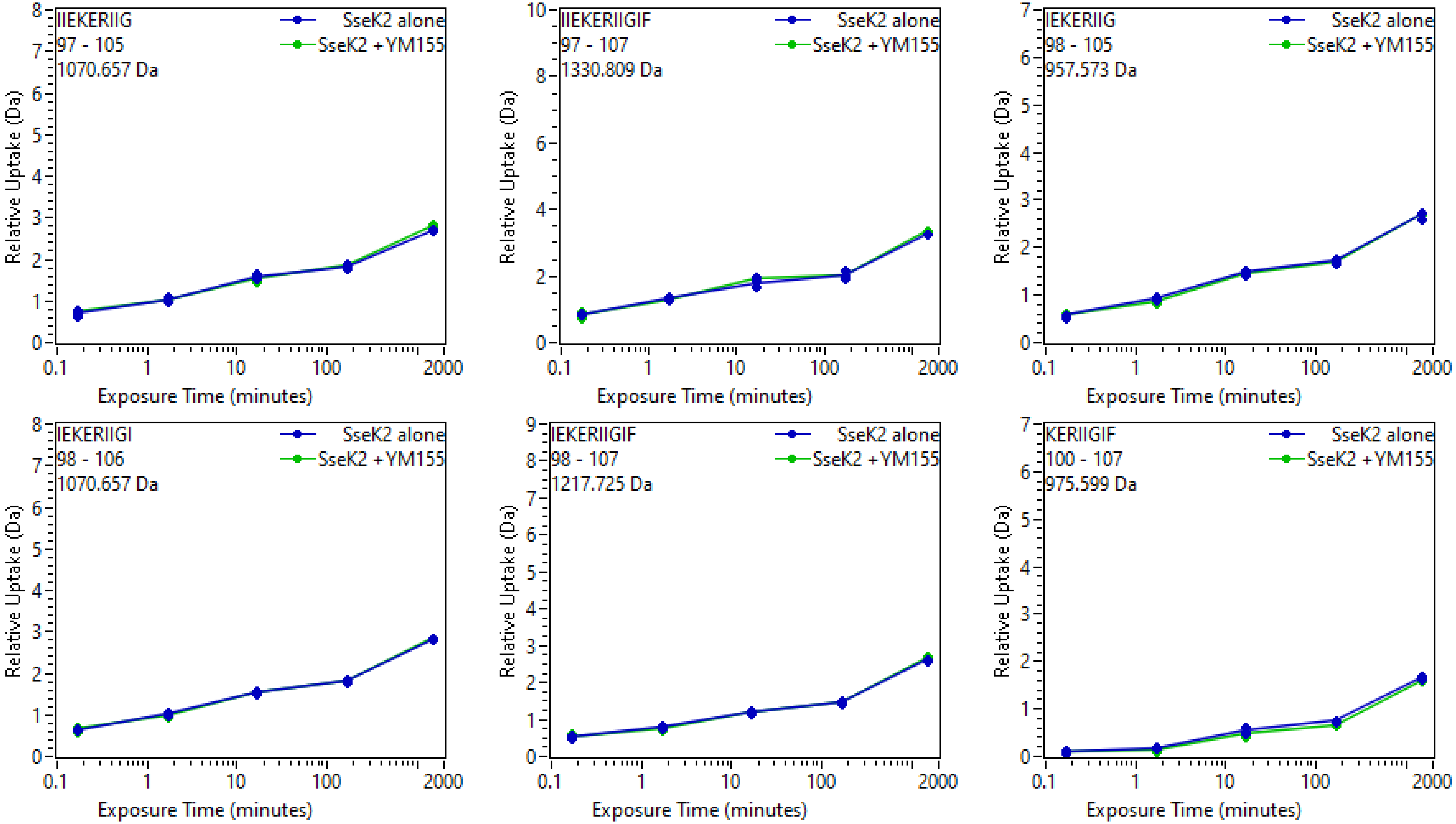
IIEKERIIG
97 - 105
1070.657 Da
SseK2 alone
SseK2 +YM155
Relative Uptake (Da)
Exposure Time (minutes)
IIEKERIIGIF
97 - 107
1330.809 Da
IEKERIIG
98 - 105
957.573 Da
IEKERIIGI
98 - 106
1070.657 Da
IEKERIIGIF
98 - 107
1217.725 Da
KERIIGIF
100 - 107
975.599 Da
0.1
1
10
100
2000

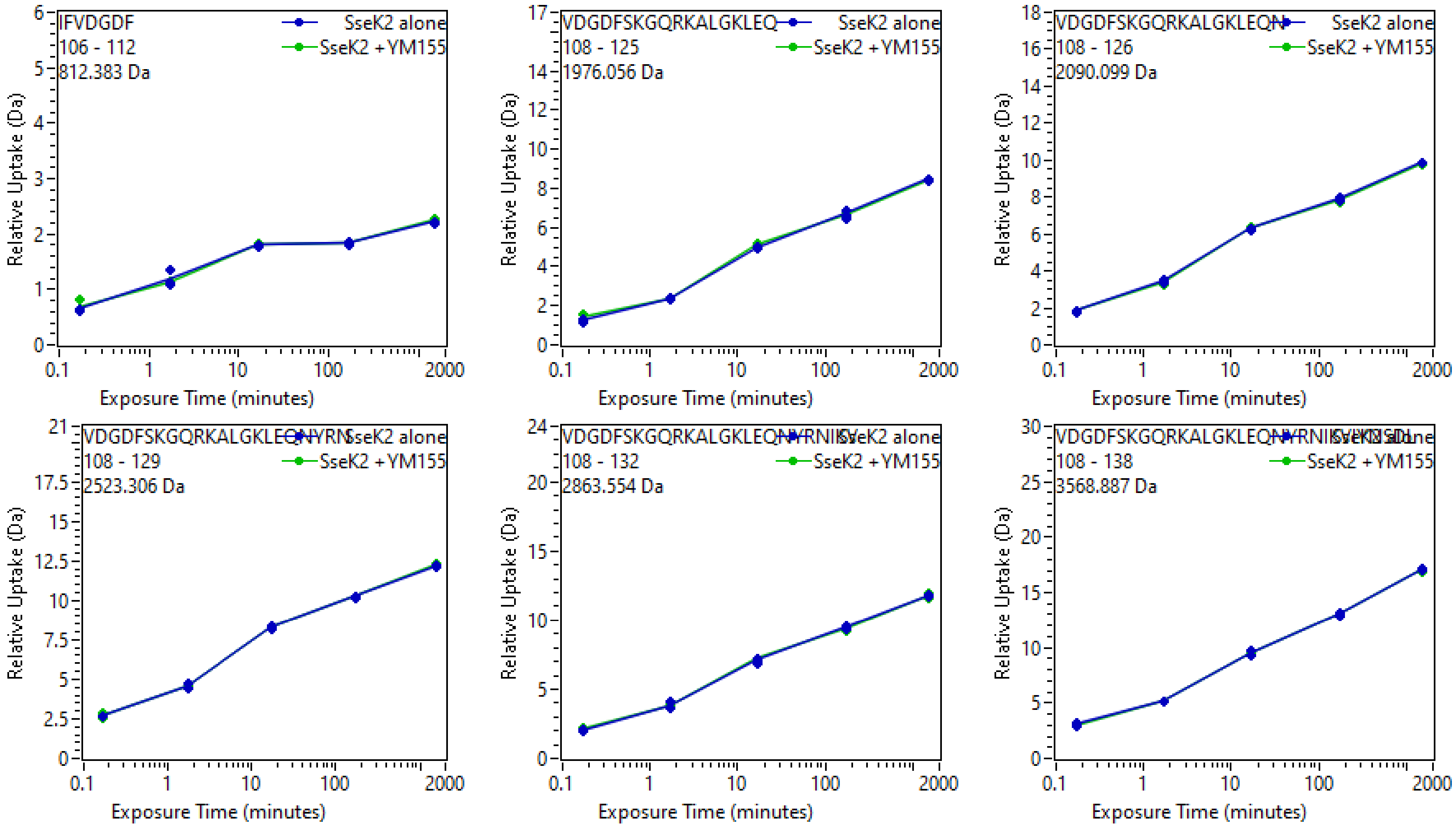
IFVDGDF
106 - 112
812.383 Da
SseK2 alone
SseK2 +YM155
VDGDFSKGQRKALGKLEQ
108 - 125
1976.056 Da
108 - 126
2090.099 Da
108 - 129
2523.306 Da
108 - 132
2863.554 Da
108 - 138
3568.887 Da
Relative Uptake (Da)
Exposure Time (minutes)

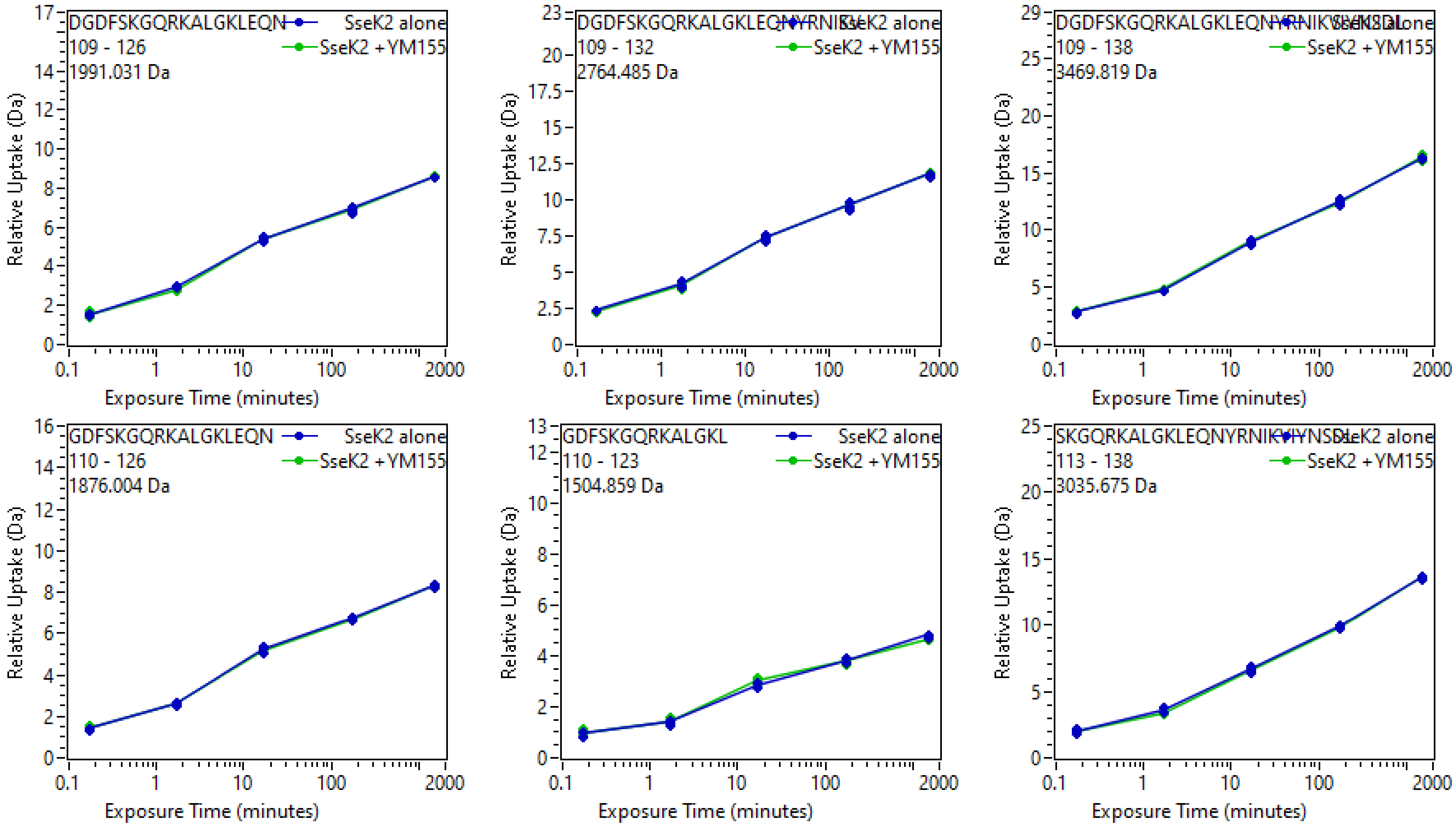
DGDFSKGQRKALGKLEQN
109 - 126
1991.031 Da
SseK2 alone
SseK2 +YM155
109 - 132
2764.485 Da
109 - 138
3469.819 Da
GDFSKGQRKALGKLEQN
110 - 126
1876.004 Da
GDFSKGQRKALGKL
110 - 123
1504.859 Da
113 - 138
3035.675 Da
Relative Uptake (Da)
Exposure Time (minutes)
0.1
1
10
100
2000

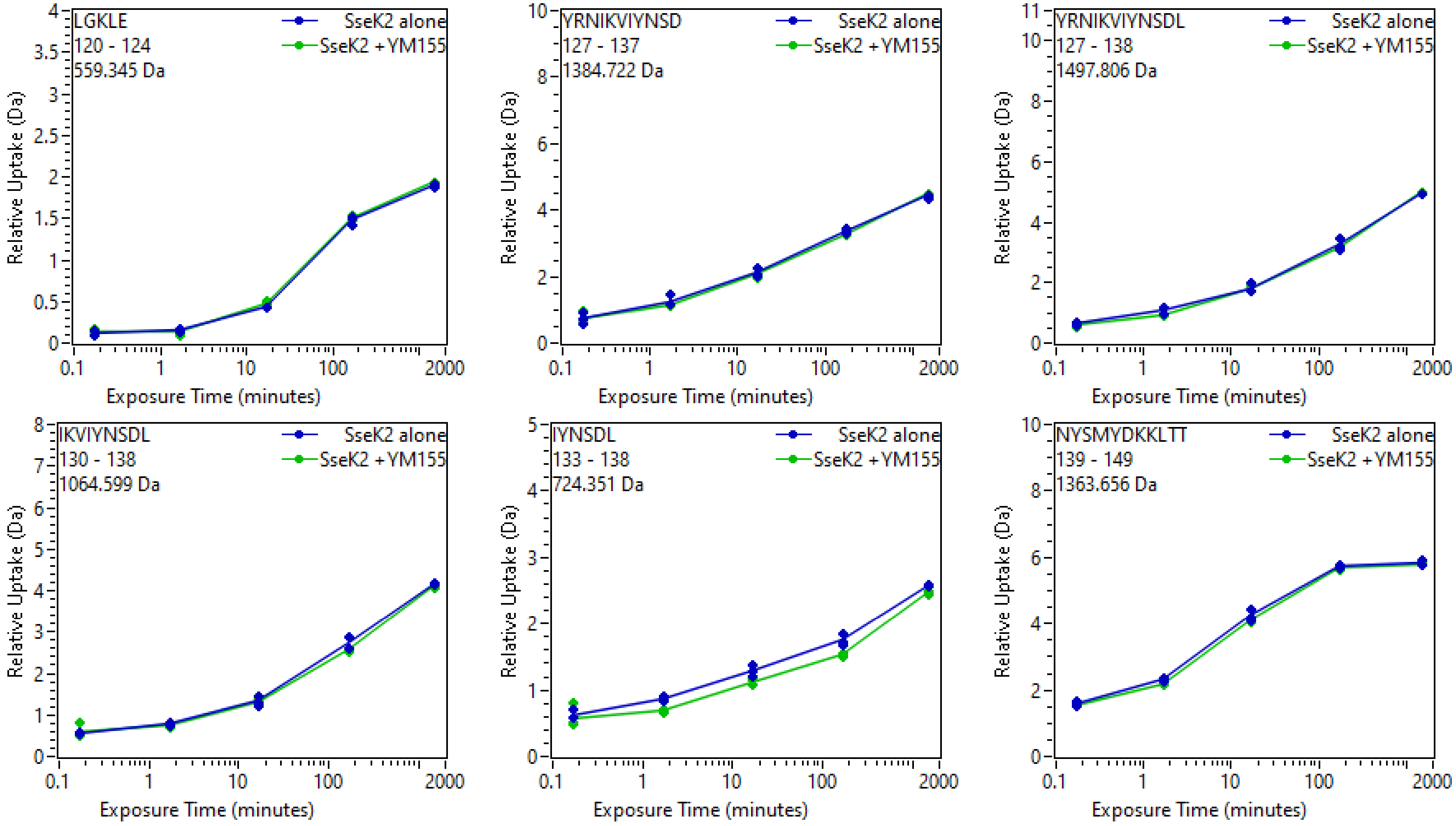
LGKLE
120 - 124
559.345 Da
YRNIKVIYNSD
127 - 137
1384.722 Da
YRNIKVIYNSDL
127 - 138
1497.806 Da
IKVIYNSDL
130 - 138
1064.599 Da
IYNSDL
133 - 138
724.351 Da
NYSMYDKKLTT
139 - 149
1363.656 Da
SseK2 alone
SseK2 +YM155
Relative Uptake (Da)
Exposure Time (minutes)

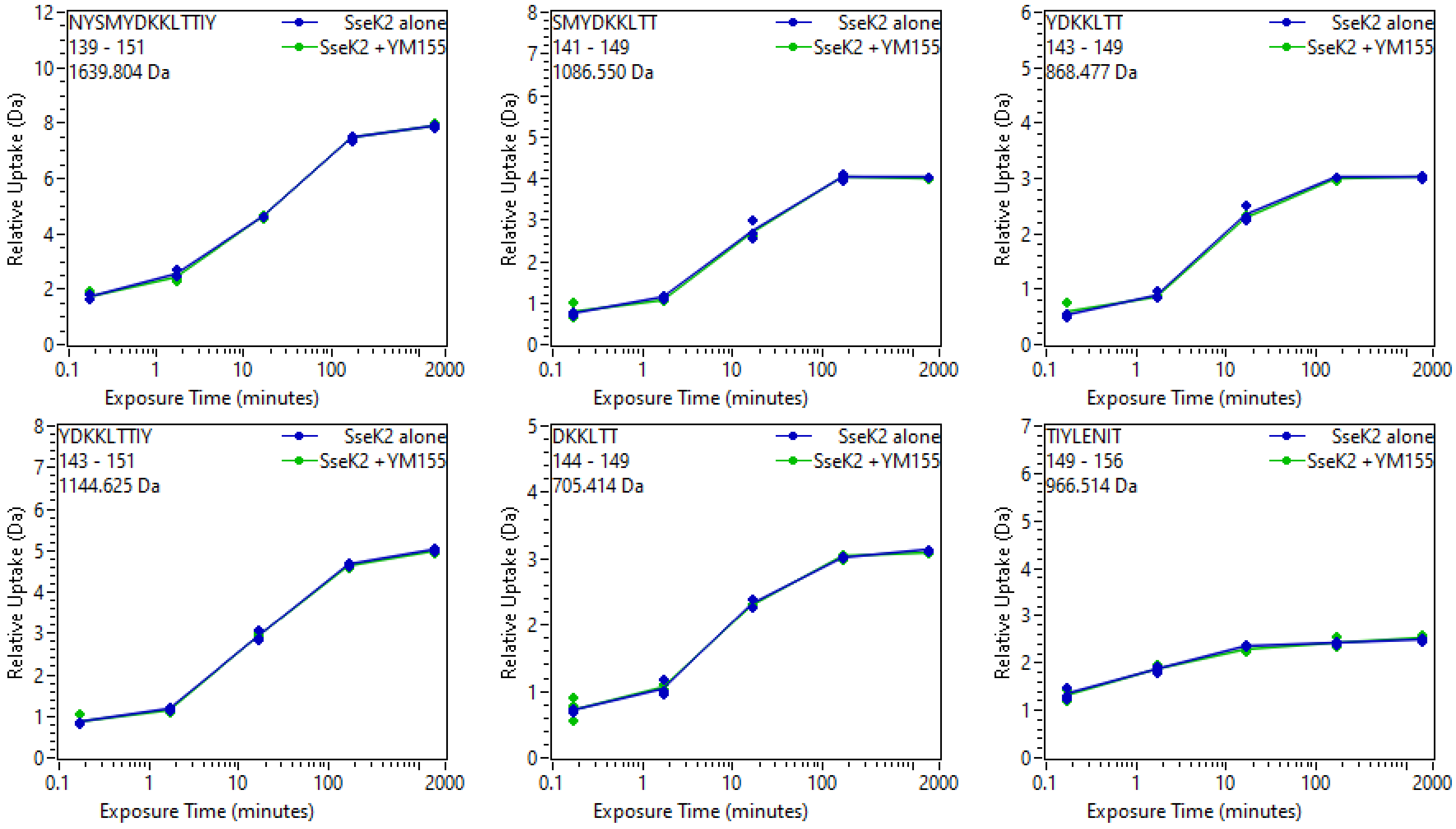
NYSMYDKKLTTIY
139 - 151
1639.804 Da
SMYDKKLTT
141 - 149
1086.550 Da
YDKKLTT
143 - 149
868.477 Da
YDKKLTTIY
143 - 151
1144.625 Da
DKKLTT
144 - 149
705.414 Da
TIYLENIT
149 - 156
966.514 Da
SseK2 alone
SseK2 +YM155
Relative Uptake (Da)
Exposure Time (minutes)
0.1
1
10
100
2000

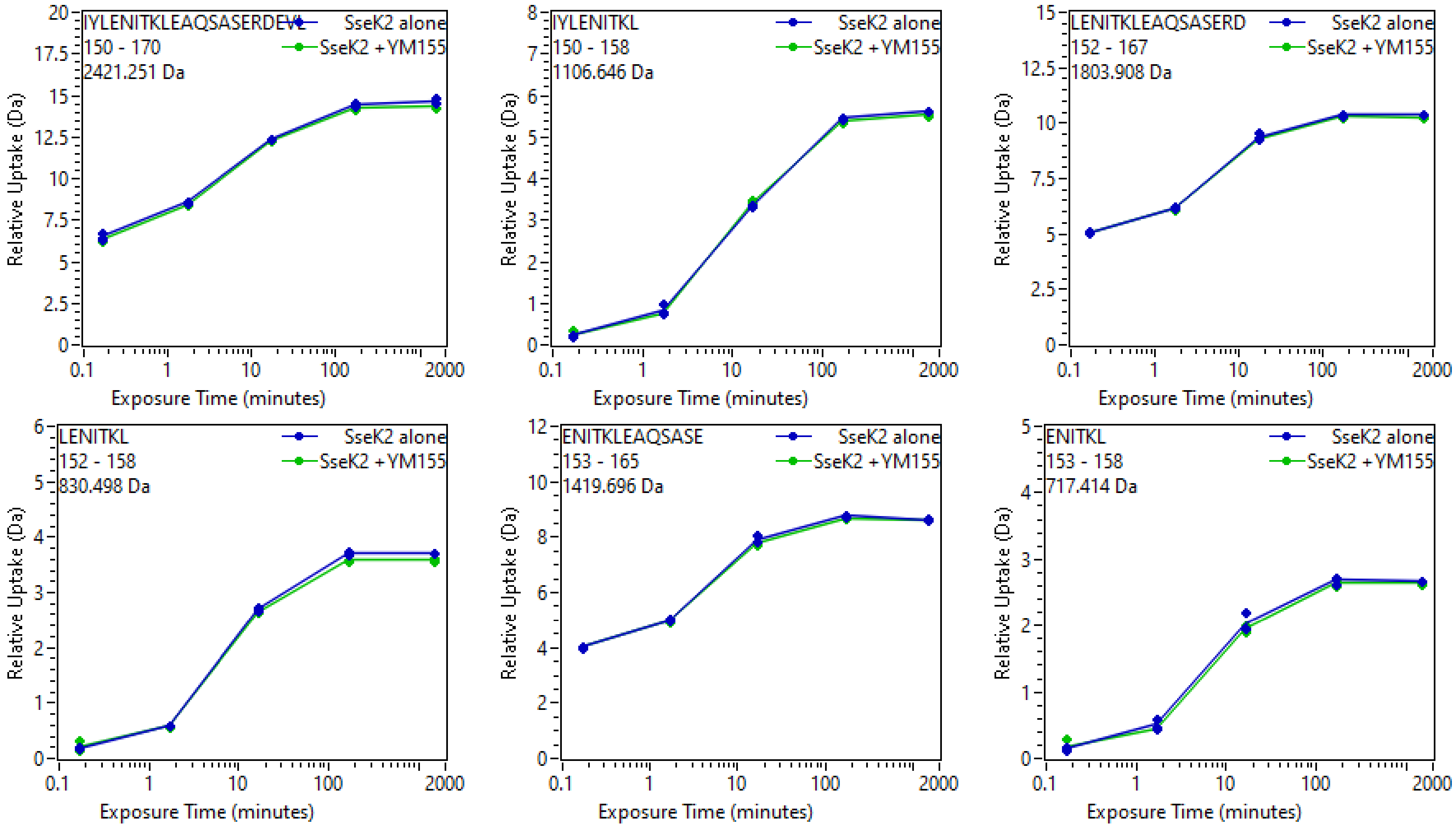
IYLENITKLEAQSASERDEVL
150 - 170
2421.251 Da
SseK2 alone
SseK2 +YM155
Relative Uptake (Da)
Exposure Time (minutes)
IYLENITKL
150 - 158
1106.646 Da
SseK2 alone
SseK2 +YM155
Relative Uptake (Da)
Exposure Time (minutes)
LENITKLEAQSASERD
152 - 167
1803.908 Da
SseK2 alone
SseK2 +YM155
Relative Uptake (Da)
Exposure Time (minutes)
LENITKL
152 - 158
830.498 Da
SseK2 alone
SseK2 +YM155
Relative Uptake (Da)
Exposure Time (minutes)
ENITKLEAQSASE
153 - 165
1419.696 Da
SseK2 alone
SseK2 +YM155
Relative Uptake (Da)
Exposure Time (minutes)
ENITKL
153 - 158
717.414 Da
SseK2 alone
SseK2 +YM155
Relative Uptake (Da)
Exposure Time (minutes)

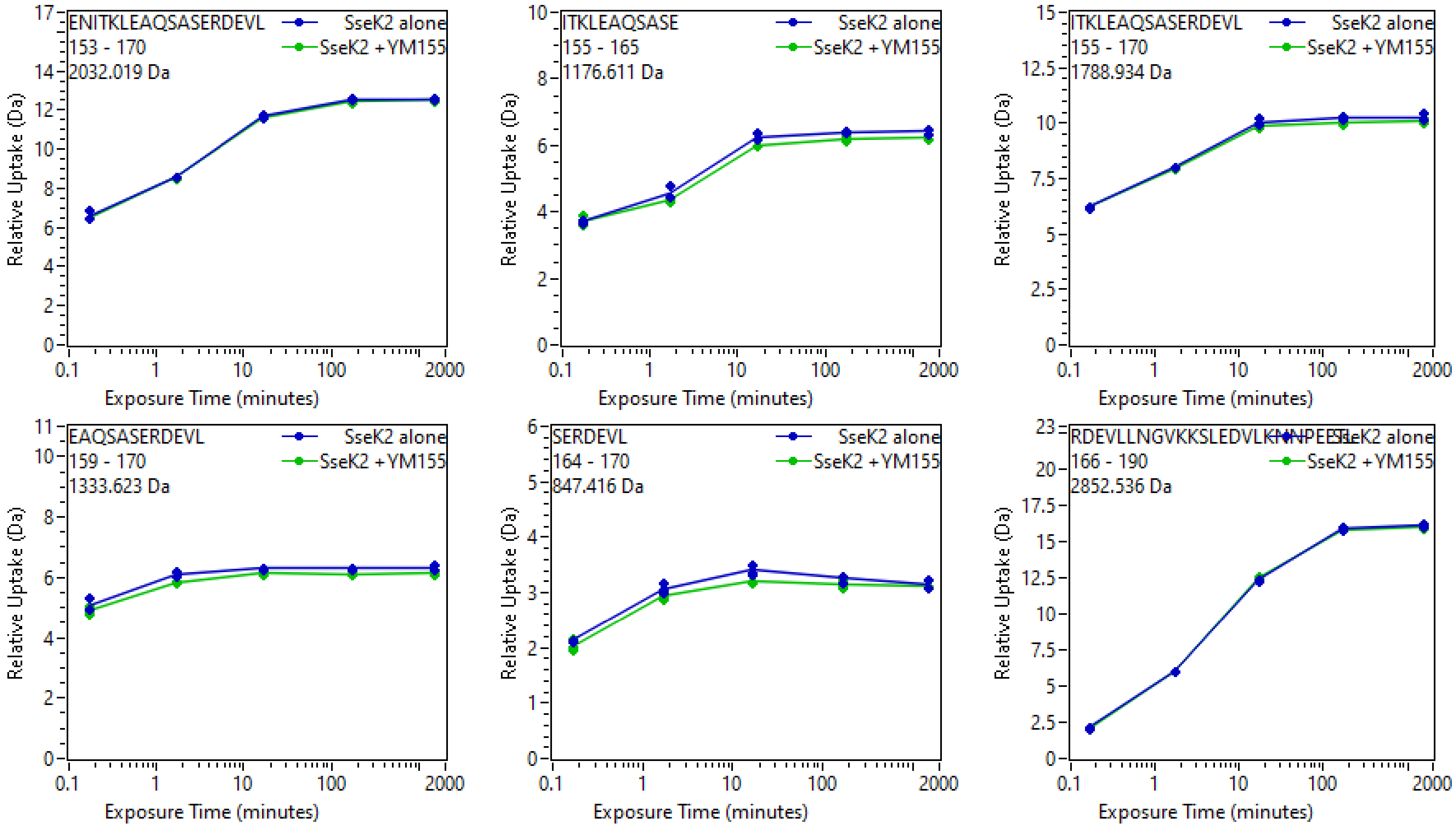
ENITKLEAQSASERDEVL
153 - 170
2032.019 Da
SseK2 alone
SseK2 +YM155
Relative Uptake (Da)
Exposure Time (minutes)
ITKLEAQSASE
155 - 165
1176.611 Da
ITKLEAQSASERDEVL
155 - 170
1788.934 Da
EAQSASERDEVL
159 - 170
1333.623 Da
SERDEVL
164 - 170
847.416 Da
RDEVLLNGVKKSLEDVLKMNPEEL
166 - 190
2852.536 Da

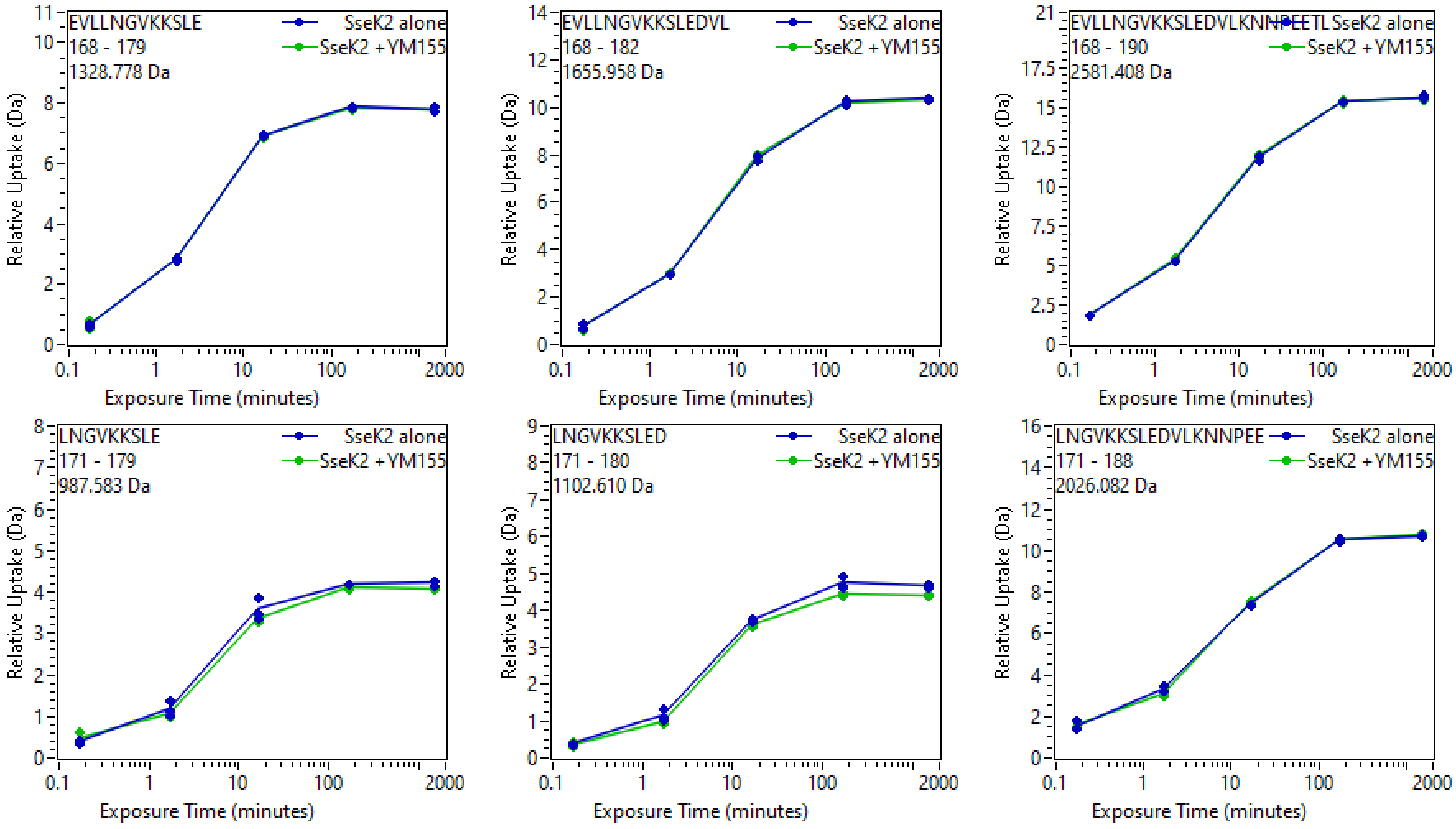
EVLLNGVKKSLE
168 - 179
1328.778 Da
SseK2 alone
SseK2 +YM155
Relative Uptake (Da)
Exposure Time (minutes)
EVLLNGVKKSLEDVL
168 - 182
1655.958 Da
EVLLNGVKKSLEDVLKNNPEETL
168 - 190
2581.408 Da
LNGVKKSLE
171 - 179
987.583 Da
LNGVKKSLED
171 - 180
1102.610 Da
LNGVKKSLEDVLKNNPEE
171 - 188
2026.082 Da

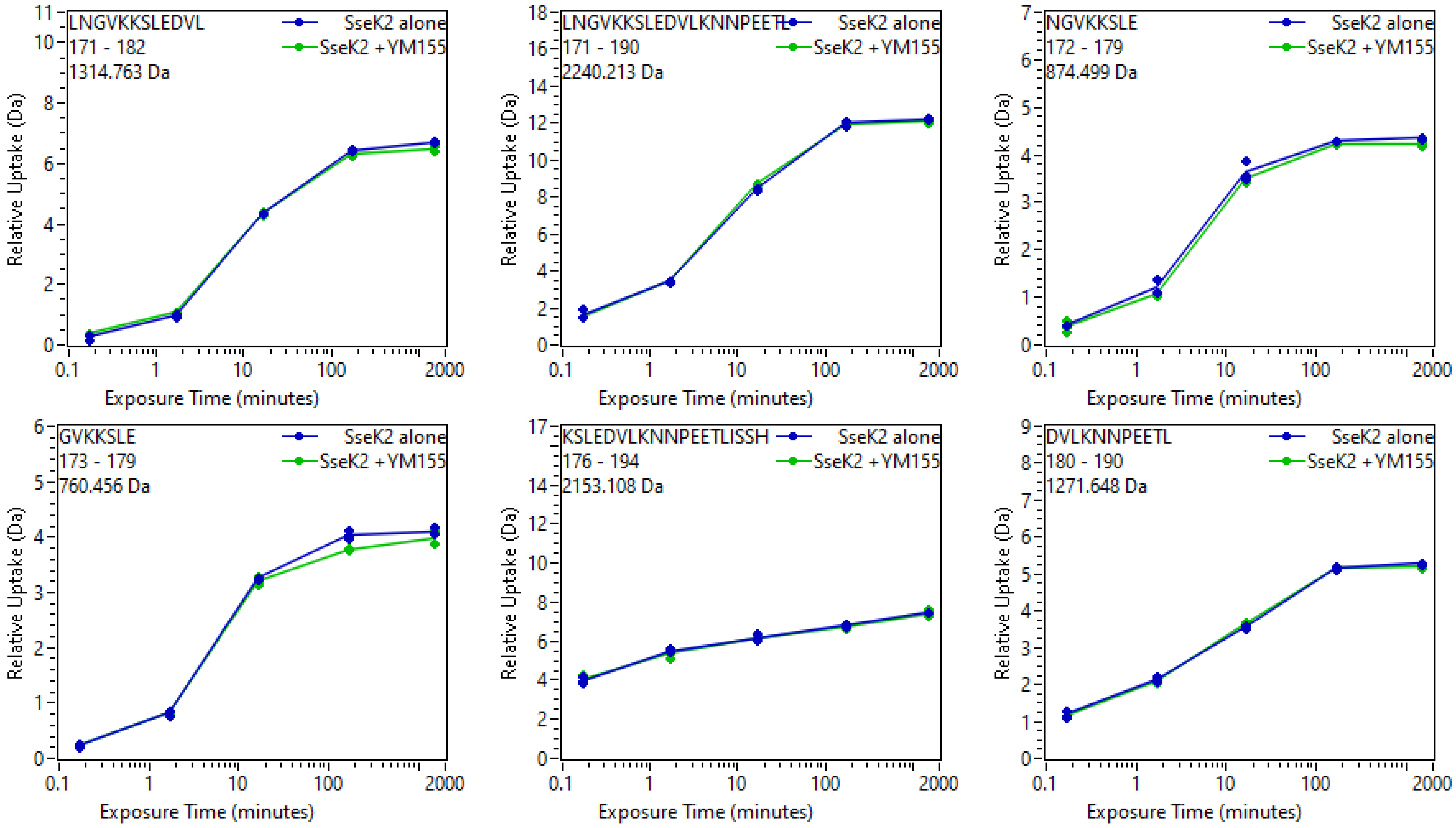
LNGVKKSLEDVL
171 - 182
1314.763 Da
SseK2 alone
SseK2 +YM155
Relative Uptake (Da)
Exposure Time (minutes)
LNGVKKSLEDVLKNNPEETL
171 - 190
2240.213 Da
NGVKKSLE
172 - 179
874.499 Da
GVKKSLE
173 - 179
760.456 Da
KSLEDVLKNNPEETLISSH
176 - 194
2153.108 Da
DVLKNNPEETL
180 - 190
1271.648 Da

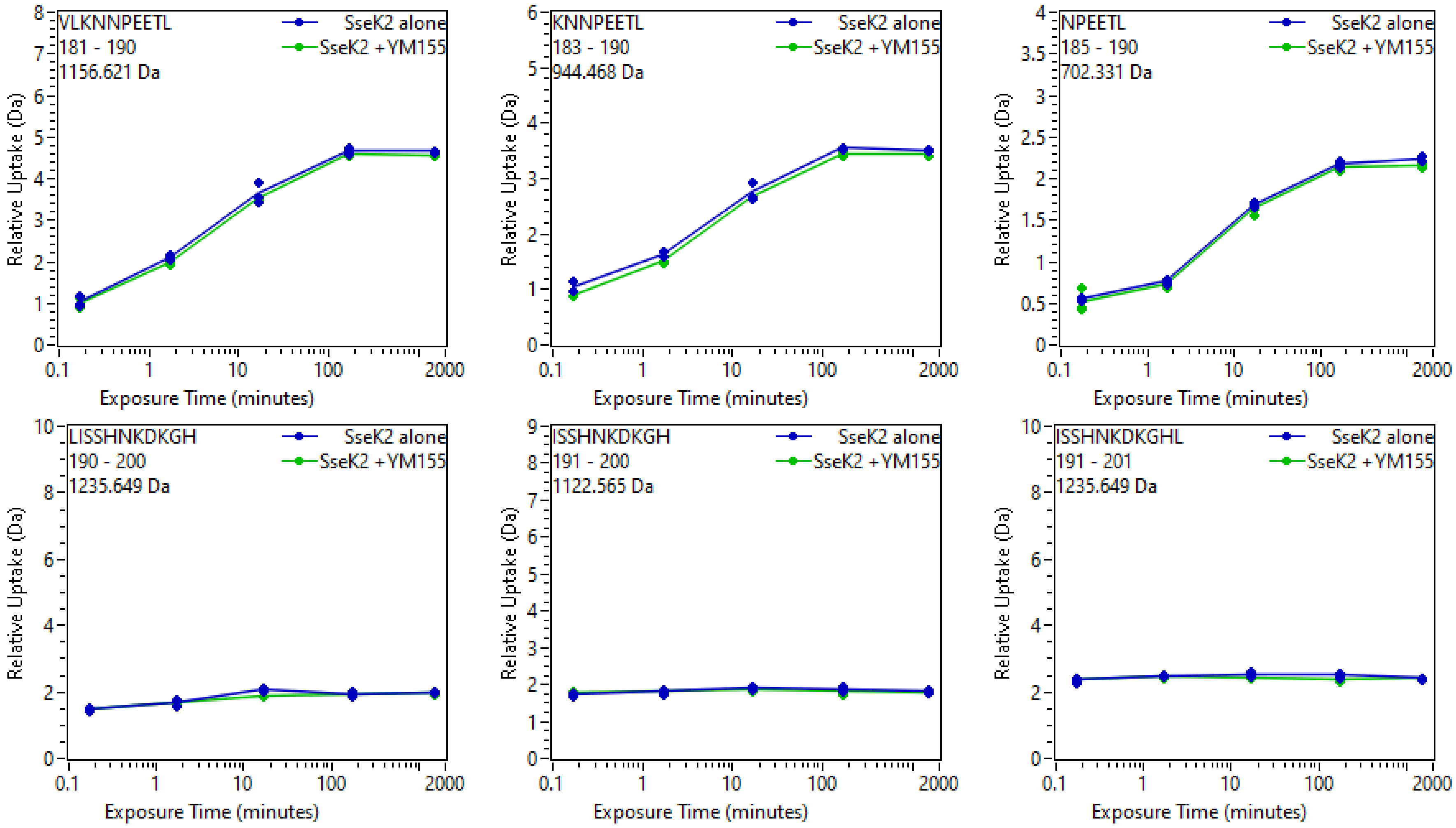

VLKNNPEETL
181 - 190
1156.621 Da
KNNPEETL
183 - 190
944.468 Da
NPEETL
185 - 190
702.331 Da
LISSHNKDKGH
190 - 200
1235.649 Da
ISSHNKDKGH
191 - 200
1122.565 Da
ISSHNKDKGHL
191 - 201
1235.649 Da
SseK2 alone
SseK2 +YM155
Relative Uptake (Da)
Exposure Time (minutes)
0.1
1
10
100
2000

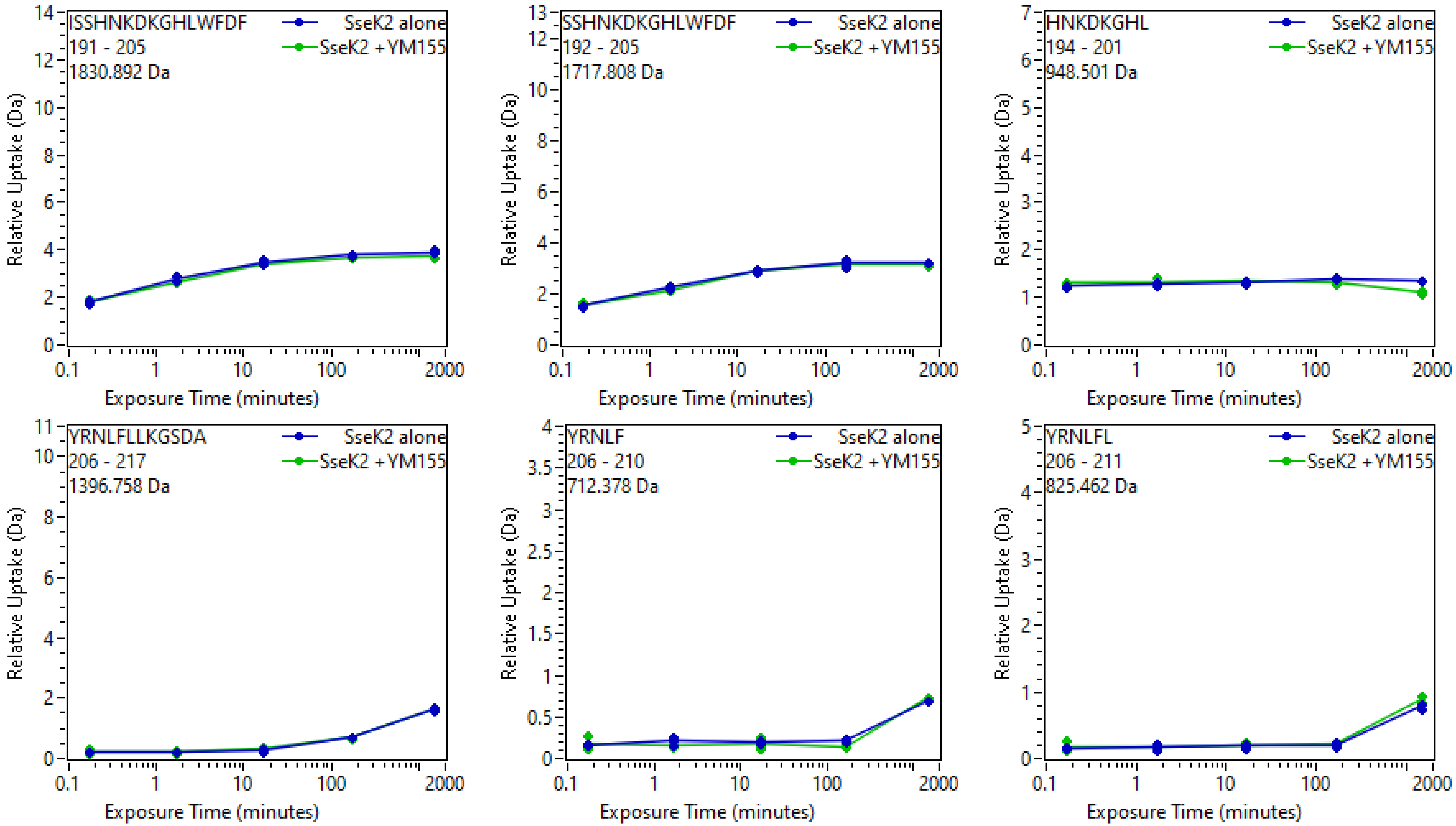
ISSHNKDKGHLWFDF
191 - 205
1830.892 Da
SSHNKDKGHLWFDF
192 - 205
1717.808 Da
HNKDKGHL
194 - 201
948.501 Da
YRNLFLLKGSDA
206 - 217
1396.758 Da
YRNLF
206 - 210
712.378 Da
YRNLFL
206 - 211
825.462 Da
SseK2 alone
SseK2 +YM155
Relative Uptake (Da)
Exposure Time (minutes)

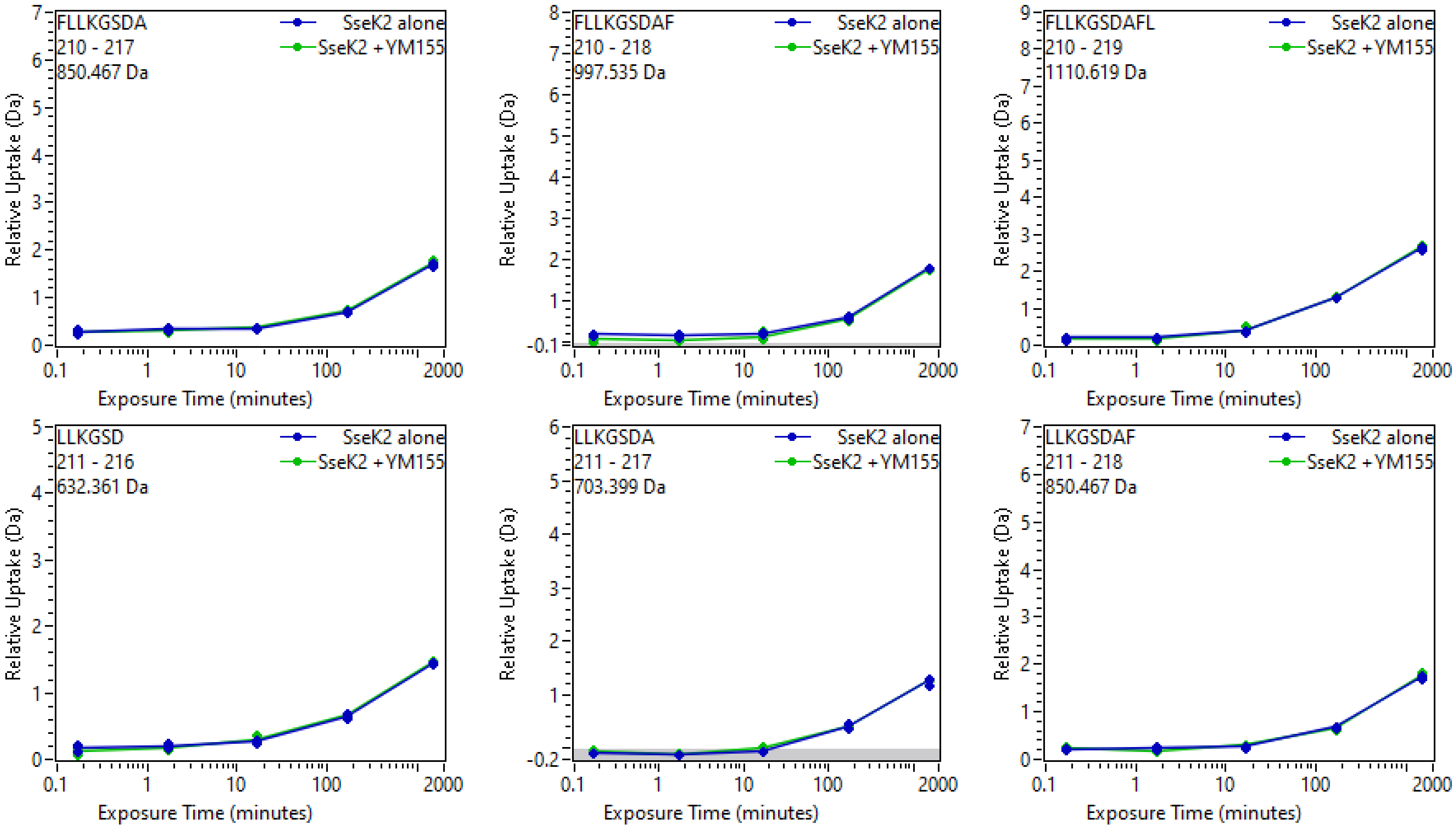
FLLKGSDA
210 - 217
850.467 Da
FLLKGSDAF
210 - 218
997.535 Da
FLLKGSDAFL
210 - 219
1110.619 Da
LLKGSD
211 - 216
632.361 Da
LLKGSDA
211 - 217
703.399 Da
LLKGSDAF
211 - 218
850.467 Da
SseK2 alone
SseK2 +YM155
Relative Uptake (Da)
Exposure Time (minutes)
0.1
1
10
100
2000

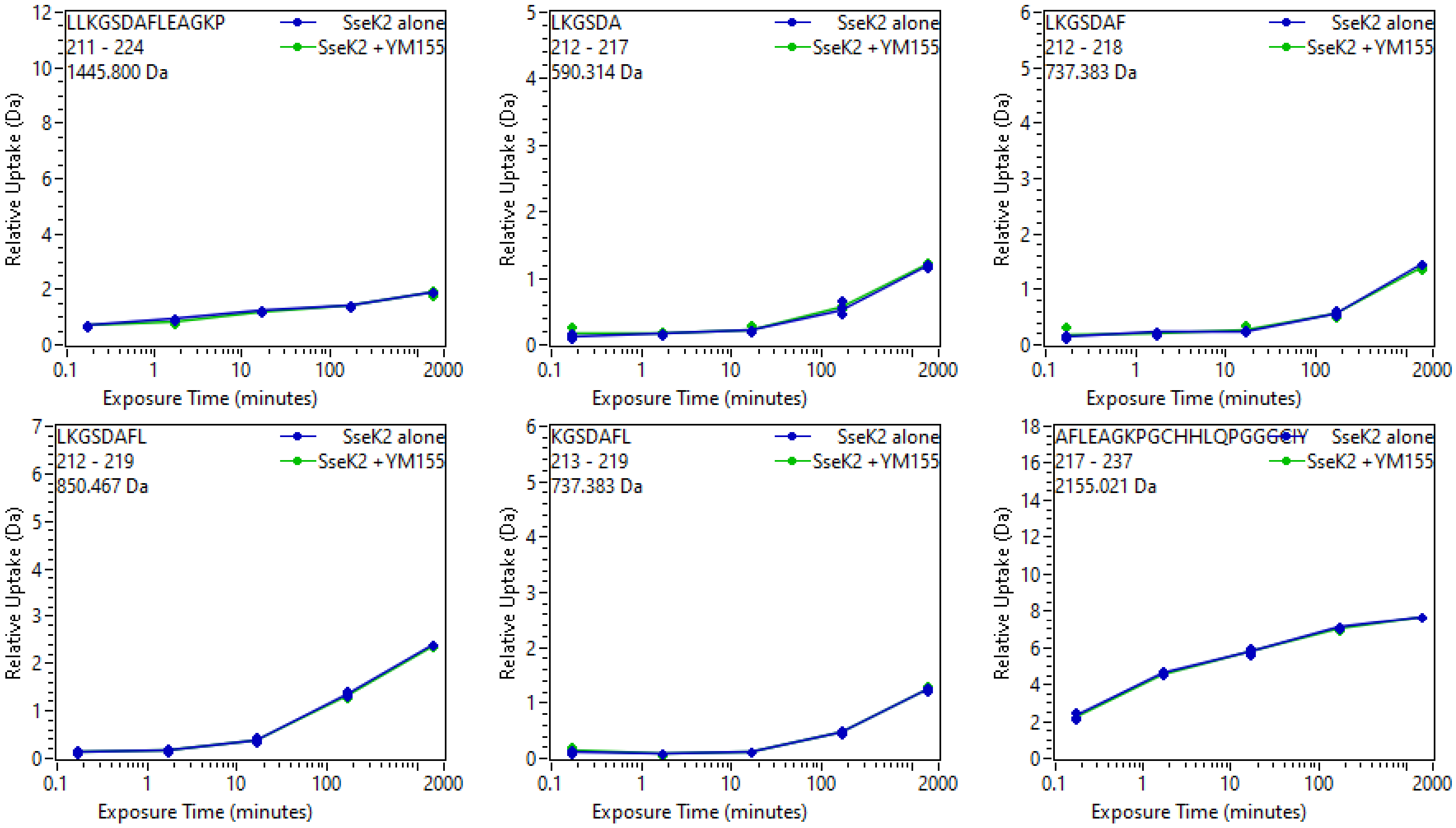
LLKGSDAFLEAGKP
211 - 224
1445.800 Da
SseK2 alone
SseK2 +YM155
LKGSDA
212 - 217
590.314 Da
LKGSDAF
212 - 218
737.383 Da
LKGSDAFL
212 - 219
850.467 Da
KGSDAFL
213 - 219
737.383 Da
217 - 237
2155.021 Da
Relative Uptake (Da)
Exposure Time (minutes)
0.1
1
10
100
2000

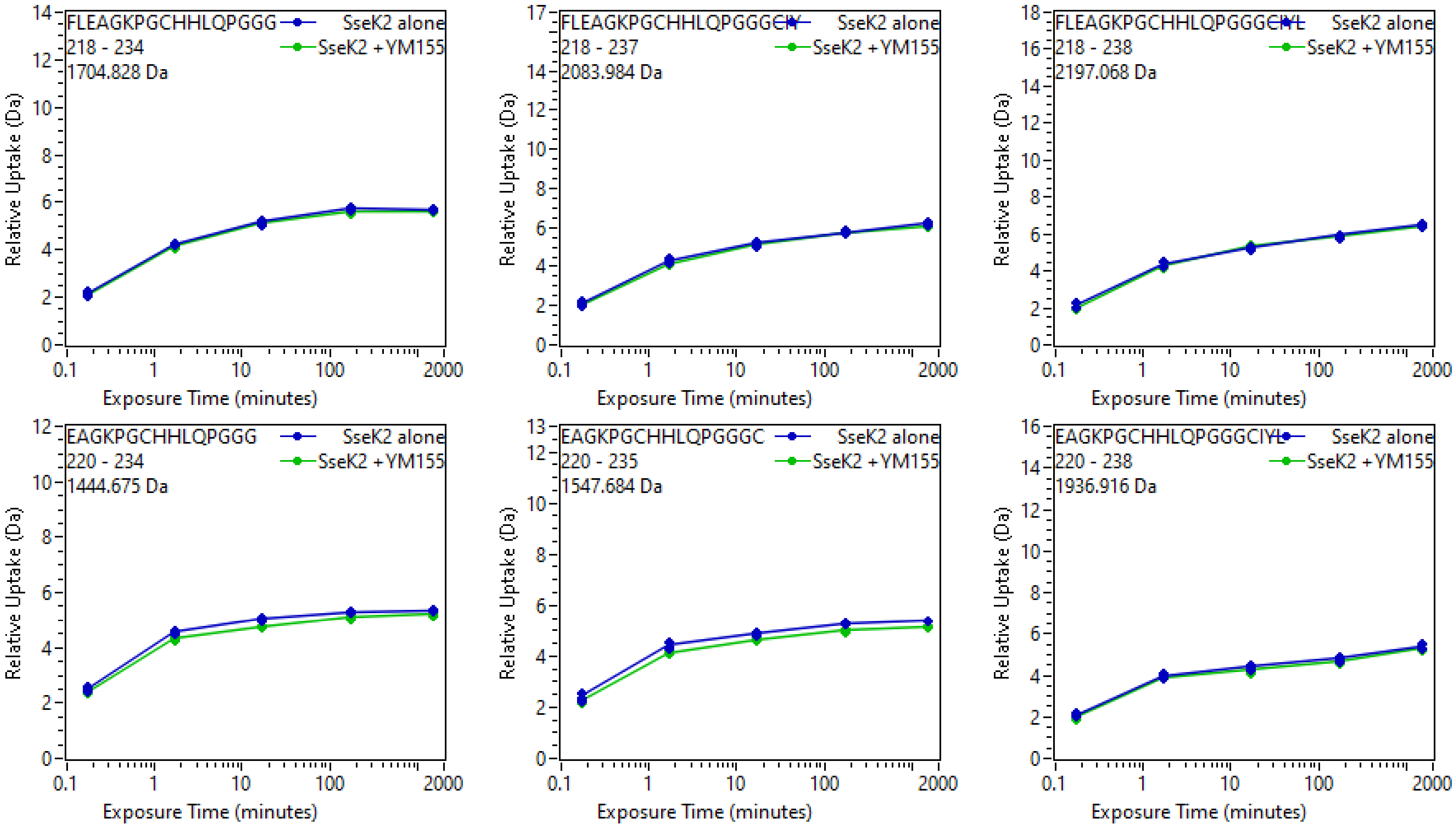
FLEAGKPGCHHLQPGGG
218 - 234
1704.828 Da
FLEAGKPGCHHLQPGGGCIY
218 - 237
2083.984 Da
FLEAGKPGCHHLQPGGGCIYL
218 - 238
2197.068 Da
EAGKPGCHHLQPGGG
220 - 234
1444.675 Da
EAGKPGCHHLQPGGGC
220 - 235
1547.684 Da
EAGKPGCHHLQPGGGCIYL
220 - 238
1936.916 Da
SseK2 alone
SseK2 + YM155
Relative Uptake (Da)
Exposure Time (minutes)
0.1
1
10
100
2000

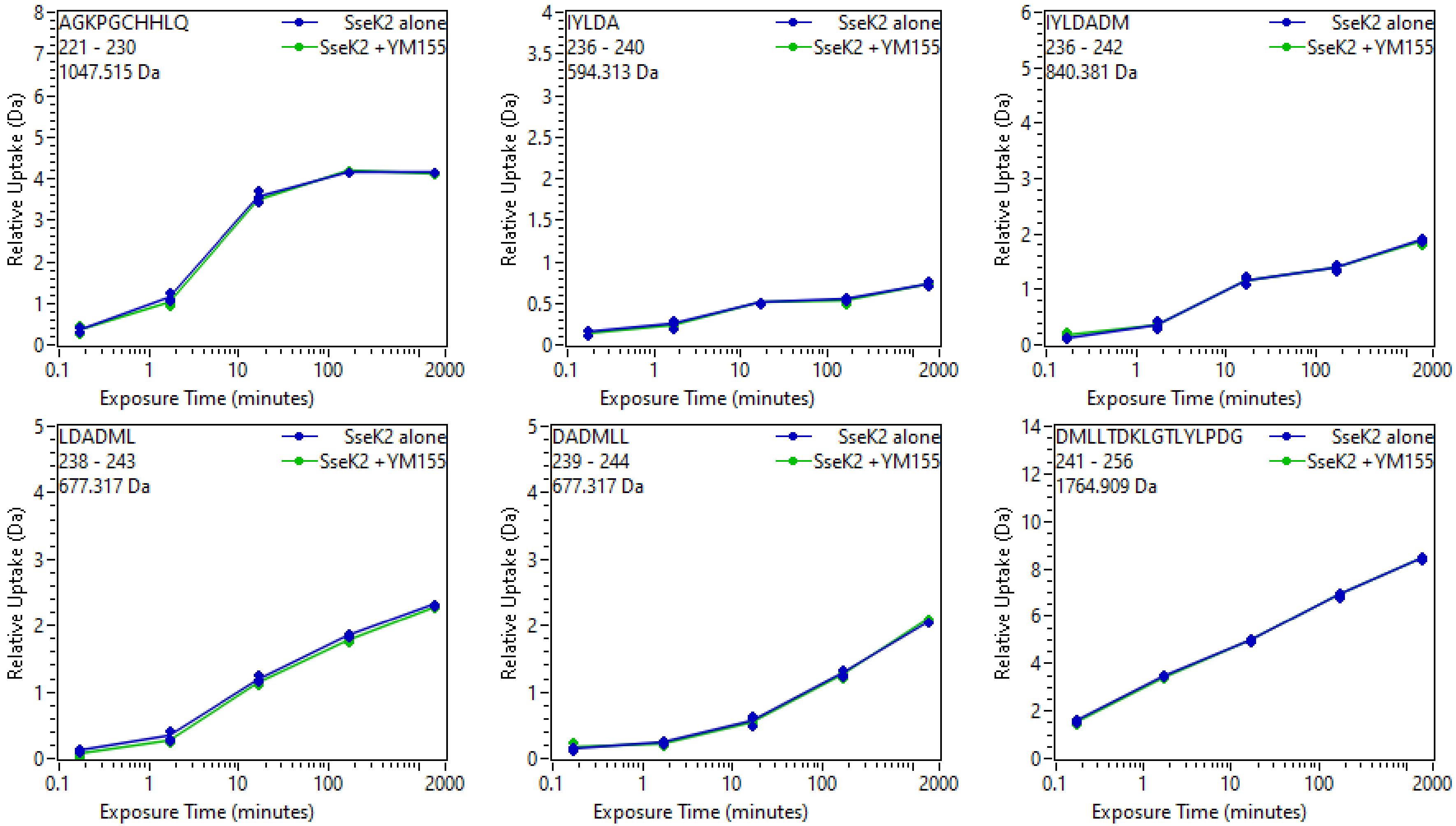
AGKPGCHHLQ
221 - 230
1047.515 Da
SseK2 alone
SseK2 +YM155
Relative Uptake (Da)
Exposure Time (minutes)
IYLDA
236 - 240
594.313 Da
IYLDADM
236 - 242
840.381 Da
LDADML
238 - 243
677.317 Da
DADMLL
239 - 244
677.317 Da
DMLLTDKLGTLYLPDG
241 - 256
1764.909 Da

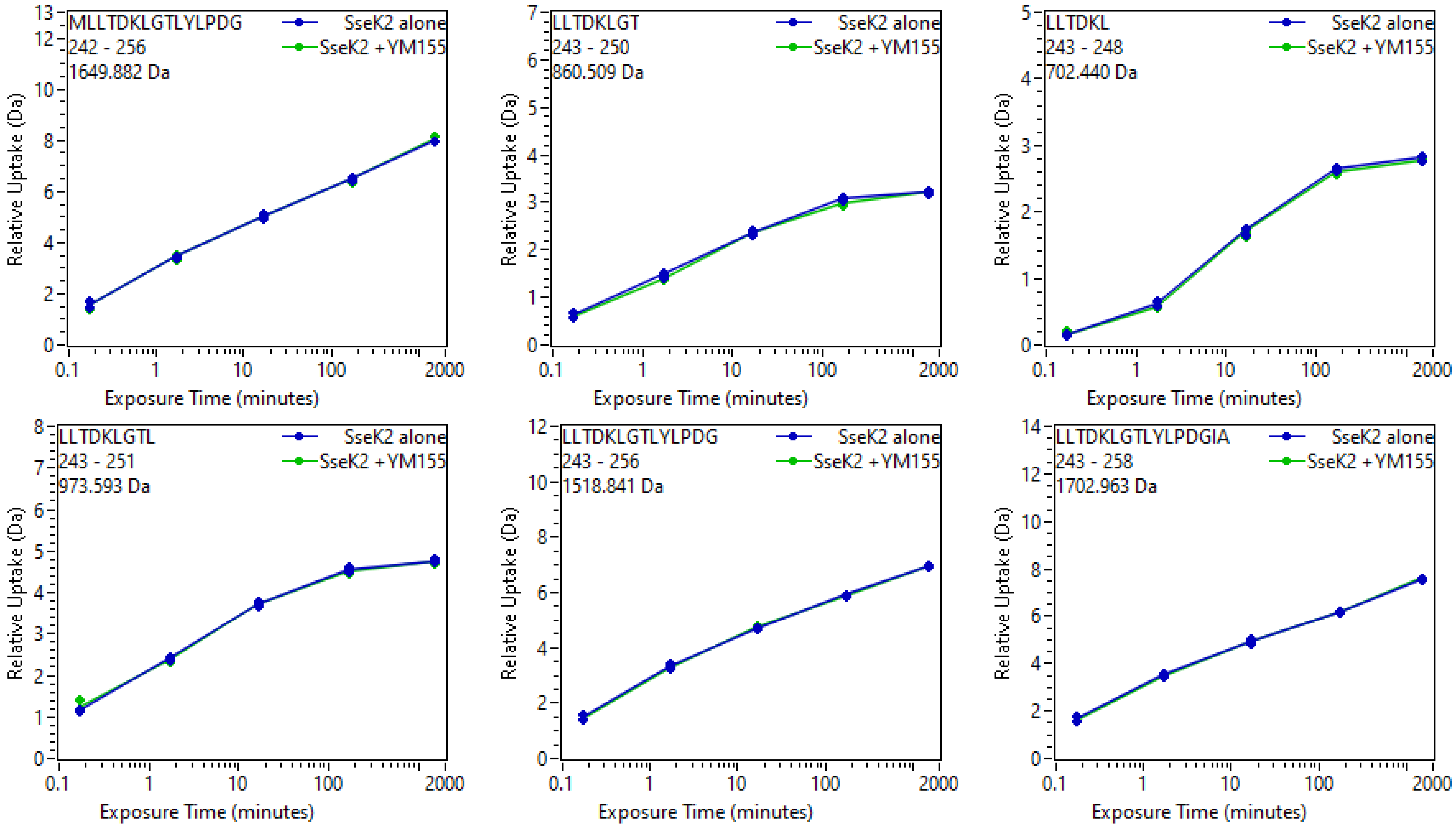
MLLTDKLGTLYLPDG
242 - 256
1649.882 Da
SseK2 alone
SseK2 +YM155
Relative Uptake (Da)
Exposure Time (minutes)
LLTDKLGT
243 - 250
860.509 Da
LLTDKL
243 - 248
702.440 Da
LLTDKLGTL
243 - 251
973.593 Da
LLTDKLGTLYLPDG
243 - 256
1518.841 Da
LLTDKLGTLYLPDGIA
243 - 258
1702.963 Da

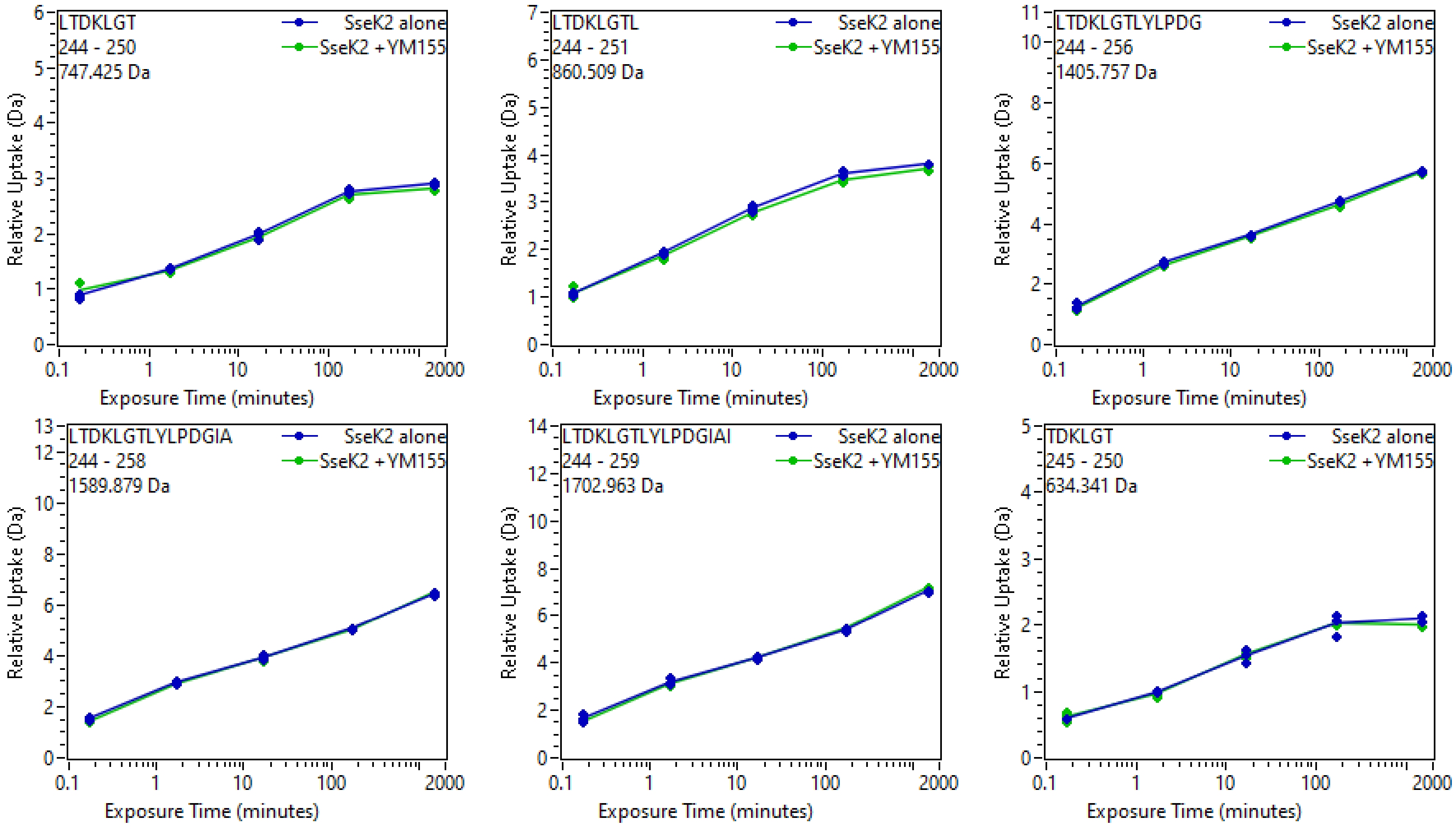
LTDKLGT
244 - 250
747.425 Da
LTDKLGTL
244 - 251
860.509 Da
LTDKLGTLYLPDG
244 - 256
1405.757 Da
LTDKLGTLYLPDGIA
244 - 258
1589.879 Da
LTDKLGTLYLPDGIAI
244 - 259
1702.963 Da
TDKLGT
245 - 250
634.341 Da
SseK2 alone
SseK2 +YM155
Relative Uptake (Da)
Exposure Time (minutes)

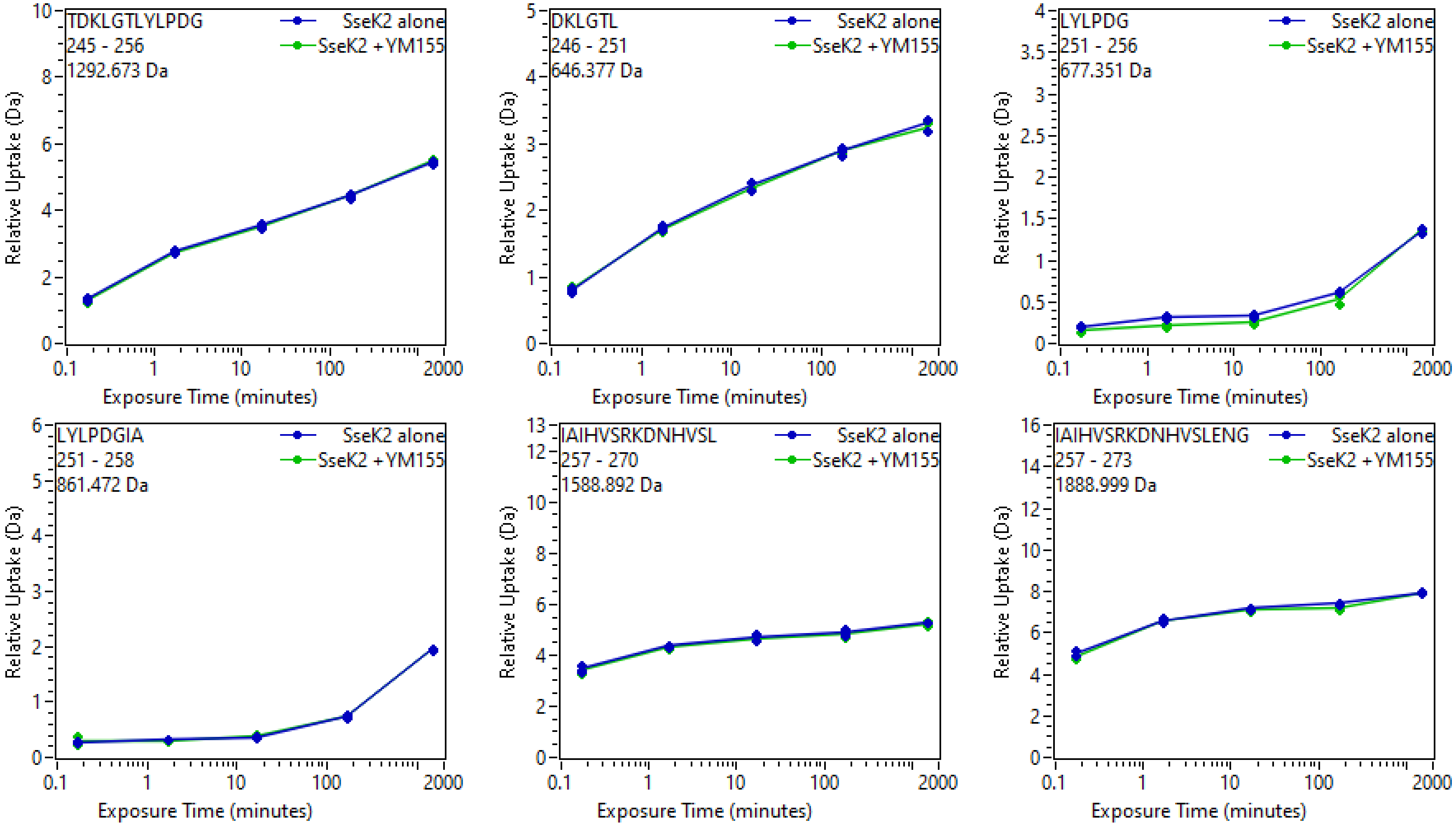

TDKLGTLYLPDG
245 - 256
1292.673 Da
DKLGTL
246 - 251
646.377 Da
LYLPDG
251 - 256
677.351 Da
LYLPDGIA
251 - 258
861.472 Da
IAIHVSRKDNHVSL
257 - 270
1588.892 Da
IAIHVSRKDNHVSLENG
257 - 273
1888.999 Da
SseK2 alone
SseK2 +YM155
Relative Uptake (Da)
Exposure Time (minutes)

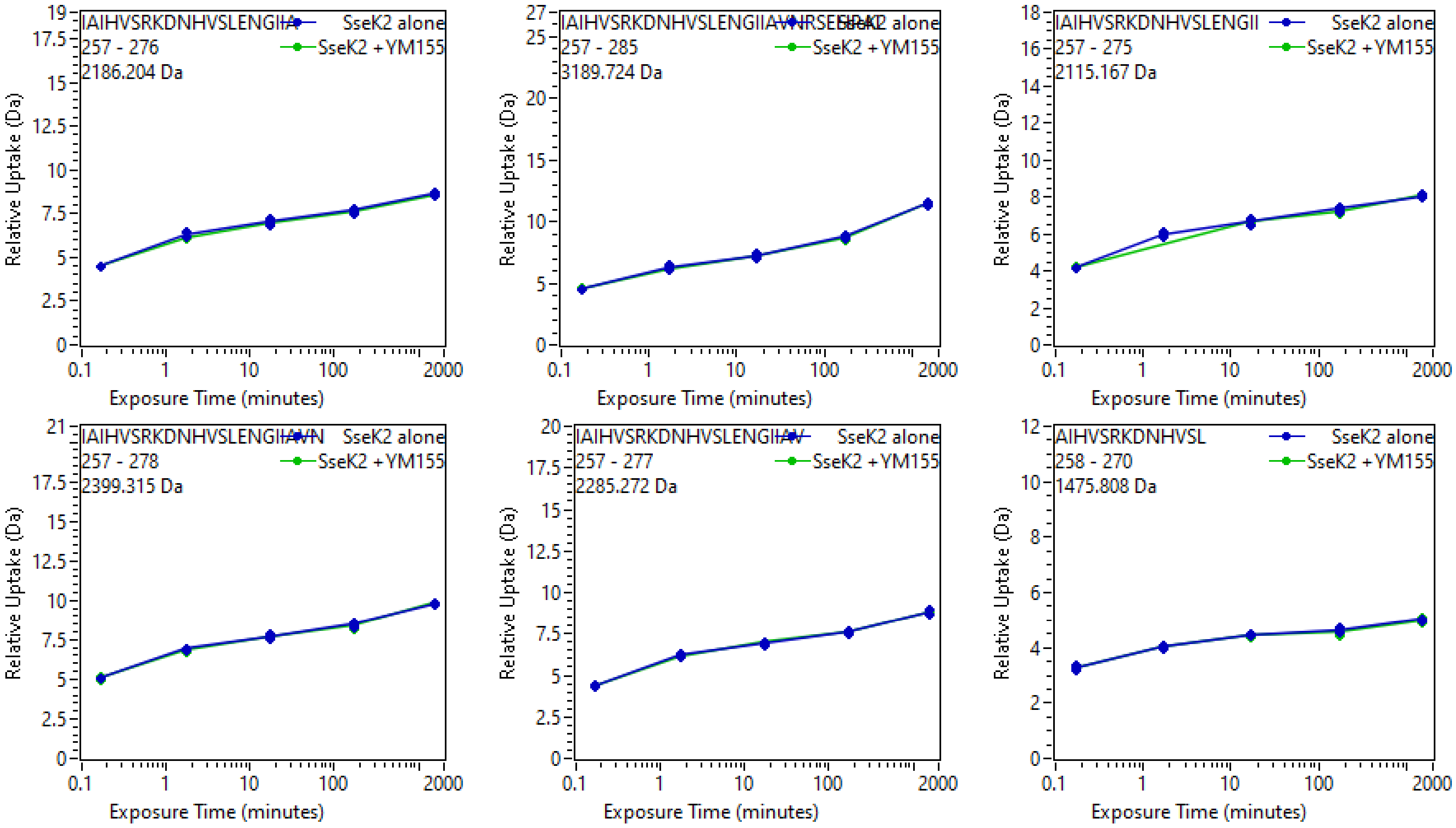
IAIHVSRKDNHVSLENGII
257 - 276
2186.204 Da
SseK2 alone
SseK2 +YM155
IAIHVSRKDNHVSLENGIIA
257 - 285
3189.724 Da
SseK2 alone
SseK2 +YM155
IAIHVSRKDNHVSLENGII
257 - 275
2115.167 Da
SseK2 alone
SseK2 +YM155
IAIHVSRKDNHVSLENGII
257 - 278
2399.315 Da
SseK2 alone
SseK2 +YM155
IAIHVSRKDNHVSLENGII
257 - 277
2285.272 Da
SseK2 alone
SseK2 +YM155
AIHVSRKDNHVSL
258 - 270
1475.808 Da
SseK2 alone
SseK2 +YM155
Relative Uptake (Da)
Exposure Time (minutes)
0.1
1
10
100
2000

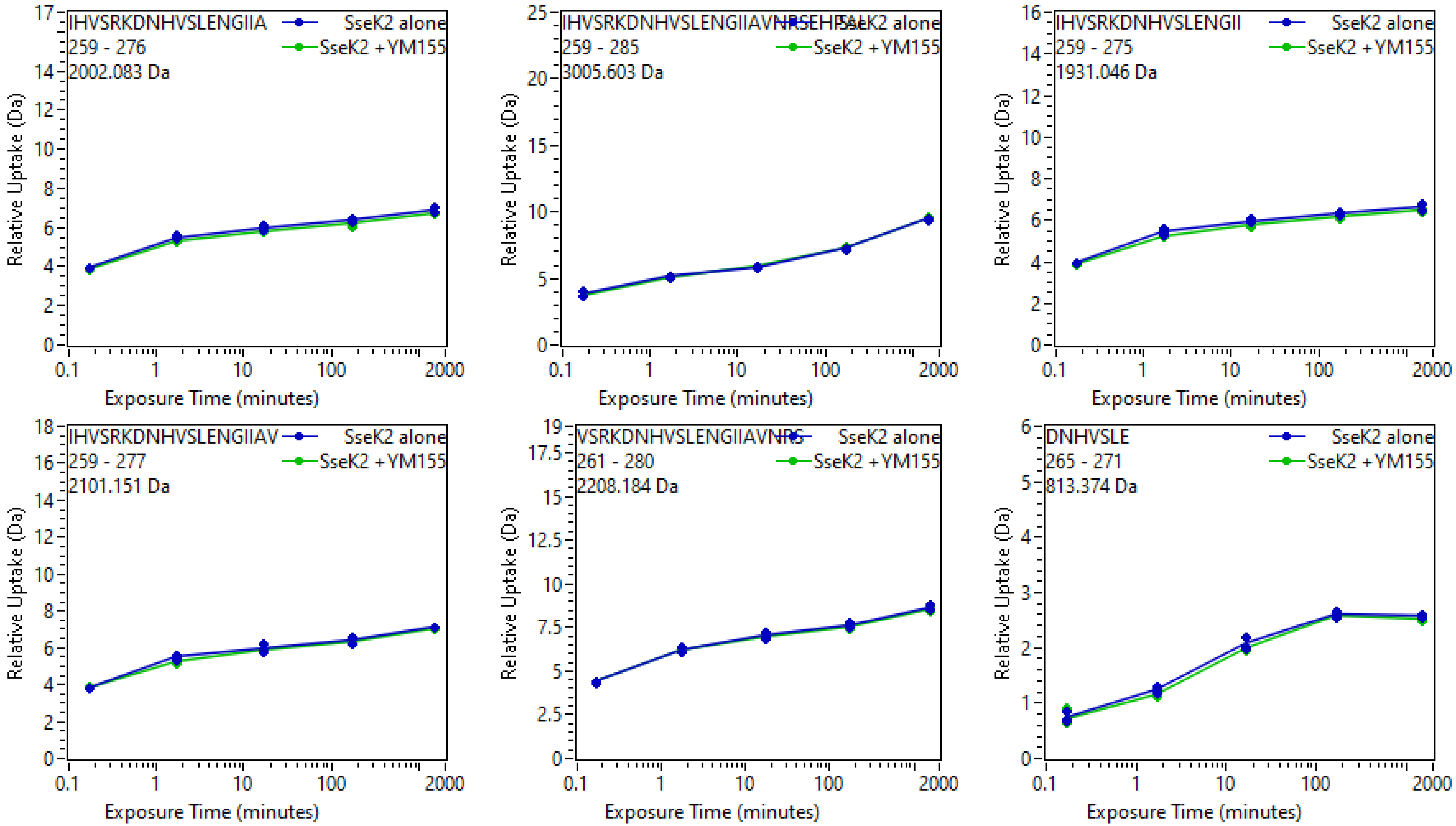
IHVSRKDNHVSLENGIIA
259 - 276
2002.083 Da
SseK2 alone
SseK2 +YM155
Relative Uptake (Da)
Exposure Time (minutes)
IHVSRKDNHVSLENGIIAVNRSEHPSseK2 alone
259 - 285
3005.603 Da
SseK2 +YM155
IHVSRKDNHVSLENGII
259 - 275
1931.046 Da
IHVSRKDNHVSLENGIIAV
259 - 277
2101.151 Da
VSRKDNHVSLENGIIAVNRS
261 - 280
2208.184 Da
DNHVSLE
265 - 271
813.374 Da

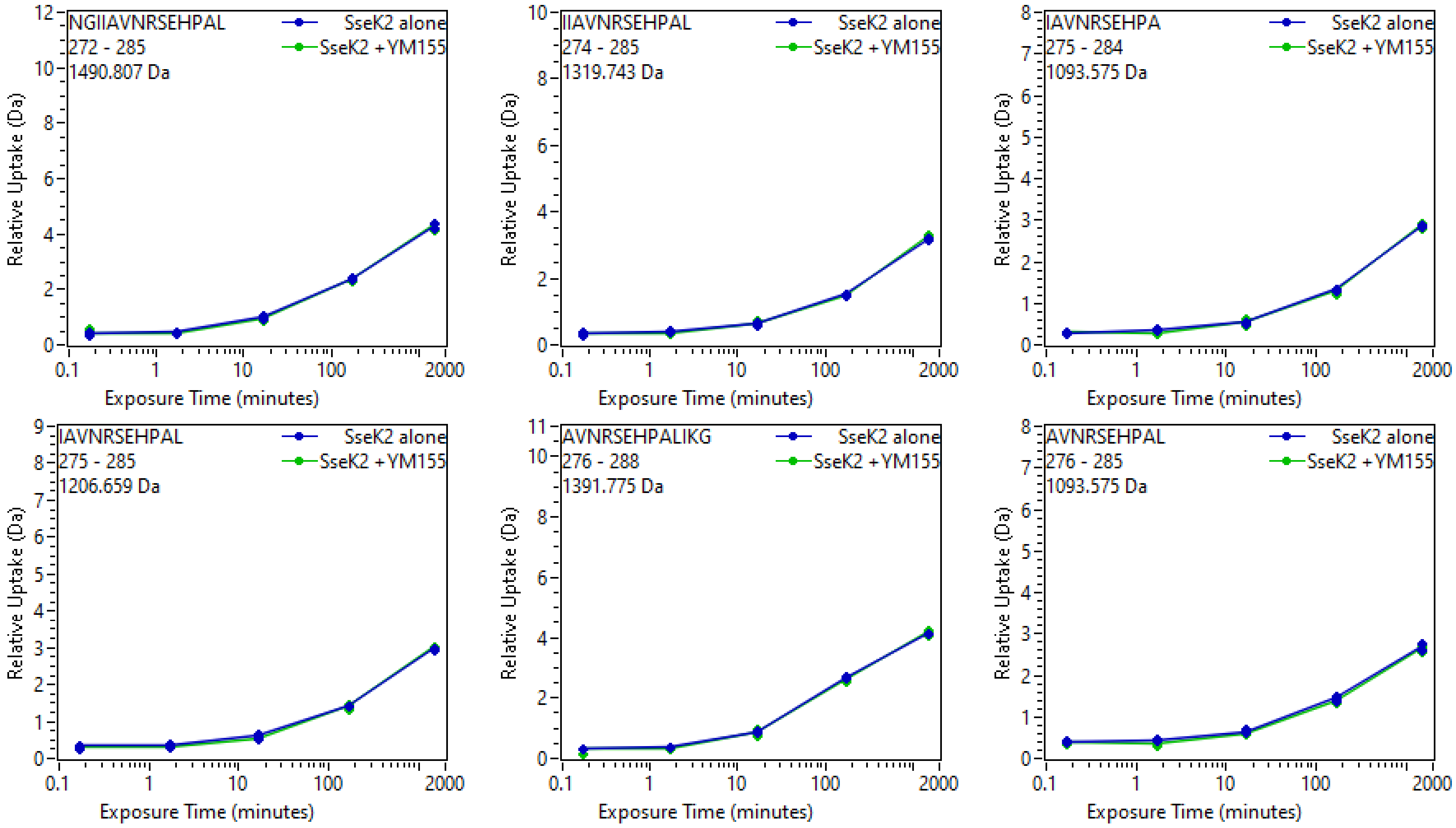
NGIIAVNRSEHPAL
272 - 285
1490.807 Da
SseK2 alone
SseK2 +YM155
Relative Uptake (Da)
Exposure Time (minutes)
IIAVNRSEHPAL
274 - 285
1319.743 Da
IAVNRSEHPA
275 - 284
1093.575 Da
IAVNRSEHPAL
275 - 285
1206.659 Da
AVNRSEHPALIKG
276 - 288
1391.775 Da
AVNRSEHPAL
276 - 285
1093.575 Da
0.1
1
10
100
2000

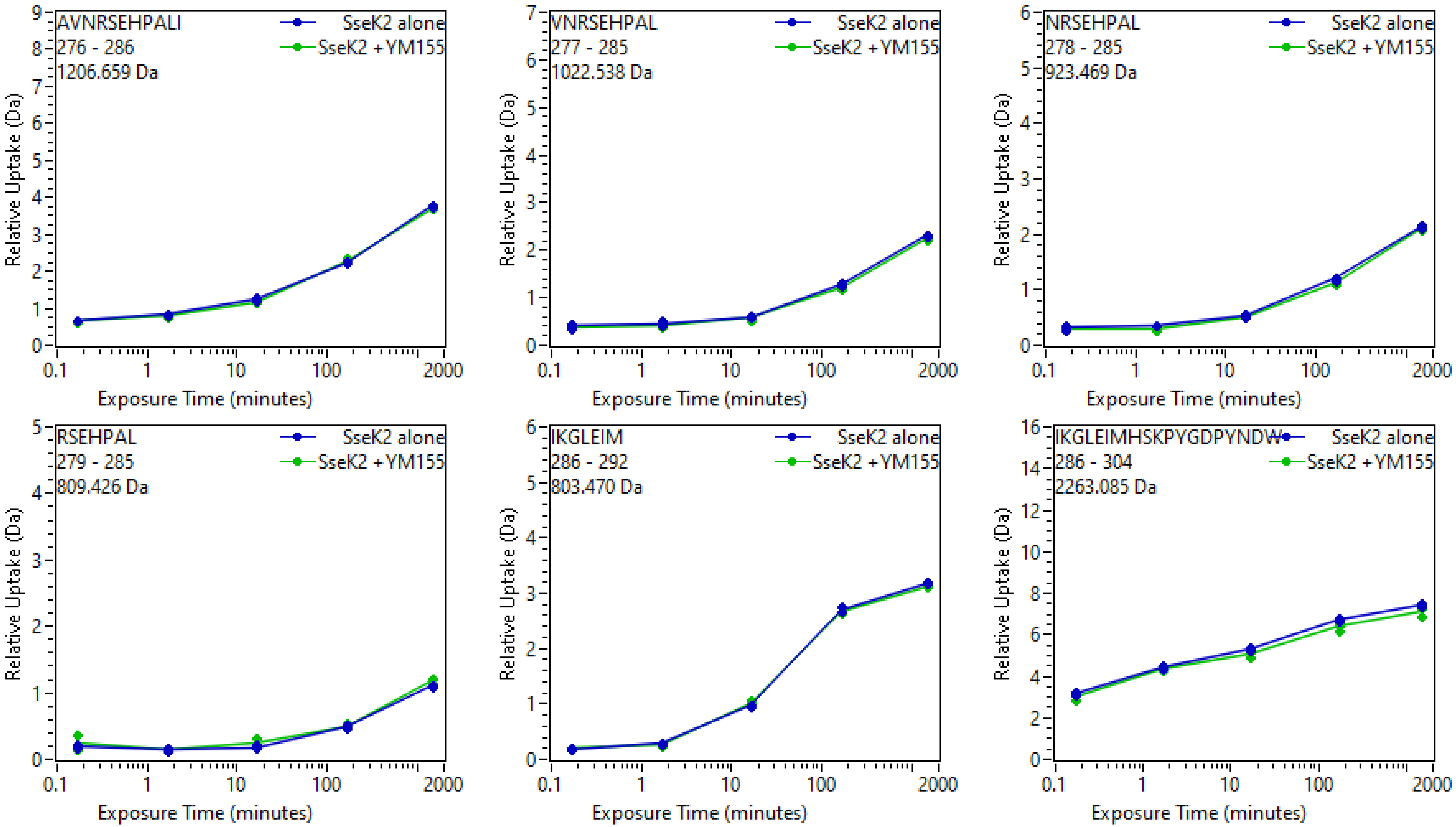
AVNRSEHPALI
276 - 286
1206.659 Da
VNRSEHPAL
277 - 285
1022.538 Da
NRSEHPAL
278 - 285
923.469 Da
RSEHPAL
279 - 285
809.426 Da
IKGLEIM
286 - 292
803.470 Da
IKGLEIMHSKPYGDPYNDW
286 - 304
2263.085 Da
SseK2 alone
SseK2 +YM155
Relative Uptake (Da)
Exposure Time (minutes)

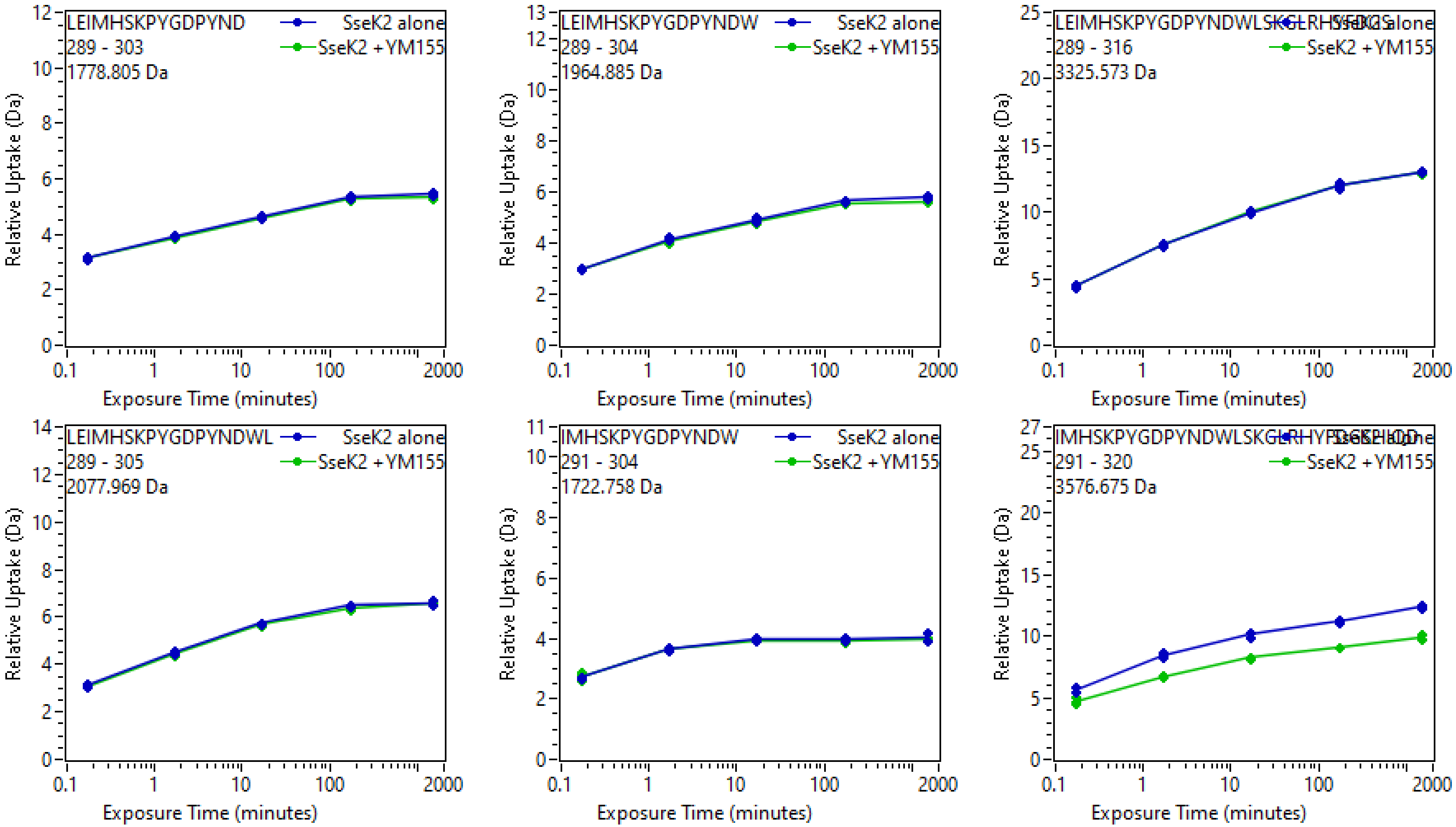
LEIMHSKPYGDPYND
289 - 303
1778.805 Da
SseK2 alone
SseK2 +YM155
LEIMHSKPYGDPYNDW
289 - 304
1964.885 Da
289 - 316
3325.573 Da
LEIMHSKPYGDPYNDWL
289 - 305
2077.969 Da
IMHSKPYGDPYNDW
291 - 304
1722.758 Da
291 - 320
3576.675 Da
Relative Uptake (Da)
Exposure Time (minutes)
0.1
1
10
100
2000

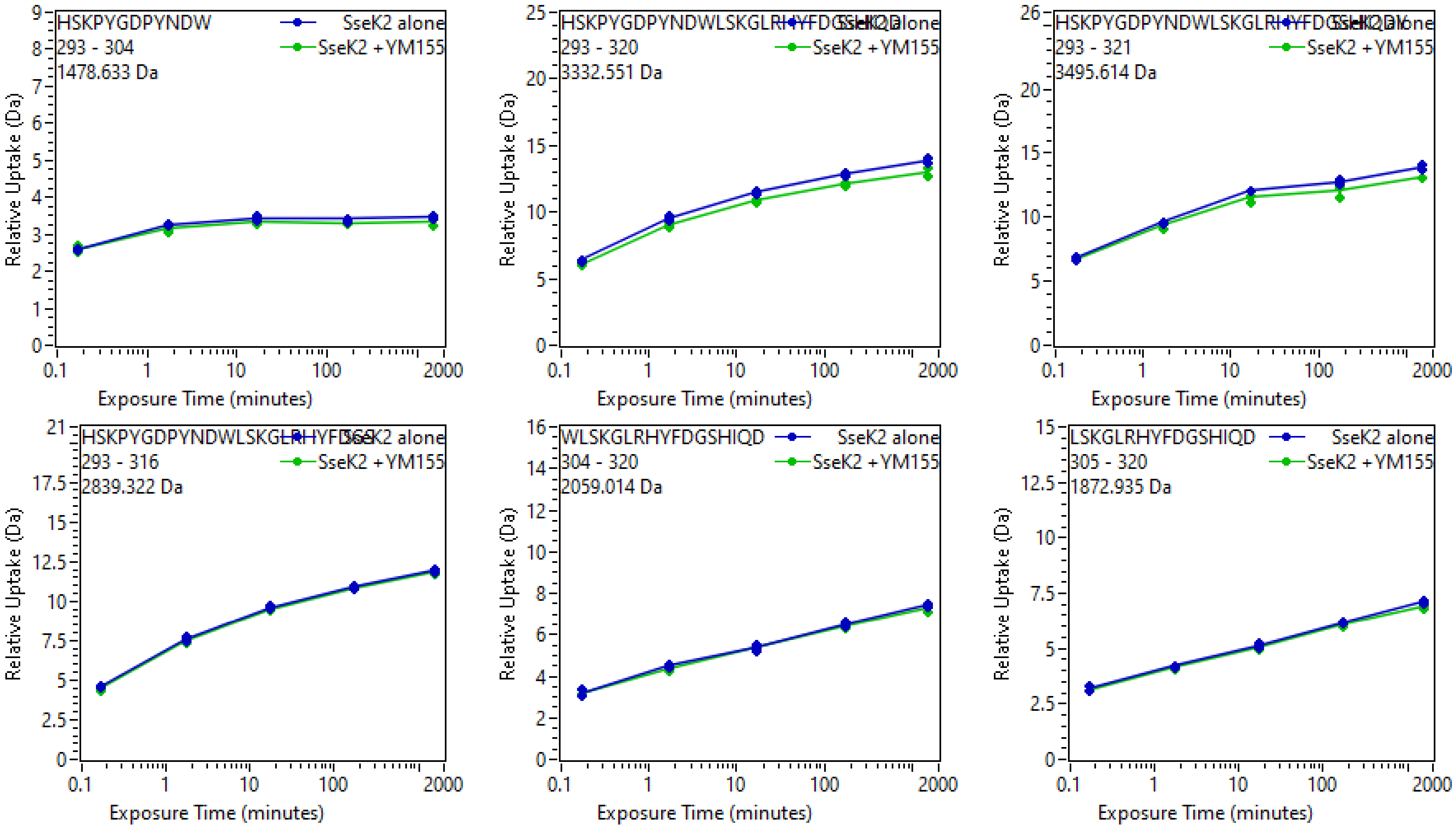
HSKPYGDPYNDW
293 - 304
1478.633 Da
SseK2 alone
SseK2 +YM155
HSKPYGDPYNDWLSKGLRHYFDGSHIQD
293 - 320
3332.551 Da
HSKPYGDPYNDWLSKGLRHYFDGSHIQDV
293 - 321
3495.614 Da
HSKPYGDPYNDWLSKGLRHYFDG
293 - 316
2839.322 Da
WLSKGLRHYFDGSHIQD
304 - 320
2059.014 Da
LSKGLRHYFDGSHIQD
305 - 320
1872.935 Da
Relative Uptake (Da)
Exposure Time (minutes)

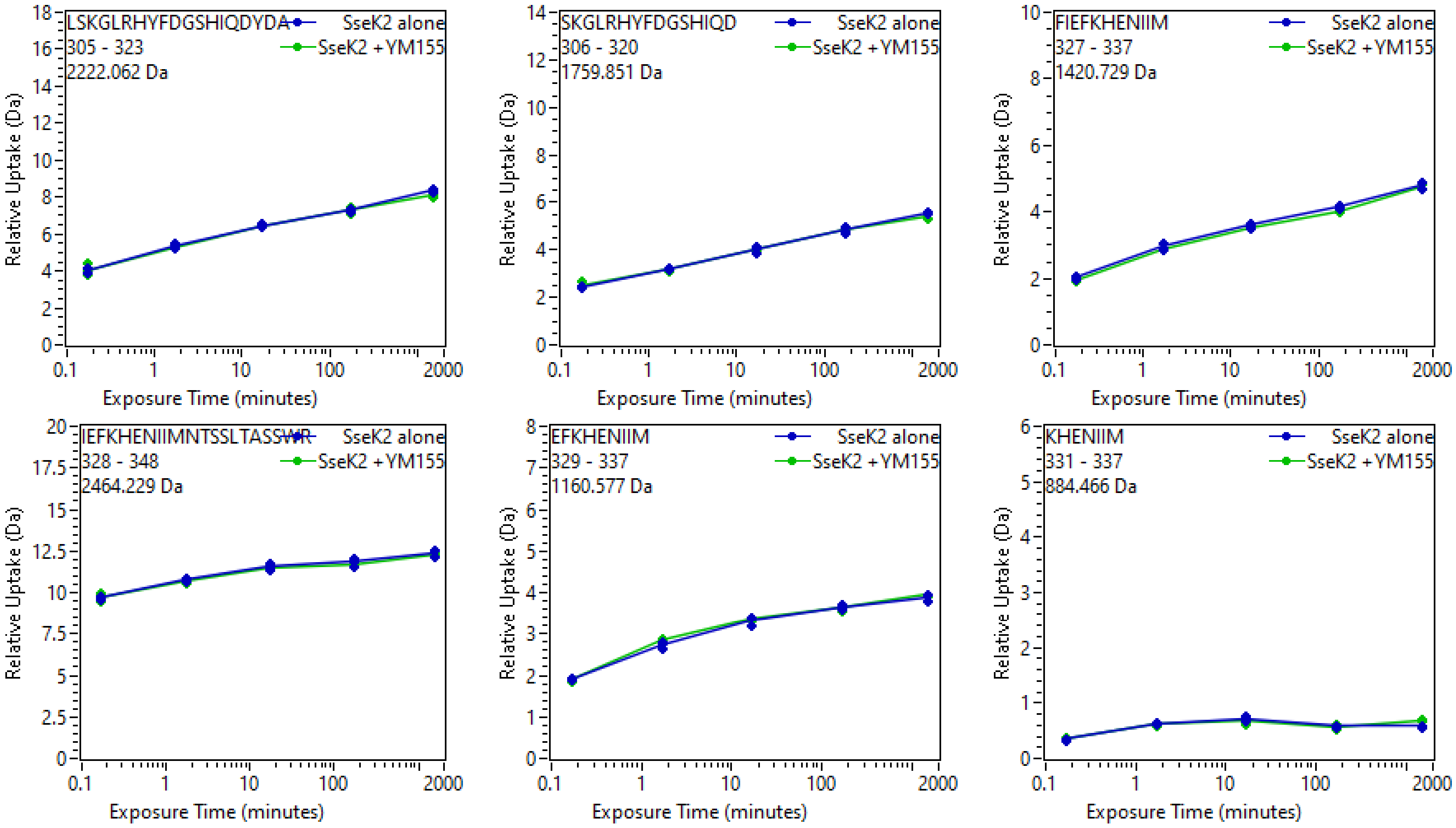
LSKGLRHYFDGSHIQDYDA
305 - 323
2222.062 Da
SKGLRHYFDGSHIQD
306 - 320
1759.851 Da
FIEFKHENIIM
327 - 337
1420.729 Da
IEFKHENIIMNTSSLTASSWR
328 - 348
2464.229 Da
EFKHENIIM
329 - 337
1160.577 Da
KHENIIM
331 - 337
884.466 Da
SseK2 alone
SseK2 +YM155
Relative Uptake (Da)
Exposure Time (minutes)
0.1
1
10
100
2000

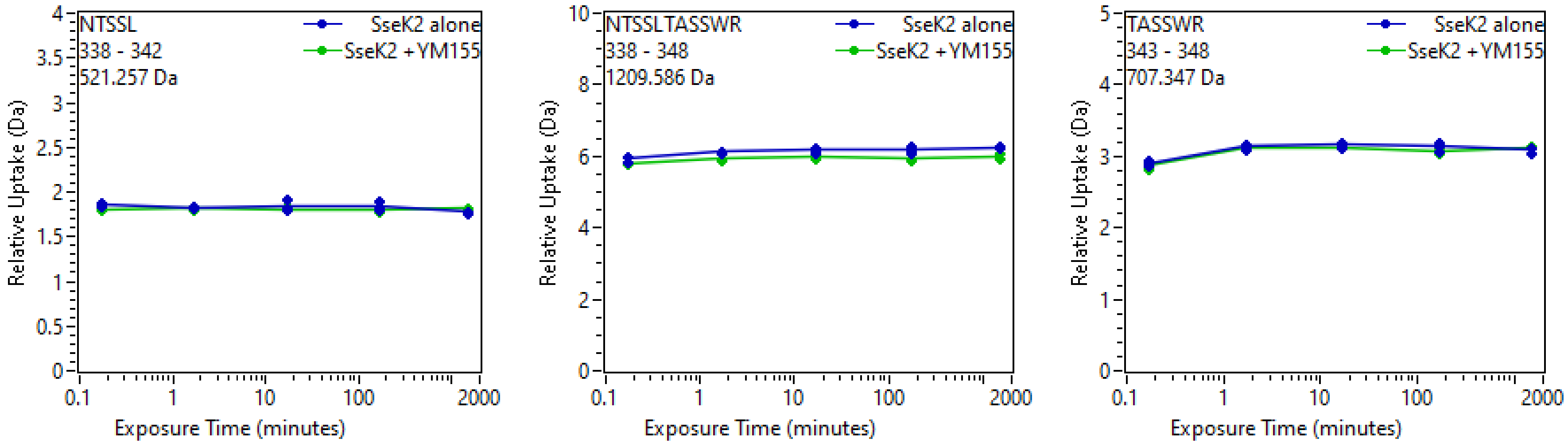

Figure S12. Deuterium uptake plot of each individual peptide whose HDX was followed for the SseK2 HDX-MS analysis.
